# Potential role of glucosamine-phosphate N-acetyltransferase 1 in the development of lung adenocarcinoma

**DOI:** 10.18632/aging.202604

**Published:** 2021-03-03

**Authors:** Shengqiang Zhang, Hongyan Zhang, Huawei Li, Jida Guo, Jun Wang, Linyou Zhang

**Affiliations:** 1 Department of Thoracic Surgery, The Second Affiliated Hospital of Harbin Medical University, Harbin 150086, Heilongjiang, People’s Republic of China; 2 Department of Physiology and Neurobiology, Mudanjiang Medical University, Mudanjiang 157000, Heilongjiang, People’s Republic of China

**Keywords:** GNPNAT1, expression, tumor immune microenvironment, prognosis, lung adenocarcinoma

## Abstract

Glucosamine-phosphate N-acetyltransferase 1 (GNPNAT1) is a key enzyme associated with glucose metabolism and uridine diphosphate-N-acetylglucosamine biosynthesis. Abnormal *GNPNAT1* expression might be associated with carcinogenesis. We analyzed multiple lung adenocarcinoma (LUAD) gene expression databases and verified *GNPNAT1* higher expression in LUAD tumor tissues than in normal tissues. Moreover, we analyzed the survival relationship between LUAD patients’ clinical status and *GNPNAT1* expression, and found higher *GNPNAT1* expression in LUAD patients with unfavorable prognosis. We built *GNPNAT1* gene co-expression networks and further annotated the co-expressed genes’ Gene Ontology (GO) terms, Kyoto Encyclopedia of Genes and Genomes (KEGG) pathways, and various associated regulatory factors. These co-expression genes’ functional networks mainly participate in chromosome segregation, RNA metabolic process, and RNA transport. We analyzed *GNPNAT1* genetic alterations and co-occurrence networks, and the functional networks of these genes showed that *GNPNAT1* participates in multiple steps of cell cycle transition and in the development of some cancers. We assessed the correlation between *GNPNAT1* expression and cancer immune infiltrates and showed that *GNPNAT1* expression is correlated with several immune cells, chemokines, and immunomodulators in LUAD. We found that *GNPNAT1* correlates with LUAD development and prognosis, laying a foundation for further research, especially in immunotherapy.

## INTRODUCTION

Lung cancer is the second most common malignant tumor and the leading cause of death in all carcinomas [1]. Nearly 85% of lung cancer patients have non-small-cell lung cancer (NSCLC) [2]. Lung adenocarcinoma (LUAD) is the primary histological subtype in NSCLC and is more likely in young, female patients [3, 4]. In recent years, LUAD morbidity has been increasing, with a five-year survival rate below 15% [5]. Although LUAD pathogenesis studies have shown significant progress, there are still several problems that need to be resolved. Therefore, there is an urgent need to identify new diagnoses and therapeutic molecular markers of LUAD.

Glucosamine-phosphate N-acetyltransferase 1 (GNPNAT1) is a key enzyme associated with uridine diphosphate-N-acetylglucosamine biosynthesis. It can participate in insulin secretion and also influence cell cycle progression and cell apoptosis [6]. If *GNPNAT1* is deficient or inactivated, the cell cycle is delayed, and subsequently cells die [7]. One of the hallmarks of tumor cells is increased metabolism, included glucose metabolism, fatty acid metabolism, amino acid metabolism, and nucleotide synthesis metabolism [8]. Zhao et al. reported that underexpression of *GNPNAT1* in lung cancer A549 cells resulted in inhibited tumor cell adhesion and infiltration [9]. However, further research is needed to explore the role of *GNPNAT1* as a tumor metabolism gene, whether it is an influencing factor in LUAD progression, and the related mechanisms.

Our study aimed to elucidate the potential role of *GNPNAT1* in the occurrence and development of LUAD. Moreover, we aimed to provide new insights to support diagnosis and therapy for LUAD.

## RESULTS

### The expression of GNPNAT1 in LUAD

**Figure 1:**
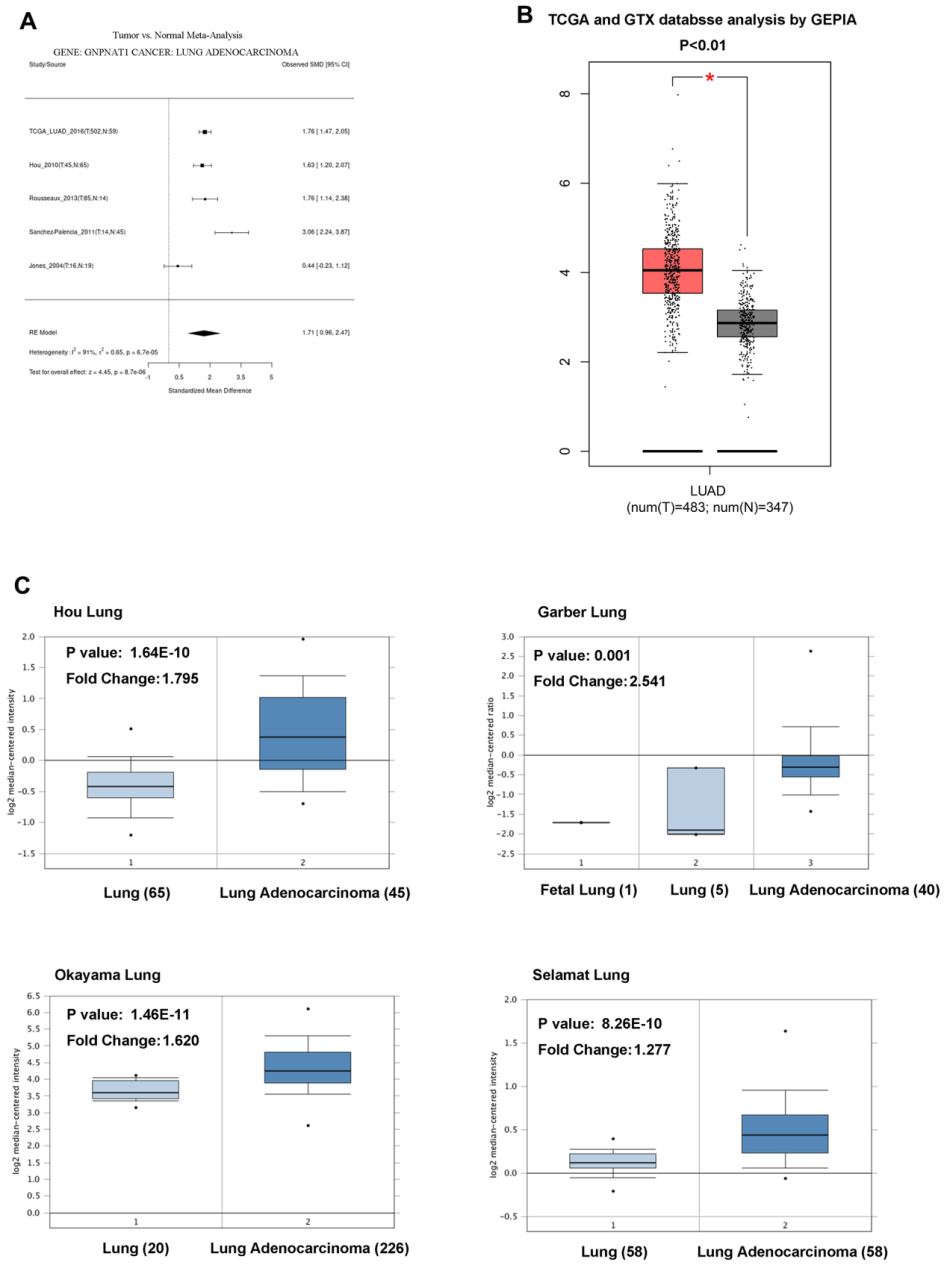
**GNPNAT1 transcription level in LUAD.** (**A**) The forest plot shows *GNPNAT1* expression level meta-analysis of LUAD tumor tissues and normal tissues in five different LUAD cohorts (LCE). (**B**) The box plot shows *GNPNAT1* mRNA expression levels of LUAD tumor tissues and normal tissues in the TCGA (GEPIA) datasets. (**C**) The box plot shows *GNPNAT1* mRNA expression levels of LUAD tumor tissues and normal tissues in the Garber Lung, Okayama Lung, Selamat Lung, and Hou Lung datasets (Oncomine), respectively.

We obtained data regarding the expression of *GNPNAT1* in LUAD and normal lung tissues from the Cancer Genome Atlas (TCGA) and the Gene Expression Omnibus (GEO) database. There were five cohort meta-analyses of the differential expression of *GNPNAT1* in the Lung Cancer Explorer (LCE) database, showing that the mRNA *GNPNAT1* expression level was significantly higher in LUAD tumor tissues than in normal tissues (Figure 1A). We obtained similar results for the mRNA *GNPNAT1* expression features using the Gene Expression Profiling Interactive Analysis (GEPIA) database (Figure 1B) and Oncomine database (Figure 1C).

**Figure 2:**
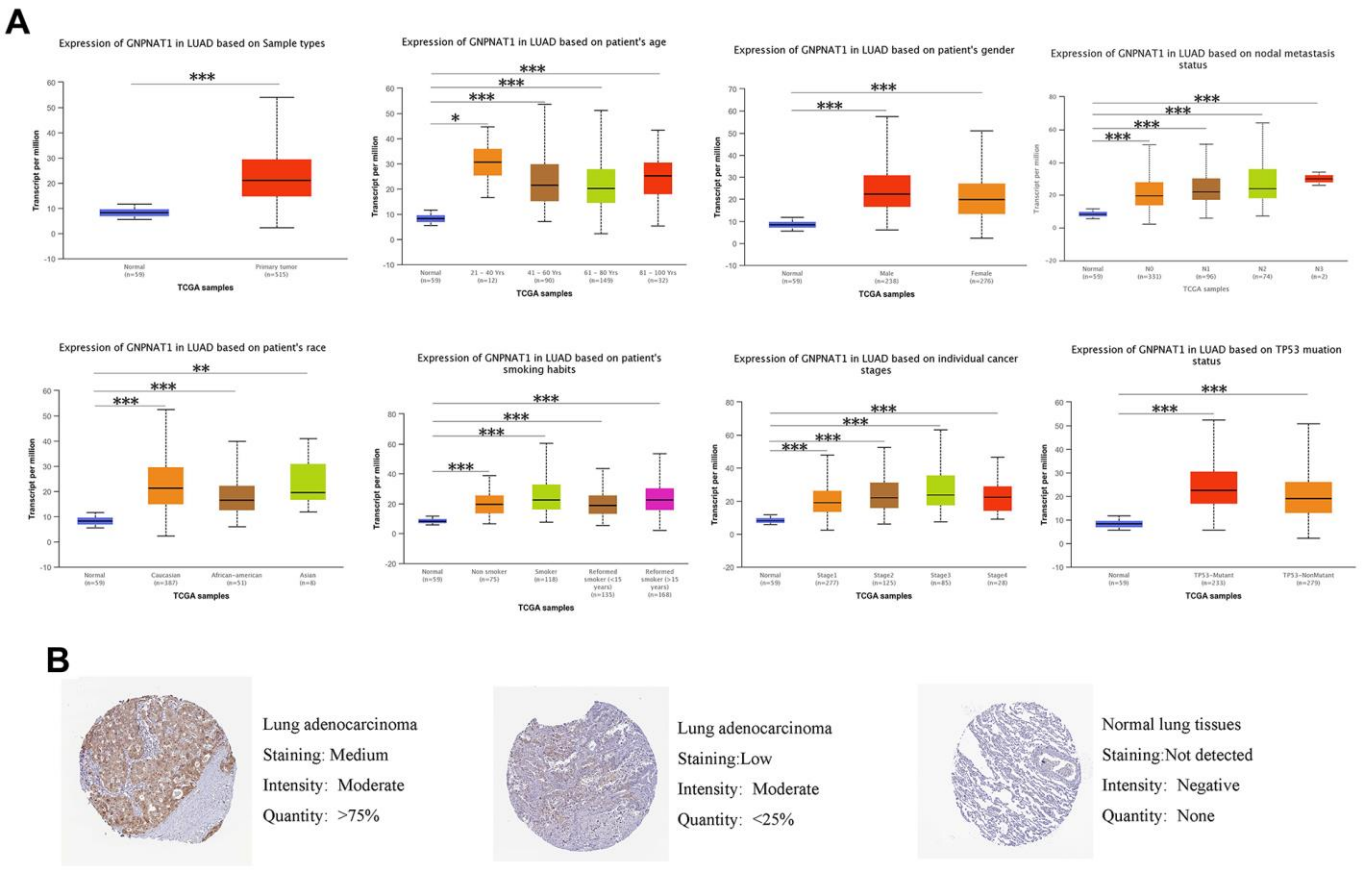
**GNPNAT1 transcription and proteomics level in LUAD patients.** (**A**) *GNPNAT1* transcription level in normal and LUAD samples, and in subgroups of LUAD patients stratified by age, nodal metastasis, gender, race, smoking status, stage, and TP53 mutant status (UALCAN). The central mark is the median; the edges of the box are the 25th and 75th percentiles. The t-test was used to estimate the significance of difference in gene expression levels between groups. *, *p* < 0.05; **, *p* < 0.01; ***, *p* < 0.001. (**B**) Expression of *GNPNAT1* in LUAD tumor specimens and normal specimens from the human protein profiles database (HPA).

We used the UALCAN database to identify the mRNA *GNPNAT1* expression in TCGA LUAD samples, grouped by characteristics such as age, sex, stage, race, and smoking status. (Figure 2A). In all subgroups, the mRNA *GNPNAT1* expression level was higher in LUAD tumor tissues than in normal tissues. Therefore, *GNPNAT1* might be a potential biomarker for the diagnosis of LUAD.

To further assess *GNPNAT1* expression levels in LUAD, we also detected *GNPNAT1* protein levels in LUAD tissues and normal tissues by immunohistochemical staining in the Human Protein Atlas (HPA) database. As shown in Figure 2B, *GNPNAT1* protein expression in cell cytoplasmic/membranous staining intensity was moderate or low in LUAD tissues, but it was not detected in normal tissues.

### GNPNAT1 expression associated with survival in LUAD

**Figure 3:**
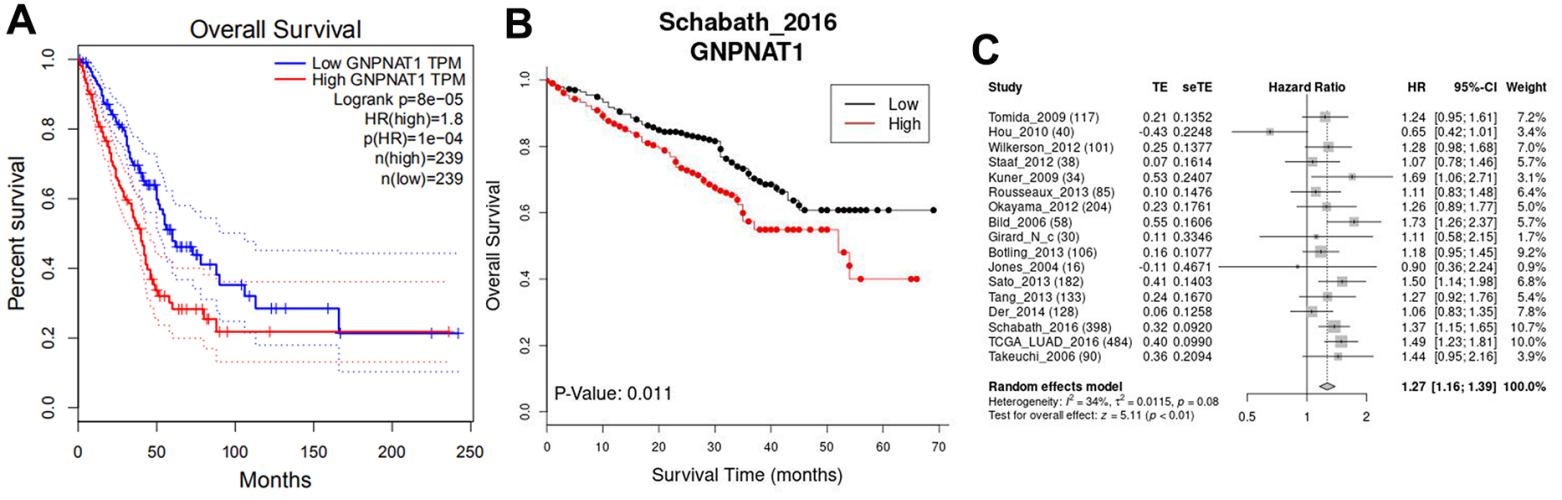
**GNPNAT1 expression was associated with the survival in LUAD.** (**A**) Overall survival (OS) in the TCGA cohort (GEPIA). (**B**) OS in GSE72094 (Schabath_2016) cohort (LCE). (**C**) The forest plot shows 17 cohorts meta-analysis OS (LCE).

This study aimed to determine the *GNPNAT1* expression associated with survival in patients with LUAD. Kaplan-Meier survival curves were used to identify survival outcomes in the multiple LUAD cohorts. The median value of *GNPNAT1* expression was the cutoff value, and each LUAD cohort was divided into a high and a low *GNPNAT1* expression group. Compared to the low *GNPNAT1* expression group, the high *GNPNAT1* expression group had significantly poorer overall survival (OS) (log-rank test, *p* < 0.05) in the LUAD TCGA cohort (Figure 3A) and in the GSE72094 cohort (Figure 3B). From the LCE database, we got a 17 cohorts meta-analysis of *GNPNAT1* expression associated with survival in LUAD; Figure 3C shows that the high *GNPNAT1* expression group had poorer OS (test for all cohorts, *p* < 0.01, HR = 1.27) compared to the low *GNPNAT1* expression group.

### The co-expression networks of GNPNAT1 in LUAD

**Figure 4:**
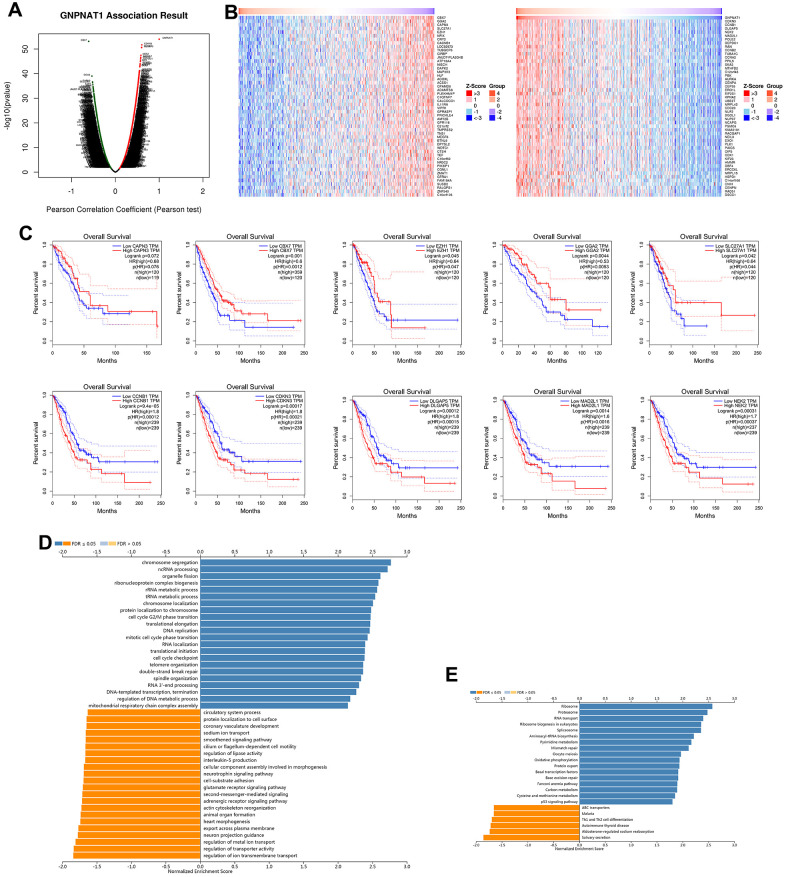
**GNPNAT1 co-expressed genes in LUAD (LinkedOmics).** (**A**) The volcano plot shows the *GNPNAT1* highly correlated genes identified by the Pearson test in the LUAD cohort. (**B**) The heat maps show the top 50 genes positively and negatively correlated with *GNPNAT1* in LUAD. (**C**) Top five genes positively and negatively correlated with *GNPNAT1* associated with survival in LUAD. (**D**) Significantly enriched GO annotations of *GNPNAT1* in the LUAD cohort. (**E**) Significantly enriched KEGG pathways of *GNPNAT1* in the LUAD cohort.

We used LinkedOmics to obtain the *GNPNAT1* co-expression networks in the LUAD TCGA cohorts. There were 4039 positively co-expressed genes and 6654 negatively co-expressed genes with *GNPNAT1* (FDR < 0.01); in Figure 4A, the dark red dots represent positively correlated genes and the dark green dots represent negatively correlated genes. We drew heatmaps with the 50 most significant positively and negatively co-expressed genes, respectively (Figure 4B). All these significantly correlated genes are shown in Supplementary Table 1.

The five most significant genes positively associated with *GNPNAT1* expression were cyclin dependent kinase inhibitor 3 (CDKN3), cyclin B1 (CCNB1), DLG associated protein 5 (DLGAP5), NIMA related kinase 2 (NEK2), and mitotic arrest deficient 2 like 1 (MAD2L1). The five most significant negatively associated genes were chromobox 7(CBX7), Golgi-associated, gamma adaptin ear-containing, and ARF-binding protein 2 (GGA2), calpain 3 (CAPN3), solute carrier family 27 member 1 (SLC27A1), and enhancer of zeste 1 polycomb repressive complex 2 subunit (EZH1). We used GEPIA to identify the 10 genes associated with OS in LUAD. Kaplan-Meier survival curves are shown in Figure 4C. All the five positive high expressions showed significant risk genes in LUAD (*p* < 0.05); conversely, the five negative high expressions showed protective genes in LUAD (*p* < 0.05; except CAPN3, *p* = 0.072).

We used gene set enrichment analysis (GSEA) to annotate *GNPNAT1* co-expressed genes with a significant GO term. It showed that *GNPNAT1* co-expressed genes are mainly involved in chromosome segregation, ncRNA processing, organelle fission, ribonucleoprotein complex biogenesis, rRNA and tRNA metabolic process, etc., and in inhibited ion transmembrane transport, transporter activity, metal ion transport, neuron projection guidance, export across plasma membrane, and so forth (Figure 4D and Supplementary Table 2). KEGG pathway analysis showed enrichment in the ribosome, proteasome, RNA transport, ribosome biogenesis in eukaryotes, spliceosome, and so forth (Figure 4E and Supplementary Table 3).

### GNPNAT1 networks of kinase, miRNA, and transcription factor targets in LUAD

To further explore the targets of *GNPNAT1* in LUAD, we analyzed the kinase, miRNA, and transcription factor target networks of *GNPNAT1* co-expressed genes. The top five most significant kinase-target networks were cyclin-dependent kinase 1 (CDK1), polo-like kinase 1 (PLK1), Aurora kinase B (AURKB), cyclin-dependent kinase 2 (CDK2), and Aurora kinase A (AURKA) (Table 1 and Supplementary Table 4). Except for *CDK2*, the expression of these genes in LUAD tissue was higher compared with that in normal tissue. Furthermore, except for *CDK2*, for all the kinase genes, higher expression was associated with poor OS of LUAD (Supplementary Figure 1). *CDK1* is a diagnostic biomarker and a prognostic biomarker in LUAD [10, 11].

**Table 1:** The kinase, miRNA and transcription factor targets of GNPNAT1 in LUAD.

**Enriched category**	**Geneset**	**LeadingEdge Num**	**FDR**
Kinase Target	Kinase_CDK1	84	0
	Kinase_PLK1	30	0
	Kinase_AURKB	34	0
	Kinase_CDK2	90	0
	Kinase_AURKA	14	0.00018945
miRNA Target	GGGGCCC,MIR-296	27	0.085139
	CCTGTGA,MIR-513	47	0.10451
	CCCAGAG,MIR-326	49	0.12147
	GAGCCTG,MIR-484	40	0.14007
	AGCGCTT,MIR-518F, MIR-518E,MIR-518A	7	0.1568
Transcription Target	V$E2F_Q6	81	0
	V$E2F1_Q6	85	0
	V$E2F_Q4	81	0
	V$E2F1DP1_01	82	0
	V$E2F1DP2_01	82	0

We obtained miRNA-target networks of *GNPNAT1* co-expressed genes by GSEA, but there was no statistical significance. (Supplementary Table 5). The transcription factor-target networks related primarily to the *E2F* transcription factor family, including E2F-Q6, E2F1-Q6, E2F-Q4, E2F1DP1_01, and E2F1DP2_01 (Supplementary Table 6). High expression levels of E2F family genes are associated with an unfavorable prognosis in LUAD [12, 13].

### Genomic alterations of GNPNAT1 in LUAD

**Figure 5:**
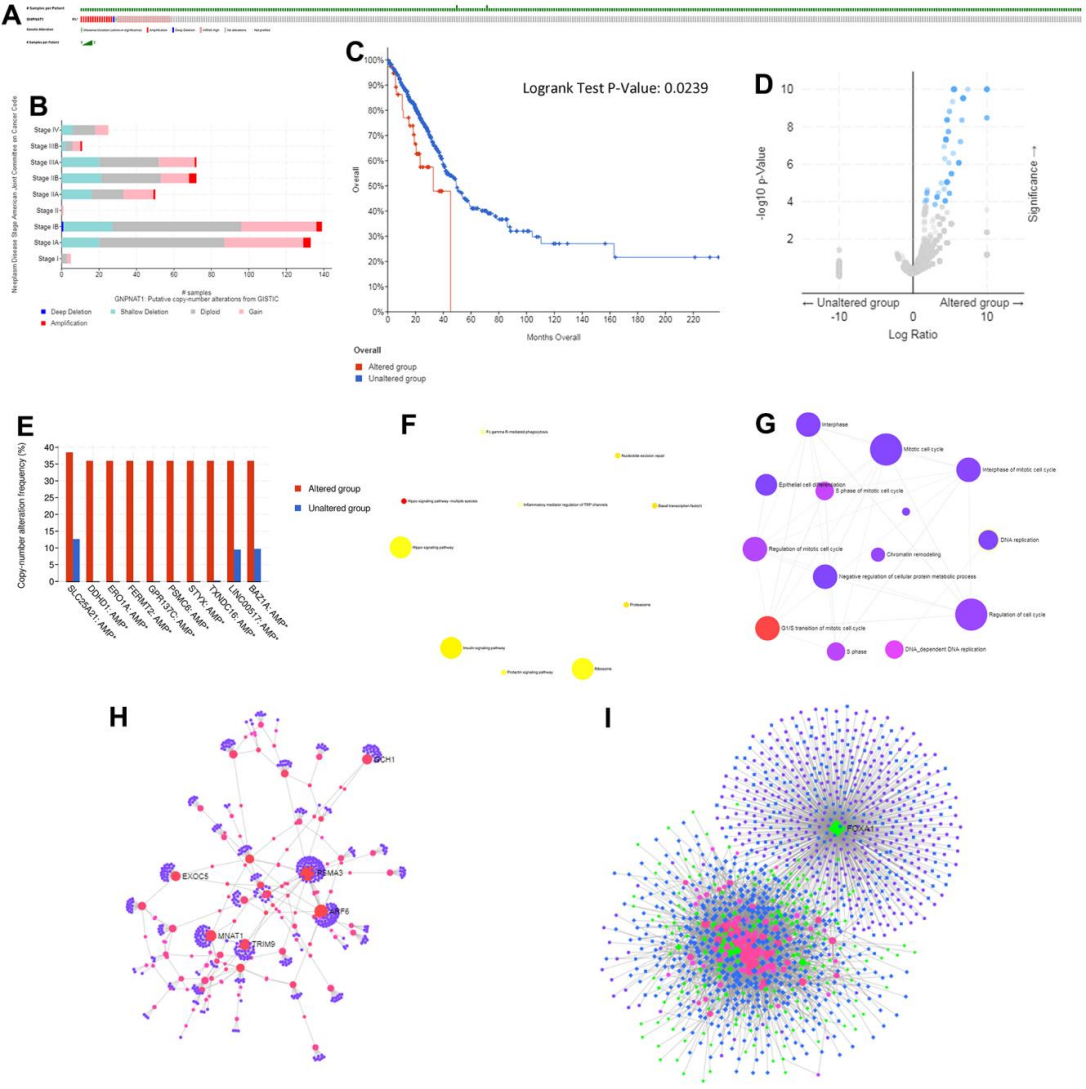
**Genomic alterations of GNPNAT1 in LUAD (cBioPortal).** *GNPNAT1* alterations in the LUAD cohort. The different types of genetic alterations highlighted in different colors. (**A**) There were 39 out of 522 (7.47%) LUAD patients with GNPNAT1 alterations. (**B**) *GNPNAT1* CNV frequency distribution in different stage subgroups. (**C**) *GNPNAT1* CNV affected OS in LUAD. (**D**) The volcano plot shows genes co-occurring with *GNPNAT1* amplification. (**E**) The bar plot shows the top 10 *GNPNAT1* co-occurrent alteration genes. (**F**) KEGG pathway analysis of significant *GNPNAT1* co-occurrent genes. (**G**) GO_BP terms of significantly *GNPNAT1* co-occurrent genes. (**H**) The lung-specific PPI network of significant *GNPNAT1* co-occurrent genes. (**I**) Transcription factor-miRNA (TF-miRNA) coregulatory network of significant *GNPNAT1* co-occurrent genes.

We used the cBioPortal to determine the types and frequency of *GNPNAT1* alterations in LUAD based on sequencing data from LUAD patients in the TCGA database. There were 39 out of 522 (7.47%) LUAD patients with *GNPNAT1* alterations (Figure 5A). The alterations were mRNA upregulation in 23 cases (4.41%), amplification in 11 cases (2.11%), mutation in 1 case (0.19%), deep deletion in 1 case (0.19%), and multiple alterations in 3 cases (0.57%). Although *GNPNAT1* alterations in LUAD were not frequent, mRNA upregulation and amplification were the most common types of *GNPNAT1* copy number variation (CNV) in LUAD. We estimated the frequency distribution of *GNPNAT1* CNV patients in different stage groups; as shown in Figure 5B, stage I and stage II patients had a high occurrence of *GNPNAT1* CNV alteration in LUAD. We divided the patients with LUAD into a *GNPNAT1* CNV alteration group and a no GNPNAT1 CNV alteration group; *GNPNAT1* CNV alteration group patients had poorer OS than patients in the no *GNPNAT1* CNV alteration group (p < 0.05) (Figure 5C). The median survival time was 32.82 months and 49.31 months, respectively.

### Gene co-occurrence of GNPNAT1 alterations in LUAD

Gene co-occurrence reflected common genetic risk factors constituting functional relationships. We subsequently identified the co-occurrence genes with *GNPNAT1* amplification in LUAD. There were 120 significant co-occurrences with *GNPNAT1* amplification genes, as shown in Figure 5D (Supplementary Table 7). The top 10 alterations were solute carrier family 25 member 21 (SLC25A21), DDHD domain containing 1(DDHD1), endoplasmic reticulum oxidoreductase 1 alpha (ERO1A), fermitin family member 2 (FERMT2), G-protein-coupled receptor 137C (GPR137C), proteasome 26S subunit, ATPase 6 (PSMC6), serine/threonine/tyrosine interacting protein (STYX), thioredoxin domain containing 16 (TXNDC16), long intergenic non-protein coding RNA 517 (LINC00517), bromodomain adjacent to zinc finger domain 1A (BAZ1A), shown in Figure 5E. Enriched KEGG pathway analysis indicated that these co-occurrent genes were mainly enriched in Hippo signaling pathway-multiple species (Figure 5F). This signal pathway is primarily associated with the proliferation and apoptosis of tumor cells. GO term analysis of these genes showed enrichment in the G1/S transition of the mitotic cell cycle (Figure 5G and Supplementary Table 8), which also indicated that these genes participate in the growth of tumors.

Moreover, we constructed a *GNPNAT1* co-occurrence gene protein-protein interaction (PPI) network using lung-specific data collected from the DifferentialNet database (Figure 5H). Proteasome 20S subunit alpha (PSMA) 3, *PSMA4*, *PSMA5*, tripartite motif containing 9 (TRIM9), and GTP cyclohydrolase 1(GCH1) were the top 5 hub genes. *PSMA* is a proteasome subunit alpha type associated with the occurrence and development of multiple cancers [14, 15].

Finally, we constructed the *GNPNAT1* co-occurrence genes TF-miRNA coregulatory interactions using the RegNetwork repository (Figure 5I). Forkhead box A1(FOXA1), Kelch-like family member 28 (KLHL28), cofilin 2 (CFL2), SIX homeobox 4 (SIX4), and glia maturation factor beta (GMFB) were the top five TFs. Many studies have shown that *FOXA1* participates in the development of lung cancer, prostate cancer, and several other types of cancers [16–19].

### Association between GNPNAT1 expression and immune infiltration level in LUAD

**Figure 6:**
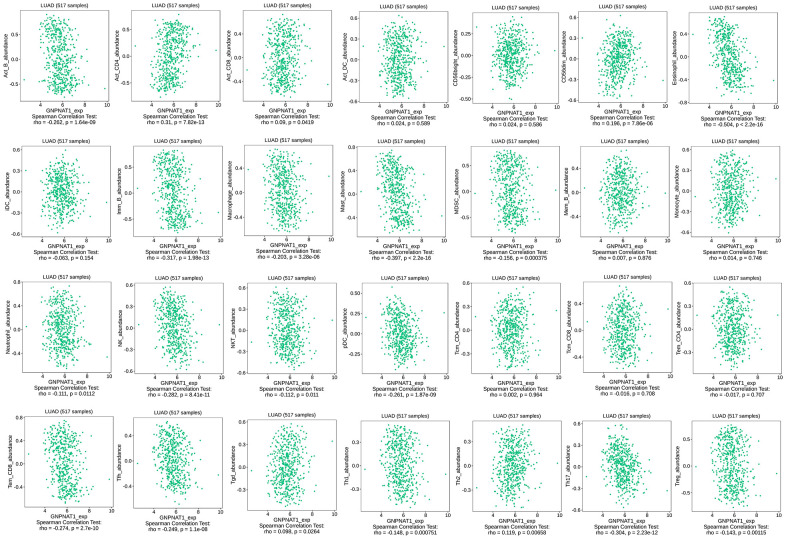
**GNPNAT1 expression had a significant correlation with immune cells infiltration level in LUAD.**

We used the TISIDB database to assess whether GNPNAT1 expression was significantly correlated with immune cell infiltration level in LUAD, as shown in Table 2 (Figure 6). There were 20/28 significantly correlated immune cells, including B cells, CD4 T cells, CD8 T cells, CD56dim cells, eosinophils, IMM B cells, macrophages, mast cells, MDSC cells, neutrophils, NK cells, NKT cells, pDC cells, Tem CD8 cells, Tfh cells, Tgd cells, Th1 cells, Th2 cells, Th17 cells, and Treg cells. We also observed associations between *GNPNAT1* expression and immune cell infiltration levels across different cancer types (Supplementary Figure 2A).

**Table 2:** Correlation analysis between GNPNAT1 expression and immune cells infiltration level in LUAD.

**Immune cell**	**Correlation coefficient**	**P value**
Activated B cell	-0.262	1.64e-09***
Activated CD4 T cell	0.31	7.82e-13***
Activated CD8 T cell	0.09	0.0419*
Activated dendritic cell	0.024	0.589
CD56bright natural killer cell	0.024	0.586
CD56dim natural killer cell	0.196	7.86e-06***
Eosinophil cell	-0.504	<2.2e-16***
Immature dendritic cell	-0.063	0.154
Immature B cell	-0.317	1.98e-13***
Macrophage cell	-0.203	3.28e-06***
Mast cell	-0.397	<2.2e-16***
Myeloid derived suppressor cell	-0.156	0.000375***
Memory B cell	0.007	0.876
Monocyte	0.014	0.746
Neutrophil	-0.111	0.0112*
Natural killer cell	-0.282	8.41e-11***
Natural killer T cell	-0.112	0.011*
Plasmacytoid dendritic cell	-0.261	1.87e-09***
Central memory CD4 T cell	0.002	0.964
Central memory CD8 T cell	-0.016	0.708
Effector memory CD4 T cell	-0.017	0.707
Effector memory CD8 T cell	-0.274	2.7e-10***
T follicular helper cell	-0.249	1.1e-08***
Gamma delta T cell	0.098	0.0264*
Type 1 T helper cell	-0.148	0.000751***
Type 2 T helper cell	0.119	0.00658**
Type 17 T helper cell	-0.304	2.23e-12***
Regulatory T cell	-0.143	0.00115**

*, *p* < 0.05; **, *p* < 0.01; ***, *p* < 0.001.

**Figure 7:**
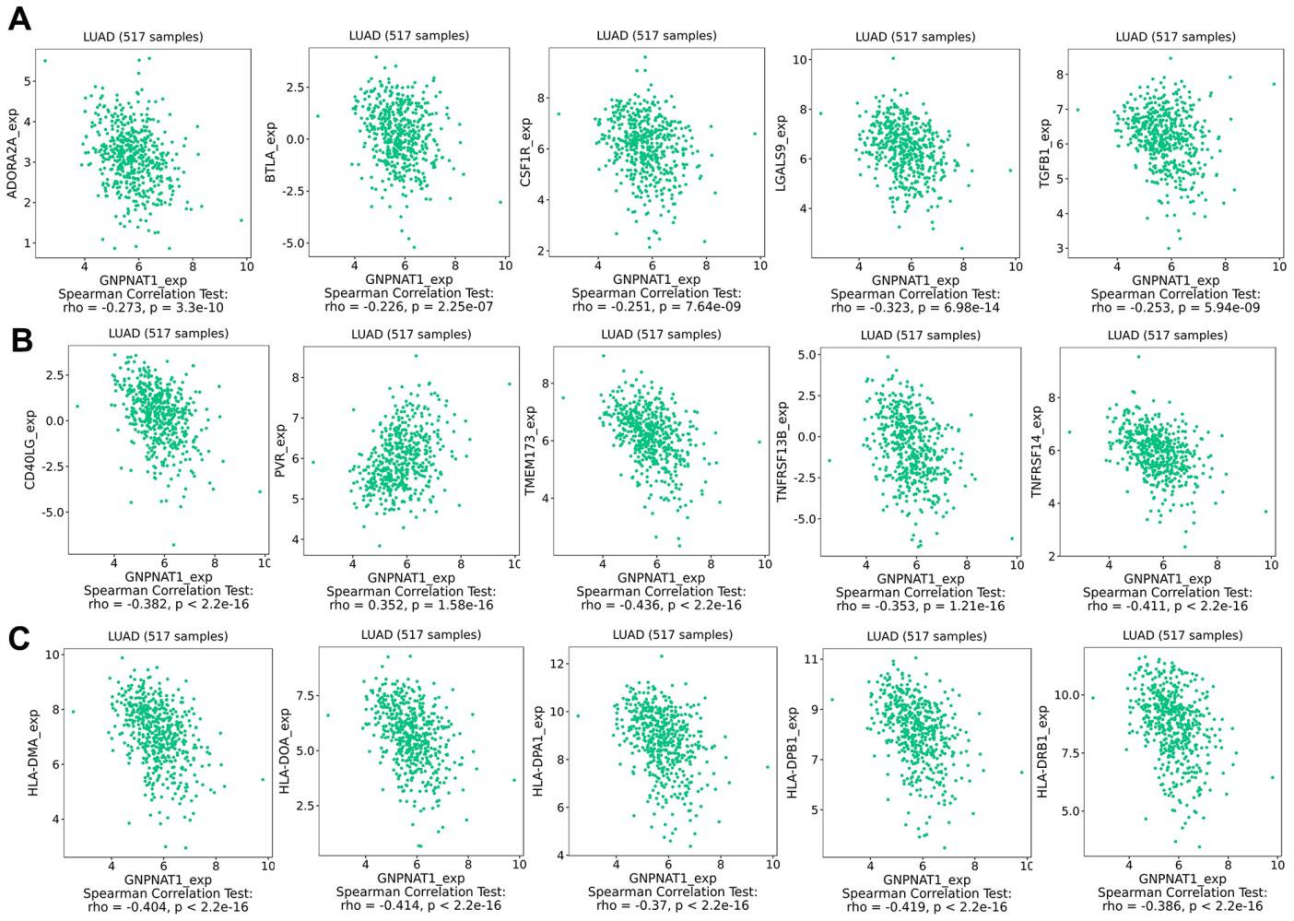
**GNPNAT1 expression associated with immunomodulators in LUAD.** (**A**) Top five immunoinhibitors correlated with *GNPNAT1* expression in LUAD. (**B**) Top five immunostimulators correlated with *GNPNAT1* expression in LUAD. (**C**) Top five MHC molecules correlated with *GNPNAT1* expression in LUAD.

Immunomodulators included immunoinhibitors, immunostimulators, and MHC molecules, which regulate immune system functions. We found that *GNPNAT1* expression was significantly negatively correlated with immunomodulators (Table 3). The five most highly correlated immunoinhibitors (Figure 7A) were galectin 9 (LGALS9), adenosine A2a receptor (ADORA2A), transforming growth factor beta 1 (TGFB1), colony-stimulating factor 1 receptor (CSF1R), B and T lymphocyte associated (BTLA). The five most highly correlated immunostimulators (Figure 7B) were transmembrane protein 173 (TMEM173), TNF receptor superfamily member 14 (TNFRSF14), CD40 ligand (CD40LG), TNF receptor superfamily member 13B (TNFRSF13B), and PVR cell adhesion molecule (PVR). The five most highly correlated MHC molecules (Figure 7C) were major histocompatibility complex, class II, DP beta 1 (HLA-DPB1), major histocompatibility complex, class II, DO alpha (HLA-DOA), major histocompatibility complex, class II, DM alpha (HLA-DMA), major histocompatibility complex, class II, DR beta 1 (HLA-DRB1), and major histocompatibility complex, class II, DP alpha 1 (HLA-DPA1). We also observed associations between *GNPNAT1* expression and immunomodulators across different cancer types (Supplementary Figure 2B–2D).

**Table 3:** Correlation analysis between GNPNAT1 expression and immunomodulators expression in LUAD.

**Immunomodulators**	**Geneset**	**Correlation coefficient**	**P value**
Immunoinhibitors	ADORA2A	-0.273	3.3e-10***
	BTLA	-0.226	2.25e-07***
	CSF1R	-0.251	7.64e-09***
	LGALS9	-0.323	6.98e-14***
	TGFB1	-0.253	5.94e-09***
Immunostimulators	CD40LG	-0.382	<2.2e-16***
	PVR	0.352	1.58e-16***
	TMEM173	-0.436	<2.2e-16***
	TNFRSF13B	-0.353	1.21e-16***
	TNFRSF14	-0.411	<2.2e-16***
MHC molecules	HLA-DMA	-0.404	<2.2e-16***
	HLA-DOA	-0.414	<2.2e-16***
	HLA-DPA1	-0.37	<2.2e-16***
	HLA-DPB1	-0.419	<2.2e-16***
	HLA-DRB1	-0.386	<2.2e-16***

***, *p* < 0.001.

**Figure 8:**
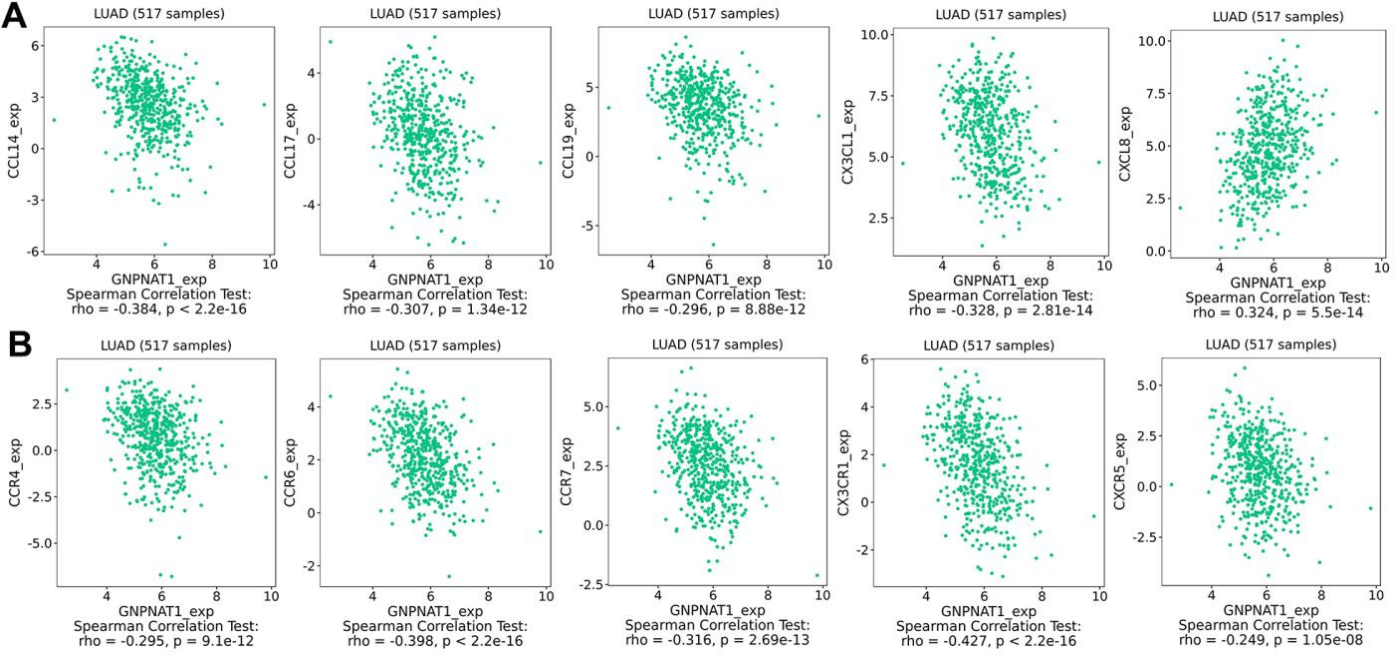
**Correlation between GNPNAT1 expression and chemokines in LUAD.** (**A**) Top five chemokines correlated with *GNPNAT1* expression in LUAD. (**B**) Top five chemokine receptors correlated with *GNPNAT1* expression in LUAD.

Chemokines expression levels are key factors in controlling immune cell infiltration. We identified the correlation between *GNPNAT1* expression and chemokines (Table 4). The five most highly correlated chemokines (Figure 8A) were C-C motif chemokine ligand (CCL)-14, C-X3-C motif chemokine ligand 1 (CX3CL1), CXC motif chemokine ligand 8 (CXCL8), *CCL17*, *CCL19*. The top five chemokine receptors (Figure 8B) were C-X3-C motif chemokine receptor 1 (CX3CR1), CC motif chemokine receptor (CCR)-6, *CCR7*, *CCR4*, CXC motif chemokine receptor 5 (CXCR5). Moreover, we described the correlation of *GNPNAT1* expression with chemokines or receptors across different cancer types (Supplementary Figure 3).

**Table 4:** Correlation analysis between GNPNAT1 expression and chemokines expression in LUAD.

**Chemokines**	**Geneset**	**Correlation coefficient**	**P value**
Chemokines	CCL14	-0.384	<2.2e-16***
	CCL17	-0.307	1.34e-12***
	CCL19	-0.296	8.88e-12***
	CX3CL1	-0.328	2.81e-14***
	CXCL8	0.324	5.5e-14***
Chemokine receptors	CCR4	-0.295	9.1e-12***
	CCR6	-0.398	<2.2e-16***
	CCR7	-0.316	2.69e-13***
	CX3CR1	-0.427	<2.2e-16***
	CXCR5	-0.249	1.05e-08***

***, *p* < 0.001.

**Figure 9:**
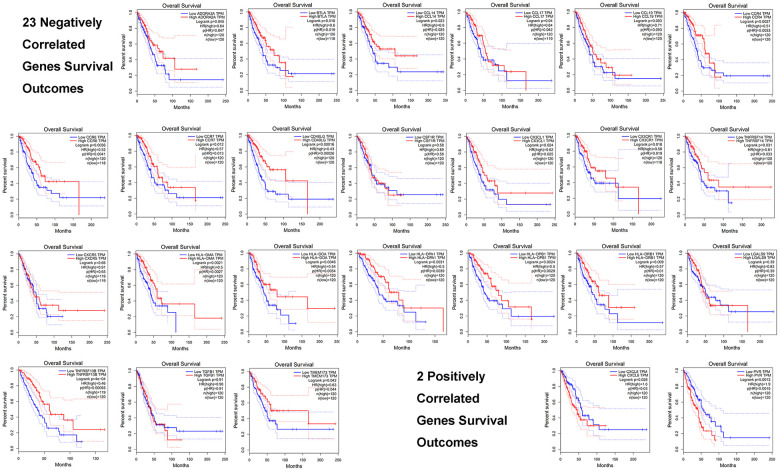
**Prognosis of the top 25 immune genes correlated with GNPNAT1 in LUAD, including 23 negatively correlated genes and 2 positively correlated genes.**

We evaluated these 25 immune genes correlated with *GNPNAT1*, including 23 negatively correlated genes and 2 positively correlated genes, and we found that higher expression of 18/23 negatively correlated genes was associated with favorable prognosis in patients with LUAD, while higher expression of 2/2 positively correlated genes was associated with unfavorable prognosis. (Table 5 and Figure 9).

**Table 5:** The prognosis of the top 25 immune genes correlative with GNPNAT1 in LUAD.

	**Geneset**	**Hazards ratio(high)**	**P value**
Negatively correlative gene	ADORA2A	0.64	0.045*
	BTLA	0.6	0.018*
	CCL14	0.6	0.023*
	CCL17	0.64	0.04*
	CCL19	0.71	0.093
	CCR4	0.51	0.0027**
	CCR6	0.52	0.0036**
	CCR7	0.57	0.012*
	CD40LG	0.43	0.00016***
	CSF1R	0.89	0.58
	CX3CL1	0.62	0.024*
	CX3CR1	0.58	0.018*
	CXCR5	0.91	0.66
	HLA-DMA	0.5	0.0021**
	HLA-DOA	0.54	0.0046**
	HLA-DPA1	0.5	0.0031**
	HLA-DPB1	0.5	0.0024**
	HLA-DRB1	0.57	0.009**
	LGALS9	0.83	0.39
	TGFB1	0.98	0.91
	TMEM173	0.63	0.042*
	TNFRSF13B	0.46	4e-04***
	TNFRSF14	0.61	0.031*
Positively correlative gene	CXCL8	1.6	0.028*
	PVR	1.9	0.0013**

*, *p* < 0.05; **, *p* < 0.01; ***, *p* < 0.001.

## DISCUSSION

Metabolic pathways, including glucose metabolism, amino acid metabolism, and fatty metabolism, participate in regulating tumor cell proliferation and progression, as reported by several researchers [20]. *GNPNAT1*, also called *GNA1*, is a protein with a crucial role in acetylglucosamine biosynthesis. Downregulation of *GNPNAT1* expression has been found to be the key reason for the inhibition of lung cancer A549 cell proliferation and adhesion [9]. *GNPNAT1* has also been reported to be a part of a metabolic gene signature in LUAD in six-gene and five-gene signatures [21, 22]. To obtain more detailed insights into *GNPNAT1* potential functions and regulatory networks in LUAD and guide future LUAD research, we conducted a bioinformatics analysis of public data.

From the TCGA and GEO databases containing five LUAD study cohorts, we analyzed transcriptomes of more than 800 clinical samples and found that *GNPNAT1* mRNA expression levels were higher in LUAD than in normal lung tissues (Figure 1). The analysis also confirmed that higher *GNPNAT1* expression in LUAD was related to more unfavorable prognosis in multiple LUAD study cohorts. We analyzed our results, which indicated that overexpression of *GNPNAT1* occurred in many patients with LUAD, and that further clinical and experimental validation was needed to investigate on its potential role as a diagnostic and prognostic marker.

The *GNPNAT1* co-expression networks in LUAD are shown in Figure 4. The positively correlated genes with higher expression in LUAD were usually associated with worse survival outcomes, while the negatively correlated genes were associated with opposite outcomes. The overexpression of *CDKN3* increased mitotic activity, resulting in more unfavorable prognosis in patients with LUAD [23]. Floriana Forzati and colleagues reported that *CBX7* is a tumor suppressor, and its inactivation promotes LUAD progression [24]. We used GSEA to annotate the co-expressed genes; GO terms were mainly enriched in cell chromosome segregation and RNA metabolisms, etc., and KEGG pathways in ribosome and proteasome, etc. These enrichment functions commonly participate in cell proliferation and differentiation.

To identify the regulators that are potentially responsible for *GNPNAT1* dysregulation, we revealed a network of kinases related to *GNPNAT1* in LUAD, including *CDK1*, *PLK1*, *AURKB*, *CDK2*, and *AURKA*. These kinases target the regulation of genomic stability, mitosis progression, and cell cycle transition, which show differential expression and different prognoses in LUAD [11, 25–28]. *CDK1* is mainly defined as a pivotal cell cycle regulator that not only participates in mitosis, but also in meiosis and protein synthesis [29]. Researchers have demonstrated that *CDK1* inactivation influences multiple tumors cell cycle progression, thereby *CDK1* might be a tumor therapeutic target. Many *CDK1* inhibitors have been discovered and used in various tumors [30]. *PLK1*, *AURKB*, and *AURKA* are crucial factors not only in mitosis, but also in non-mitosis function and DNA damage response. For cancer therapies, these kinase inhibitors have been developed in diverse tumors [31–34]. In LUAD, *GNPNAT1* deficiency results in cell cycle arrest, DNA damage, and repair response dysfunction, which might be due to the synergistic effects of these kinases. *E2F* family members are the key transcription factors of *GNPNAT1* in LUAD. These *E2F* family genes mainly participate in cell cycle regulation, and uncontrolled cell cycle progression results in cancerous events [35]. Previous studies have shown that *E2F* transcription factors are significantly enriched in multiple tumor tissues and have verified that high *E2F* expression in hepatocellular carcinoma and LUAD is correlated to a worse prognosis [12, 13]. *E2F1* is a key factor that prompts cell cycle transition in LUAD, and higher *E2F1* expression in LUAD indicates unfavorable survival outcome [36]. Retinoblastoma (RB) protein and tumor protein P53 (TP53) participate in increasing *E2F* family gene expression in some particular tumors, and *GNPNAT1* might also be a targeted gene in LUAD. Though, further research is required to prove this [37, 38].

Genomic alteration is frequently detected in patients with LUAD, and this alteration might predict unfavorable prognosis. CNVs might have significant genomic influence, disrupt genes, and change genetic content, leading to phenotypic differences [39, 40]. Our analysis results showed that the CNV of *GNPNAT1* in LUAD had increased, and mRNA upregulation and amplification were the main types of *GNPNAT1* alterations associated with unfavorable prognosis. By analyzing *GNPNAT1* co-occurrence gene function, we found that *GNPNAT1* might participate in multiple tumor cell cycle progression, further influencing tumor cell proliferation and apoptosis.

Malignant tumors are composed of not only tumor cells, but also non-tumor cells, such as immune cells, stromal cells, and normal epithelial cells, which are also called tumor micro-environment (TME) [41]. These cells in the TME can promote or inhibit tumor cell growth [42]. In TME, with the occurrence and development of tumors, abnormal tumor metabolism might lead to immunosuppression, and tumor cells might evade the immune response [43]. In this study, the tumor metabolism gene *GNPNAT1* expression was closely related to immune cell infiltration in LUAD and was correlated with immunomodulators and chemokines. These immune factors were significantly associated with the prognosis of LUAD. In recent years, immunotherapy for LUAD has progressed significantly, and more research is needed to assess whether *GNPNAT1* may be an immunotherapy critical factor in the future.

In this study, we found that *GNPNAT1* is an important gene in the development and progression of LUAD by multi-omics analyses. *GNPNAT1* was expressed at higher levels in LUAD tumor tissues than in normal tissues, which makes *GNPNAT1* a potential biomarker in LUAD. *GNPNAT1* overexpression in LUAD predicts a worse prognosis, which might be caused by the disruption of RNA metabolism and transport, and by that of mitotic cell cycle progression. We also found that *GNPNAT1* has a potential novel immunomodulatory role in LUAD tumor immunity, and it might be a new target for lung cancer immunotherapy in the future. Nonetheless, all our findings need to be verified by further LUAD genomics research and subsequent functional studies.

## MATERIALS AND METHODS

### LCE database analysis

LCE is a lung cancer-specific database including expression data and clinical data from over 6700 patients in 56 studies [44]. We could easily obtain an overview analysis of tumor versus non-malignant tissue (normal tissue) differential gene expression and expression–survival association by meta-analyses. In addition, we obtained an individual data set-based survival analysis, comparative analysis, and correlation analysis.

### GEPIA database analysis

The GEPIA database (http://gepia.cancer-pku.cn/) is an interactive website that contains 9736 tumor samples and 8587 normal samples from TCGA and GTEx datasets [45]. We used GEPIA to generate OS curves, based on gene expression with the log-rank test and the Mantel-Cox test in LUAD. We obtained tumor versus non-malignant tissue (normal) gene differential expression map. The threshold values were *p*-value of 0.05 and fold change of 1.0.

### Oncomine database analysis

The Oncomine database (https://www.oncomine.org/) is a cancer microarray database and web-based data-mining platform. The gene GNPNAT1 expression level in LUAD was examined in the Oncomine 4.5 database. We used a *p*-value of 0.05, fold change of 1.2, and gene ranking of all as the threshold values.

### UALCAN database analysis

The UALCAN (http://ualcan.path.uab.edu) database is a comprehensive, user-friendly, and interactive web resource for analyzing cancer omics data [46]. We used the UALCAN database to obtain the gene *GNPNAT1* expression analysis across LUAD and normal samples in various tumor sub-groups based on TCGA data.

### HPA database analysis

The HPA (http://www.proteinatlas.org) database maps human proteins in cells, tissues, and organs using the integration of various omics technologies [47]. *GNPNAT1* protein expression in LUAD tissues and normal lung tissues was mapped by immunohistochemistry.

### LinkedOmics database analysis

The LinkedOmics database (http://www.linkedomics.org/login.php) is a publicly available portal that includes multi-omics data from all 32 TCGA cancer types [48]. We used LinkedOmics to gain the *GNPNAT1* co-expression assessed by Pearson’s correlation coefficient statistical analysis, presented in volcano plots and heat maps. We used the GSEA function modules in the LinkedOmics database to obtain Gene Ontology biological process (GO_BP), KEGG pathways, kinase-target enrichment, miRNA-target enrichment, and transcription factor-target enrichment analysis. FDR < 0.05 and 1000 simulations were the standard in this enrichment analysis.

### c-BioPortal database analysis

The cBioPortal (http://cbioportal.org) includes multidimensional cancer genomics [49]. We used the c-BioPortal tool to analyze *GNPNAT1* mutation, gene co-occurrence, and CNV in LUAD (TCGA, Firehose Legacy). We also obtained the *GNPNAT1* alterations overview per sample via this portal.

### NetworkAnalyst database analysis

NetworkAnalyst 3.0 (https://www.networkanalyst.ca/) [50] is a tool that allows to create cell-type or tissue-specific PPI networks, gene regulatory networks, enrichment networks, and gene co-expression networks. All the *GNPNAT1* co-occurrence gene networks were built using this tool.

### TISIDB database analysis

TISIDB is a web portal for tumor and immune system interaction, which integrates data regarding 30 cancer types from TCGA (http://cis.hku.hk/TISIDB/) [51]. We used TISIDB tools to analyze the correlation of *GNPNAT1* with 28 tumor-infiltrating lymphocytes (TILs), 45 immunostimulators, 24 immunoinhibitors, 21 MHC molecules, 41 chemokines, and 18 receptors.

### Statistical analysis

We used Student’s t-tests to identify the different *GNPNAT1* expression levels. Kaplan-Meier curves and the log-rank test were used to compare the OS of various gene expression subgroups. The correlation between *GNPNAT1* expression, immune cell infiltration, and immune genes was evaluated by Spearman’s method. In these analyses, *p* < 0.05 was considered statistically significant.

## Supplementary Material

Supplementary Figures

Supplementary Table 1

Supplementary Tables 2, 3, 4, 5 and 6

Supplementary Table 7

Supplementary Table 8
