# Supplementary material for: Potential role of glucosamine-phosphate N-acetyltransferase 1 in the development of lung adenocarcinoma
Source: Aging (Albany NY). 2021 Mar 3;13(5):7430–53. doi: 10.18632/aging.202604 (PMC7993716; doi:10.18632/aging.202604)
Supplement: Supplementary Figures [file aging-13-202604-s001.pdf]

SUPPLEMENTARY FIGURES

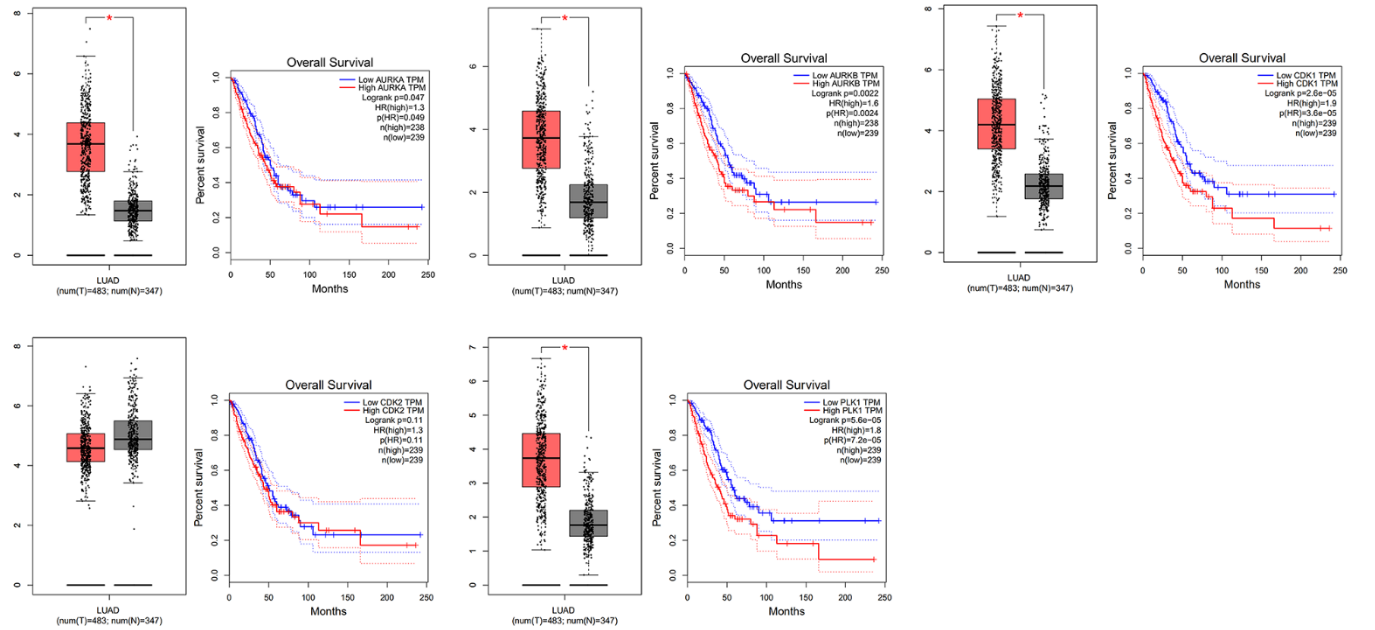

Supplementary Figure 1. The expression and survival outcome of *GNPNAT1*-associated top five most significant kinase in LUAD.

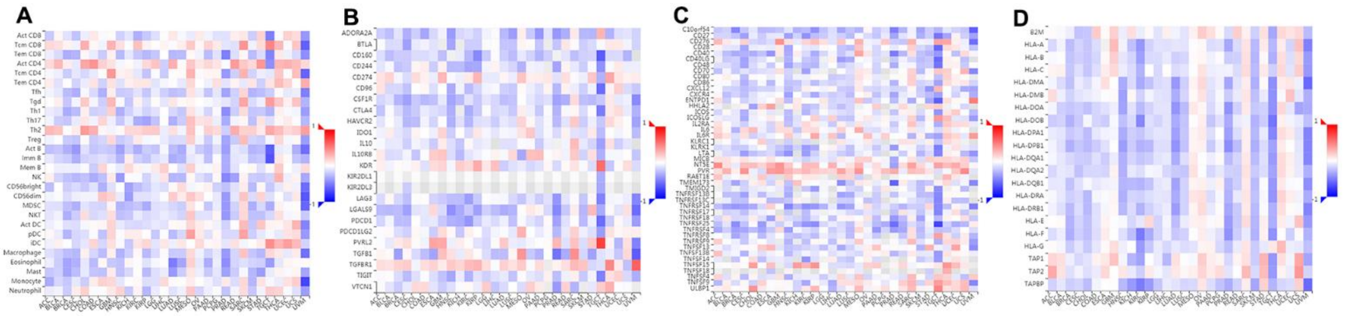

Supplementary Figure 2. The relationship between *GNPNAT1* expression and immune cells, immunomodulators across different cancers. (**A**) *GNPNAT1* expression correlated with immune cells infiltration level across different cancer types. (**B**) *GNPNAT1* expression correlated with immunoinhibitors across different cancer types. (**C**) *GNPNAT1* expression correlated with immunostimulators across different cancer types. (**D**) *GNPNAT1* expression correlated with MHC molecules across different cancer types.

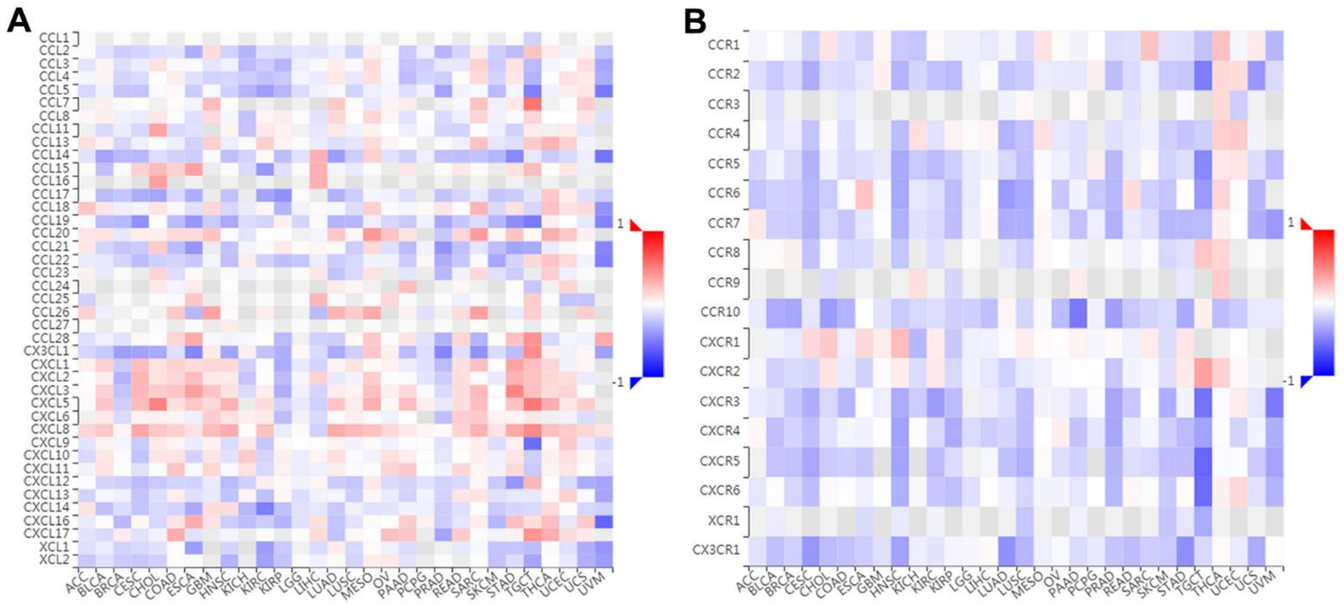

Supplementary Figure 3. The relationship between *GNPNAT1* expression and chemokines across different cancers. (**A**) *GNPNAT1* expression correlated with chemokines across different cancer types. (**B**) *GNPNAT1* expression correlated with chemokine receptors across different cancer types.
