# Supplementary material for: Potential role of glucosamine-phosphate N-acetyltransferase 1 in the development of lung adenocarcinoma
Source: Aging (Albany NY). 2021 Mar 3;13(5):7430–53. doi: 10.18632/aging.202604 (PMC7993716; doi:10.18632/aging.202604)
Supplement: Supplementary Table 1 [file aging-13-202604-s002.docx]

**Supplementary Table 1. GNPNAT1 co-expressed genes.**

| **Query** | **Statistic** | **P-value** | **FDR (BH)** |
| --- | --- | --- | --- |
| A1CF | 0.144260373 | 0.001027005 | 0.002181254 |
| A2M | -0.37658219 | 8.50E-19 | 1.87E-17 |
| A4GNT | -0.213617454 | 9.95E-07 | 3.50E-06 |
| AAAS | 0.105652204 | 0.01646146 | 0.027917162 |
| AACSL | -0.108419775 | 0.013827332 | 0.023836198 |
| AACS | 0.254661778 | 4.57E-09 | 2.29E-08 |
| AADACL2 | -0.119822936 | 0.006480525 | 0.011914344 |
| AADAC | -0.180733406 | 3.70E-05 | 0.000100881 |
| AADAT | -0.219649637 | 4.80E-07 | 1.78E-06 |
| AAGAB | 0.38305059 | 1.92E-19 | 4.65E-18 |
| AAK1 | -0.208271628 | 1.87E-06 | 6.28E-06 |
| AANAT | -0.146240115 | 0.00087295 | 0.001875178 |
| AARS2 | -0.108945876 | 0.013370889 | 0.02310657 |
| AARS | 0.174286369 | 7.01E-05 | 0.000182603 |
| AASDHPPT | 0.248920572 | 1.03E-08 | 4.93E-08 |
| AASS | -0.297932077 | 5.12E-12 | 3.99E-11 |
| AATF | 0.142709288 | 0.001164828 | 0.002448736 |
| AATK | -0.244684125 | 1.86E-08 | 8.53E-08 |
| ABAT | -0.308817125 | 7.66E-13 | 6.83E-12 |
| ABCA10 | -0.448152503 | 8.27E-27 | 5.90E-25 |
| ABCA13 | -0.224060796 | 2.78E-07 | 1.07E-06 |
| ABCA17P | -0.130976617 | 0.002901851 | 0.005681476 |
| ABCA1 | -0.104195758 | 0.018016752 | 0.030310483 |
| ABCA2 | -0.302770488 | 2.22E-12 | 1.84E-11 |
| ABCA3 | -0.353434414 | 1.34E-16 | 2.09E-15 |
| ABCA4 | -0.218258385 | 5.69E-07 | 2.08E-06 |
| ABCA5 | -0.378424609 | 5.58E-19 | 1.26E-17 |
| ABCA6 | -0.329636443 | 1.61E-14 | 1.85E-13 |
| ABCA7 | -0.311718544 | 4.56E-13 | 4.19E-12 |
| ABCA8 | -0.400258463 | 3.09E-21 | 9.76E-20 |
| ABCA9 | -0.36722352 | 6.92E-18 | 1.33E-16 |
| ABCB10 | 0.167722497 | 0.000131249 | 0.000327721 |
| ABCB1 | -0.173087057 | 7.87E-05 | 0.00020334 |
| ABCB6 | 0.181744665 | 3.34E-05 | 9.18E-05 |
| ABCB7 | 0.150591667 | 0.000606397 | 0.001340336 |
| ABCC10 | -0.16202216 | 0.000222185 | 0.0005332 |
| ABCC12 | -0.379898753 | 3.98E-19 | 9.20E-18 |
| ABCC13 | -0.189231403 | 1.54E-05 | 4.49E-05 |
| ABCC2 | 0.304713091 | 1.58E-12 | 1.34E-11 |
| ABCC3 | -0.259171719 | 2.38E-09 | 1.24E-08 |
| ABCC4 | -0.153508839 | 0.000472382 | 0.001063046 |
| ABCC5 | -0.172827928 | 8.07E-05 | 0.000208083 |
| ABCC6P1 | -0.260251233 | 2.03E-09 | 1.07E-08 |
| ABCC6P2 | -0.298004333 | 5.06E-12 | 3.95E-11 |
| ABCC6 | -0.39529518 | 1.04E-20 | 3.02E-19 |
| ABCC8 | -0.119272003 | 0.006732082 | 0.012347297 |
| ABCD2 | -0.107896067 | 0.014295267 | 0.024575023 |
| ABCD3 | -0.103508185 | 0.018794561 | 0.03148916 |
| ABCD4 | -0.220360436 | 4.40E-07 | 1.64E-06 |
| ABCE1 | 0.48134611 | 3.17E-31 | 4.40E-29 |
| ABCF1 | 0.097091126 | 0.027580529 | 0.044638025 |
| ABCF2 | 0.361152836 | 2.60E-17 | 4.55E-16 |
| ABCG1 | -0.124908731 | 0.004527387 | 0.008562964 |
| ABCG8 | 0.145704787 | 0.000912351 | 0.001953046 |
| ABHD10 | 0.163861064 | 0.000187837 | 0.000456471 |
| ABHD14A | -0.202073589 | 3.79E-06 | 1.21E-05 |
| ABHD14B | -0.198336895 | 5.76E-06 | 1.79E-05 |
| ABHD15 | -0.121820816 | 0.005637495 | 0.010490852 |
| ABHD1 | -0.09547749 | 0.030279462 | 0.048569608 |
| ABHD3 | 0.148009754 | 0.000753623 | 0.001639756 |
| ABHD4 | 0.107039312 | 0.015090846 | 0.02582177 |
| ABHD6 | -0.107105639 | 0.015027895 | 0.025729114 |
| ABHD8 | -0.106953084 | 0.01517303 | 0.025934542 |
| ABI1 | 0.184279002 | 2.58E-05 | 7.22E-05 |
| ABI2 | -0.161440492 | 0.000234219 | 0.000559394 |
| ABI3BP | -0.394686023 | 1.21E-20 | 3.45E-19 |
| ABI3 | -0.230205147 | 1.27E-07 | 5.17E-07 |
| ABL2 | 0.096241409 | 0.028974779 | 0.046664078 |
| ABLIM2 | -0.134389596 | 0.002241246 | 0.00447801 |
| ABO | -0.251539308 | 7.13E-09 | 3.48E-08 |
| ABRA | -0.191644517 | 1.19E-05 | 3.53E-05 |
| ABR | -0.247707242 | 1.22E-08 | 5.76E-08 |
| ABT1 | 0.172827146 | 8.07E-05 | 0.000208083 |
| ABTB1 | -0.452004807 | 2.69E-27 | 2.06E-25 |
| ACAA1 | -0.249882045 | 9.01E-09 | 4.34E-08 |
| ACACB | -0.393878286 | 1.47E-20 | 4.12E-19 |
| ACAD10 | -0.134000067 | 0.002308993 | 0.004602787 |
| ACAD11 | -0.240772434 | 3.16E-08 | 1.40E-07 |
| ACAD8 | -0.354692719 | 1.03E-16 | 1.63E-15 |
| ACADL | -0.324294912 | 4.47E-14 | 4.78E-13 |
| ACADSB | -0.239768777 | 3.62E-08 | 1.59E-07 |
| ACADS | -0.250112418 | 8.72E-09 | 4.21E-08 |
| ACADVL | -0.175027895 | 6.52E-05 | 0.000170703 |
| ACAN | 0.128278959 | 0.003544418 | 0.006827198 |
| ACAP1 | -0.257927452 | 2.85E-09 | 1.47E-08 |
| ACAP3 | -0.213424674 | 1.02E-06 | 3.57E-06 |
| ACAT2 | 0.180910328 | 3.63E-05 | 9.93E-05 |
| ACBD3 | 0.163576608 | 0.000192803 | 0.000467573 |
| ACBD4 | -0.322184116 | 6.65E-14 | 6.92E-13 |
| ACBD5 | 0.219527938 | 4.87E-07 | 1.80E-06 |
| ACBD6 | 0.177731791 | 4.99E-05 | 0.00013334 |
| ACCN3 | -0.203278805 | 3.31E-06 | 1.07E-05 |
| ACCN4 | -0.203745732 | 3.14E-06 | 1.02E-05 |
| ACCSL | -0.11578899 | 0.008535043 | 0.015331934 |
| ACCS | -0.402578041 | 1.74E-21 | 5.75E-20 |
| ACE2 | -0.169715311 | 0.000108744 | 0.000275414 |
| ACER1 | -0.18264772 | 3.05E-05 | 8.43E-05 |
| ACER2 | -0.138596633 | 0.00161688 | 0.003309933 |
| ACE | -0.230370034 | 1.25E-07 | 5.07E-07 |
| ACHE | -0.246148823 | 1.52E-08 | 7.06E-08 |
| ACIN1 | -0.141493655 | 0.001284543 | 0.002680388 |
| ACLY | 0.308576765 | 8.00E-13 | 7.12E-12 |
| ACMSD | 0.128125309 | 0.003584636 | 0.006897353 |
| ACN9 | 0.321247605 | 7.93E-14 | 8.14E-13 |
| ACOT12 | 0.128346149 | 0.00352696 | 0.006798157 |
| ACOT13 | 0.17574442 | 6.08E-05 | 0.000159718 |
| ACOT4 | 0.132613814 | 0.002565539 | 0.005071703 |
| ACOT6 | 0.158604845 | 0.000302129 | 0.00070534 |
| ACOT7 | 0.252171588 | 6.52E-09 | 3.20E-08 |
| ACOT9 | 0.204250921 | 2.96E-06 | 9.62E-06 |
| ACOX2 | -0.143456624 | 0.001096424 | 0.002316386 |
| ACOX3 | -0.170430914 | 0.000101588 | 0.000258241 |
| ACOXL | -0.490071228 | 1.81E-32 | 3.12E-30 |
| ACP1 | 0.298194533 | 4.90E-12 | 3.82E-11 |
| ACP5 | -0.248342125 | 1.12E-08 | 5.30E-08 |
| ACRBP | -0.116253505 | 0.008272016 | 0.014914402 |
| ACRC | -0.122171587 | 0.005500107 | 0.01025715 |
| ACRV1 | 0.260834841 | 1.86E-09 | 9.90E-09 |
| ACR | -0.229371733 | 1.42E-07 | 5.70E-07 |
| ACSBG1 | -0.272428632 | 3.24E-10 | 1.93E-09 |
| ACSBG2 | -0.117820136 | 0.007437433 | 0.013529251 |
| ACSF2 | -0.189215857 | 1.54E-05 | 4.49E-05 |
| ACSF3 | -0.172509499 | 8.32E-05 | 0.000214119 |
| ACSL1 | -0.147488173 | 0.000787122 | 0.001705104 |
| ACSL3 | 0.223810216 | 2.87E-07 | 1.10E-06 |
| ACSL4 | 0.165937156 | 0.000155062 | 0.000382261 |
| ACSL5 | -0.204149322 | 3.00E-06 | 9.72E-06 |
| ACSL6 | -0.234725914 | 7.07E-08 | 2.98E-07 |
| ACSM1 | -0.387605024 | 6.58E-20 | 1.68E-18 |
| ACSM2A | -0.101526761 | 0.021202026 | 0.035166052 |
| ACSM3 | -0.242326608 | 2.56E-08 | 1.15E-07 |
| ACSM5 | -0.361582231 | 2.37E-17 | 4.16E-16 |
| ACSS1 | -0.488674517 | 2.88E-32 | 4.79E-30 |
| ACSS2 | -0.291618012 | 1.49E-11 | 1.09E-10 |
| ACSS3 | -0.095468355 | 0.030295362 | 0.048591213 |
| ACTC1 | -0.142939018 | 0.001143393 | 0.002406997 |
| ACTG1 | 0.236380616 | 5.69E-08 | 2.42E-07 |
| ACTG2 | -0.138232692 | 0.001663795 | 0.003400055 |
| ACTL6A | 0.367031619 | 7.22E-18 | 1.38E-16 |
| ACTL7B | -0.119062561 | 0.006829986 | 0.012513085 |
| ACTL8 | 0.268877242 | 5.59E-10 | 3.23E-09 |
| ACTN1 | 0.158149846 | 0.000314603 | 0.000731706 |
| ACTN2 | -0.378004349 | 6.14E-19 | 1.38E-17 |
| ACTR10 | 0.248072044 | 1.16E-08 | 5.49E-08 |
| ACTR1A | 0.101906342 | 0.020721105 | 0.034428383 |
| ACTR1B | -0.165573731 | 0.000160381 | 0.000394548 |
| ACTR2 | 0.227041899 | 1.91E-07 | 7.52E-07 |
| ACTR3B | 0.220660675 | 4.24E-07 | 1.58E-06 |
| ACTR3 | 0.392775832 | 1.92E-20 | 5.30E-19 |
| ACTR6 | 0.301313711 | 2.86E-12 | 2.32E-11 |
| ACVR1B | -0.099655356 | 0.023717356 | 0.03888627 |
| ACVR1 | -0.099871579 | 0.023414122 | 0.038450937 |
| ACVR2A | -0.216819653 | 6.78E-07 | 2.44E-06 |
| ACVRL1 | -0.223437793 | 3.00E-07 | 1.15E-06 |
| ACY1 | 0.131822806 | 0.002723319 | 0.005359231 |
| ACYP1 | 0.123849578 | 0.004883672 | 0.009193335 |
| ACYP2 | -0.149131392 | 0.00068601 | 0.001504329 |
| ADAL | -0.11176336 | 0.011145373 | 0.019587946 |
| ADAM10 | 0.185581889 | 2.25E-05 | 6.37E-05 |
| ADAM11 | -0.12315712 | 0.00513008 | 0.009621849 |
| ADAM12 | 0.244779621 | 1.83E-08 | 8.42E-08 |
| ADAM17 | 0.232895751 | 8.99E-08 | 3.73E-07 |
| ADAM20 | -0.162484052 | 0.000213043 | 0.000513234 |
| ADAM21 | 0.136764272 | 0.00186605 | 0.003779753 |
| ADAM28 | -0.161275645 | 0.000237739 | 0.000566515 |
| ADAM29 | -0.106730573 | 0.015386926 | 0.026254407 |
| ADAM2 | 0.142965497 | 0.001140946 | 0.00240308 |
| ADAM32 | 0.152297943 | 0.000524263 | 0.001171096 |
| ADAM33 | -0.330974409 | 1.25E-14 | 1.45E-13 |
| ADAM8 | -0.169243817 | 0.000113714 | 0.000286732 |
| ADAM9 | 0.164477802 | 0.000177478 | 0.000433513 |
| ADAMTS10 | -0.286056063 | 3.73E-11 | 2.55E-10 |
| ADAMTS12 | 0.174655037 | 6.76E-05 | 0.000176526 |
| ADAMTS13 | -0.313386499 | 3.37E-13 | 3.17E-12 |
| ADAMTS15 | -0.107856888 | 0.014330827 | 0.024625565 |
| ADAMTS17 | -0.42153638 | 1.33E-23 | 6.01E-22 |
| ADAMTS1 | -0.203340045 | 3.29E-06 | 1.06E-05 |
| ADAMTS20 | 0.183915396 | 2.68E-05 | 7.48E-05 |
| ADAMTS4 | 0.240635416 | 3.22E-08 | 1.43E-07 |
| ADAMTS5 | 0.277299864 | 1.52E-10 | 9.50E-10 |
| ADAMTS6 | 0.159034462 | 0.000290776 | 0.000681043 |
| ADAMTS8 | -0.487566735 | 4.15E-32 | 6.75E-30 |
| ADAMTSL2 | -0.374912559 | 1.24E-18 | 2.66E-17 |
| ADAMTSL3 | -0.29680661 | 6.21E-12 | 4.79E-11 |
| ADAMTSL4 | -0.237406913 | 4.96E-08 | 2.13E-07 |
| ADARB1 | -0.293406421 | 1.10E-11 | 8.19E-11 |
| ADARB2 | -0.142367815 | 0.001197373 | 0.002510553 |
| ADAT2 | -0.146690075 | 0.000841056 | 0.001812315 |
| ADA | 0.277652801 | 1.43E-10 | 9.03E-10 |
| ADCK4 | -0.168393 | 0.000123229 | 0.000309085 |
| ADCY10 | -0.096388932 | 0.028728463 | 0.046308429 |
| ADCY1 | -0.1533667 | 0.000478215 | 0.001075566 |
| ADCY2 | -0.23471075 | 7.09E-08 | 2.98E-07 |
| ADCY4 | -0.300678739 | 3.20E-12 | 2.57E-11 |
| ADCY5 | -0.157843373 | 0.000323274 | 0.000750041 |
| ADCY6 | -0.304315519 | 1.70E-12 | 1.43E-11 |
| ADCY7 | -0.137142764 | 0.001811868 | 0.003679701 |
| ADCY9 | -0.436238807 | 2.44E-25 | 1.43E-23 |
| ADCYAP1R1 | -0.13500915 | 0.002137225 | 0.004292059 |
| ADCYAP1 | -0.185701343 | 2.23E-05 | 6.30E-05 |
| ADC | -0.291823614 | 1.44E-11 | 1.05E-10 |
| ADD1 | -0.256334756 | 3.59E-09 | 1.82E-08 |
| ADD2 | -0.129692641 | 0.003193152 | 0.006204407 |
| ADD3 | -0.130444481 | 0.003019511 | 0.005893945 |
| ADH1A | -0.366597437 | 7.94E-18 | 1.51E-16 |
| ADH1B | -0.404450351 | 1.09E-21 | 3.69E-20 |
| ADH1C | -0.185810872 | 2.20E-05 | 6.24E-05 |
| ADH5 | 0.110760948 | 0.011896441 | 0.020761903 |
| ADH7 | -0.103843449 | 0.018411713 | 0.030907309 |
| ADHFE1 | -0.411565723 | 1.79E-22 | 6.71E-21 |
| ADIPOR2 | 0.305458406 | 1.39E-12 | 1.19E-11 |
| ADM | 0.24038981 | 3.33E-08 | 1.47E-07 |
| ADNP2 | 0.132317658 | 0.002623609 | 0.005178306 |
| ADNP | 0.141329677 | 0.001301531 | 0.002712722 |
| ADORA1 | -0.17124171 | 9.40E-05 | 0.000240186 |
| ADORA2A | -0.288083675 | 2.67E-11 | 1.87E-10 |
| ADORA3 | -0.142792712 | 0.001157002 | 0.002433245 |
| ADO | 0.213896757 | 9.63E-07 | 3.39E-06 |
| ADPRHL2 | -0.111417913 | 0.011399321 | 0.020000845 |
| ADPRH | -0.28176614 | 7.46E-11 | 4.89E-10 |
| ADRA1A | -0.345908426 | 6.39E-16 | 9.08E-15 |
| ADRA1B | -0.129145346 | 0.003325207 | 0.006443454 |
| ADRA1D | -0.212690728 | 1.11E-06 | 3.87E-06 |
| ADRA2A | -0.216109304 | 7.38E-07 | 2.65E-06 |
| ADRA2B | -0.19757103 | 6.26E-06 | 1.93E-05 |
| ADRA2C | -0.161414358 | 0.000234774 | 0.000560317 |
| ADRB1 | -0.34734693 | 4.76E-16 | 6.92E-15 |
| ADRB2 | -0.411283464 | 1.93E-22 | 7.17E-21 |
| ADRB3 | -0.202101626 | 3.78E-06 | 1.21E-05 |
| ADRBK1 | -0.103564698 | 0.018729544 | 0.031401286 |
| ADRBK2 | -0.186022844 | 2.15E-05 | 6.12E-05 |
| ADRM1 | 0.133229393 | 0.002448569 | 0.004859213 |
| ADSL | 0.317445349 | 1.60E-13 | 1.58E-12 |
| ADSSL1 | 0.136969237 | 0.001836527 | 0.003725617 |
| ADSS | 0.128950197 | 0.00337348 | 0.006524346 |
| AEBP1 | -0.126281087 | 0.004100708 | 0.007815862 |
| AEBP2 | 0.141997376 | 0.001233625 | 0.002581688 |
| AEN | 0.217390744 | 6.32E-07 | 2.29E-06 |
| AES | -0.224676781 | 2.57E-07 | 9.93E-07 |
| AFARP1 | 0.180304097 | 3.86E-05 | 0.000105025 |
| AFF1 | -0.219399631 | 4.95E-07 | 1.83E-06 |
| AFF2 | -0.237084974 | 5.18E-08 | 2.22E-07 |
| AFF3 | -0.355351525 | 8.97E-17 | 1.43E-15 |
| AFG3L1 | -0.193719556 | 9.53E-06 | 2.86E-05 |
| AFG3L2 | 0.104794467 | 0.01736252 | 0.029273896 |
| AFMID | 0.095347842 | 0.030505808 | 0.0488856 |
| AFM | 0.111051611 | 0.011674183 | 0.02042037 |
| AFP | 0.198835246 | 5.45E-06 | 1.70E-05 |
| AG2 | -0.265416463 | 9.43E-10 | 5.27E-09 |
| AGAP11 | -0.376729785 | 8.22E-19 | 1.81E-17 |
| AGAP2 | -0.20490144 | 2.75E-06 | 8.98E-06 |
| AGAP3 | -0.234929511 | 6.89E-08 | 2.90E-07 |
| AGAP4 | -0.244263829 | 1.97E-08 | 9.01E-08 |
| AGAP5 | -0.341667177 | 1.51E-15 | 2.04E-14 |
| AGAP6 | -0.216006935 | 7.47E-07 | 2.67E-06 |
| AGAP7 | -0.154011315 | 0.00045229 | 0.001022203 |
| AGAP8 | -0.229273334 | 1.44E-07 | 5.76E-07 |
| AGBL1 | -0.15916307 | 0.000287455 | 0.000674177 |
| AGBL2 | -0.302343747 | 2.40E-12 | 1.96E-11 |
| AGBL3 | -0.172950134 | 7.98E-05 | 0.000205811 |
| AGBL4 | -0.118955939 | 0.006880315 | 0.012597209 |
| AGER | -0.357018304 | 6.30E-17 | 1.04E-15 |
| AGFG1 | 0.331389283 | 1.15E-14 | 1.34E-13 |
| AGFG2 | -0.215722765 | 7.73E-07 | 2.76E-06 |
| AGGF1 | -0.142881386 | 0.001148736 | 0.00241745 |
| AGMAT | 0.426000087 | 4.04E-24 | 1.98E-22 |
| AGPAT2 | -0.100216879 | 0.022936824 | 0.037758297 |
| AGPAT3 | -0.262180261 | 1.53E-09 | 8.25E-09 |
| AGPAT4 | -0.147965109 | 0.000756438 | 0.001644875 |
| AGPAT5 | 0.277396844 | 1.49E-10 | 9.38E-10 |
| AGPAT9 | 0.102579205 | 0.019891878 | 0.033163639 |
| AGPS | 0.33328423 | 7.96E-15 | 9.52E-14 |
| AGR3 | -0.236194826 | 5.83E-08 | 2.48E-07 |
| AGRN | -0.138420575 | 0.001639421 | 0.003352988 |
| AGRP | -0.226438525 | 2.06E-07 | 8.08E-07 |
| AGTR1 | -0.157071138 | 0.000346123 | 0.000798604 |
| AGTR2 | -0.167907544 | 0.000128988 | 0.000322519 |
| AGXT2L1 | 0.233807283 | 7.98E-08 | 3.33E-07 |
| AGXT2L2 | -0.435116481 | 3.34E-25 | 1.93E-23 |
| AHCTF1 | 0.219105107 | 5.13E-07 | 1.89E-06 |
| AHCYL1 | -0.127875735 | 0.003650844 | 0.007012596 |
| AHCYL2 | -0.138990513 | 0.001567475 | 0.003216703 |
| AHCY | 0.411207135 | 1.97E-22 | 7.30E-21 |
| AHDC1 | -0.320649158 | 8.87E-14 | 9.04E-13 |
| AHI1 | -0.141116591 | 0.001323916 | 0.002754494 |
| AHNAK2 | 0.151451899 | 0.000563602 | 0.001252114 |
| AHNAK | -0.222319864 | 3.45E-07 | 1.31E-06 |
| AHR | -0.179106884 | 4.35E-05 | 0.000117355 |
| AHSA1 | 0.437852165 | 1.56E-25 | 9.46E-24 |
| AHSA2 | -0.321224387 | 7.96E-14 | 8.17E-13 |
| AHSG | 0.201196233 | 4.19E-06 | 1.33E-05 |
| AIF1L | -0.119058817 | 0.006831747 | 0.012515165 |
| AIF1 | -0.149479899 | 0.000666175 | 0.001463563 |
| AIFM2 | 0.166011776 | 0.00015399 | 0.000379807 |
| AIFM3 | -0.153888566 | 0.000457123 | 0.001031961 |
| AIG1 | 0.134317311 | 0.002253679 | 0.004501052 |
| AIM1 | -0.154205006 | 0.000444759 | 0.00100712 |
| AIMP1 | 0.317516517 | 1.58E-13 | 1.56E-12 |
| AIMP2 | 0.455678426 | 9.08E-28 | 7.32E-26 |
| AIRE | -0.156700539 | 0.000357615 | 0.000822907 |
| AJAP1 | -0.199261148 | 5.20E-06 | 1.63E-05 |
| AK1 | -0.336637055 | 4.12E-15 | 5.19E-14 |
| AK3L1 | 0.30655824 | 1.14E-12 | 9.93E-12 |
| AKAP12 | 0.197624511 | 6.23E-06 | 1.92E-05 |
| AKAP13 | -0.399207851 | 4.01E-21 | 1.25E-19 |
| AKAP14 | -0.192628028 | 1.07E-05 | 3.20E-05 |
| AKAP1 | -0.241220091 | 2.97E-08 | 1.32E-07 |
| AKAP3 | -0.206977747 | 2.17E-06 | 7.20E-06 |
| AKAP6 | -0.156847706 | 0.000353009 | 0.000812898 |
| AKAP7 | -0.295136062 | 8.24E-12 | 6.26E-11 |
| AKAP8L | -0.208333032 | 1.85E-06 | 6.24E-06 |
| AKAP8 | -0.17093905 | 9.68E-05 | 0.000246863 |
| AKAP9 | -0.156482735 | 0.000364534 | 0.000836833 |
| AKD1 | -0.258680201 | 2.56E-09 | 1.33E-08 |
| AKIRIN1 | 0.181906252 | 3.28E-05 | 9.04E-05 |
| AKIRIN2 | 0.136916667 | 0.001844058 | 0.003739756 |
| AKNAD1 | 0.097204565 | 0.027398833 | 0.044354731 |
| AKNA | -0.37643851 | 8.78E-19 | 1.93E-17 |
| AKR1B10 | 0.216549027 | 7.00E-07 | 2.52E-06 |
| AKR1B15 | 0.26761973 | 6.77E-10 | 3.86E-09 |
| AKR1C1 | 0.155625909 | 0.000392982 | 0.000897296 |
| AKR1C2 | 0.211056574 | 1.35E-06 | 4.63E-06 |
| AKR1C3 | 0.156902874 | 0.000351297 | 0.000809328 |
| AKR1C4 | 0.251248954 | 7.43E-09 | 3.63E-08 |
| AKR1CL1 | 0.130162212 | 0.00308367 | 0.006009203 |
| AKR1D1 | 0.139322842 | 0.001526872 | 0.003142738 |
| AKR1E2 | 0.138429136 | 0.001638319 | 0.003351419 |
| AKR7A3 | 0.183537467 | 2.78E-05 | 7.75E-05 |
| AKT1 | -0.104909957 | 0.017238737 | 0.029087276 |
| AKTIP | -0.141470906 | 0.001286887 | 0.00268472 |
| ALAD | -0.285725935 | 3.93E-11 | 2.69E-10 |
| ALB | -0.101437795 | 0.021316137 | 0.035340655 |
| ALCAM | -0.131991556 | 0.002688936 | 0.005300439 |
| ALDH16A1 | -0.197355619 | 6.41E-06 | 1.98E-05 |
| ALDH18A1 | 0.342297257 | 1.33E-15 | 1.81E-14 |
| ALDH1A1 | -0.170481505 | 0.0001011 | 0.000257129 |
| ALDH1A2 | -0.11378788 | 0.00975537 | 0.017361797 |
| ALDH1B1 | 0.299829063 | 3.70E-12 | 2.94E-11 |
| ALDH1L2 | 0.242424059 | 2.53E-08 | 1.14E-07 |
| ALDH2 | -0.286883324 | 3.25E-11 | 2.25E-10 |
| ALDH3A2 | -0.102300597 | 0.020231661 | 0.033671144 |
| ALDH3B1 | -0.132686735 | 0.002551422 | 0.005047789 |
| ALDH3B2 | 0.10463174 | 0.017538249 | 0.029557717 |
| ALDH4A1 | -0.19791902 | 6.03E-06 | 1.87E-05 |
| ALDH5A1 | -0.231803264 | 1.04E-07 | 4.25E-07 |
| ALDH8A1 | -0.16364382 | 0.000191618 | 0.000465209 |
| ALDH9A1 | -0.103255269 | 0.019087949 | 0.031943228 |
| ALDOA | 0.255981081 | 3.78E-09 | 1.91E-08 |
| ALDOB | -0.217833618 | 5.99E-07 | 2.18E-06 |
| ALG10 | 0.323322818 | 5.37E-14 | 5.65E-13 |
| ALG12 | -0.208258277 | 1.87E-06 | 6.28E-06 |
| ALG13 | -0.111940152 | 0.011017356 | 0.019379998 |
| ALG14 | 0.212923336 | 1.08E-06 | 3.77E-06 |
| ALG1L2 | 0.111990341 | 0.010981252 | 0.01931989 |
| ALG1L | 0.106342237 | 0.015766571 | 0.02684346 |
| ALG1 | -0.122072451 | 0.005538627 | 0.010325133 |
| ALG3 | 0.24473049 | 1.84E-08 | 8.48E-08 |
| ALG6 | 0.157567818 | 0.000331261 | 0.000766435 |
| ALG8 | 0.336197654 | 4.49E-15 | 5.63E-14 |
| ALKBH1 | 0.185699346 | 2.23E-05 | 6.30E-05 |
| ALKBH2 | 0.332260286 | 9.72E-15 | 1.15E-13 |
| ALKBH3 | -0.104958342 | 0.017187108 | 0.029017308 |
| ALKBH6 | -0.121762992 | 0.005660436 | 0.010529163 |
| ALK | -0.17952504 | 4.18E-05 | 0.000112879 |
| ALMS1P | -0.191708786 | 1.18E-05 | 3.51E-05 |
| ALMS1 | -0.13556379 | 0.002047868 | 0.004125457 |
| ALOX12 | -0.097834762 | 0.026408074 | 0.042910705 |
| ALOX15B | -0.30513792 | 1.47E-12 | 1.25E-11 |
| ALOX15 | -0.245197528 | 1.73E-08 | 7.98E-08 |
| ALOX5AP | -0.169673554 | 0.000109175 | 0.000276298 |
| ALOX5 | -0.283735808 | 5.43E-11 | 3.63E-10 |
| ALOXE3 | 0.157652103 | 0.000328798 | 0.000761532 |
| ALPI | 0.096813467 | 0.028029637 | 0.045295205 |
| ALPK1 | -0.283041767 | 6.08E-11 | 4.03E-10 |
| ALPK2 | 0.119038981 | 0.006841088 | 0.012529979 |
| ALPL | -0.333294744 | 7.94E-15 | 9.51E-14 |
| ALS2CL | -0.2980374 | 5.03E-12 | 3.93E-11 |
| ALS2CR11 | -0.099821327 | 0.023484295 | 0.038556365 |
| ALS2CR12 | -0.300435143 | 3.33E-12 | 2.67E-11 |
| ALS2CR8 | -0.423901764 | 7.09E-24 | 3.31E-22 |
| ALX1 | 0.133024459 | 0.002486958 | 0.004927569 |
| AMAC1L2 | -0.109153648 | 0.013194317 | 0.022831618 |
| AMAC1L3 | -0.17633949 | 5.73E-05 | 0.000151118 |
| AMBN | 0.095281679 | 0.030621877 | 0.049051938 |
| AMBRA1 | -0.230277652 | 1.26E-07 | 5.12E-07 |
| AMD1 | 0.240865041 | 3.12E-08 | 1.39E-07 |
| AMDHD2 | -0.175657738 | 6.13E-05 | 0.000160934 |
| AMELY | 0.113124321 | 0.010192973 | 0.018066608 |
| AMFR | 0.116599165 | 0.008081007 | 0.014606907 |
| AMHR2 | -0.108925253 | 0.013388528 | 0.023129637 |
| AMH | -0.111533836 | 0.011313537 | 0.019864282 |
| AMICA1 | -0.362940234 | 1.77E-17 | 3.16E-16 |
| AMIGO1 | -0.351433502 | 2.04E-16 | 3.10E-15 |
| AMIGO3 | -0.138847815 | 0.001585211 | 0.003249861 |
| AMMECR1 | 0.187559379 | 1.83E-05 | 5.28E-05 |
| AMN1 | 0.131870173 | 0.002713628 | 0.005344895 |
| AMN | -0.097841342 | 0.026397894 | 0.042901138 |
| AMOTL1 | -0.182149058 | 3.20E-05 | 8.83E-05 |
| AMOTL2 | -0.200447999 | 4.55E-06 | 1.44E-05 |
| AMOT | -0.311802167 | 4.49E-13 | 4.14E-12 |
| AMPD1 | -0.207999078 | 1.93E-06 | 6.46E-06 |
| AMPD2 | -0.137353493 | 0.001782331 | 0.003623027 |
| AMPD3 | -0.212161574 | 1.18E-06 | 4.10E-06 |
| AMTN | -0.107281869 | 0.014861754 | 0.025476565 |
| AMT | -0.371749127 | 2.53E-18 | 5.15E-17 |
| AMY1A | -0.355805234 | 8.15E-17 | 1.32E-15 |
| AMY2A | -0.35582539 | 8.12E-17 | 1.32E-15 |
| AMY2B | -0.481679015 | 2.85E-31 | 4.01E-29 |
| AMZ2P1 | -0.203605885 | 3.19E-06 | 1.03E-05 |
| AMZ2 | 0.112548459 | 0.010586772 | 0.018697645 |
| ANAPC10 | 0.19221932 | 1.12E-05 | 3.33E-05 |
| ANAPC11 | 0.172274712 | 8.51E-05 | 0.000218596 |
| ANAPC1 | 0.232717696 | 9.20E-08 | 3.81E-07 |
| ANAPC2 | -0.266705016 | 7.77E-10 | 4.39E-09 |
| ANAPC4 | -0.096343487 | 0.02880415 | 0.046411717 |
| ANAPC5 | 0.30355775 | 1.94E-12 | 1.62E-11 |
| ANAPC7 | 0.363973118 | 1.41E-17 | 2.57E-16 |
| ANGEL2 | -0.132150473 | 0.002656918 | 0.00524146 |
| ANGPT1 | -0.242975009 | 2.34E-08 | 1.06E-07 |
| ANGPT2 | 0.240663577 | 3.21E-08 | 1.42E-07 |
| ANGPT4 | -0.239673003 | 3.66E-08 | 1.61E-07 |
| ANGPTL1 | -0.153770297 | 0.000461826 | 0.001041989 |
| ANGPTL3 | 0.100728023 | 0.022245763 | 0.036729581 |
| ANGPTL4 | 0.249176855 | 9.95E-09 | 4.77E-08 |
| ANGPTL5 | -0.35418913 | 1.15E-16 | 1.81E-15 |
| ANGPTL6 | -0.325011432 | 3.91E-14 | 4.23E-13 |
| ANGPTL7 | -0.301169224 | 2.94E-12 | 2.37E-11 |
| ANK2 | -0.177154752 | 5.29E-05 | 0.000140398 |
| ANK3 | -0.291789772 | 1.45E-11 | 1.06E-10 |
| ANKAR | -0.259607349 | 2.23E-09 | 1.17E-08 |
| ANKDD1A | -0.263866533 | 1.19E-09 | 6.53E-09 |
| ANKFN1 | -0.324702361 | 4.14E-14 | 4.46E-13 |
| ANKFY1 | -0.254996987 | 4.36E-09 | 2.19E-08 |
| ANKHD1-EIF4EBP3 | -0.286032402 | 3.74E-11 | 2.56E-10 |
| ANKHD1 | -0.281610941 | 7.65E-11 | 5.01E-10 |
| ANKH | -0.109215839 | 0.013141867 | 0.022758589 |
| ANKIB1 | 0.279126066 | 1.14E-10 | 7.25E-10 |
| ANKK1 | -0.381349416 | 2.85E-19 | 6.76E-18 |
| ANKLE1 | 0.128321579 | 0.003533335 | 0.006808475 |
| ANKLE2 | 0.221142918 | 3.99E-07 | 1.50E-06 |
| ANKMY1 | -0.395282377 | 1.05E-20 | 3.02E-19 |
| ANKMY2 | -0.15111329 | 0.000580101 | 0.001285768 |
| ANKRA2 | -0.133367604 | 0.002422984 | 0.004812263 |
| ANKRD10 | -0.294485314 | 9.20E-12 | 6.93E-11 |
| ANKRD11 | -0.186022713 | 2.15E-05 | 6.12E-05 |
| ANKRD12 | -0.184075553 | 2.63E-05 | 7.37E-05 |
| ANKRD13A | -0.225920448 | 2.20E-07 | 8.57E-07 |
| ANKRD13D | -0.136529427 | 0.00190041 | 0.003844287 |
| ANKRD20A3 | -0.186163965 | 2.12E-05 | 6.04E-05 |
| ANKRD20A4 | -0.147938268 | 0.000758135 | 0.001648206 |
| ANKRD20B | -0.172954158 | 7.97E-05 | 0.000205758 |
| ANKRD23 | -0.243438072 | 2.20E-08 | 9.99E-08 |
| ANKRD24 | -0.406345552 | 6.77E-22 | 2.36E-20 |
| ANKRD29 | -0.156314117 | 0.000369976 | 0.000848449 |
| ANKRD30B | 0.11238499 | 0.01070099 | 0.018876657 |
| ANKRD32 | 0.22883525 | 1.52E-07 | 6.07E-07 |
| ANKRD34A | -0.150659049 | 0.000602939 | 0.001333577 |
| ANKRD34B | -0.096067137 | 0.029268088 | 0.047094714 |
| ANKRD34C | -0.163969971 | 0.000185967 | 0.000452314 |
| ANKRD35 | -0.130784395 | 0.002943863 | 0.005758091 |
| ANKRD36 | -0.21047292 | 1.44E-06 | 4.93E-06 |
| ANKRD39 | 0.112220148 | 0.010817273 | 0.019068318 |
| ANKRD42 | -0.185881706 | 2.18E-05 | 6.20E-05 |
| ANKRD44 | -0.417098012 | 4.28E-23 | 1.78E-21 |
| ANKRD45 | -0.150158957 | 0.000629045 | 0.001386257 |
| ANKRD50 | -0.164494141 | 0.000177211 | 0.00043296 |
| ANKRD53 | -0.11950174 | 0.006626139 | 0.012166385 |
| ANKRD55 | -0.282988418 | 6.13E-11 | 4.06E-10 |
| ANKRD57 | 0.206909192 | 2.19E-06 | 7.25E-06 |
| ANKRD58 | -0.25778081 | 2.91E-09 | 1.50E-08 |
| ANKRD6 | -0.369021531 | 4.65E-18 | 9.14E-17 |
| ANKRD7 | 0.142176804 | 0.001215942 | 0.00254708 |
| ANKS1B | -0.240033877 | 3.49E-08 | 1.54E-07 |
| ANKS3 | -0.329564425 | 1.64E-14 | 1.88E-13 |
| ANKS6 | -0.218371111 | 5.61E-07 | 2.06E-06 |
| ANKZF1 | -0.165326126 | 0.000164103 | 0.00040296 |
| ANLN | 0.510763809 | 1.45E-35 | 4.68E-33 |
| ANO10 | 0.193544444 | 9.71E-06 | 2.91E-05 |
| ANO2 | -0.290202012 | 1.88E-11 | 1.35E-10 |
| ANO3 | 0.120090732 | 0.006361322 | 0.011713505 |
| ANO4 | -0.106635555 | 0.015479069 | 0.026396692 |
| ANO5 | -0.284857068 | 4.53E-11 | 3.07E-10 |
| ANO7 | 0.178014907 | 4.86E-05 | 0.000129939 |
| ANO8 | -0.19989067 | 4.84E-06 | 1.52E-05 |
| ANO9 | -0.276981832 | 1.59E-10 | 9.95E-10 |
| ANP32A | 0.146875476 | 0.000828231 | 0.00178699 |
| ANP32B | 0.279452345 | 1.08E-10 | 6.90E-10 |
| ANP32C | 0.134630062 | 0.002200335 | 0.004405078 |
| ANP32E | 0.245592775 | 1.64E-08 | 7.58E-08 |
| ANPEP | -0.118304742 | 0.007194976 | 0.013120443 |
| ANTXRL | -0.230073696 | 1.30E-07 | 5.25E-07 |
| ANXA11 | -0.169345078 | 0.000112629 | 0.00028421 |
| ANXA13 | 0.107285149 | 0.014858678 | 0.025473476 |
| ANXA2P1 | 0.135265189 | 0.002095541 | 0.004213402 |
| ANXA2P3 | 0.113133951 | 0.010186499 | 0.018056735 |
| ANXA4 | -0.102208535 | 0.020345041 | 0.033845749 |
| ANXA6 | -0.173569611 | 7.51E-05 | 0.000194684 |
| ANXA7 | 0.26711287 | 7.31E-10 | 4.15E-09 |
| ANXA9 | -0.158920987 | 0.000293735 | 0.00068733 |
| AOAH | -0.133257314 | 0.00244338 | 0.004849879 |
| AOC3 | -0.314949239 | 2.54E-13 | 2.43E-12 |
| AOX2P | 0.209408509 | 1.64E-06 | 5.54E-06 |
| AP1AR | 0.331655175 | 1.09E-14 | 1.28E-13 |
| AP1B1 | -0.196632629 | 6.94E-06 | 2.13E-05 |
| AP1G2 | -0.214165639 | 9.32E-07 | 3.29E-06 |
| AP1M1 | -0.19282575 | 1.05E-05 | 3.13E-05 |
| AP1M2 | -0.107980347 | 0.014219034 | 0.024458697 |
| AP1S1 | 0.205647019 | 2.53E-06 | 8.31E-06 |
| AP1S2 | -0.16179321 | 0.000226851 | 0.000543259 |
| AP1S3 | 0.217272186 | 6.41E-07 | 2.32E-06 |
| AP2A2 | -0.242917369 | 2.36E-08 | 1.07E-07 |
| AP2M1 | 0.10823087 | 0.01399454 | 0.024103651 |
| AP2S1 | 0.248354424 | 1.12E-08 | 5.30E-08 |
| AP3B2 | -0.205320762 | 2.62E-06 | 8.59E-06 |
| AP3M1 | 0.119378617 | 0.006682729 | 0.012265785 |
| AP3S1 | 0.229302819 | 1.43E-07 | 5.74E-07 |
| AP4B1 | -0.26460019 | 1.07E-09 | 5.90E-09 |
| AP4M1 | 0.141204528 | 0.001314635 | 0.00273661 |
| APBA1 | -0.264266782 | 1.12E-09 | 6.18E-09 |
| APBB1IP | -0.221738539 | 3.71E-07 | 1.40E-06 |
| APBB1 | -0.361036805 | 2.67E-17 | 4.66E-16 |
| APBB3 | -0.333417203 | 7.75E-15 | 9.31E-14 |
| APCDD1L | 0.315079898 | 2.48E-13 | 2.37E-12 |
| APCDD1 | -0.253303927 | 5.55E-09 | 2.75E-08 |
| APCS | 0.134016801 | 0.002306044 | 0.004597827 |
| APC | -0.200031752 | 4.77E-06 | 1.50E-05 |
| APEX1 | 0.365250594 | 1.07E-17 | 1.99E-16 |
| APEX2 | 0.177241187 | 5.24E-05 | 0.000139369 |
| APH1B | -0.210732497 | 1.40E-06 | 4.80E-06 |
| API5 | 0.172644925 | 8.22E-05 | 0.000211531 |
| APLP1 | -0.15813512 | 0.000315014 | 0.000732493 |
| APLP2 | -0.3246122 | 4.21E-14 | 4.53E-13 |
| APOA1BP | 0.224494689 | 2.63E-07 | 1.01E-06 |
| APOA2 | 0.177183015 | 5.27E-05 | 0.00014008 |
| APOB48R | -0.376003378 | 9.70E-19 | 2.11E-17 |
| APOBEC1 | 0.203922125 | 3.08E-06 | 9.96E-06 |
| APOBEC2 | -0.196394898 | 7.13E-06 | 2.18E-05 |
| APOBEC3B | 0.139713554 | 0.001480374 | 0.003054579 |
| APOBEC3C | -0.23055863 | 1.22E-07 | 4.95E-07 |
| APOBEC3D | -0.167344367 | 0.000135986 | 0.000338365 |
| APOBEC3F | -0.253572782 | 5.34E-09 | 2.66E-08 |
| APOBEC3G | -0.136681014 | 0.001878166 | 0.003801983 |
| APOBEC3H | -0.2455829 | 1.64E-08 | 7.59E-08 |
| APOBEC4 | -0.208744285 | 1.77E-06 | 5.96E-06 |
| APOC1 | -0.098343077 | 0.025631569 | 0.041759533 |
| APOC2 | -0.147261643 | 0.000802096 | 0.001735472 |
| APOC3 | 0.141388297 | 0.001295434 | 0.00270086 |
| APOC4 | -0.169210493 | 0.000114074 | 0.000287456 |
| APOD | -0.280957985 | 8.49E-11 | 5.53E-10 |
| APOE | -0.120801985 | 0.006054218 | 0.011190282 |
| APOF | 0.241239398 | 2.97E-08 | 1.32E-07 |
| APOH | -0.223248924 | 3.08E-07 | 1.17E-06 |
| APOL3 | -0.161388581 | 0.000235322 | 0.000561358 |
| APOOL | 0.106184432 | 0.015923177 | 0.027073193 |
| APOO | 0.230867812 | 1.17E-07 | 4.76E-07 |
| APRT | 0.136800425 | 0.001860811 | 0.003770436 |
| APTX | 0.237677661 | 4.79E-08 | 2.06E-07 |
| AQP10 | -0.288218632 | 2.61E-11 | 1.83E-10 |
| AQP11 | 0.15628973 | 0.000370769 | 0.000849878 |
| AQP1 | -0.321345469 | 7.79E-14 | 8.02E-13 |
| AQP2 | -0.24159713 | 2.83E-08 | 1.26E-07 |
| AQP3 | -0.256850235 | 3.33E-09 | 1.70E-08 |
| AQP4 | -0.300729842 | 3.17E-12 | 2.55E-11 |
| AQP5 | -0.305444135 | 1.39E-12 | 1.19E-11 |
| AQP6 | -0.207715027 | 1.99E-06 | 6.65E-06 |
| AQP7P1 | -0.108834015 | 0.013466812 | 0.023256838 |
| AQP7 | -0.176327637 | 5.74E-05 | 0.000151255 |
| AQP8 | -0.157128817 | 0.000344365 | 0.000794824 |
| AQP9 | 0.102874482 | 0.0195372 | 0.032629474 |
| AQPEP | 0.127662808 | 0.003708205 | 0.007117988 |
| AQR | 0.108090329 | 0.014120093 | 0.024303144 |
| ARAF | -0.180217294 | 3.90E-05 | 0.000105874 |
| ARAP1 | -0.365484422 | 1.01E-17 | 1.90E-16 |
| ARAP2 | -0.194040235 | 9.21E-06 | 2.77E-05 |
| ARAP3 | -0.331121686 | 1.21E-14 | 1.41E-13 |
| ARCN1 | 0.24530783 | 1.70E-08 | 7.87E-08 |
| ARC | -0.26712026 | 7.30E-10 | 4.14E-09 |
| ARF1 | 0.129657979 | 0.003201373 | 0.006218565 |
| ARF4 | 0.193454804 | 9.81E-06 | 2.94E-05 |
| ARF6 | 0.373873123 | 1.57E-18 | 3.32E-17 |
| ARFGAP2 | -0.324436277 | 4.36E-14 | 4.68E-13 |
| ARFGAP3 | 0.115935484 | 0.008451298 | 0.015199457 |
| ARFGEF2 | 0.126080759 | 0.004160634 | 0.007920262 |
| ARFIP2 | 0.170792183 | 9.81E-05 | 0.000250102 |
| ARFRP1 | -0.208188739 | 1.88E-06 | 6.33E-06 |
| ARG2 | 0.229626783 | 1.37E-07 | 5.53E-07 |
| ARGFXP2 | -0.227366646 | 1.83E-07 | 7.23E-07 |
| ARGLU1 | -0.341824295 | 1.46E-15 | 1.97E-14 |
| ARHGAP11A | 0.494980902 | 3.48E-33 | 6.89E-31 |
| ARHGAP11B | 0.334168676 | 6.69E-15 | 8.15E-14 |
| ARHGAP15 | -0.225963424 | 2.19E-07 | 8.53E-07 |
| ARHGAP17 | -0.218175302 | 5.75E-07 | 2.10E-06 |
| ARHGAP18 | -0.156448111 | 0.000365645 | 0.000839095 |
| ARHGAP1 | -0.239489618 | 3.76E-08 | 1.65E-07 |
| ARHGAP20 | -0.332976987 | 8.45E-15 | 1.01E-13 |
| ARHGAP21 | 0.123631521 | 0.00496009 | 0.009324894 |
| ARHGAP23 | -0.28950559 | 2.11E-11 | 1.51E-10 |
| ARHGAP24 | -0.38430241 | 1.43E-19 | 3.55E-18 |
| ARHGAP25 | -0.293734095 | 1.04E-11 | 7.79E-11 |
| ARHGAP27 | -0.323356122 | 5.34E-14 | 5.62E-13 |
| ARHGAP28 | -0.168371788 | 0.000123475 | 0.000309587 |
| ARHGAP30 | -0.329001885 | 1.82E-14 | 2.07E-13 |
| ARHGAP31 | -0.4003649 | 3.01E-21 | 9.52E-20 |
| ARHGAP33 | -0.222828316 | 3.24E-07 | 1.23E-06 |
| ARHGAP4 | -0.138578812 | 0.001619148 | 0.003314238 |
| ARHGAP5 | 0.16466485 | 0.000174444 | 0.000426832 |
| ARHGAP6 | -0.316017266 | 2.09E-13 | 2.02E-12 |
| ARHGAP8 | -0.266744397 | 7.73E-10 | 4.37E-09 |
| ARHGAP9 | -0.256950416 | 3.29E-09 | 1.67E-08 |
| ARHGDIB | -0.323769597 | 4.94E-14 | 5.24E-13 |
| ARHGEF10L | -0.279649662 | 1.05E-10 | 6.72E-10 |
| ARHGEF10 | -0.095408398 | 0.030399907 | 0.048731521 |
| ARHGEF11 | -0.211633805 | 1.26E-06 | 4.35E-06 |
| ARHGEF12 | -0.17835891 | 4.69E-05 | 0.00012582 |
| ARHGEF15 | -0.329259253 | 1.74E-14 | 1.98E-13 |
| ARHGEF17 | -0.424128971 | 6.67E-24 | 3.15E-22 |
| ARHGEF18 | -0.187136804 | 1.92E-05 | 5.50E-05 |
| ARHGEF19 | -0.3042115 | 1.73E-12 | 1.46E-11 |
| ARHGEF1 | -0.378884818 | 5.02E-19 | 1.14E-17 |
| ARHGEF2 | -0.377039862 | 7.66E-19 | 1.69E-17 |
| ARHGEF37 | -0.240466022 | 3.29E-08 | 1.46E-07 |
| ARHGEF38 | -0.122477275 | 0.005382843 | 0.010057231 |
| ARHGEF3 | -0.170477462 | 0.000101139 | 0.000257196 |
| ARHGEF4 | 0.183611401 | 2.76E-05 | 7.69E-05 |
| ARHGEF6 | -0.308899258 | 7.55E-13 | 6.74E-12 |
| ARHGEF7 | -0.259982936 | 2.11E-09 | 1.11E-08 |
| ARHGEF9 | -0.337882697 | 3.22E-15 | 4.13E-14 |
| ARID1A | -0.164795204 | 0.000172359 | 0.000422091 |
| ARID1B | -0.165866858 | 0.000156078 | 0.000384671 |
| ARID3A | 0.168984829 | 0.000116535 | 0.000293252 |
| ARID3C | 0.106438897 | 0.015671314 | 0.026694923 |
| ARID4A | -0.24704835 | 1.34E-08 | 6.27E-08 |
| ARID4B | -0.146076577 | 0.000884817 | 0.001899239 |
| ARID5A | -0.196926728 | 6.72E-06 | 2.06E-05 |
| ARID5B | -0.188200645 | 1.72E-05 | 4.96E-05 |
| ARL13A | -0.128973943 | 0.003367572 | 0.006516073 |
| ARL15 | -0.173852169 | 7.31E-05 | 0.000189786 |
| ARL17A | -0.175237702 | 6.39E-05 | 0.000167301 |
| ARL17B | -0.237308406 | 5.03E-08 | 2.16E-07 |
| ARL1 | 0.264583998 | 1.07E-09 | 5.92E-09 |
| ARL2 | 0.102257468 | 0.020284708 | 0.033750998 |
| ARL4A | 0.143253863 | 0.001114607 | 0.00235157 |
| ARL4D | 0.097080046 | 0.027598332 | 0.044663222 |
| ARL5A | 0.265437537 | 9.40E-10 | 5.26E-09 |
| ARL5B | 0.32750584 | 2.43E-14 | 2.70E-13 |
| ARL5C | -0.132411011 | 0.002605177 | 0.005143448 |
| ARL6IP1 | 0.357069505 | 6.23E-17 | 1.03E-15 |
| ARL6IP6 | 0.298743272 | 4.46E-12 | 3.49E-11 |
| ARL8A | -0.106194763 | 0.015912883 | 0.027060294 |
| ARL9 | 0.186949482 | 1.96E-05 | 5.60E-05 |
| ARMC10 | 0.244286594 | 1.96E-08 | 8.98E-08 |
| ARMC1 | 0.437381667 | 1.78E-25 | 1.07E-23 |
| ARMC2 | -0.243492485 | 2.18E-08 | 9.92E-08 |
| ARMC4 | -0.167516351 | 0.000133812 | 0.000333275 |
| ARMC5 | -0.314171886 | 2.92E-13 | 2.77E-12 |
| ARMC7 | -0.172666467 | 8.20E-05 | 0.000211146 |
| ARMC9 | -0.313720482 | 3.17E-13 | 2.99E-12 |
| ARMCX1 | -0.181795665 | 3.32E-05 | 9.13E-05 |
| ARMCX2 | -0.126500731 | 0.004035902 | 0.007701156 |
| ARMCX3 | -0.118609206 | 0.007046289 | 0.01287044 |
| ARMS2 | -0.11938131 | 0.006681487 | 0.012264631 |
| ARNT2 | -0.250901902 | 7.80E-09 | 3.80E-08 |
| ARNTL2 | 0.379164739 | 4.71E-19 | 1.08E-17 |
| ARNTL | -0.160515243 | 0.000254626 | 0.000603088 |
| ARPC1A | 0.331059012 | 1.23E-14 | 1.43E-13 |
| ARPC1B | 0.108665797 | 0.013612204 | 0.023491689 |
| ARPC3 | 0.349340646 | 3.15E-16 | 4.70E-15 |
| ARPC4 | 0.135981064 | 0.001982909 | 0.004002665 |
| ARPC5L | 0.183798204 | 2.71E-05 | 7.56E-05 |
| ARPC5 | 0.185031897 | 2.39E-05 | 6.72E-05 |
| ARPP19 | 0.222994719 | 3.17E-07 | 1.21E-06 |
| ARRB1 | -0.167860261 | 0.000129562 | 0.000323833 |
| ARRB2 | -0.300232042 | 3.45E-12 | 2.76E-11 |
| ARRDC1 | -0.198208537 | 5.84E-06 | 1.81E-05 |
| ARRDC2 | -0.199046481 | 5.32E-06 | 1.66E-05 |
| ARRDC3 | -0.200994426 | 4.28E-06 | 1.36E-05 |
| ARRDC4 | -0.222352407 | 3.44E-07 | 1.30E-06 |
| ARRDC5 | -0.21713269 | 6.52E-07 | 2.36E-06 |
| ARSA | -0.188538742 | 1.66E-05 | 4.80E-05 |
| ARSD | -0.245469361 | 1.67E-08 | 7.70E-08 |
| ARSG | -0.208316116 | 1.86E-06 | 6.25E-06 |
| ART4 | -0.328135902 | 2.15E-14 | 2.41E-13 |
| ARTN | 0.221689429 | 3.73E-07 | 1.40E-06 |
| ARV1 | -0.172695994 | 8.18E-05 | 0.000210627 |
| ARVCF | -0.339305517 | 2.42E-15 | 3.17E-14 |
| ARX | -0.143684045 | 0.001076356 | 0.002276635 |
| AR | -0.217295274 | 6.40E-07 | 2.32E-06 |
| ASAH1 | -0.207730023 | 1.99E-06 | 6.65E-06 |
| ASAH2B | 0.125963051 | 0.004196214 | 0.007981151 |
| ASAH2 | 0.153934788 | 0.000455298 | 0.00102842 |
| ASAM | 0.1544082 | 0.000436985 | 0.000991087 |
| ASAP1 | 0.160227372 | 0.000261306 | 0.000617594 |
| ASAP3 | -0.384015454 | 1.53E-19 | 3.78E-18 |
| ASB10 | 0.100249672 | 0.022891937 | 0.037693717 |
| ASB11 | -0.136803728 | 0.001860333 | 0.003770084 |
| ASB12 | -0.227368375 | 1.83E-07 | 7.23E-07 |
| ASB14 | -0.229452039 | 1.40E-07 | 5.64E-07 |
| ASB16 | -0.239610696 | 3.70E-08 | 1.62E-07 |
| ASB2 | -0.182733231 | 3.02E-05 | 8.36E-05 |
| ASB3 | -0.148474511 | 0.000724891 | 0.001582474 |
| ASB7 | 0.107737944 | 0.014439262 | 0.02479461 |
| ASB9 | 0.099670303 | 0.023696286 | 0.038858099 |
| ASCC1 | 0.236567653 | 5.55E-08 | 2.37E-07 |
| ASCC2 | -0.184981002 | 2.40E-05 | 6.75E-05 |
| ASCC3 | 0.115029782 | 0.008981013 | 0.01606951 |
| ASCL1 | 0.095231171 | 0.030710736 | 0.049174573 |
| ASCL3 | -0.144866645 | 0.000977351 | 0.002081544 |
| ASCL4 | -0.29218107 | 1.35E-11 | 9.95E-11 |
| ASF1A | 0.176984207 | 5.38E-05 | 0.000142558 |
| ASF1B | 0.395900136 | 9.00E-21 | 2.63E-19 |
| ASFMR1 | -0.113381042 | 0.010021645 | 0.017794496 |
| ASH1L | -0.200508475 | 4.52E-06 | 1.43E-05 |
| ASMTL | -0.271608417 | 3.68E-10 | 2.18E-09 |
| ASNA1 | 0.122974887 | 0.005196762 | 0.009735041 |
| ASNSD1 | 0.226373376 | 2.08E-07 | 8.14E-07 |
| ASNS | 0.336819844 | 3.97E-15 | 5.02E-14 |
| ASPA | -0.331688961 | 1.09E-14 | 1.27E-13 |
| ASPDH | -0.206131213 | 2.39E-06 | 7.88E-06 |
| ASPHD1 | -0.114277511 | 0.009443257 | 0.016843817 |
| ASPH | 0.18291697 | 2.96E-05 | 8.22E-05 |
| ASPM | 0.488815214 | 2.75E-32 | 4.65E-30 |
| ASPRV1 | -0.299673858 | 3.80E-12 | 3.01E-11 |
| ASRGL1 | -0.143638232 | 0.001080371 | 0.002284403 |
| ASS1 | -0.10319867 | 0.019154149 | 0.032037919 |
| ASTE1 | -0.166118109 | 0.000152475 | 0.000376303 |
| ASTL | -0.112019378 | 0.010960412 | 0.019295114 |
| ASTN1 | -0.144046494 | 0.001045071 | 0.002214916 |
| ASTN2 | -0.185773889 | 2.21E-05 | 6.26E-05 |
| ATAD1 | 0.249022961 | 1.02E-08 | 4.87E-08 |
| ATAD2B | -0.115507318 | 0.008698144 | 0.015601086 |
| ATAD2 | 0.396020985 | 8.74E-21 | 2.56E-19 |
| ATAD3A | 0.117695172 | 0.007501129 | 0.013635191 |
| ATAD3C | -0.256570014 | 3.47E-09 | 1.77E-08 |
| ATAD5 | 0.237958571 | 4.61E-08 | 1.99E-07 |
| ATE1 | 0.217911414 | 5.94E-07 | 2.17E-06 |
| ATF1 | 0.376132498 | 9.42E-19 | 2.06E-17 |
| ATF2 | 0.162675248 | 0.000209363 | 0.000504868 |
| ATF3 | -0.102271657 | 0.020267242 | 0.033724745 |
| ATF4 | 0.218525119 | 5.51E-07 | 2.02E-06 |
| ATF5 | 0.111805603 | 0.011114665 | 0.019535695 |
| ATF7IP2 | -0.260103432 | 2.08E-09 | 1.09E-08 |
| ATF7 | -0.13753259 | 0.001757574 | 0.003575249 |
| ATG12 | 0.176201264 | 5.81E-05 | 0.000153067 |
| ATG16L1 | 0.149777301 | 0.00064967 | 0.001428874 |
| ATG16L2 | -0.402394489 | 1.82E-21 | 6.01E-20 |
| ATG2A | -0.194601805 | 8.66E-06 | 2.62E-05 |
| ATG2B | -0.171469291 | 9.20E-05 | 0.000235312 |
| ATG3 | 0.09808024 | 0.026030586 | 0.042348963 |
| ATG4B | -0.174777555 | 6.68E-05 | 0.00017461 |
| ATG4C | 0.163175422 | 0.000200016 | 0.000483892 |
| ATG5 | 0.213332592 | 1.03E-06 | 3.61E-06 |
| ATHL1 | -0.169782011 | 0.000108057 | 0.000273849 |
| ATIC | 0.334674891 | 6.06E-15 | 7.45E-14 |
| ATL1 | 0.133064335 | 0.002479445 | 0.004913259 |
| ATL3 | 0.323058298 | 5.65E-14 | 5.92E-13 |
| ATMIN | 0.22752519 | 1.79E-07 | 7.10E-07 |
| ATM | -0.2352458 | 6.61E-08 | 2.79E-07 |
| ATN1 | -0.135057772 | 0.002129252 | 0.004276905 |
| ATOH7 | 0.096410333 | 0.02869288 | 0.046267095 |
| ATOH8 | -0.244928489 | 1.79E-08 | 8.26E-08 |
| ATOX1 | 0.155224693 | 0.000407003 | 0.000926979 |
| ATP10A | -0.197121398 | 6.58E-06 | 2.02E-05 |
| ATP10B | -0.175998935 | 5.93E-05 | 0.000156062 |
| ATP10D | -0.148353047 | 0.000732301 | 0.001597367 |
| ATP11A | -0.238651827 | 4.20E-08 | 1.83E-07 |
| ATP13A1 | -0.120450234 | 0.006204392 | 0.011446684 |
| ATP13A2 | -0.142400932 | 0.001194181 | 0.002504647 |
| ATP13A3 | 0.264891588 | 1.02E-09 | 5.67E-09 |
| ATP13A4 | -0.498962662 | 8.98E-34 | 1.97E-31 |
| ATP13A5 | -0.24496394 | 1.79E-08 | 8.23E-08 |
| ATP1A1 | -0.294745246 | 8.81E-12 | 6.64E-11 |
| ATP1A2 | -0.433286264 | 5.54E-25 | 3.10E-23 |
| ATP1A3 | -0.124641652 | 0.004614943 | 0.008721963 |
| ATP1A4 | -0.284747093 | 4.61E-11 | 3.11E-10 |
| ATP1B2 | -0.354418246 | 1.09E-16 | 1.73E-15 |
| ATP1B3 | 0.242612011 | 2.46E-08 | 1.11E-07 |
| ATP2A2 | 0.28504691 | 4.39E-11 | 2.98E-10 |
| ATP2B1 | 0.257707188 | 2.95E-09 | 1.51E-08 |
| ATP2B2 | -0.189127001 | 1.56E-05 | 4.53E-05 |
| ATP2B3 | -0.224785933 | 2.54E-07 | 9.80E-07 |
| ATP2B4 | -0.111976931 | 0.010990889 | 0.019335142 |
| ATP2C1 | 0.123892075 | 0.004868903 | 0.009170716 |
| ATP2C2 | -0.145500274 | 0.000927832 | 0.001983689 |
| ATP4B | -0.106563389 | 0.015549374 | 0.026503018 |
| ATP5A1 | 0.134395164 | 0.00224029 | 0.00447655 |
| ATP5B | 0.435251571 | 3.22E-25 | 1.87E-23 |
| ATP5C1 | 0.347344052 | 4.76E-16 | 6.92E-15 |
| ATP5EP2 | 0.14792439 | 0.000759014 | 0.001649937 |
| ATP5E | 0.168954932 | 0.000116865 | 0.000294045 |
| ATP5F1 | 0.245800719 | 1.59E-08 | 7.38E-08 |
| ATP5G1 | 0.205630049 | 2.53E-06 | 8.32E-06 |
| ATP5G2 | 0.2103523 | 1.46E-06 | 5.00E-06 |
| ATP5G3 | 0.359517622 | 3.70E-17 | 6.33E-16 |
| ATP5H | 0.196556287 | 7.00E-06 | 2.14E-05 |
| ATP5I | 0.130700421 | 0.002962389 | 0.005792061 |
| ATP5J2 | 0.311917405 | 4.39E-13 | 4.05E-12 |
| ATP5J | 0.16166509 | 0.000229502 | 0.000549112 |
| ATP5L | 0.174284488 | 7.01E-05 | 0.000182612 |
| ATP5SL | 0.096743916 | 0.028143116 | 0.045467555 |
| ATP5S | 0.267341704 | 7.06E-10 | 4.02E-09 |
| ATP6AP1L | -0.232681898 | 9.24E-08 | 3.83E-07 |
| ATP6AP1 | -0.122485601 | 0.005379681 | 0.010052263 |
| ATP6AP2 | 0.110464221 | 0.012127188 | 0.021131396 |
| ATP6V0A1 | -0.450110508 | 4.68E-27 | 3.45E-25 |
| ATP6V0A2 | 0.174739969 | 6.70E-05 | 0.000175227 |
| ATP6V0C | -0.106824691 | 0.015296131 | 0.02611592 |
| ATP6V0D1 | -0.217190435 | 6.48E-07 | 2.35E-06 |
| ATP6V0D2 | -0.206036641 | 2.42E-06 | 7.96E-06 |
| ATP6V0E1 | -0.109249564 | 0.013113501 | 0.022717338 |
| ATP6V0E2 | -0.121873144 | 0.005616805 | 0.010458193 |
| ATP6V1B1 | -0.105851928 | 0.016257624 | 0.027587859 |
| ATP6V1B2 | -0.166138532 | 0.000152186 | 0.000375681 |
| ATP6V1C1 | 0.214201517 | 9.28E-07 | 3.28E-06 |
| ATP6V1D | 0.207944392 | 1.94E-06 | 6.50E-06 |
| ATP6V1E2 | 0.104816049 | 0.017339329 | 0.029237262 |
| ATP6V1F | 0.154104293 | 0.00044866 | 0.001014804 |
| ATP6V1G1 | 0.140094101 | 0.001436339 | 0.002969542 |
| ATP6V1G2 | -0.11734323 | 0.007683145 | 0.013935636 |
| ATP6V1G3 | -0.118510631 | 0.007094125 | 0.012949531 |
| ATP6V1H | 0.097236569 | 0.027347758 | 0.044282808 |
| ATP7A | -0.182863599 | 2.98E-05 | 8.26E-05 |
| ATP8A1 | -0.332218666 | 9.80E-15 | 1.16E-13 |
| ATP8A2 | -0.270557255 | 4.33E-10 | 2.54E-09 |
| ATP8B2 | -0.257341792 | 3.11E-09 | 1.59E-08 |
| ATP8B3 | 0.100898073 | 0.022019896 | 0.036383705 |
| ATP8B4 | -0.24416717 | 1.99E-08 | 9.12E-08 |
| ATP8B5P | -0.13934683 | 0.001523979 | 0.003138344 |
| ATP9A | -0.222468721 | 3.39E-07 | 1.28E-06 |
| ATP9B | -0.207763135 | 1.98E-06 | 6.62E-06 |
| ATPAF1 | -0.103545605 | 0.018751488 | 0.031430167 |
| ATPBD4 | 0.240655607 | 3.21E-08 | 1.42E-07 |
| ATPIF1 | -0.158742539 | 0.000298446 | 0.00069721 |
| ATRNL1 | -0.20731493 | 2.09E-06 | 6.94E-06 |
| ATRX | -0.162961015 | 0.000203974 | 0.000492871 |
| ATXN1L | -0.233623051 | 8.17E-08 | 3.41E-07 |
| ATXN1 | -0.229483479 | 1.40E-07 | 5.62E-07 |
| ATXN7L1 | -0.192733394 | 1.06E-05 | 3.16E-05 |
| ATXN7L2 | -0.184277578 | 2.58E-05 | 7.22E-05 |
| ATXN7L3B | 0.218708811 | 5.39E-07 | 1.98E-06 |
| ATXN7L3 | 0.108841546 | 0.013460335 | 0.023247661 |
| ATXN7 | -0.30413486 | 1.75E-12 | 1.47E-11 |
| AUP1 | 0.141521735 | 0.001281654 | 0.00267492 |
| AURKAPS1 | 0.215643645 | 7.81E-07 | 2.79E-06 |
| AURKA | 0.546669009 | 1.81E-41 | 1.91E-38 |
| AURKB | 0.505380581 | 9.72E-35 | 2.62E-32 |
| AUTS2 | -0.379476711 | 4.38E-19 | 1.01E-17 |
| AVEN | 0.355489677 | 8.72E-17 | 1.40E-15 |
| AVIL | -0.222532249 | 3.36E-07 | 1.28E-06 |
| AVL9 | 0.317844061 | 1.49E-13 | 1.48E-12 |
| AVPR1A | 0.133627613 | 0.002375514 | 0.004727377 |
| AVPR1B | -0.161488264 | 0.000233208 | 0.000557113 |
| AVPR2 | -0.200364106 | 4.59E-06 | 1.45E-05 |
| AWAT1 | 0.103084626 | 0.019288146 | 0.032245857 |
| AXIN2 | -0.279216651 | 1.12E-10 | 7.15E-10 |
| AXL | -0.140127877 | 0.001432489 | 0.00296189 |
| AZGP1 | -0.101698195 | 0.020983635 | 0.03483273 |
| AZI1 | -0.146859781 | 0.000829309 | 0.001789008 |
| AZIN1 | 0.345895743 | 6.41E-16 | 9.10E-15 |
| AZU1 | -0.332300316 | 9.64E-15 | 1.14E-13 |
| B3GALNT1 | 0.13030038 | 0.003052112 | 0.005952347 |
| B3GALNT2 | 0.207439872 | 2.06E-06 | 6.85E-06 |
| B3GALT1 | 0.10816301 | 0.01405504 | 0.024199513 |
| B3GALT2 | -0.258704748 | 2.55E-09 | 1.32E-08 |
| B3GALT4 | -0.154997864 | 0.000415134 | 0.000944207 |
| B3GAT1 | -0.370615384 | 3.26E-18 | 6.53E-17 |
| B3GAT2 | -0.196660943 | 6.92E-06 | 2.12E-05 |
| B3GNT1 | -0.273615039 | 2.70E-10 | 1.63E-09 |
| B3GNT3 | 0.161535945 | 0.000232203 | 0.000554946 |
| B3GNT4 | 0.23218979 | 9.85E-08 | 4.06E-07 |
| B3GNT5 | 0.340348055 | 1.97E-15 | 2.61E-14 |
| B3GNT7 | -0.289331228 | 2.18E-11 | 1.54E-10 |
| B3GNT8 | -0.415995224 | 5.71E-23 | 2.33E-21 |
| B3GNT9 | -0.323786483 | 4.92E-14 | 5.23E-13 |
| B4GALNT1 | 0.139325505 | 0.001526551 | 0.0031424 |
| B4GALNT2 | 0.192796466 | 1.05E-05 | 3.14E-05 |
| B4GALNT3 | -0.186273037 | 2.10E-05 | 5.97E-05 |
| B4GALT1 | 0.356321773 | 7.31E-17 | 1.19E-15 |
| B4GALT2 | 0.201767118 | 3.93E-06 | 1.25E-05 |
| B4GALT3 | 0.180316042 | 3.86E-05 | 0.000104927 |
| B4GALT4 | 0.145327651 | 0.000941088 | 0.002009665 |
| B4GALT5 | 0.121711451 | 0.005680957 | 0.01056288 |
| B4GALT6 | 0.171488983 | 9.18E-05 | 0.000234953 |
| B4GALT7 | -0.101178051 | 0.021652348 | 0.035844553 |
| B9D2 | -0.191863509 | 1.16E-05 | 3.45E-05 |
| BAAT | -0.195281103 | 8.05E-06 | 2.44E-05 |
| BACE1 | -0.200354197 | 4.60E-06 | 1.45E-05 |
| BACH1 | 0.131708091 | 0.002746921 | 0.005403018 |
| BACH2 | -0.095341395 | 0.030517101 | 0.048899777 |
| BAD | -0.125986909 | 0.00418898 | 0.00796941 |
| BAG2 | 0.406433693 | 6.62E-22 | 2.31E-20 |
| BAG3 | 0.100533243 | 0.022506945 | 0.037105644 |
| BAG4 | 0.184102034 | 2.63E-05 | 7.35E-05 |
| BAG5 | 0.151314767 | 0.00057023 | 0.001265996 |
| BAGE | 0.14483835 | 0.000979618 | 0.002085928 |
| BAHCC1 | -0.289653679 | 2.06E-11 | 1.47E-10 |
| BAHD1 | -0.192346574 | 1.10E-05 | 3.29E-05 |
| BAI1 | -0.163264031 | 0.000198401 | 0.000480277 |
| BAI3 | -0.289643738 | 2.07E-11 | 1.47E-10 |
| BAIAP2L1 | 0.100702134 | 0.022280326 | 0.03677146 |
| BAIAP2L2 | 0.101903749 | 0.020724358 | 0.034430926 |
| BAIAP2 | -0.29475949 | 8.79E-12 | 6.63E-11 |
| BAIAP3 | -0.192842242 | 1.05E-05 | 3.13E-05 |
| BAK1 | 0.103462632 | 0.018847111 | 0.03156662 |
| BANF1 | 0.312138671 | 4.22E-13 | 3.91E-12 |
| BANF2 | 0.159735465 | 0.000273103 | 0.000642966 |
| BANK1 | -0.294941665 | 8.52E-12 | 6.45E-11 |
| BANP | 0.097420397 | 0.027055972 | 0.043863637 |
| BAP1 | -0.222592817 | 3.34E-07 | 1.27E-06 |
| BARD1 | 0.152529113 | 0.000513967 | 0.001149767 |
| BARX1 | 0.163765106 | 0.000189498 | 0.00046023 |
| BASP1 | 0.262213581 | 1.52E-09 | 8.21E-09 |
| BAT2L1 | -0.109220716 | 0.013137762 | 0.022754757 |
| BATF3 | 0.144417472 | 0.00101392 | 0.002155753 |
| BAZ1A | 0.161389115 | 0.000235311 | 0.000561358 |
| BAZ2B | -0.229544263 | 1.39E-07 | 5.58E-07 |
| BBC3 | -0.164178565 | 0.000182435 | 0.000444643 |
| BBOX1 | -0.1900313 | 1.41E-05 | 4.15E-05 |
| BBS1 | -0.413071369 | 1.22E-22 | 4.71E-21 |
| BBS2 | -0.297164241 | 5.84E-12 | 4.52E-11 |
| BBS4 | -0.139428858 | 0.001514125 | 0.00311939 |
| BBS5 | -0.377605185 | 6.73E-19 | 1.50E-17 |
| BBS7 | 0.195049764 | 8.25E-06 | 2.50E-05 |
| BBS9 | -0.187385123 | 1.87E-05 | 5.37E-05 |
| BCAM | -0.365159425 | 1.09E-17 | 2.02E-16 |
| BCAN | 0.198560928 | 5.61E-06 | 1.75E-05 |
| BCAR3 | 0.167707786 | 0.000131431 | 0.000328092 |
| BCAS2 | 0.163595564 | 0.000192468 | 0.00046687 |
| BCAS3 | -0.211787643 | 1.24E-06 | 4.27E-06 |
| BCAS4 | -0.103668649 | 0.018610464 | 0.031214732 |
| BCAT2 | -0.216874885 | 6.73E-07 | 2.43E-06 |
| BCCIP | 0.417659255 | 3.70E-23 | 1.56E-21 |
| BCHE | -0.199939683 | 4.82E-06 | 1.51E-05 |
| BCKDHA | -0.249223954 | 9.88E-09 | 4.74E-08 |
| BCKDHB | -0.145013446 | 0.000965666 | 0.002059071 |
| BCL10 | 0.115850281 | 0.008499916 | 0.015275698 |
| BCL11A | -0.209537914 | 1.61E-06 | 5.47E-06 |
| BCL11B | -0.179071103 | 4.37E-05 | 0.000117722 |
| BCL2L10 | 0.19095129 | 1.28E-05 | 3.78E-05 |
| BCL2L12 | 0.181880409 | 3.29E-05 | 9.06E-05 |
| BCL2L13 | 0.172838889 | 8.06E-05 | 0.000207928 |
| BCL2L14 | -0.16683434 | 0.00014263 | 0.000353927 |
| BCL2L15 | -0.170435005 | 0.000101549 | 0.000258173 |
| BCL2L2 | -0.200923666 | 4.32E-06 | 1.37E-05 |
| BCL2 | -0.21889686 | 5.26E-07 | 1.94E-06 |
| BCL6 | -0.276409781 | 1.75E-10 | 1.08E-09 |
| BCL7A | -0.100618831 | 0.022391854 | 0.036937226 |
| BCMO1 | -0.12787745 | 0.003650386 | 0.007012389 |
| BCO2 | -0.319060233 | 1.19E-13 | 1.20E-12 |
| BCORL1 | -0.118332789 | 0.007181162 | 0.013096449 |
| BCORL2 | 0.106337418 | 0.015771334 | 0.026847728 |
| BCOR | -0.095052586 | 0.031026697 | 0.049629466 |
| BCR | -0.142207272 | 0.001212962 | 0.002541905 |
| BDH2 | -0.2855621 | 4.04E-11 | 2.75E-10 |
| BDKRB1 | 0.209505192 | 1.62E-06 | 5.48E-06 |
| BDKRB2 | 0.101657387 | 0.021035443 | 0.034912932 |
| BDNFOS | -0.364214013 | 1.34E-17 | 2.45E-16 |
| BEND3 | 0.13143331 | 0.002804215 | 0.005503796 |
| BEND5 | -0.333692045 | 7.35E-15 | 8.88E-14 |
| BEND6 | 0.210303859 | 1.47E-06 | 5.02E-06 |
| BEND7 | -0.216029981 | 7.45E-07 | 2.67E-06 |
| BEST1 | -0.125580567 | 0.004313735 | 0.008189868 |
| BEST3 | 0.248541999 | 1.09E-08 | 5.17E-08 |
| BEST4 | -0.111879944 | 0.011060806 | 0.019453004 |
| BEX1 | -0.10385556 | 0.018398011 | 0.030886902 |
| BEX2 | -0.265489438 | 9.33E-10 | 5.22E-09 |
| BEX4 | -0.339947408 | 2.13E-15 | 2.81E-14 |
| BEX5 | -0.193018871 | 1.03E-05 | 3.07E-05 |
| BHLHA15 | 0.210688919 | 1.41E-06 | 4.82E-06 |
| BHLHE22 | -0.199168876 | 5.25E-06 | 1.64E-05 |
| BHLHE40 | -0.126358055 | 0.004077892 | 0.007774599 |
| BHLHE41 | -0.212283619 | 1.17E-06 | 4.04E-06 |
| BICC1 | -0.110032769 | 0.012469758 | 0.021673524 |
| BICD1 | 0.130288414 | 0.003054833 | 0.005956492 |
| BICD2 | -0.209199235 | 1.68E-06 | 5.67E-06 |
| BID | 0.147353971 | 0.000795961 | 0.001723132 |
| BIK | 0.164200934 | 0.00018206 | 0.000443832 |
| BIN2 | -0.210477906 | 1.44E-06 | 4.93E-06 |
| BIN3 | -0.147542808 | 0.00078355 | 0.00169847 |
| BIRC2 | 0.151304067 | 0.000570751 | 0.001267011 |
| BIRC5 | 0.504031941 | 1.56E-34 | 3.99E-32 |
| BIRC7 | -0.251873567 | 6.80E-09 | 3.33E-08 |
| BIVM | -0.126409335 | 0.004062755 | 0.007747218 |
| BLCAP | -0.187963394 | 1.76E-05 | 5.08E-05 |
| BLID | 0.167539741 | 0.000133519 | 0.00033264 |
| BLK | -0.263938916 | 1.18E-09 | 6.46E-09 |
| BLM | 0.411943513 | 1.63E-22 | 6.12E-21 |
| BLNK | -0.318715539 | 1.27E-13 | 1.27E-12 |
| BLOC1S2 | 0.180244362 | 3.89E-05 | 0.0001056 |
| BLVRA | -0.128679455 | 0.003441506 | 0.006646905 |
| BLZF1 | 0.183661126 | 2.75E-05 | 7.66E-05 |
| BMF | -0.23540868 | 6.46E-08 | 2.74E-07 |
| BMI1 | 0.098158313 | 0.025911507 | 0.042179266 |
| BMP15 | -0.100146498 | 0.023033421 | 0.037898586 |
| BMP2 | -0.10213132 | 0.020440562 | 0.033993339 |
| BMP3 | -0.334495923 | 6.28E-15 | 7.70E-14 |
| BMP4 | -0.120231798 | 0.006299324 | 0.011610556 |
| BMP5 | -0.180743579 | 3.69E-05 | 0.000100791 |
| BMP7 | -0.184679007 | 2.47E-05 | 6.95E-05 |
| BMPER | -0.231025595 | 1.15E-07 | 4.68E-07 |
| BMPR1A | 0.128021213 | 0.003612118 | 0.006945551 |
| BMPR1B | 0.10509823 | 0.017038599 | 0.028803072 |
| BMPR2 | -0.180314411 | 3.86E-05 | 0.00010493 |
| BMS1P4 | -0.225241258 | 2.40E-07 | 9.29E-07 |
| BMS1P5 | -0.272329766 | 3.29E-10 | 1.96E-09 |
| BMS1 | 0.216139413 | 7.36E-07 | 2.64E-06 |
| BMX | -0.231466143 | 1.08E-07 | 4.44E-07 |
| BNIP1 | 0.168654374 | 0.000120229 | 0.000302016 |
| BNIP3L | -0.141790283 | 0.00125433 | 0.002623369 |
| BNIP3 | 0.209054694 | 1.70E-06 | 5.76E-06 |
| BNIPL | -0.303021141 | 2.13E-12 | 1.77E-11 |
| BOC | -0.246773121 | 1.39E-08 | 6.51E-08 |
| BOD1L | -0.11439501 | 0.009369691 | 0.016718567 |
| BOD1 | 0.134818897 | 0.002168688 | 0.004347382 |
| BOK | -0.229170196 | 1.45E-07 | 5.83E-07 |
| BOLA1 | 0.106846369 | 0.015275286 | 0.026082557 |
| BOLA2 | 0.302299239 | 2.41E-12 | 1.98E-11 |
| BOLA3 | 0.41303913 | 1.23E-22 | 4.73E-21 |
| BOP1 | 0.289827948 | 2.00E-11 | 1.43E-10 |
| BPHL | 0.208269591 | 1.87E-06 | 6.28E-06 |
| BPI | -0.120321379 | 0.006260235 | 0.01154332 |
| BPNT1 | 0.161231385 | 0.000238692 | 0.000568448 |
| BPTF | -0.143447835 | 0.001097207 | 0.00231755 |
| BRAP | 0.22320703 | 3.09E-07 | 1.18E-06 |
| BRCA1 | 0.338722082 | 2.72E-15 | 3.54E-14 |
| BRCA2 | 0.247378081 | 1.28E-08 | 6.01E-08 |
| BRCC3 | 0.162674977 | 0.000209368 | 0.000504868 |
| BRD1 | -0.349090082 | 3.32E-16 | 4.93E-15 |
| BRD8 | -0.173135246 | 7.84E-05 | 0.000202473 |
| BREA2 | -0.176528454 | 5.63E-05 | 0.000148566 |
| BRF1 | -0.127048228 | 0.003878379 | 0.007421832 |
| BRF2 | 0.127340297 | 0.003796644 | 0.007275869 |
| BRI3BP | 0.397979677 | 5.42E-21 | 1.64E-19 |
| BRIP1 | 0.324307467 | 4.46E-14 | 4.77E-13 |
| BRIX1 | 0.373352261 | 1.77E-18 | 3.68E-17 |
| BRMS1L | 0.188696534 | 1.63E-05 | 4.72E-05 |
| BRMS1 | 0.147972391 | 0.000755978 | 0.001644054 |
| BRP44L | -0.157012113 | 0.00034793 | 0.000802125 |
| BRPF1 | -0.10466704 | 0.017499998 | 0.029498225 |
| BRWD1 | -0.303548604 | 1.94E-12 | 1.62E-11 |
| BSCL2 | -0.126943522 | 0.003908067 | 0.00747435 |
| BSDC1 | -0.289024267 | 2.29E-11 | 1.62E-10 |
| BSN | -0.20516081 | 2.67E-06 | 8.73E-06 |
| BTAF1 | -0.177008447 | 5.36E-05 | 0.000142274 |
| BTBD10 | 0.183975059 | 2.66E-05 | 7.44E-05 |
| BTBD12 | -0.137706064 | 0.001733896 | 0.003532835 |
| BTBD16 | 0.188718306 | 1.62E-05 | 4.71E-05 |
| BTBD18 | -0.19509385 | 8.21E-06 | 2.49E-05 |
| BTBD19 | -0.217718411 | 6.08E-07 | 2.21E-06 |
| BTBD1 | 0.170156962 | 0.000104273 | 0.000264696 |
| BTBD9 | -0.401797492 | 2.11E-21 | 6.88E-20 |
| BTC | -0.160823798 | 0.000247642 | 0.000588221 |
| BTD | -0.164214169 | 0.000181839 | 0.000443351 |
| BTF3L4 | 0.212987203 | 1.07E-06 | 3.75E-06 |
| BTF3 | 0.136723993 | 0.001871902 | 0.003790839 |
| BTG2 | -0.358990831 | 4.14E-17 | 7.03E-16 |
| BTG3 | -0.192110368 | 1.13E-05 | 3.36E-05 |
| BTG4 | -0.10816487 | 0.014053379 | 0.024198737 |
| BTK | -0.264025974 | 1.16E-09 | 6.39E-09 |
| BTLA | -0.204355509 | 2.93E-06 | 9.52E-06 |
| BTN1A1 | -0.141687318 | 0.001264742 | 0.002644317 |
| BTN2A1 | -0.250222199 | 8.59E-09 | 4.15E-08 |
| BTN2A2 | -0.398413585 | 4.87E-21 | 1.49E-19 |
| BTN2A3 | -0.264290774 | 1.12E-09 | 6.16E-09 |
| BTN3A1 | -0.218250601 | 5.70E-07 | 2.08E-06 |
| BTN3A2 | -0.199946338 | 4.81E-06 | 1.51E-05 |
| BTN3A3 | -0.243502489 | 2.18E-08 | 9.91E-08 |
| BTNL8 | -0.193994887 | 9.25E-06 | 2.78E-05 |
| BTNL9 | -0.48000762 | 4.88E-31 | 6.38E-29 |
| BUB1B | 0.507759501 | 4.21E-35 | 1.22E-32 |
| BUB1 | 0.474353926 | 2.96E-30 | 3.44E-28 |
| BUB3 | 0.473943881 | 3.37E-30 | 3.90E-28 |
| BUD13 | 0.120375134 | 0.006236884 | 0.011501323 |
| BUD31 | 0.275601077 | 1.98E-10 | 1.22E-09 |
| BYSL | 0.421142239 | 1.48E-23 | 6.58E-22 |
| BZRAP1 | -0.443797862 | 2.90E-26 | 1.95E-24 |
| BZW1 | 0.468943289 | 1.61E-29 | 1.65E-27 |
| BZW2 | 0.403009109 | 1.56E-21 | 5.20E-20 |
| C10orf105 | -0.46867111 | 1.76E-29 | 1.77E-27 |
| C10orf107 | -0.315817882 | 2.16E-13 | 2.09E-12 |
| C10orf10 | -0.172784685 | 8.11E-05 | 0.000208858 |
| C10orf110 | -0.150485316 | 0.000611892 | 0.001351137 |
| C10orf111 | -0.168899312 | 0.00011748 | 0.00029552 |
| C10orf116 | -0.247890526 | 1.19E-08 | 5.63E-08 |
| C10orf118 | -0.103723342 | 0.018548077 | 0.031120538 |
| C10orf119 | 0.359365397 | 3.82E-17 | 6.52E-16 |
| C10orf11 | -0.243872709 | 2.07E-08 | 9.46E-08 |
| C10orf128 | -0.226542536 | 2.03E-07 | 7.98E-07 |
| C10orf12 | 0.1735437 | 7.53E-05 | 0.000195098 |
| C10orf137 | -0.095999369 | 0.029382832 | 0.047267931 |
| C10orf140 | -0.148991198 | 0.000694142 | 0.001520494 |
| C10orf18 | 0.164311388 | 0.000180219 | 0.000439885 |
| C10orf26 | -0.133574764 | 0.002385094 | 0.004745023 |
| C10orf28 | -0.14106563 | 0.001329321 | 0.002763728 |
| C10orf2 | 0.386392237 | 8.77E-20 | 2.20E-18 |
| C10orf32 | -0.176929754 | 5.41E-05 | 0.000143213 |
| C10orf46 | 0.207831931 | 1.96E-06 | 6.58E-06 |
| C10orf50 | -0.269893327 | 4.79E-10 | 2.79E-09 |
| C10orf53 | -0.162239453 | 0.000217839 | 0.000523715 |
| C10orf54 | -0.330439741 | 1.38E-14 | 1.60E-13 |
| C10orf67 | -0.26136606 | 1.72E-09 | 9.20E-09 |
| C10orf68 | -0.314518212 | 2.74E-13 | 2.61E-12 |
| C10orf72 | -0.295253788 | 8.08E-12 | 6.14E-11 |
| C10orf76 | -0.290404333 | 1.82E-11 | 1.31E-10 |
| C10orf78 | 0.22517369 | 2.42E-07 | 9.36E-07 |
| C10orf79 | -0.231679006 | 1.05E-07 | 4.32E-07 |
| C10orf81 | -0.169834606 | 0.000107519 | 0.000272623 |
| C10orf84 | 0.221712889 | 3.72E-07 | 1.40E-06 |
| C10orf88 | 0.258077986 | 2.79E-09 | 1.44E-08 |
| C10orf90 | 0.294051259 | 9.90E-12 | 7.41E-11 |
| C10orf93 | -0.178871972 | 4.46E-05 | 0.000119996 |
| C10orf95 | -0.177085305 | 5.32E-05 | 0.000141328 |
| C11orf10 | 0.255840091 | 3.86E-09 | 1.95E-08 |
| C11orf16 | -0.217759546 | 6.05E-07 | 2.20E-06 |
| C11orf20 | 0.102703539 | 0.019741854 | 0.032943745 |
| C11orf21 | -0.389289475 | 4.41E-20 | 1.16E-18 |
| C11orf24 | 0.321420712 | 7.68E-14 | 7.92E-13 |
| C11orf2 | -0.156973479 | 0.000349117 | 0.000804584 |
| C11orf31 | 0.144738801 | 0.000987633 | 0.002102099 |
| C11orf35 | -0.325807289 | 3.36E-14 | 3.67E-13 |
| C11orf42 | -0.17307345 | 7.88E-05 | 0.000203582 |
| C11orf45 | -0.20066202 | 4.44E-06 | 1.40E-05 |
| C11orf48 | 0.286979202 | 3.20E-11 | 2.22E-10 |
| C11orf49 | -0.151460731 | 0.000563177 | 0.001251589 |
| C11orf51 | 0.134977439 | 0.00214244 | 0.004300968 |
| C11orf52 | -0.252944733 | 5.84E-09 | 2.89E-08 |
| C11orf57 | 0.121696508 | 0.005686918 | 0.010572981 |
| C11orf58 | 0.139977126 | 0.001449744 | 0.00299509 |
| C11orf59 | 0.172412546 | 8.40E-05 | 0.00021599 |
| C11orf61 | -0.326712742 | 2.83E-14 | 3.12E-13 |
| C11orf63 | -0.211624369 | 1.26E-06 | 4.35E-06 |
| C11orf65 | -0.189216345 | 1.54E-05 | 4.49E-05 |
| C11orf66 | -0.317659703 | 1.54E-13 | 1.52E-12 |
| C11orf67 | -0.105861028 | 0.01624839 | 0.02757453 |
| C11orf68 | -0.109700266 | 0.012739564 | 0.022104029 |
| C11orf70 | -0.115450961 | 0.008731109 | 0.015654593 |
| C11orf71 | -0.110024262 | 0.012476598 | 0.021679757 |
| C11orf73 | 0.223314651 | 3.05E-07 | 1.17E-06 |
| C11orf74 | -0.102339813 | 0.02018353 | 0.033600835 |
| C11orf80 | 0.105085492 | 0.017052076 | 0.028821154 |
| C11orf82 | 0.437764532 | 1.60E-25 | 9.66E-24 |
| C11orf83 | 0.205675193 | 2.52E-06 | 8.28E-06 |
| C11orf84 | 0.168718086 | 0.000119508 | 0.000300319 |
| C11orf86 | 0.211165407 | 1.33E-06 | 4.58E-06 |
| C11orf88 | -0.22622352 | 2.12E-07 | 8.28E-07 |
| C11orf92 | -0.415131674 | 7.15E-23 | 2.86E-21 |
| C11orf93 | -0.365454978 | 1.02E-17 | 1.91E-16 |
| C11orf9 | -0.095021366 | 0.031082217 | 0.049705685 |
| C12orf10 | 0.185192573 | 2.35E-05 | 6.61E-05 |
| C12orf11 | 0.444439775 | 2.41E-26 | 1.65E-24 |
| C12orf23 | 0.432209245 | 7.45E-25 | 4.06E-23 |
| C12orf24 | 0.269271825 | 5.27E-10 | 3.05E-09 |
| C12orf27 | -0.125493773 | 0.004340814 | 0.008238932 |
| C12orf29 | 0.405215898 | 9.00E-22 | 3.08E-20 |
| C12orf32 | 0.344020999 | 9.39E-16 | 1.30E-14 |
| C12orf34 | 0.247889489 | 1.19E-08 | 5.63E-08 |
| C12orf36 | 0.101172806 | 0.021659184 | 0.035849932 |
| C12orf39 | 0.248262363 | 1.13E-08 | 5.36E-08 |
| C12orf41 | 0.241682809 | 2.79E-08 | 1.25E-07 |
| C12orf43 | 0.292689732 | 1.24E-11 | 9.18E-11 |
| C12orf44 | 0.298516531 | 4.64E-12 | 3.63E-11 |
| C12orf45 | 0.324875967 | 4.01E-14 | 4.33E-13 |
| C12orf47 | 0.16400279 | 0.000185407 | 0.000451226 |
| C12orf48 | 0.547575294 | 1.26E-41 | 1.48E-38 |
| C12orf4 | 0.221322537 | 3.91E-07 | 1.46E-06 |
| C12orf51 | -0.144202561 | 0.00103186 | 0.002189238 |
| C12orf52 | 0.239059878 | 3.98E-08 | 1.74E-07 |
| C12orf56 | 0.214437155 | 9.03E-07 | 3.19E-06 |
| C12orf57 | 0.12029009 | 0.006273863 | 0.011567381 |
| C12orf59 | -0.171012395 | 9.61E-05 | 0.000245201 |
| C12orf5 | 0.296676056 | 6.35E-12 | 4.89E-11 |
| C12orf60 | 0.121503132 | 0.005764578 | 0.010700444 |
| C12orf63 | -0.191640143 | 1.19E-05 | 3.53E-05 |
| C12orf65 | 0.10543558 | 0.016685071 | 0.028246058 |
| C12orf66 | 0.287402729 | 2.99E-11 | 2.08E-10 |
| C12orf68 | -0.151570746 | 0.000557915 | 0.001240307 |
| C12orf69 | -0.324232616 | 4.53E-14 | 4.83E-13 |
| C12orf70 | 0.236831989 | 5.36E-08 | 2.29E-07 |
| C12orf71 | -0.244941592 | 1.79E-08 | 8.25E-08 |
| C12orf72 | -0.238001995 | 4.58E-08 | 1.98E-07 |
| C12orf73 | 0.323962238 | 4.76E-14 | 5.07E-13 |
| C12orf74 | -0.153285047 | 0.000481596 | 0.001082804 |
| C12orf76 | -0.16083441 | 0.000247406 | 0.000587728 |
| C13orf15 | -0.31815488 | 1.41E-13 | 1.40E-12 |
| C13orf18 | -0.175154978 | 6.44E-05 | 0.000168617 |
| C13orf1 | 0.131652687 | 0.002758387 | 0.005422901 |
| C13orf23 | 0.105355897 | 0.016767992 | 0.028379223 |
| C13orf26 | -0.151595904 | 0.000556718 | 0.001237922 |
| C13orf27 | 0.344739408 | 8.11E-16 | 1.13E-14 |
| C13orf29 | 0.213182115 | 1.05E-06 | 3.67E-06 |
| C13orf30 | -0.274278571 | 2.43E-10 | 1.48E-09 |
| C13orf33 | 0.129614255 | 0.003211769 | 0.006237547 |
| C13orf34 | 0.403220828 | 1.48E-21 | 4.94E-20 |
| C13orf36 | -0.222132386 | 3.53E-07 | 1.34E-06 |
| C13orf37 | 0.395480374 | 9.97E-21 | 2.89E-19 |
| C13orf38 | 0.152945892 | 0.000495877 | 0.001112163 |
| C13orf39 | -0.143922004 | 0.00105572 | 0.002234643 |
| C14orf101 | 0.137448625 | 0.001769141 | 0.003597314 |
| C14orf102 | -0.115641762 | 0.008619951 | 0.015467778 |
| C14orf104 | 0.478159754 | 8.84E-31 | 1.10E-28 |
| C14orf106 | 0.27126885 | 3.88E-10 | 2.29E-09 |
| C14orf109 | 0.136902544 | 0.001846086 | 0.00374311 |
| C14orf115 | -0.096529655 | 0.028495181 | 0.045969465 |
| C14orf118 | 0.207073838 | 2.14E-06 | 7.12E-06 |
| C14orf119 | 0.299657266 | 3.81E-12 | 3.02E-11 |
| C14orf126 | 0.337137285 | 3.73E-15 | 4.74E-14 |
| C14orf128 | 0.134052514 | 0.002299763 | 0.004587134 |
| C14orf129 | 0.430150712 | 1.31E-24 | 6.77E-23 |
| C14orf132 | -0.294778147 | 8.76E-12 | 6.61E-11 |
| C14orf135 | 0.141258153 | 0.001309006 | 0.002726005 |
| C14orf138 | 0.113270648 | 0.010095003 | 0.017908841 |
| C14orf139 | -0.341938946 | 1.43E-15 | 1.93E-14 |
| C14orf142 | 0.141922131 | 0.001241111 | 0.00259681 |
| C14orf143 | 0.19750255 | 6.31E-06 | 1.95E-05 |
| C14orf145 | 0.204308637 | 2.94E-06 | 9.57E-06 |
| C14orf147 | 0.318666666 | 1.28E-13 | 1.28E-12 |
| C14orf148 | -0.136666029 | 0.001880354 | 0.003806027 |
| C14orf149 | 0.16326417 | 0.000198399 | 0.000480277 |
| C14orf153 | 0.286106942 | 3.70E-11 | 2.54E-10 |
| C14orf156 | 0.370485238 | 3.36E-18 | 6.72E-17 |
| C14orf159 | -0.271892009 | 3.52E-10 | 2.09E-09 |
| C14orf166 | 0.519727972 | 5.66E-37 | 2.26E-34 |
| C14orf169 | 0.243454192 | 2.20E-08 | 9.97E-08 |
| C14orf174 | -0.095195754 | 0.030773178 | 0.049258771 |
| C14orf176 | -0.156211726 | 0.000373317 | 0.000855425 |
| C14orf178 | -0.180975064 | 3.61E-05 | 9.87E-05 |
| C14orf179 | 0.134530083 | 0.002217261 | 0.004435854 |
| C14orf180 | -0.260784778 | 1.88E-09 | 9.96E-09 |
| C14orf181 | -0.150818674 | 0.00059482 | 0.001316348 |
| C14orf1 | 0.140008429 | 0.001446146 | 0.002987964 |
| C14orf23 | 0.165044044 | 0.000168442 | 0.000412955 |
| C14orf2 | 0.314510859 | 2.75E-13 | 2.61E-12 |
| C14orf33 | 0.409658983 | 2.92E-22 | 1.06E-20 |
| C14orf34 | 0.225295694 | 2.38E-07 | 9.23E-07 |
| C14orf37 | 0.136799922 | 0.001860884 | 0.003770436 |
| C14orf43 | -0.124209076 | 0.004760002 | 0.008981678 |
| C14orf45 | -0.106700964 | 0.015415588 | 0.026299655 |
| C14orf49 | -0.188738371 | 1.62E-05 | 4.70E-05 |
| C14orf4 | -0.097339648 | 0.027183812 | 0.044038745 |
| C14orf53 | 0.138310719 | 0.001653633 | 0.003381016 |
| C14orf64 | -0.33796982 | 3.16E-15 | 4.07E-14 |
| C14orf73 | -0.291122681 | 1.62E-11 | 1.17E-10 |
| C14orf80 | 0.17001946 | 0.000105646 | 0.000268079 |
| C15orf17 | -0.387750537 | 6.36E-20 | 1.63E-18 |
| C15orf21 | 0.123546174 | 0.004990292 | 0.009376382 |
| C15orf23 | 0.500146477 | 5.98E-34 | 1.39E-31 |
| C15orf24 | 0.158904128 | 0.000294177 | 0.000688284 |
| C15orf26 | -0.177163276 | 5.28E-05 | 0.000140317 |
| C15orf27 | -0.267400748 | 7.00E-10 | 3.98E-09 |
| C15orf28 | -0.231525462 | 1.07E-07 | 4.40E-07 |
| C15orf29 | 0.146521223 | 0.000852896 | 0.001835256 |
| C15orf2 | -0.148196201 | 0.000741973 | 0.001616937 |
| C15orf34 | -0.357118923 | 6.17E-17 | 1.02E-15 |
| C15orf37 | -0.189225222 | 1.54E-05 | 4.49E-05 |
| C15orf38 | -0.153618244 | 0.000467938 | 0.001054113 |
| C15orf41 | 0.222515844 | 3.37E-07 | 1.28E-06 |
| C15orf42 | 0.395769331 | 9.29E-21 | 2.71E-19 |
| C15orf44 | 0.273819914 | 2.61E-10 | 1.58E-09 |
| C15orf48 | 0.23413596 | 7.64E-08 | 3.20E-07 |
| C15orf51 | -0.345677684 | 6.70E-16 | 9.48E-15 |
| C15orf52 | -0.245139256 | 1.74E-08 | 8.04E-08 |
| C15orf56 | -0.269891588 | 4.79E-10 | 2.79E-09 |
| C15orf59 | -0.476385597 | 1.56E-30 | 1.89E-28 |
| C15orf60 | 0.105321417 | 0.016803984 | 0.028435322 |
| C15orf61 | 0.129223875 | 0.003305959 | 0.006407399 |
| C15orf62 | -0.334061388 | 6.84E-15 | 8.30E-14 |
| C15orf63 | 0.290415042 | 1.82E-11 | 1.31E-10 |
| C16orf3 | -0.175266921 | 6.37E-05 | 0.000166903 |
| C16orf48 | -0.321264976 | 7.90E-14 | 8.12E-13 |
| C16orf52 | -0.158008086 | 0.000318586 | 0.000739938 |
| C16orf54 | -0.299517057 | 3.91E-12 | 3.09E-11 |
| C16orf55 | -0.137543402 | 0.00175609 | 0.003572593 |
| C16orf57 | 0.102532056 | 0.019949028 | 0.033242281 |
| C16orf58 | -0.219000993 | 5.20E-07 | 1.92E-06 |
| C16orf59 | 0.332055133 | 1.01E-14 | 1.19E-13 |
| C16orf5 | -0.389905418 | 3.81E-20 | 1.02E-18 |
| C16orf61 | 0.34748068 | 4.63E-16 | 6.74E-15 |
| C16orf63 | 0.194532139 | 8.73E-06 | 2.64E-05 |
| C16orf71 | -0.279342134 | 1.10E-10 | 7.01E-10 |
| C16orf73 | 0.147115829 | 0.000811873 | 0.001755107 |
| C16orf75 | 0.22717264 | 1.88E-07 | 7.40E-07 |
| C16orf79 | -0.245962193 | 1.56E-08 | 7.23E-08 |
| C16orf80 | 0.116086561 | 0.008365702 | 0.015067007 |
| C16orf81 | -0.188172913 | 1.72E-05 | 4.97E-05 |
| C16orf86 | -0.327589256 | 2.39E-14 | 2.66E-13 |
| C16orf87 | 0.282617369 | 6.51E-11 | 4.30E-10 |
| C16orf88 | 0.307537147 | 9.62E-13 | 8.45E-12 |
| C16orf89 | -0.430237942 | 1.28E-24 | 6.64E-23 |
| C16orf91 | 0.159357304 | 0.000282508 | 0.000663935 |
| C16orf93 | -0.319050029 | 1.19E-13 | 1.20E-12 |
| C17orf101 | -0.129381743 | 0.003267571 | 0.00633607 |
| C17orf102 | -0.170554223 | 0.000100401 | 0.000255482 |
| C17orf103 | -0.451304084 | 3.30E-27 | 2.51E-25 |
| C17orf104 | 0.116860194 | 0.007939385 | 0.014369107 |
| C17orf105 | -0.10614269 | 0.015964829 | 0.027137863 |
| C17orf107 | -0.185625 | 2.24E-05 | 6.35E-05 |
| C17orf108 | -0.425490898 | 4.63E-24 | 2.24E-22 |
| C17orf28 | -0.156558014 | 0.000362129 | 0.000832076 |
| C17orf37 | 0.126115646 | 0.004150141 | 0.00790104 |
| C17orf39 | -0.141822004 | 0.001251138 | 0.002617241 |
| C17orf42 | 0.215742326 | 7.72E-07 | 2.75E-06 |
| C17orf44 | -0.197728133 | 6.16E-06 | 1.90E-05 |
| C17orf46 | -0.150848024 | 0.000593338 | 0.001313214 |
| C17orf47 | -0.240789363 | 3.15E-08 | 1.40E-07 |
| C17orf50 | -0.396178173 | 8.41E-21 | 2.47E-19 |
| C17orf53 | 0.44418036 | 2.60E-26 | 1.76E-24 |
| C17orf54 | -0.20038178 | 4.59E-06 | 1.45E-05 |
| C17orf55 | -0.200831248 | 4.36E-06 | 1.38E-05 |
| C17orf56 | -0.271807953 | 3.57E-10 | 2.12E-09 |
| C17orf57 | -0.199944685 | 4.81E-06 | 1.51E-05 |
| C17orf58 | 0.277082705 | 1.57E-10 | 9.80E-10 |
| C17orf59 | -0.120205049 | 0.006311038 | 0.011630558 |
| C17orf60 | -0.14061664 | 0.001377833 | 0.002858342 |
| C17orf61 | 0.188989455 | 1.58E-05 | 4.59E-05 |
| C17orf65 | -0.324396531 | 4.39E-14 | 4.71E-13 |
| C17orf66 | -0.157103391 | 0.000345139 | 0.000796426 |
| C17orf68 | -0.24180966 | 2.75E-08 | 1.23E-07 |
| C17orf69 | -0.260553365 | 1.94E-09 | 1.03E-08 |
| C17orf70 | -0.143562395 | 0.001087048 | 0.002298035 |
| C17orf71 | 0.196970333 | 6.69E-06 | 2.06E-05 |
| C17orf72 | -0.290643473 | 1.75E-11 | 1.26E-10 |
| C17orf75 | 0.218452448 | 5.56E-07 | 2.04E-06 |
| C17orf76 | -0.130496463 | 0.003007829 | 0.005871715 |
| C17orf79 | 0.181470248 | 3.43E-05 | 9.42E-05 |
| C17orf80 | 0.147956897 | 0.000756957 | 0.001645824 |
| C17orf81 | 0.148448268 | 0.000726486 | 0.001585654 |
| C17orf82 | -0.110994675 | 0.011717428 | 0.020481675 |
| C17orf86 | -0.189581169 | 1.48E-05 | 4.33E-05 |
| C17orf87 | -0.1901986 | 1.39E-05 | 4.07E-05 |
| C17orf88 | -0.122217214 | 0.00548246 | 0.010229012 |
| C17orf89 | 0.14529634 | 0.000943511 | 0.002014409 |
| C17orf91 | -0.188984682 | 1.58E-05 | 4.59E-05 |
| C17orf93 | 0.12897649 | 0.003366939 | 0.006515479 |
| C17orf96 | 0.248231534 | 1.14E-08 | 5.38E-08 |
| C17orf97 | -0.131449259 | 0.00280086 | 0.005497751 |
| C17orf99 | -0.095158545 | 0.030838895 | 0.04935606 |
| C18orf16 | -0.306753625 | 1.11E-12 | 9.63E-12 |
| C18orf18 | -0.157851255 | 0.000323048 | 0.000749604 |
| C18orf19 | 0.337900373 | 3.21E-15 | 4.12E-14 |
| C18orf1 | -0.389438851 | 4.26E-20 | 1.13E-18 |
| C18orf21 | 0.158375976 | 0.000308344 | 0.000718571 |
| C18orf2 | 0.169083197 | 0.000115456 | 0.00029072 |
| C18orf32 | 0.159421192 | 0.000280898 | 0.000660384 |
| C18orf34 | -0.099254959 | 0.024287857 | 0.039743404 |
| C18orf54 | 0.318817333 | 1.25E-13 | 1.25E-12 |
| C18orf55 | 0.239053149 | 3.98E-08 | 1.74E-07 |
| C18orf56 | 0.245983982 | 1.55E-08 | 7.21E-08 |
| C18orf62 | 0.110828776 | 0.011844244 | 0.020674417 |
| C19orf10 | 0.248470675 | 1.10E-08 | 5.22E-08 |
| C19orf18 | -0.200015463 | 4.78E-06 | 1.50E-05 |
| C19orf21 | 0.168047448 | 0.000127303 | 0.000318544 |
| C19orf25 | -0.103233978 | 0.019112828 | 0.031979508 |
| C19orf26 | 0.115777786 | 0.008541478 | 0.015340737 |
| C19orf28 | 0.173234499 | 7.76E-05 | 0.000200667 |
| C19orf29 | -0.129717345 | 0.003187306 | 0.006194251 |
| C19orf2 | 0.130730533 | 0.002955734 | 0.005780744 |
| C19orf34 | -0.183402197 | 2.82E-05 | 7.84E-05 |
| C19orf35 | -0.242143445 | 2.62E-08 | 1.18E-07 |
| C19orf36 | -0.260953985 | 1.83E-09 | 9.74E-09 |
| C19orf38 | -0.096525017 | 0.028502843 | 0.045978115 |
| C19orf39 | -0.134870773 | 0.002160068 | 0.004331404 |
| C19orf40 | 0.301925595 | 2.58E-12 | 2.10E-11 |
| C19orf44 | -0.270673172 | 4.25E-10 | 2.50E-09 |
| C19orf45 | 0.165858655 | 0.000156197 | 0.000384916 |
| C19orf46 | -0.144958177 | 0.00097005 | 0.002067757 |
| C19orf47 | 0.138396189 | 0.001642566 | 0.003359077 |
| C19orf48 | 0.391663407 | 2.50E-20 | 6.89E-19 |
| C19orf51 | -0.148135175 | 0.000745768 | 0.001624505 |
| C19orf52 | 0.11791272 | 0.007390555 | 0.013454997 |
| C19orf53 | 0.132463728 | 0.00259482 | 0.005124519 |
| C19orf59 | -0.179426207 | 4.22E-05 | 0.000113941 |
| C19orf66 | -0.334661704 | 6.08E-15 | 7.46E-14 |
| C19orf69 | -0.138827647 | 0.001587733 | 0.0032546 |
| C19orf6 | -0.215796051 | 7.67E-07 | 2.74E-06 |
| C19orf71 | -0.207733708 | 1.99E-06 | 6.64E-06 |
| C19orf76 | -0.147853879 | 0.000763494 | 0.001658592 |
| C19orf77 | 0.127334845 | 0.003798155 | 0.007276829 |
| C1D | 0.204462899 | 2.89E-06 | 9.42E-06 |
| C1GALT1C1 | 0.189110944 | 1.56E-05 | 4.54E-05 |
| C1QA | -0.105181023 | 0.016951235 | 0.028662658 |
| C1QBP | 0.405591517 | 8.19E-22 | 2.81E-20 |
| C1QL2 | -0.221669054 | 3.74E-07 | 1.41E-06 |
| C1QL4 | 0.179246303 | 4.29E-05 | 0.000115823 |
| C1QTNF2 | -0.323695224 | 5.01E-14 | 5.31E-13 |
| C1QTNF3 | -0.188800063 | 1.61E-05 | 4.67E-05 |
| C1QTNF4 | -0.189635851 | 1.47E-05 | 4.31E-05 |
| C1QTNF6 | 0.224495341 | 2.63E-07 | 1.01E-06 |
| C1QTNF7 | -0.48464741 | 1.08E-31 | 1.64E-29 |
| C1QTNF8 | -0.143955385 | 0.001052855 | 0.002229286 |
| C1QTNF9B | -0.209880986 | 1.55E-06 | 5.26E-06 |
| C1QTNF9 | -0.156660762 | 0.000358869 | 0.000825441 |
| C1orf101 | -0.417544324 | 3.81E-23 | 1.60E-21 |
| C1orf103 | 0.271500063 | 3.74E-10 | 2.21E-09 |
| C1orf104 | -0.234575079 | 7.21E-08 | 3.03E-07 |
| C1orf105 | 0.193389218 | 9.88E-06 | 2.96E-05 |
| C1orf107 | 0.171663241 | 9.03E-05 | 0.000231207 |
| C1orf109 | 0.199610446 | 5.00E-06 | 1.57E-05 |
| C1orf110 | -0.158371074 | 0.000308479 | 0.000718801 |
| C1orf111 | -0.14656426 | 0.000849864 | 0.00182959 |
| C1orf112 | 0.384820383 | 1.27E-19 | 3.16E-18 |
| C1orf113 | -0.180571924 | 3.76E-05 | 0.000102439 |
| C1orf115 | -0.140297933 | 0.00141325 | 0.00292544 |
| C1orf116 | -0.416156496 | 5.48E-23 | 2.24E-21 |
| C1orf122 | 0.118010007 | 0.00734158 | 0.013374362 |
| C1orf124 | 0.284950174 | 4.46E-11 | 3.02E-10 |
| C1orf126 | -0.249270832 | 9.82E-09 | 4.71E-08 |
| C1orf127 | -0.321523236 | 7.53E-14 | 7.78E-13 |
| C1orf128 | -0.145992238 | 0.000890995 | 0.001910653 |
| C1orf129 | -0.230248787 | 1.27E-07 | 5.14E-07 |
| C1orf130 | -0.308355526 | 8.32E-13 | 7.38E-12 |
| C1orf131 | 0.254809243 | 4.48E-09 | 2.24E-08 |
| C1orf133 | -0.34203813 | 1.40E-15 | 1.90E-14 |
| C1orf135 | 0.493301803 | 6.14E-33 | 1.15E-30 |
| C1orf141 | -0.203521287 | 3.22E-06 | 1.04E-05 |
| C1orf144 | 0.113744991 | 0.009783141 | 0.017408122 |
| C1orf146 | -0.106303522 | 0.015804867 | 0.026897205 |
| C1orf150 | -0.288003849 | 2.71E-11 | 1.89E-10 |
| C1orf151 | 0.12501397 | 0.0044933 | 0.008508961 |
| C1orf152 | -0.381441058 | 2.79E-19 | 6.63E-18 |
| C1orf158 | -0.242227811 | 2.59E-08 | 1.17E-07 |
| C1orf162 | -0.236544236 | 5.56E-08 | 2.38E-07 |
| C1orf163 | 0.442947855 | 3.69E-26 | 2.43E-24 |
| C1orf168 | -0.251287358 | 7.39E-09 | 3.61E-08 |
| C1orf170 | -0.122753961 | 0.005278649 | 0.009875481 |
| C1orf173 | -0.252122673 | 6.57E-09 | 3.22E-08 |
| C1orf174 | 0.18009337 | 3.94E-05 | 0.000107085 |
| C1orf175 | -0.421333187 | 1.40E-23 | 6.30E-22 |
| C1orf180 | 0.102858765 | 0.019555939 | 0.032658042 |
| C1orf182 | 0.258353203 | 2.68E-09 | 1.39E-08 |
| C1orf183 | -0.343045157 | 1.14E-15 | 1.57E-14 |
| C1orf186 | -0.413077056 | 1.22E-22 | 4.71E-21 |
| C1orf189 | -0.189331351 | 1.52E-05 | 4.44E-05 |
| C1orf190 | -0.3149909 | 2.52E-13 | 2.41E-12 |
| C1orf192 | -0.216894698 | 6.71E-07 | 2.42E-06 |
| C1orf194 | -0.203306321 | 3.30E-06 | 1.06E-05 |
| C1orf198 | -0.303909271 | 1.82E-12 | 1.53E-11 |
| C1orf200 | -0.172361449 | 8.44E-05 | 0.000216945 |
| C1orf201 | -0.21499607 | 8.44E-07 | 3.00E-06 |
| C1orf203 | -0.188006054 | 1.75E-05 | 5.06E-05 |
| C1orf204 | -0.319322964 | 1.13E-13 | 1.14E-12 |
| C1orf210 | -0.209167036 | 1.68E-06 | 5.69E-06 |
| C1orf212 | 0.181622559 | 3.38E-05 | 9.28E-05 |
| C1orf213 | -0.263637769 | 1.23E-09 | 6.73E-09 |
| C1orf21 | -0.415275669 | 6.89E-23 | 2.77E-21 |
| C1orf223 | -0.150192264 | 0.000627274 | 0.001382812 |
| C1orf226 | -0.110251388 | 0.012295123 | 0.02140163 |
| C1orf228 | -0.275180768 | 2.12E-10 | 1.30E-09 |
| C1orf229 | -0.147491148 | 0.000786927 | 0.001704866 |
| C1orf230 | -0.176067059 | 5.89E-05 | 0.000155061 |
| C1orf27 | 0.099983486 | 0.023258504 | 0.038237455 |
| C1orf31 | 0.398904433 | 4.32E-21 | 1.33E-19 |
| C1orf38 | -0.148008951 | 0.000753674 | 0.001639756 |
| C1orf43 | 0.260677155 | 1.91E-09 | 1.01E-08 |
| C1orf55 | 0.166437334 | 0.000148011 | 0.000366506 |
| C1orf57 | 0.116325026 | 0.008232167 | 0.014849251 |
| C1orf58 | 0.126187353 | 0.004128649 | 0.007863869 |
| C1orf61 | 0.218725212 | 5.37E-07 | 1.97E-06 |
| C1orf63 | -0.335895969 | 4.77E-15 | 5.94E-14 |
| C1orf66 | -0.10775759 | 0.014421302 | 0.024768258 |
| C1orf69 | -0.131831114 | 0.002721617 | 0.005357464 |
| C1orf70 | -0.219588068 | 4.84E-07 | 1.79E-06 |
| C1orf74 | 0.292169512 | 1.36E-11 | 9.97E-11 |
| C1orf85 | 0.172805007 | 8.09E-05 | 0.000208475 |
| C1orf87 | -0.231265513 | 1.11E-07 | 4.55E-07 |
| C1orf88 | -0.335886191 | 4.78E-15 | 5.95E-14 |
| C1orf89 | -0.23817848 | 4.48E-08 | 1.94E-07 |
| C1orf91 | -0.108827572 | 0.013472355 | 0.023264401 |
| C1orf92 | -0.261143607 | 1.78E-09 | 9.49E-09 |
| C1orf93 | -0.157765909 | 0.000325501 | 0.000755032 |
| C1orf96 | 0.140696363 | 0.001369102 | 0.002841409 |
| C1orf9 | 0.140504815 | 0.001390166 | 0.002881236 |
| C20orf103 | -0.202125338 | 3.77E-06 | 1.21E-05 |
| C20orf106 | -0.126486589 | 0.004040046 | 0.007708328 |
| C20orf107 | -0.177555173 | 5.08E-05 | 0.000135534 |
| C20orf108 | -0.257475375 | 3.05E-09 | 1.56E-08 |
| C20orf111 | 0.221886812 | 3.64E-07 | 1.37E-06 |
| C20orf112 | -0.159038153 | 0.00029068 | 0.000680898 |
| C20orf114 | -0.104993778 | 0.017149382 | 0.0289634 |
| C20orf11 | 0.103938514 | 0.018304402 | 0.030750411 |
| C20orf123 | -0.145963277 | 0.000893125 | 0.001914401 |
| C20orf12 | -0.277662874 | 1.43E-10 | 9.02E-10 |
| C20orf132 | -0.33049148 | 1.37E-14 | 1.58E-13 |
| C20orf134 | -0.126166377 | 0.004134926 | 0.007875073 |
| C20orf135 | -0.177631558 | 5.04E-05 | 0.00013458 |
| C20orf141 | 0.302644616 | 2.27E-12 | 1.87E-11 |
| C20orf151 | -0.192887523 | 1.04E-05 | 3.11E-05 |
| C20orf160 | -0.32473946 | 4.11E-14 | 4.43E-13 |
| C20orf165 | -0.118882291 | 0.006915273 | 0.012648469 |
| C20orf191 | 0.112137214 | 0.010876199 | 0.019165429 |
| C20orf194 | -0.435098136 | 3.36E-25 | 1.94E-23 |
| C20orf195 | -0.13463809 | 0.002198981 | 0.00440325 |
| C20orf197 | -0.125184477 | 0.004438566 | 0.008410091 |
| C20orf199 | 0.255900186 | 3.83E-09 | 1.94E-08 |
| C20orf200 | -0.32309324 | 5.61E-14 | 5.88E-13 |
| C20orf201 | -0.102425603 | 0.020078587 | 0.033447021 |
| C20orf202 | -0.102717043 | 0.019725619 | 0.03292091 |
| C20orf203 | -0.119829107 | 0.006477756 | 0.011911443 |
| C20orf20 | 0.323384943 | 5.31E-14 | 5.60E-13 |
| C20orf24 | 0.425514879 | 4.60E-24 | 2.23E-22 |
| C20orf26 | -0.200701266 | 4.42E-06 | 1.40E-05 |
| C20orf27 | 0.113151923 | 0.010174428 | 0.018036937 |
| C20orf30 | 0.153717817 | 0.000463927 | 0.001046375 |
| C20orf46 | -0.116934679 | 0.007899381 | 0.014300592 |
| C20orf56 | -0.319117321 | 1.18E-13 | 1.18E-12 |
| C20orf70 | 0.108778744 | 0.013514432 | 0.023331013 |
| C20orf72 | 0.16270531 | 0.00020879 | 0.000503778 |
| C20orf7 | 0.216854808 | 6.75E-07 | 2.43E-06 |
| C20orf85 | -0.202095368 | 3.78E-06 | 1.21E-05 |
| C20orf96 | -0.197292753 | 6.46E-06 | 1.99E-05 |
| C21orf121 | -0.135298901 | 0.002090109 | 0.004203773 |
| C21orf122 | -0.22638156 | 2.07E-07 | 8.13E-07 |
| C21orf125 | 0.190792842 | 1.30E-05 | 3.84E-05 |
| C21orf128 | -0.118616854 | 0.00704259 | 0.012864859 |
| C21orf129 | -0.183686139 | 2.74E-05 | 7.64E-05 |
| C21orf130 | -0.1111391 | 0.011608006 | 0.020317061 |
| C21orf15 | -0.284247899 | 5.00E-11 | 3.35E-10 |
| C21orf29 | -0.263809957 | 1.20E-09 | 6.58E-09 |
| C21orf2 | -0.48093502 | 3.62E-31 | 4.92E-29 |
| C21orf33 | -0.138496135 | 0.001629712 | 0.003335178 |
| C21orf34 | -0.355289505 | 9.09E-17 | 1.45E-15 |
| C21orf45 | 0.37708128 | 7.59E-19 | 1.68E-17 |
| C21orf49 | -0.269397195 | 5.17E-10 | 2.99E-09 |
| C21orf58 | -0.192562028 | 1.08E-05 | 3.22E-05 |
| C21orf62 | -0.287134452 | 3.12E-11 | 2.17E-10 |
| C21orf63 | -0.273447064 | 2.77E-10 | 1.67E-09 |
| C21orf67 | -0.215476613 | 7.97E-07 | 2.84E-06 |
| C21orf70 | 0.09881161 | 0.024933357 | 0.040713009 |
| C21orf71 | -0.166855679 | 0.000142346 | 0.000353266 |
| C21orf81 | -0.195697813 | 7.69E-06 | 2.34E-05 |
| C21orf82 | -0.178673295 | 4.55E-05 | 0.000122234 |
| C21orf84 | 0.146401895 | 0.000861356 | 0.001852463 |
| C21orf90 | -0.1769668 | 5.39E-05 | 0.000142766 |
| C21orf91 | -0.097588635 | 0.026791285 | 0.043491539 |
| C21orf96 | -0.171486104 | 9.18E-05 | 0.000234988 |
| C22orf13 | -0.220958085 | 4.09E-07 | 1.53E-06 |
| C22orf15 | -0.236815105 | 5.37E-08 | 2.30E-07 |
| C22orf23 | -0.168240755 | 0.000125009 | 0.000313195 |
| C22orf25 | -0.216134272 | 7.36E-07 | 2.64E-06 |
| C22orf26 | -0.1251168 | 0.004460218 | 0.008448716 |
| C22orf28 | 0.137667675 | 0.001739111 | 0.003542016 |
| C22orf29 | -0.265914688 | 8.75E-10 | 4.91E-09 |
| C22orf31 | -0.125616354 | 0.004302614 | 0.008171083 |
| C22orf32 | -0.132655492 | 0.002557461 | 0.005058737 |
| C22orf33 | -0.114793519 | 0.00912397 | 0.016309239 |
| C22orf34 | -0.165653226 | 0.000159203 | 0.000391746 |
| C22orf36 | -0.383683525 | 1.65E-19 | 4.06E-18 |
| C22orf39 | -0.16104509 | 0.000242745 | 0.000577274 |
| C22orf40 | -0.182487491 | 3.10E-05 | 8.56E-05 |
| C22orf43 | -0.153616378 | 0.000468013 | 0.001054164 |
| C22orf45 | -0.270555791 | 4.33E-10 | 2.54E-09 |
| C22orf46 | -0.234909226 | 6.90E-08 | 2.91E-07 |
| C2CD2L | -0.293674914 | 1.05E-11 | 7.86E-11 |
| C2CD2 | -0.255852705 | 3.85E-09 | 1.95E-08 |
| C2CD4A | 0.127021174 | 0.00388603 | 0.007435412 |
| C2CD4B | -0.142410787 | 0.001193232 | 0.002502964 |
| C2CD4C | -0.190723667 | 1.31E-05 | 3.87E-05 |
| C2CD4D | 0.105549637 | 0.016567006 | 0.02807234 |
| C2orf24 | -0.216851093 | 6.75E-07 | 2.43E-06 |
| C2orf28 | 0.129714142 | 0.003188063 | 0.00619512 |
| C2orf29 | 0.222718097 | 3.29E-07 | 1.25E-06 |
| C2orf34 | -0.182461765 | 3.10E-05 | 8.58E-05 |
| C2orf39 | -0.187537732 | 1.84E-05 | 5.29E-05 |
| C2orf3 | 0.255942031 | 3.80E-09 | 1.92E-08 |
| C2orf40 | -0.386785757 | 7.99E-20 | 2.03E-18 |
| C2orf42 | -0.144638416 | 0.000995777 | 0.00211853 |
| C2orf43 | 0.232922155 | 8.96E-08 | 3.72E-07 |
| C2orf44 | 0.167470332 | 0.00013439 | 0.000334603 |
| C2orf47 | 0.355828885 | 8.11E-17 | 1.32E-15 |
| C2orf49 | 0.407294476 | 5.33E-22 | 1.87E-20 |
| C2orf50 | -0.137170157 | 0.001808003 | 0.003672225 |
| C2orf51 | 0.110849883 | 0.011828042 | 0.020649743 |
| C2orf54 | -0.277665002 | 1.43E-10 | 9.02E-10 |
| C2orf55 | -0.285469568 | 4.10E-11 | 2.79E-10 |
| C2orf57 | -0.153195892 | 0.000485312 | 0.001090425 |
| C2orf58 | -0.391430967 | 2.65E-20 | 7.25E-19 |
| C2orf62 | -0.22304316 | 3.16E-07 | 1.20E-06 |
| C2orf63 | -0.342716304 | 1.22E-15 | 1.67E-14 |
| C2orf65 | -0.171574058 | 9.11E-05 | 0.000233104 |
| C2orf66 | -0.208885548 | 1.74E-06 | 5.87E-06 |
| C2orf67 | -0.222109002 | 3.54E-07 | 1.34E-06 |
| C2orf68 | -0.159845994 | 0.00027041 | 0.000637601 |
| C2orf69 | 0.278326699 | 1.29E-10 | 8.17E-10 |
| C2orf71 | -0.20495441 | 2.74E-06 | 8.93E-06 |
| C2orf73 | -0.272467939 | 3.22E-10 | 1.92E-09 |
| C2orf74 | -0.153133702 | 0.000487921 | 0.001095793 |
| C2orf76 | 0.190478814 | 1.35E-05 | 3.96E-05 |
| C2orf77 | -0.201157536 | 4.20E-06 | 1.33E-05 |
| C2orf78 | 0.156608136 | 0.000360535 | 0.000828796 |
| C2orf7 | 0.096539407 | 0.028479074 | 0.045950899 |
| C2orf80 | 0.1003865 | 0.022705466 | 0.037398999 |
| C2orf81 | -0.232991304 | 8.88E-08 | 3.69E-07 |
| C2orf82 | 0.166372044 | 0.000148914 | 0.000368424 |
| C2orf83 | 0.096975499 | 0.027766796 | 0.044906766 |
| C2orf85 | -0.250949754 | 7.75E-09 | 3.77E-08 |
| C2orf86 | -0.207268992 | 2.10E-06 | 6.97E-06 |
| C2orf89 | -0.195178016 | 8.14E-06 | 2.47E-05 |
| C2 | -0.129510304 | 0.003236609 | 0.006282736 |
| C3AR1 | -0.112546685 | 0.010588006 | 0.018697645 |
| C3P1 | 0.176408652 | 5.69E-05 | 0.000150211 |
| C3orf15 | -0.345229249 | 7.34E-16 | 1.03E-14 |
| C3orf16 | -0.146019729 | 0.000888977 | 0.001906735 |
| C3orf17 | 0.13732376 | 0.001786471 | 0.003630705 |
| C3orf18 | -0.279923753 | 1.00E-10 | 6.45E-10 |
| C3orf19 | -0.154792266 | 0.000422634 | 0.000960393 |
| C3orf1 | 0.202891751 | 3.46E-06 | 1.11E-05 |
| C3orf21 | 0.14147509 | 0.001286456 | 0.0026841 |
| C3orf26 | 0.371239148 | 2.84E-18 | 5.74E-17 |
| C3orf32 | -0.12387728 | 0.00487404 | 0.009176932 |
| C3orf33 | 0.111049301 | 0.011675934 | 0.020421646 |
| C3orf35 | -0.235007307 | 6.82E-08 | 2.88E-07 |
| C3orf36 | -0.208296082 | 1.86E-06 | 6.26E-06 |
| C3orf37 | 0.141423197 | 0.001291817 | 0.00269388 |
| C3orf38 | 0.191760686 | 1.18E-05 | 3.49E-05 |
| C3orf42 | -0.353744032 | 1.26E-16 | 1.97E-15 |
| C3orf47 | -0.336092521 | 4.59E-15 | 5.73E-14 |
| C3orf48 | -0.188986011 | 1.58E-05 | 4.59E-05 |
| C3orf50 | -0.159765926 | 0.000272358 | 0.000641515 |
| C3orf52 | -0.161351703 | 0.000236109 | 0.0005631 |
| C3orf54 | -0.229072331 | 1.47E-07 | 5.90E-07 |
| C3orf62 | -0.42606266 | 3.97E-24 | 1.95E-22 |
| C3orf64 | -0.099936169 | 0.023324193 | 0.038329686 |
| C3orf67 | 0.117814212 | 0.007440442 | 0.013533493 |
| C3orf70 | -0.121344435 | 0.005829022 | 0.010809025 |
| C3orf71 | -0.216049989 | 7.44E-07 | 2.66E-06 |
| C3orf75 | 0.099195348 | 0.024373799 | 0.039867716 |
| C3 | -0.224908054 | 2.50E-07 | 9.66E-07 |
| C4A | -0.414828444 | 7.74E-23 | 3.08E-21 |
| C4BPA | -0.293778936 | 1.04E-11 | 7.73E-11 |
| C4orf10 | -0.181040944 | 3.59E-05 | 9.81E-05 |
| C4orf12 | -0.234071787 | 7.71E-08 | 3.22E-07 |
| C4orf14 | 0.158189694 | 0.000313491 | 0.00072929 |
| C4orf19 | -0.231646242 | 1.06E-07 | 4.34E-07 |
| C4orf22 | -0.158953223 | 0.000292891 | 0.000685436 |
| C4orf23 | -0.261041762 | 1.81E-09 | 9.63E-09 |
| C4orf26 | 0.21398742 | 9.52E-07 | 3.36E-06 |
| C4orf27 | 0.123643611 | 0.004955825 | 0.009317752 |
| C4orf31 | -0.334050808 | 6.85E-15 | 8.31E-14 |
| C4orf34 | 0.109542247 | 0.012869584 | 0.022314126 |
| C4orf37 | -0.19158632 | 1.20E-05 | 3.55E-05 |
| C4orf38 | -0.106928178 | 0.015196842 | 0.02597302 |
| C4orf39 | -0.195519947 | 7.84E-06 | 2.38E-05 |
| C4orf43 | 0.403292919 | 1.46E-21 | 4.87E-20 |
| C4orf44 | -0.239296134 | 3.86E-08 | 1.69E-07 |
| C4orf46 | 0.346670137 | 5.47E-16 | 7.88E-15 |
| C4orf47 | -0.096963867 | 0.027785595 | 0.044929898 |
| C4orf52 | 0.211791185 | 1.24E-06 | 4.27E-06 |
| C4orf6 | 0.143965063 | 0.001052026 | 0.002228002 |
| C4orf7 | -0.212708604 | 1.11E-06 | 3.86E-06 |
| C5AR1 | -0.127145792 | 0.0038509 | 0.007370659 |
| C5orf15 | 0.175728224 | 6.09E-05 | 0.00015993 |
| C5orf20 | -0.292324139 | 1.32E-11 | 9.73E-11 |
| C5orf22 | 0.180903366 | 3.64E-05 | 9.94E-05 |
| C5orf23 | -0.151810521 | 0.000546603 | 0.001216785 |
| C5orf24 | 0.100883281 | 0.022039463 | 0.036413025 |
| C5orf25 | -0.158494397 | 0.000305113 | 0.000711623 |
| C5orf28 | 0.218198887 | 5.73E-07 | 2.10E-06 |
| C5orf30 | 0.196255035 | 7.24E-06 | 2.21E-05 |
| C5orf32 | -0.151443847 | 0.000563989 | 0.001252696 |
| C5orf33 | 0.161792491 | 0.000226865 | 0.000543259 |
| C5orf34 | 0.290530521 | 1.78E-11 | 1.28E-10 |
| C5orf38 | -0.400723887 | 2.76E-21 | 8.77E-20 |
| C5orf39 | -0.169935667 | 0.000106491 | 0.00027012 |
| C5orf41 | -0.338384824 | 2.91E-15 | 3.77E-14 |
| C5orf42 | -0.246100281 | 1.53E-08 | 7.10E-08 |
| C5orf43 | 0.291563271 | 1.50E-11 | 1.10E-10 |
| C5orf45 | -0.354120926 | 1.16E-16 | 1.83E-15 |
| C5orf46 | 0.325803271 | 3.36E-14 | 3.67E-13 |
| C5orf49 | -0.30110248 | 2.97E-12 | 2.40E-11 |
| C5orf4 | -0.159223114 | 0.000285917 | 0.000671158 |
| C5orf53 | -0.412646826 | 1.36E-22 | 5.18E-21 |
| C5orf56 | -0.287359587 | 3.01E-11 | 2.09E-10 |
| C5orf58 | -0.196330253 | 7.18E-06 | 2.19E-05 |
| C6orf103 | -0.262472302 | 1.46E-09 | 7.91E-09 |
| C6orf106 | -0.117413961 | 0.007646251 | 0.013877934 |
| C6orf115 | 0.230161471 | 1.28E-07 | 5.19E-07 |
| C6orf118 | -0.236971738 | 5.26E-08 | 2.25E-07 |
| C6orf120 | 0.12389989 | 0.004866191 | 0.009166472 |
| C6orf123 | -0.305793723 | 1.31E-12 | 1.12E-11 |
| C6orf124 | -0.332527149 | 9.22E-15 | 1.09E-13 |
| C6orf125 | 0.099958668 | 0.023292938 | 0.038287767 |
| C6orf129 | 0.334362145 | 6.45E-15 | 7.88E-14 |
| C6orf134 | -0.14161688 | 0.001271911 | 0.002655972 |
| C6orf138 | -0.269247364 | 5.29E-10 | 3.06E-09 |
| C6orf145 | -0.205191925 | 2.66E-06 | 8.71E-06 |
| C6orf147 | -0.146056975 | 0.000886249 | 0.001901905 |
| C6orf150 | 0.293471732 | 1.09E-11 | 8.11E-11 |
| C6orf153 | 0.353544977 | 1.31E-16 | 2.05E-15 |
| C6orf155 | -0.34270887 | 1.22E-15 | 1.67E-14 |
| C6orf163 | -0.192991786 | 1.03E-05 | 3.08E-05 |
| C6orf164 | -0.302345436 | 2.39E-12 | 1.96E-11 |
| C6orf165 | -0.151682328 | 0.000552624 | 0.00122964 |
| C6orf167 | 0.197015194 | 6.66E-06 | 2.05E-05 |
| C6orf168 | -0.196007733 | 7.43E-06 | 2.27E-05 |
| C6orf170 | -0.17799986 | 4.86E-05 | 0.000130098 |
| C6orf174 | -0.277786015 | 1.41E-10 | 8.86E-10 |
| C6orf176 | 0.281429795 | 7.87E-11 | 5.15E-10 |
| C6orf182 | 0.220323513 | 4.42E-07 | 1.65E-06 |
| C6orf186 | -0.113318435 | 0.01006319 | 0.017858744 |
| C6orf195 | 0.132649179 | 0.002558683 | 0.005060654 |
| C6orf1 | -0.138615888 | 0.001614432 | 0.003305443 |
| C6orf201 | -0.312292868 | 4.11E-13 | 3.81E-12 |
| C6orf203 | -0.144809888 | 0.000981904 | 0.002090572 |
| C6orf204 | -0.098170694 | 0.025892666 | 0.042152029 |
| C6orf217 | -0.267953672 | 6.43E-10 | 3.68E-09 |
| C6orf218 | 0.225496003 | 2.32E-07 | 9.01E-07 |
| C6orf222 | -0.173555422 | 7.52E-05 | 0.000194902 |
| C6orf225 | -0.168921141 | 0.000117238 | 0.000294948 |
| C6orf226 | -0.124746524 | 0.004580382 | 0.008661559 |
| C6orf227 | -0.138941554 | 0.001573539 | 0.003227492 |
| C6orf25 | -0.174672919 | 6.75E-05 | 0.000176241 |
| C6orf26 | -0.129435343 | 0.003254629 | 0.006314038 |
| C6orf41 | -0.271374823 | 3.82E-10 | 2.25E-09 |
| C6orf47 | -0.190752447 | 1.31E-05 | 3.86E-05 |
| C6orf57 | 0.107829142 | 0.014356057 | 0.024666799 |
| C6orf58 | -0.095437393 | 0.03034931 | 0.04866993 |
| C6orf59 | -0.179576525 | 4.15E-05 | 0.000112344 |
| C6orf70 | -0.282456132 | 6.68E-11 | 4.41E-10 |
| C6orf89 | -0.184314684 | 2.57E-05 | 7.20E-05 |
| C6orf97 | -0.286332551 | 3.56E-11 | 2.45E-10 |
| C6 | -0.287909835 | 2.75E-11 | 1.92E-10 |
| C7orf10 | 0.10898574 | 0.013336851 | 0.023052316 |
| C7orf11 | 0.311699572 | 4.57E-13 | 4.20E-12 |
| C7orf23 | -0.424903588 | 5.42E-24 | 2.59E-22 |
| C7orf25 | 0.174598307 | 6.80E-05 | 0.000177459 |
| C7orf27 | -0.096498037 | 0.028547453 | 0.046042644 |
| C7orf28A | 0.197187136 | 6.53E-06 | 2.01E-05 |
| C7orf28B | 0.228748865 | 1.54E-07 | 6.13E-07 |
| C7orf30 | 0.136438093 | 0.001913929 | 0.003869676 |
| C7orf31 | -0.140190793 | 0.001425343 | 0.00294864 |
| C7orf36 | 0.185794204 | 2.20E-05 | 6.25E-05 |
| C7orf40 | 0.132182661 | 0.002650475 | 0.005229783 |
| C7orf41 | -0.336186408 | 4.50E-15 | 5.64E-14 |
| C7orf44 | 0.223420385 | 3.01E-07 | 1.15E-06 |
| C7orf45 | -0.14592063 | 0.000896271 | 0.001920938 |
| C7orf46 | -0.185473035 | 2.28E-05 | 6.44E-05 |
| C7orf47 | 0.133209607 | 0.002452251 | 0.004865555 |
| C7orf49 | 0.326566918 | 2.91E-14 | 3.20E-13 |
| C7orf4 | -0.117824965 | 0.007434982 | 0.013526023 |
| C7orf53 | -0.118851672 | 0.006929853 | 0.012672818 |
| C7orf54 | -0.176480445 | 5.65E-05 | 0.000149218 |
| C7orf57 | -0.243531386 | 2.17E-08 | 9.87E-08 |
| C7orf58 | -0.248992874 | 1.02E-08 | 4.89E-08 |
| C7orf61 | 0.095478879 | 0.030277045 | 0.048569608 |
| C7orf63 | -0.235360739 | 6.51E-08 | 2.75E-07 |
| C7orf68 | 0.379075467 | 4.81E-19 | 1.10E-17 |
| C7orf70 | 0.267957572 | 6.43E-10 | 3.68E-09 |
| C7 | -0.371056021 | 2.96E-18 | 5.96E-17 |
| C8orf12 | -0.105827644 | 0.016282289 | 0.027627368 |
| C8orf30A | 0.175933141 | 5.96E-05 | 0.000157012 |
| C8orf33 | 0.313165813 | 3.51E-13 | 3.29E-12 |
| C8orf34 | -0.35247172 | 1.64E-16 | 2.54E-15 |
| C8orf38 | 0.165189356 | 0.000166194 | 0.000407793 |
| C8orf41 | 0.132894887 | 0.002511512 | 0.004974248 |
| C8orf42 | -0.252813542 | 5.95E-09 | 2.94E-08 |
| C8orf46 | -0.183851291 | 2.69E-05 | 7.52E-05 |
| C8orf48 | -0.225141553 | 2.43E-07 | 9.40E-07 |
| C8orf58 | -0.118548289 | 0.007075816 | 0.012920831 |
| C8orf59 | 0.339823915 | 2.19E-15 | 2.88E-14 |
| C8orf73 | -0.146895216 | 0.000826876 | 0.001784645 |
| C8orf74 | 0.123551022 | 0.004988572 | 0.009374031 |
| C8orf75 | 0.198773659 | 5.48E-06 | 1.71E-05 |
| C8orf76 | 0.486718267 | 5.49E-32 | 8.57E-30 |
| C8orf77 | -0.158229987 | 0.000312371 | 0.000726854 |
| C8orf79 | -0.332818302 | 8.72E-15 | 1.04E-13 |
| C8orf83 | 0.125360047 | 0.004382836 | 0.008313165 |
| C8orf84 | -0.237752765 | 4.74E-08 | 2.04E-07 |
| C8orf85 | -0.324801692 | 4.06E-14 | 4.38E-13 |
| C9orf100 | 0.378226718 | 5.84E-19 | 1.32E-17 |
| C9orf103 | -0.201241194 | 4.17E-06 | 1.32E-05 |
| C9orf106 | -0.22151115 | 3.82E-07 | 1.43E-06 |
| C9orf117 | -0.261381866 | 1.72E-09 | 9.19E-09 |
| C9orf119 | 0.154761348 | 0.000423773 | 0.000962871 |
| C9orf11 | -0.178850618 | 4.47E-05 | 0.00012022 |
| C9orf128 | -0.36668537 | 7.79E-18 | 1.48E-16 |
| C9orf130 | -0.209106763 | 1.69E-06 | 5.73E-06 |
| C9orf131 | -0.262631802 | 1.43E-09 | 7.74E-09 |
| C9orf135 | -0.213303714 | 1.03E-06 | 3.62E-06 |
| C9orf139 | -0.338057809 | 3.11E-15 | 4.01E-14 |
| C9orf140 | 0.445414056 | 1.82E-26 | 1.25E-24 |
| C9orf144B | -0.216960352 | 6.66E-07 | 2.41E-06 |
| C9orf144 | -0.154831434 | 0.000421196 | 0.000957232 |
| C9orf150 | -0.17363401 | 7.47E-05 | 0.000193597 |
| C9orf152 | -0.23937386 | 3.82E-08 | 1.67E-07 |
| C9orf156 | -0.167997158 | 0.000127906 | 0.000320014 |
| C9orf163 | -0.13526396 | 0.00209574 | 0.004213402 |
| C9orf167 | -0.106877355 | 0.015245532 | 0.026040651 |
| C9orf171 | -0.214274647 | 9.20E-07 | 3.25E-06 |
| C9orf173 | -0.175414045 | 6.28E-05 | 0.000164674 |
| C9orf21 | 0.10894744 | 0.013369553 | 0.02310657 |
| C9orf23 | 0.21037218 | 1.46E-06 | 4.98E-06 |
| C9orf24 | -0.238849466 | 4.09E-08 | 1.78E-07 |
| C9orf27 | 0.095052355 | 0.031027107 | 0.049629466 |
| C9orf30 | 0.463365276 | 8.98E-29 | 8.20E-27 |
| C9orf37 | -0.113724612 | 0.009796362 | 0.017430095 |
| C9orf40 | 0.35060697 | 2.43E-16 | 3.64E-15 |
| C9orf41 | 0.214856436 | 8.58E-07 | 3.04E-06 |
| C9orf44 | -0.21155523 | 1.27E-06 | 4.38E-06 |
| C9orf45 | -0.405050549 | 9.38E-22 | 3.20E-20 |
| C9orf46 | 0.218515715 | 5.51E-07 | 2.02E-06 |
| C9orf50 | -0.199497099 | 5.06E-06 | 1.59E-05 |
| C9orf5 | -0.166329634 | 0.000149503 | 0.000369744 |
| C9orf64 | 0.107034144 | 0.01509576 | 0.025826761 |
| C9orf66 | -0.285673453 | 3.97E-11 | 2.71E-10 |
| C9orf68 | -0.337477775 | 3.49E-15 | 4.45E-14 |
| C9orf69 | 0.137917258 | 0.001705464 | 0.003479872 |
| C9orf6 | 0.098714921 | 0.025076083 | 0.040929344 |
| C9orf70 | -0.170799361 | 9.81E-05 | 0.000249988 |
| C9orf71 | -0.125868259 | 0.004225066 | 0.008032207 |
| C9orf72 | -0.147972942 | 0.000755944 | 0.001644054 |
| C9orf7 | -0.282546004 | 6.58E-11 | 4.35E-10 |
| C9orf80 | 0.155829495 | 0.000386041 | 0.000882658 |
| C9orf82 | 0.165948494 | 0.000154898 | 0.000381906 |
| C9orf85 | 0.173321918 | 7.70E-05 | 0.00019913 |
| C9orf91 | -0.144331722 | 0.001021043 | 0.002169975 |
| C9orf93 | -0.149124713 | 0.000686395 | 0.001504915 |
| C9orf95 | -0.121454296 | 0.005784341 | 0.010734138 |
| C9orf96 | -0.297234023 | 5.77E-12 | 4.47E-11 |
| C9orf98 | -0.234092485 | 7.69E-08 | 3.22E-07 |
| C9orf9 | -0.247689556 | 1.22E-08 | 5.78E-08 |
| C9 | 0.111856883 | 0.011077489 | 0.019479822 |
| CA10 | -0.334949509 | 5.74E-15 | 7.08E-14 |
| CA11 | -0.131668537 | 0.002755102 | 0.00541751 |
| CA12 | 0.178975595 | 4.41E-05 | 0.000118809 |
| CA13 | -0.265400065 | 9.46E-10 | 5.28E-09 |
| CA1 | -0.18768005 | 1.81E-05 | 5.22E-05 |
| CA2 | -0.111999122 | 0.010974946 | 0.019313895 |
| CA3 | -0.411136563 | 2.00E-22 | 7.40E-21 |
| CA4 | -0.232283076 | 9.73E-08 | 4.02E-07 |
| CA5A | 0.167697431 | 0.000131558 | 0.00032837 |
| CA5BP | -0.099493649 | 0.023946351 | 0.03922633 |
| CA5B | -0.337788606 | 3.28E-15 | 4.20E-14 |
| CA6 | 0.098413288 | 0.025525878 | 0.041602352 |
| CAB39L | -0.198557577 | 5.62E-06 | 1.75E-05 |
| CABC1 | -0.277957608 | 1.37E-10 | 8.63E-10 |
| CABIN1 | -0.358663176 | 4.44E-17 | 7.49E-16 |
| CABLES1 | -0.267492483 | 6.90E-10 | 3.93E-09 |
| CABP7 | -0.142848315 | 0.001151812 | 0.002423668 |
| CABYR | 0.264078871 | 1.15E-09 | 6.34E-09 |
| CACHD1 | -0.356332074 | 7.29E-17 | 1.19E-15 |
| CACNA1C | -0.313194753 | 3.49E-13 | 3.27E-12 |
| CACNA1D | -0.298839181 | 4.39E-12 | 3.44E-11 |
| CACNA1F | -0.381311274 | 2.87E-19 | 6.81E-18 |
| CACNA1G | -0.326907687 | 2.72E-14 | 3.01E-13 |
| CACNA1H | -0.128462109 | 0.003497014 | 0.006744338 |
| CACNA1I | -0.148561261 | 0.000719642 | 0.001571529 |
| CACNA2D2 | -0.387604147 | 6.58E-20 | 1.68E-18 |
| CACNA2D3 | -0.264232869 | 1.13E-09 | 6.21E-09 |
| CACNA2D4 | -0.198634482 | 5.57E-06 | 1.73E-05 |
| CACNB1 | -0.508055468 | 3.79E-35 | 1.12E-32 |
| CACNB3 | -0.177560223 | 5.08E-05 | 0.000135484 |
| CACNB4 | -0.305748215 | 1.32E-12 | 1.13E-11 |
| CACNG4 | -0.169266394 | 0.000113471 | 0.00028618 |
| CACNG6 | -0.156967938 | 0.000349288 | 0.000804884 |
| CACNG7 | 0.164294241 | 0.000180504 | 0.000440365 |
| CACNG8 | 0.133540323 | 0.002391355 | 0.004755587 |
| CACYBP | 0.427230515 | 2.90E-24 | 1.46E-22 |
| CADM1 | -0.187755337 | 1.80E-05 | 5.18E-05 |
| CADM3 | -0.340734102 | 1.82E-15 | 2.43E-14 |
| CADM4 | -0.108441186 | 0.013808491 | 0.023805796 |
| CADPS2 | -0.248548645 | 1.09E-08 | 5.17E-08 |
| CADPS | -0.21185143 | 1.23E-06 | 4.24E-06 |
| CAD | 0.19356552 | 9.69E-06 | 2.91E-05 |
| CAGE1 | 0.166130441 | 0.0001523 | 0.000375918 |
| CALB1 | 0.139672921 | 0.001485148 | 0.003063166 |
| CALCA | 0.174735814 | 6.71E-05 | 0.000175254 |
| CALCB | 0.17658355 | 5.59E-05 | 0.000147839 |
| CALCOCO1 | -0.484380507 | 1.18E-31 | 1.78E-29 |
| CALCOCO2 | -0.208737015 | 1.77E-06 | 5.97E-06 |
| CALCRL | -0.135788011 | 0.002012724 | 0.004058751 |
| CALCR | 0.099820624 | 0.023485278 | 0.038556365 |
| CALM2 | 0.159876143 | 0.000269679 | 0.00063603 |
| CALML3 | 0.125284957 | 0.004406594 | 0.008354263 |
| CALML4 | -0.222022047 | 3.58E-07 | 1.35E-06 |
| CALML6 | -0.222473856 | 3.39E-07 | 1.28E-06 |
| CALR | 0.248729727 | 1.06E-08 | 5.05E-08 |
| CALU | 0.404319852 | 1.13E-21 | 3.80E-20 |
| CALY | 0.121134132 | 0.005915419 | 0.010957473 |
| CAMK1D | -0.312903298 | 3.68E-13 | 3.44E-12 |
| CAMK1G | -0.176643998 | 5.56E-05 | 0.000147096 |
| CAMK1 | -0.240336522 | 3.35E-08 | 1.48E-07 |
| CAMK2A | -0.262070952 | 1.55E-09 | 8.37E-09 |
| CAMK2D | -0.279511277 | 1.07E-10 | 6.85E-10 |
| CAMK2G | -0.175247806 | 6.38E-05 | 0.000167158 |
| CAMK4 | -0.190242386 | 1.38E-05 | 4.06E-05 |
| CAMKK1 | -0.30650442 | 1.16E-12 | 1.00E-11 |
| CAMKK2 | 0.131825026 | 0.002722864 | 0.005358863 |
| CAMP | -0.218790566 | 5.33E-07 | 1.96E-06 |
| CAMTA1 | -0.183647757 | 2.75E-05 | 7.67E-05 |
| CAMTA2 | -0.335246536 | 5.42E-15 | 6.69E-14 |
| CAND1 | 0.249856544 | 9.04E-09 | 4.36E-08 |
| CAND2 | -0.220436094 | 4.36E-07 | 1.63E-06 |
| CANT1 | 0.212682333 | 1.11E-06 | 3.87E-06 |
| CANX | 0.110163077 | 0.012365405 | 0.021510854 |
| CAPG | -0.118982735 | 0.006867635 | 0.01257745 |
| CAPN10 | -0.166106043 | 0.000152646 | 0.000376632 |
| CAPN11 | -0.224114051 | 2.76E-07 | 1.06E-06 |
| CAPN12 | -0.196515924 | 7.03E-06 | 2.15E-05 |
| CAPN13 | -0.216962384 | 6.66E-07 | 2.41E-06 |
| CAPN2 | -0.127265299 | 0.003817482 | 0.007309495 |
| CAPN3 | -0.522188531 | 2.29E-37 | 9.93E-35 |
| CAPN6 | -0.139604208 | 0.001493254 | 0.003078296 |
| CAPN8 | -0.326314929 | 3.05E-14 | 3.35E-13 |
| CAPN9 | -0.118915214 | 0.006899626 | 0.012626783 |
| CAPNS2 | -0.171072985 | 9.55E-05 | 0.000243851 |
| CAPRIN1 | 0.202116753 | 3.77E-06 | 1.21E-05 |
| CAPRIN2 | -0.131755872 | 0.002737068 | 0.005385226 |
| CAPS2 | -0.10068769 | 0.02229963 | 0.036800281 |
| CAPSL | -0.181621175 | 3.38E-05 | 9.28E-05 |
| CAPS | -0.312729457 | 3.80E-13 | 3.54E-12 |
| CAPZA1 | 0.289802418 | 2.01E-11 | 1.44E-10 |
| CAPZA2 | 0.19664349 | 6.93E-06 | 2.13E-05 |
| CARD11 | -0.150569772 | 0.000607524 | 0.001342383 |
| CARD14 | 0.215430568 | 8.01E-07 | 2.85E-06 |
| CARD16 | -0.232194159 | 9.85E-08 | 4.06E-07 |
| CARD18 | 0.099647463 | 0.023728489 | 0.038901332 |
| CARD6 | -0.141555057 | 0.001278234 | 0.002668339 |
| CARD8 | -0.359643708 | 3.60E-17 | 6.18E-16 |
| CARD9 | -0.29533387 | 7.97E-12 | 6.06E-11 |
| CARHSP1 | 0.134881318 | 0.002158319 | 0.004328766 |
| CARM1 | 0.183797159 | 2.71E-05 | 7.56E-05 |
| CARNS1 | -0.256445541 | 3.54E-09 | 1.80E-08 |
| CARS | 0.284377896 | 4.90E-11 | 3.29E-10 |
| CASC1 | -0.302897861 | 2.17E-12 | 1.80E-11 |
| CASC2 | -0.27894902 | 1.17E-10 | 7.45E-10 |
| CASC5 | 0.438963273 | 1.14E-25 | 7.10E-24 |
| CASD1 | -0.179770379 | 4.07E-05 | 0.000110345 |
| CASKIN1 | 0.102360067 | 0.020158712 | 0.033566505 |
| CASKIN2 | -0.299125337 | 4.18E-12 | 3.29E-11 |
| CASP10 | -0.290473157 | 1.80E-11 | 1.30E-10 |
| CASP12 | -0.287088231 | 3.15E-11 | 2.18E-10 |
| CASP14 | 0.117674285 | 0.007511822 | 0.013650904 |
| CASP1 | -0.260420977 | 1.98E-09 | 1.05E-08 |
| CASP3 | 0.248723724 | 1.06E-08 | 5.05E-08 |
| CASP6 | 0.189662046 | 1.47E-05 | 4.30E-05 |
| CASQ1 | -0.380341473 | 3.59E-19 | 8.36E-18 |
| CASQ2 | -0.373317952 | 1.78E-18 | 3.69E-17 |
| CASR | -0.318660668 | 1.28E-13 | 1.28E-12 |
| CASS4 | -0.348818889 | 3.51E-16 | 5.20E-15 |
| CAST | -0.122776361 | 0.005270294 | 0.009862619 |
| CASZ1 | -0.398057676 | 5.31E-21 | 1.61E-19 |
| CATSPER2P1 | 0.13582619 | 0.002006796 | 0.004048429 |
| CATSPER2 | -0.294193387 | 9.67E-12 | 7.26E-11 |
| CATSPERG | -0.28804148 | 2.69E-11 | 1.88E-10 |
| CAT | -0.290991355 | 1.65E-11 | 1.20E-10 |
| CAV3 | -0.36546036 | 1.02E-17 | 1.91E-16 |
| CBFA2T2 | -0.162499231 | 0.000212749 | 0.000512711 |
| CBFA2T3 | -0.394934923 | 1.14E-20 | 3.26E-19 |
| CBLC | 0.141211358 | 0.001313917 | 0.0027354 |
| CBLL1 | 0.184309855 | 2.57E-05 | 7.20E-05 |
| CBLN3 | -0.204594721 | 2.85E-06 | 9.28E-06 |
| CBLN4 | -0.159207837 | 0.000286308 | 0.000671917 |
| CBR1 | 0.117994399 | 0.007349418 | 0.01338742 |
| CBR4 | -0.199926387 | 4.82E-06 | 1.52E-05 |
| CBS | 0.17894096 | 4.43E-05 | 0.000119204 |
| CBWD1 | 0.109099817 | 0.013239867 | 0.022899367 |
| CBWD3 | 0.145167467 | 0.000953545 | 0.002034528 |
| CBWD5 | 0.101037734 | 0.021835881 | 0.036109506 |
| CBX1 | 0.187560743 | 1.83E-05 | 5.28E-05 |
| CBX2 | 0.242689416 | 2.44E-08 | 1.10E-07 |
| CBX3 | 0.408489264 | 3.94E-22 | 1.42E-20 |
| CBX6 | -0.18339461 | 2.82E-05 | 7.85E-05 |
| CBX7 | -0.610103406 | 7.88E-54 | 7.87E-50 |
| CC2D1A | -0.239438398 | 3.78E-08 | 1.66E-07 |
| CC2D1B | -0.257678118 | 2.96E-09 | 1.52E-08 |
| CC2D2A | -0.198226483 | 5.83E-06 | 1.81E-05 |
| CC2D2B | -0.330838636 | 1.28E-14 | 1.48E-13 |
| CCAR1 | 0.253590791 | 5.33E-09 | 2.65E-08 |
| CCDC101 | -0.328767961 | 1.91E-14 | 2.16E-13 |
| CCDC102A | -0.259298516 | 2.34E-09 | 1.22E-08 |
| CCDC106 | -0.160304162 | 0.000259508 | 0.000613853 |
| CCDC108 | -0.229295434 | 1.43E-07 | 5.75E-07 |
| CCDC109A | 0.186654466 | 2.02E-05 | 5.76E-05 |
| CCDC110 | 0.103064828 | 0.019311492 | 0.032282186 |
| CCDC113 | -0.14714258 | 0.000810071 | 0.00175156 |
| CCDC114 | -0.350169837 | 2.66E-16 | 3.97E-15 |
| CCDC115 | -0.318585708 | 1.30E-13 | 1.30E-12 |
| CCDC116 | -0.180631718 | 3.74E-05 | 0.000101862 |
| CCDC11 | -0.185998347 | 2.16E-05 | 6.13E-05 |
| CCDC120 | -0.252182049 | 6.51E-09 | 3.20E-08 |
| CCDC121 | -0.16230119 | 0.000216619 | 0.000520845 |
| CCDC125 | -0.111204518 | 0.011558741 | 0.020245016 |
| CCDC126 | 0.150500484 | 0.000611105 | 0.001349848 |
| CCDC129 | -0.156455617 | 0.000365404 | 0.000838638 |
| CCDC12 | -0.113638398 | 0.009852467 | 0.017523679 |
| CCDC130 | -0.278830717 | 1.19E-10 | 7.58E-10 |
| CCDC132 | 0.120842374 | 0.006037185 | 0.01116396 |
| CCDC135 | -0.212668045 | 1.11E-06 | 3.87E-06 |
| CCDC136 | -0.219470318 | 4.91E-07 | 1.82E-06 |
| CCDC137 | 0.139099136 | 0.001554096 | 0.003192525 |
| CCDC138 | 0.363418826 | 1.59E-17 | 2.87E-16 |
| CCDC13 | -0.294417891 | 9.31E-12 | 7.00E-11 |
| CCDC141 | -0.268974274 | 5.51E-10 | 3.18E-09 |
| CCDC144A | -0.143361043 | 0.001104961 | 0.002332943 |
| CCDC144B | -0.160568101 | 0.000253416 | 0.000600722 |
| CCDC146 | -0.401796112 | 2.11E-21 | 6.88E-20 |
| CCDC147 | -0.278526737 | 1.25E-10 | 7.94E-10 |
| CCDC148 | -0.165534439 | 0.000160967 | 0.000395842 |
| CCDC149 | -0.101331584 | 0.021453064 | 0.035538193 |
| CCDC14 | -0.208697825 | 1.78E-06 | 5.99E-06 |
| CCDC150 | 0.176637302 | 5.57E-05 | 0.000147155 |
| CCDC151 | -0.143304429 | 0.001110047 | 0.002343185 |
| CCDC152 | -0.313489959 | 3.31E-13 | 3.12E-12 |
| CCDC153 | -0.263648437 | 1.23E-09 | 6.72E-09 |
| CCDC154 | -0.205182434 | 2.67E-06 | 8.71E-06 |
| CCDC155 | -0.110279222 | 0.012273044 | 0.021365059 |
| CCDC157 | -0.288201219 | 2.62E-11 | 1.83E-10 |
| CCDC159 | -0.425637018 | 4.45E-24 | 2.16E-22 |
| CCDC17 | -0.273657031 | 2.68E-10 | 1.62E-09 |
| CCDC19 | -0.219080893 | 5.15E-07 | 1.90E-06 |
| CCDC21 | 0.115777823 | 0.008541457 | 0.015340737 |
| CCDC22 | -0.106032572 | 0.016075169 | 0.027300897 |
| CCDC24 | -0.23954103 | 3.73E-08 | 1.64E-07 |
| CCDC28A | -0.299492486 | 3.92E-12 | 3.10E-11 |
| CCDC30 | -0.390160095 | 3.59E-20 | 9.61E-19 |
| CCDC33 | -0.221687252 | 3.73E-07 | 1.40E-06 |
| CCDC34 | 0.378032964 | 6.10E-19 | 1.37E-17 |
| CCDC36 | -0.127650665 | 0.003711501 | 0.00712354 |
| CCDC37 | -0.286942461 | 3.22E-11 | 2.23E-10 |
| CCDC39 | -0.325589495 | 3.50E-14 | 3.81E-13 |
| CCDC3 | -0.133781207 | 0.002347873 | 0.004674231 |
| CCDC40 | -0.2889036 | 2.34E-11 | 1.65E-10 |
| CCDC42B | -0.276278057 | 1.78E-10 | 1.11E-09 |
| CCDC42 | -0.240246137 | 3.39E-08 | 1.50E-07 |
| CCDC43 | 0.303750992 | 1.87E-12 | 1.57E-11 |
| CCDC45 | -0.133631197 | 0.002374866 | 0.004726557 |
| CCDC46 | -0.348423133 | 3.81E-16 | 5.61E-15 |
| CCDC48 | -0.458469761 | 3.95E-28 | 3.25E-26 |
| CCDC51 | 0.287831344 | 2.79E-11 | 1.94E-10 |
| CCDC57 | -0.328785933 | 1.90E-14 | 2.16E-13 |
| CCDC58 | 0.44825644 | 8.02E-27 | 5.75E-25 |
| CCDC59 | 0.412831976 | 1.30E-22 | 4.96E-21 |
| CCDC60 | -0.227468273 | 1.81E-07 | 7.15E-07 |
| CCDC61 | -0.278815271 | 1.19E-10 | 7.60E-10 |
| CCDC64B | -0.397689448 | 5.81E-21 | 1.75E-19 |
| CCDC65 | -0.205551585 | 2.55E-06 | 8.39E-06 |
| CCDC66 | -0.300396217 | 3.36E-12 | 2.69E-11 |
| CCDC68 | -0.165690655 | 0.000158652 | 0.000390485 |
| CCDC69 | -0.286840136 | 3.28E-11 | 2.26E-10 |
| CCDC70 | 0.105659916 | 0.016453549 | 0.027906112 |
| CCDC73 | -0.097432329 | 0.027037127 | 0.043840195 |
| CCDC74A | -0.108131431 | 0.014083272 | 0.024243944 |
| CCDC74B | -0.130169732 | 0.003081945 | 0.006006427 |
| CCDC77 | 0.322960612 | 5.75E-14 | 6.02E-13 |
| CCDC78 | -0.240878769 | 3.12E-08 | 1.38E-07 |
| CCDC81 | -0.289488803 | 2.12E-11 | 1.51E-10 |
| CCDC82 | -0.112590386 | 0.010557651 | 0.018651789 |
| CCDC84 | -0.298754953 | 4.45E-12 | 3.49E-11 |
| CCDC85A | -0.265501383 | 9.31E-10 | 5.21E-09 |
| CCDC85C | 0.165350892 | 0.000163727 | 0.000402185 |
| CCDC86 | 0.381519115 | 2.74E-19 | 6.53E-18 |
| CCDC88B | -0.334358023 | 6.45E-15 | 7.89E-14 |
| CCDC88C | -0.192527196 | 1.08E-05 | 3.23E-05 |
| CCDC89 | -0.226168216 | 2.13E-07 | 8.33E-07 |
| CCDC8 | -0.229795357 | 1.34E-07 | 5.42E-07 |
| CCDC90B | 0.228433252 | 1.60E-07 | 6.36E-07 |
| CCDC91 | 0.174030013 | 7.18E-05 | 0.000186704 |
| CCDC92 | -0.302492347 | 2.33E-12 | 1.92E-11 |
| CCDC93 | -0.260461708 | 1.97E-09 | 1.04E-08 |
| CCDC94 | -0.143675878 | 0.001077071 | 0.002277906 |
| CCDC96 | -0.272643964 | 3.14E-10 | 1.88E-09 |
| CCDC97 | -0.17666694 | 5.55E-05 | 0.000146803 |
| CCDC99 | 0.41829966 | 3.13E-23 | 1.34E-21 |
| CCDC9 | -0.097517287 | 0.026903262 | 0.043655009 |
| CCHCR1 | -0.112954537 | 0.010307704 | 0.01825216 |
| CCL13 | -0.110923388 | 0.011771773 | 0.020562283 |
| CCL14 | -0.311957228 | 4.36E-13 | 4.03E-12 |
| CCL16 | -0.318238977 | 1.39E-13 | 1.38E-12 |
| CCL17 | -0.290549115 | 1.78E-11 | 1.28E-10 |
| CCL19 | -0.245178141 | 1.73E-08 | 8.00E-08 |
| CCL20 | 0.217753785 | 6.05E-07 | 2.20E-06 |
| CCL22 | -0.179513825 | 4.18E-05 | 0.000112991 |
| CCL23 | -0.195230073 | 8.09E-06 | 2.46E-05 |
| CCL26 | 0.287718972 | 2.84E-11 | 1.98E-10 |
| CCL27 | -0.097262312 | 0.027306734 | 0.044219963 |
| CCL28 | -0.238491452 | 4.29E-08 | 1.87E-07 |
| CCL3L3 | -0.119346612 | 0.006697511 | 0.01228923 |
| CCL4 | 0.0996863 | 0.023673753 | 0.038824333 |
| CCL7 | 0.199579286 | 5.01E-06 | 1.57E-05 |
| CCL8 | 0.166153113 | 0.00015198 | 0.000375311 |
| CCNA2 | 0.561194523 | 4.57E-44 | 7.03E-41 |
| CCNB1IP1 | 0.224432085 | 2.65E-07 | 1.02E-06 |
| CCNB1 | 0.598637682 | 2.17E-51 | 1.09E-47 |
| CCNB2 | 0.565093446 | 8.73E-45 | 1.59E-41 |
| CCNC | 0.212448639 | 1.14E-06 | 3.97E-06 |
| CCND2 | -0.229220874 | 1.45E-07 | 5.80E-07 |
| CCND3 | -0.194142219 | 9.11E-06 | 2.74E-05 |
| CCNDBP1 | -0.157030957 | 0.000347352 | 0.000800977 |
| CCNE1 | 0.331316058 | 1.17E-14 | 1.36E-13 |
| CCNE2 | 0.34315991 | 1.12E-15 | 1.53E-14 |
| CCNF | 0.32637213 | 3.02E-14 | 3.31E-13 |
| CCNG2 | 0.126577364 | 0.00401351 | 0.007661009 |
| CCNI | 0.129016749 | 0.003356946 | 0.006498658 |
| CCNJL | -0.241940421 | 2.70E-08 | 1.21E-07 |
| CCNJ | 0.232532805 | 9.42E-08 | 3.89E-07 |
| CCNK | 0.194303766 | 8.95E-06 | 2.70E-05 |
| CCNL1 | -0.277166697 | 1.55E-10 | 9.68E-10 |
| CCNL2 | -0.307445897 | 9.78E-13 | 8.58E-12 |
| CCNT1 | 0.118917093 | 0.006898734 | 0.012626306 |
| CCNT2 | -0.167719099 | 0.000131291 | 0.000327785 |
| CCNY | 0.153135665 | 0.000487838 | 0.001095731 |
| CCPG1 | -0.154037011 | 0.000451284 | 0.001020161 |
| CCR2 | -0.181045781 | 3.58E-05 | 9.80E-05 |
| CCR4 | -0.262036931 | 1.56E-09 | 8.41E-09 |
| CCR5 | -0.139930716 | 0.001455095 | 0.003004901 |
| CCR6 | -0.377647349 | 6.67E-19 | 1.49E-17 |
| CCR7 | -0.282379594 | 6.76E-11 | 4.46E-10 |
| CCR9 | -0.164494004 | 0.000177214 | 0.00043296 |
| CCRL1 | -0.169305755 | 0.000113049 | 0.000285163 |
| CCRL2 | -0.202851086 | 3.47E-06 | 1.12E-05 |
| CCRN4L | 0.426415418 | 3.61E-24 | 1.80E-22 |
| CCS | -0.10483929 | 0.017314386 | 0.029202594 |
| CCT2 | 0.399333492 | 3.88E-21 | 1.21E-19 |
| CCT3 | 0.468259718 | 2.00E-29 | 2.00E-27 |
| CCT4 | 0.46226084 | 1.26E-28 | 1.13E-26 |
| CCT5 | 0.458373997 | 4.06E-28 | 3.33E-26 |
| CCT6A | 0.450749111 | 3.88E-27 | 2.92E-25 |
| CCT6B | -0.317301788 | 1.65E-13 | 1.62E-12 |
| CCT6P1 | -0.121575695 | 0.005735326 | 0.010653071 |
| CCT7 | 0.48686943 | 5.22E-32 | 8.22E-30 |
| CCT8L2 | -0.102586133 | 0.019883492 | 0.033154378 |
| CCT8 | 0.392802649 | 1.90E-20 | 5.27E-19 |
| CD101 | -0.263026276 | 1.35E-09 | 7.32E-09 |
| CD109 | 0.206603513 | 2.26E-06 | 7.49E-06 |
| CD151 | -0.101222416 | 0.021594598 | 0.035751911 |
| CD160 | -0.188687941 | 1.63E-05 | 4.72E-05 |
| CD164L2 | -0.31283593 | 3.72E-13 | 3.48E-12 |
| CD180 | -0.13164677 | 0.002759614 | 0.005423894 |
| CD19 | -0.139702354 | 0.001481688 | 0.003056976 |
| CD1A | -0.331632042 | 1.10E-14 | 1.28E-13 |
| CD1B | -0.346541881 | 5.61E-16 | 8.06E-15 |
| CD1C | -0.398807399 | 4.42E-21 | 1.36E-19 |
| CD1D | -0.216019195 | 7.46E-07 | 2.67E-06 |
| CD1E | -0.423984964 | 6.93E-24 | 3.25E-22 |
| CD200R1 | -0.259934016 | 2.13E-09 | 1.12E-08 |
| CD207 | -0.383834012 | 1.60E-19 | 3.94E-18 |
| CD22 | -0.375455362 | 1.10E-18 | 2.37E-17 |
| CD247 | -0.129014671 | 0.003357461 | 0.006499025 |
| CD276 | 0.195324183 | 8.01E-06 | 2.43E-05 |
| CD27 | -0.155019828 | 0.00041434 | 0.000942509 |
| CD28 | -0.187498961 | 1.85E-05 | 5.31E-05 |
| CD2AP | 0.195891049 | 7.53E-06 | 2.29E-05 |
| CD2 | -0.135404027 | 0.00207325 | 0.004172805 |
| CD300C | -0.198467132 | 5.67E-06 | 1.76E-05 |
| CD300LB | -0.272240038 | 3.34E-10 | 1.99E-09 |
| CD300LF | -0.258749701 | 2.53E-09 | 1.31E-08 |
| CD300LG | -0.374951959 | 1.23E-18 | 2.64E-17 |
| CD302 | -0.423497203 | 7.90E-24 | 3.66E-22 |
| CD33 | -0.270979285 | 4.05E-10 | 2.39E-09 |
| CD34 | -0.299741887 | 3.76E-12 | 2.98E-11 |
| CD36 | -0.174164598 | 7.09E-05 | 0.000184588 |
| CD37 | -0.339342652 | 2.41E-15 | 3.15E-14 |
| CD3EAP | 0.111387162 | 0.011422173 | 0.020039182 |
| CD3E | -0.160552957 | 0.000253762 | 0.000601399 |
| CD40LG | -0.35993227 | 3.38E-17 | 5.85E-16 |
| CD40 | -0.20501975 | 2.72E-06 | 8.87E-06 |
| CD44 | -0.184891083 | 2.42E-05 | 6.81E-05 |
| CD47 | -0.238121081 | 4.51E-08 | 1.95E-07 |
| CD48 | -0.178431225 | 4.66E-05 | 0.000124951 |
| CD4 | -0.269837364 | 4.83E-10 | 2.81E-09 |
| CD52 | -0.256234241 | 3.65E-09 | 1.85E-08 |
| CD53 | -0.138050805 | 0.001687707 | 0.003447863 |
| CD59 | -0.185149502 | 2.36E-05 | 6.64E-05 |
| CD5L | -0.147649784 | 0.000776598 | 0.00168468 |
| CD5 | -0.250822454 | 7.89E-09 | 3.84E-08 |
| CD68 | -0.097205104 | 0.027397971 | 0.044354731 |
| CD69 | -0.22685476 | 1.95E-07 | 7.69E-07 |
| CD6 | -0.241608819 | 2.82E-08 | 1.26E-07 |
| CD74 | -0.383530792 | 1.71E-19 | 4.18E-18 |
| CD79A | -0.105715526 | 0.016396594 | 0.027816594 |
| CD79B | -0.141442849 | 0.001289784 | 0.002690203 |
| CD80 | -0.163759818 | 0.00018959 | 0.000460397 |
| CD81 | -0.373961122 | 1.54E-18 | 3.25E-17 |
| CD82 | -0.347235213 | 4.87E-16 | 7.07E-15 |
| CD83 | -0.397723127 | 5.77E-21 | 1.74E-19 |
| CD84 | -0.167881123 | 0.000129309 | 0.00032328 |
| CD86 | -0.118431431 | 0.007132769 | 0.013017693 |
| CD93 | -0.179714076 | 4.10E-05 | 0.000110895 |
| CD96 | -0.179538859 | 4.17E-05 | 0.000112754 |
| CD97 | -0.253102975 | 5.71E-09 | 2.83E-08 |
| CD99 | 0.145364243 | 0.000938264 | 0.002004062 |
| CD9 | -0.103085296 | 0.019287356 | 0.032245857 |
| CDADC1 | -0.205486308 | 2.57E-06 | 8.45E-06 |
| CDAN1 | -0.189568144 | 1.49E-05 | 4.34E-05 |
| CDA | 0.218991514 | 5.20E-07 | 1.92E-06 |
| CDC123 | 0.387953279 | 6.06E-20 | 1.57E-18 |
| CDC14A | -0.190026484 | 1.41E-05 | 4.15E-05 |
| CDC14B | -0.183918736 | 2.67E-05 | 7.48E-05 |
| CDC20 | 0.53314987 | 3.67E-39 | 2.62E-36 |
| CDC25A | 0.461807405 | 1.44E-28 | 1.29E-26 |
| CDC25C | 0.500856046 | 4.68E-34 | 1.10E-31 |
| CDC26 | 0.118939915 | 0.006887908 | 0.012609956 |
| CDC27 | 0.344422028 | 8.65E-16 | 1.20E-14 |
| CDC34 | 0.186220442 | 2.11E-05 | 6.01E-05 |
| CDC42BPA | -0.247753902 | 1.21E-08 | 5.73E-08 |
| CDC42BPG | -0.290266799 | 1.86E-11 | 1.34E-10 |
| CDC42EP1 | -0.244034916 | 2.03E-08 | 9.27E-08 |
| CDC42EP2 | 0.12875771 | 0.003421717 | 0.006611879 |
| CDC42EP3 | -0.22632583 | 2.09E-07 | 8.18E-07 |
| CDC42EP4 | -0.248286372 | 1.13E-08 | 5.34E-08 |
| CDC42SE2 | -0.113436168 | 0.009985192 | 0.017734495 |
| CDC45 | 0.460568905 | 2.10E-28 | 1.79E-26 |
| CDC5L | 0.27962685 | 1.05E-10 | 6.74E-10 |
| CDC6 | 0.494597122 | 3.97E-33 | 7.68E-31 |
| CDC73 | 0.304404305 | 1.67E-12 | 1.41E-11 |
| CDC7 | 0.288931237 | 2.32E-11 | 1.64E-10 |
| CDCA2 | 0.482186474 | 2.41E-31 | 3.42E-29 |
| CDCA3 | 0.501647651 | 3.56E-34 | 8.47E-32 |
| CDCA4 | 0.443057132 | 3.58E-26 | 2.38E-24 |
| CDCA5 | 0.507526917 | 4.57E-35 | 1.30E-32 |
| CDCA7L | -0.17741277 | 5.15E-05 | 0.000137277 |
| CDCA7 | 0.156589511 | 0.000361127 | 0.000829869 |
| CDCA8 | 0.48809563 | 3.49E-32 | 5.71E-30 |
| CDCP1 | 0.112651833 | 0.010515102 | 0.01857826 |
| CDCP2 | -0.11200491 | 0.010970792 | 0.019309985 |
| CDH11 | -0.097784319 | 0.026486225 | 0.0430272 |
| CDH16 | -0.130099806 | 0.003098021 | 0.006034227 |
| CDH17 | 0.150015142 | 0.000636745 | 0.001402452 |
| CDH18 | 0.207247699 | 2.10E-06 | 6.99E-06 |
| CDH19 | -0.189662657 | 1.47E-05 | 4.30E-05 |
| CDH1 | -0.158284032 | 0.000310875 | 0.000723709 |
| CDH20 | -0.352197785 | 1.74E-16 | 2.68E-15 |
| CDH22 | -0.148646645 | 0.00071451 | 0.001561106 |
| CDH23 | -0.40990497 | 2.75E-22 | 9.99E-21 |
| CDH24 | 0.212326858 | 1.16E-06 | 4.02E-06 |
| CDH26 | -0.15737934 | 0.000336829 | 0.000778328 |
| CDH2 | 0.145242975 | 0.000947654 | 0.002022822 |
| CDH4 | -0.159188885 | 0.000286793 | 0.000672819 |
| CDH5 | -0.224472001 | 2.64E-07 | 1.02E-06 |
| CDH6 | -0.130913938 | 0.00291549 | 0.005705944 |
| CDH7 | 0.148951588 | 0.000696456 | 0.001525061 |
| CDHR1 | -0.271660448 | 3.65E-10 | 2.16E-09 |
| CDHR3 | -0.186471147 | 2.05E-05 | 5.86E-05 |
| CDHR4 | -0.219549745 | 4.86E-07 | 1.80E-06 |
| CDIPT | -0.261293948 | 1.74E-09 | 9.29E-09 |
| CDK10 | -0.303486677 | 1.96E-12 | 1.64E-11 |
| CDK11A | -0.245366193 | 1.69E-08 | 7.81E-08 |
| CDK11B | -0.206099596 | 2.40E-06 | 7.91E-06 |
| CDK12 | 0.125385554 | 0.004374793 | 0.008298695 |
| CDK15 | -0.153700804 | 0.00046461 | 0.001047561 |
| CDK16 | 0.173387586 | 7.65E-05 | 0.000197919 |
| CDK17 | 0.184499779 | 2.52E-05 | 7.07E-05 |
| CDK18 | -0.418797214 | 2.74E-23 | 1.18E-21 |
| CDK19 | -0.214947469 | 8.49E-07 | 3.01E-06 |
| CDK1 | 0.524044698 | 1.15E-37 | 5.59E-35 |
| CDK20 | -0.239501939 | 3.75E-08 | 1.64E-07 |
| CDK2AP1 | 0.207212511 | 2.11E-06 | 7.01E-06 |
| CDK2 | 0.339095966 | 2.53E-15 | 3.30E-14 |
| CDK3 | -0.30050354 | 3.29E-12 | 2.64E-11 |
| CDK4 | 0.265135063 | 9.84E-10 | 5.49E-09 |
| CDK5R1 | 0.186630521 | 2.02E-05 | 5.77E-05 |
| CDK5R2 | 0.146849943 | 0.000829986 | 0.001789812 |
| CDK5RAP1 | 0.123882387 | 0.004872266 | 0.009175321 |
| CDK5RAP3 | -0.288616359 | 2.45E-11 | 1.72E-10 |
| CDK5 | 0.125840263 | 0.004233622 | 0.008045411 |
| CDK6 | 0.208652822 | 1.79E-06 | 6.02E-06 |
| CDK7 | 0.246084612 | 1.53E-08 | 7.11E-08 |
| CDK8 | 0.376973996 | 7.77E-19 | 1.72E-17 |
| CDK9 | -0.194009124 | 9.24E-06 | 2.78E-05 |
| CDKL2 | -0.362189401 | 2.08E-17 | 3.68E-16 |
| CDKL3 | -0.176048875 | 5.90E-05 | 0.000155318 |
| CDKL5 | -0.130370364 | 0.003036239 | 0.005922548 |
| CDKN1C | -0.180531013 | 3.77E-05 | 0.000102834 |
| CDKN2AIPNL | 0.217637491 | 6.14E-07 | 2.23E-06 |
| CDKN2D | 0.131860668 | 0.00271557 | 0.005347667 |
| CDKN3 | 0.603483893 | 2.08E-52 | 1.38E-48 |
| CDNF | -0.170259994 | 0.000103256 | 0.000262246 |
| CDO1 | -0.267674639 | 6.71E-10 | 3.83E-09 |
| CDON | -0.191995457 | 1.15E-05 | 3.41E-05 |
| CDRT15P | -0.210149044 | 1.50E-06 | 5.11E-06 |
| CDRT4 | -0.213052925 | 1.06E-06 | 3.72E-06 |
| CDS1 | -0.21995428 | 4.62E-07 | 1.72E-06 |
| CDSN | -0.150608869 | 0.000605512 | 0.001338529 |
| CDT1 | 0.410607113 | 2.29E-22 | 8.43E-21 |
| CDV3 | 0.162707024 | 0.000208757 | 0.000503778 |
| CDX2 | 0.136422173 | 0.001916294 | 0.003874066 |
| CDYL | 0.21197163 | 1.21E-06 | 4.18E-06 |
| CEACAM16 | 0.102614389 | 0.019849324 | 0.033106499 |
| CEACAM19 | 0.124420898 | 0.004688464 | 0.008855888 |
| CEACAM1 | -0.107068803 | 0.015062827 | 0.025779243 |
| CEACAM21 | -0.228643657 | 1.56E-07 | 6.21E-07 |
| CEACAM4 | -0.232424336 | 9.56E-08 | 3.95E-07 |
| CEACAM6 | -0.181323726 | 3.48E-05 | 9.55E-05 |
| CEACAM7 | -0.117523861 | 0.007589239 | 0.013785325 |
| CEACAM8 | -0.308000116 | 8.86E-13 | 7.82E-12 |
| CEBPA | -0.348220301 | 3.98E-16 | 5.84E-15 |
| CEBPB | 0.158070259 | 0.000316833 | 0.000736208 |
| CEBPD | -0.142063485 | 0.001227082 | 0.002568861 |
| CEBPG | 0.244914618 | 1.80E-08 | 8.28E-08 |
| CEBPZ | 0.292368976 | 1.31E-11 | 9.66E-11 |
| CECR1 | -0.269406453 | 5.16E-10 | 2.99E-09 |
| CECR2 | -0.222779954 | 3.26E-07 | 1.24E-06 |
| CECR4 | -0.191821512 | 1.17E-05 | 3.47E-05 |
| CECR5 | 0.103305018 | 0.019029925 | 0.031859464 |
| CECR6 | -0.193410436 | 9.86E-06 | 2.95E-05 |
| CELF1 | -0.110036559 | 0.012466712 | 0.021670114 |
| CELF2 | -0.381702987 | 2.62E-19 | 6.27E-18 |
| CELF4 | -0.129353071 | 0.003274513 | 0.006348299 |
| CELF6 | -0.386864385 | 7.84E-20 | 1.99E-18 |
| CELSR1 | -0.254807685 | 4.48E-09 | 2.24E-08 |
| CELSR2 | -0.239558717 | 3.72E-08 | 1.63E-07 |
| CEL | -0.098356229 | 0.025611742 | 0.041735488 |
| CEMP1 | -0.362624914 | 1.89E-17 | 3.37E-16 |
| CENPA | 0.54496671 | 3.58E-41 | 3.58E-38 |
| CENPBD1 | 0.115855759 | 0.008496782 | 0.015272742 |
| CENPC1 | -0.277188591 | 1.54E-10 | 9.66E-10 |
| CENPE | 0.445487477 | 1.78E-26 | 1.23E-24 |
| CENPF | 0.422790296 | 9.54E-24 | 4.36E-22 |
| CENPH | 0.400670918 | 2.79E-21 | 8.86E-20 |
| CENPI | 0.471807108 | 6.61E-30 | 7.13E-28 |
| CENPJ | 0.122051423 | 0.005546829 | 0.010337531 |
| CENPK | 0.398171831 | 5.17E-21 | 1.57E-19 |
| CENPL | 0.479031584 | 6.68E-31 | 8.51E-29 |
| CENPM | 0.346805255 | 5.32E-16 | 7.69E-15 |
| CENPN | 0.517179396 | 1.44E-36 | 5.53E-34 |
| CENPO | 0.454823895 | 1.17E-27 | 9.28E-26 |
| CENPP | 0.193594405 | 9.66E-06 | 2.90E-05 |
| CENPQ | 0.317714715 | 1.53E-13 | 1.51E-12 |
| CENPT | -0.202850961 | 3.47E-06 | 1.12E-05 |
| CENPW | 0.447500479 | 9.99E-27 | 7.08E-25 |
| CEP110 | -0.287915727 | 2.75E-11 | 1.92E-10 |
| CEP120 | -0.208327475 | 1.85E-06 | 6.24E-06 |
| CEP152 | 0.117413535 | 0.007646472 | 0.013877934 |
| CEP164 | -0.121373798 | 0.005817049 | 0.010789827 |
| CEP192 | -0.152125559 | 0.000532065 | 0.00118733 |
| CEP55 | 0.543240825 | 7.12E-41 | 6.78E-38 |
| CEP57 | 0.134056771 | 0.002299016 | 0.004586101 |
| CEP68 | -0.301989628 | 2.55E-12 | 2.08E-11 |
| CEP70 | -0.101144124 | 0.0216966 | 0.035897212 |
| CEP76 | 0.293419857 | 1.10E-11 | 8.17E-11 |
| CEP78 | 0.299709174 | 3.78E-12 | 3.00E-11 |
| CERCAM | 0.12277234 | 0.005271793 | 0.009864501 |
| CERKL | -0.258886051 | 2.48E-09 | 1.29E-08 |
| CERK | -0.140522441 | 0.001388215 | 0.002878088 |
| CES1 | -0.158297913 | 0.000310491 | 0.000723069 |
| CES2 | -0.319282445 | 1.14E-13 | 1.15E-12 |
| CES3 | -0.204325138 | 2.94E-06 | 9.55E-06 |
| CES4 | -0.139329916 | 0.001526019 | 0.003141951 |
| CES8 | -0.325668068 | 3.45E-14 | 3.76E-13 |
| CFC1B | -0.118238377 | 0.007227756 | 0.013179017 |
| CFD | -0.273425848 | 2.78E-10 | 1.67E-09 |
| CFHR1 | 0.123992031 | 0.004834324 | 0.009110737 |
| CFHR2 | 0.120244515 | 0.006293762 | 0.011601928 |
| CFHR4 | 0.165019005 | 0.000168832 | 0.000413861 |
| CFHR5 | 0.148743201 | 0.000708747 | 0.001550108 |
| CFH | -0.11117178 | 0.011583372 | 0.020282825 |
| CFI | -0.203877496 | 3.09E-06 | 1.00E-05 |
| CFL1 | 0.349980595 | 2.76E-16 | 4.13E-15 |
| CFL2 | 0.195641876 | 7.74E-06 | 2.35E-05 |
| CFLAR | -0.161130614 | 0.000240877 | 0.000573376 |
| CFLP1 | -0.248938903 | 1.03E-08 | 4.92E-08 |
| CFP | -0.292949061 | 1.19E-11 | 8.81E-11 |
| CFTR | -0.29665025 | 6.38E-12 | 4.91E-11 |
| CG030 | -0.33079943 | 1.29E-14 | 1.49E-13 |
| CGA | 0.181467958 | 3.43E-05 | 9.42E-05 |
| CGB1 | 0.106099885 | 0.016007641 | 0.027200606 |
| CGB7 | -0.26146882 | 1.70E-09 | 9.08E-09 |
| CGNL1 | -0.475116166 | 2.33E-30 | 2.75E-28 |
| CGN | -0.195417186 | 7.93E-06 | 2.41E-05 |
| CGREF1 | 0.294803667 | 8.72E-12 | 6.59E-11 |
| CGRRF1 | 0.191007643 | 1.27E-05 | 3.76E-05 |
| CH25H | -0.317652259 | 1.54E-13 | 1.52E-12 |
| CHAC1 | 0.226257702 | 2.11E-07 | 8.25E-07 |
| CHAC2 | 0.441507376 | 5.56E-26 | 3.61E-24 |
| CHADL | -0.404853734 | 9.85E-22 | 3.36E-20 |
| CHAD | -0.405919297 | 7.54E-22 | 2.61E-20 |
| CHAF1A | 0.250835376 | 7.88E-09 | 3.83E-08 |
| CHAF1B | 0.293841466 | 1.03E-11 | 7.65E-11 |
| CHAT | -0.112751779 | 0.010446218 | 0.018469616 |
| CHCHD10 | 0.128726876 | 0.003429502 | 0.006625641 |
| CHCHD1 | 0.232200991 | 9.84E-08 | 4.06E-07 |
| CHCHD2 | 0.337823799 | 3.26E-15 | 4.18E-14 |
| CHCHD3 | 0.410027009 | 2.66E-22 | 9.71E-21 |
| CHCHD4 | 0.222132091 | 3.53E-07 | 1.34E-06 |
| CHCHD5 | 0.104069211 | 0.01815776 | 0.030524585 |
| CHCHD7 | 0.11123474 | 0.011536044 | 0.020208804 |
| CHCHD8 | 0.363420952 | 1.59E-17 | 2.87E-16 |
| CHD1L | 0.26970437 | 4.93E-10 | 2.86E-09 |
| CHD2 | -0.31012704 | 6.06E-13 | 5.50E-12 |
| CHD3 | -0.175625944 | 6.15E-05 | 0.000161394 |
| CHD5 | -0.221452753 | 3.84E-07 | 1.44E-06 |
| CHD6 | -0.205483351 | 2.58E-06 | 8.45E-06 |
| CHD7 | 0.16450631 | 0.000177013 | 0.000432693 |
| CHD9 | -0.273073472 | 2.94E-10 | 1.76E-09 |
| CHDH | -0.160649208 | 0.000251571 | 0.000596702 |
| CHEK1 | 0.468065924 | 2.12E-29 | 2.12E-27 |
| CHEK2 | 0.345038989 | 7.63E-16 | 1.07E-14 |
| CHFR | 0.130091995 | 0.003099821 | 0.006037146 |
| CHI3L1 | -0.187657178 | 1.82E-05 | 5.23E-05 |
| CHI3L2 | -0.194976764 | 8.32E-06 | 2.52E-05 |
| CHIA | -0.313475948 | 3.32E-13 | 3.13E-12 |
| CHIC1 | -0.238742334 | 4.15E-08 | 1.81E-07 |
| CHIC2 | 0.139657054 | 0.001487016 | 0.003066386 |
| CHIT1 | -0.28433352 | 4.93E-11 | 3.31E-10 |
| CHKA | -0.205134248 | 2.68E-06 | 8.76E-06 |
| CHKB-CPT1B | -0.341156389 | 1.67E-15 | 2.24E-14 |
| CHKB | -0.144247214 | 0.001028109 | 0.002182974 |
| CHML | 0.218238641 | 5.70E-07 | 2.09E-06 |
| CHMP1A | 0.099911597 | 0.02335837 | 0.03837323 |
| CHMP1B | 0.173625998 | 7.47E-05 | 0.000193697 |
| CHMP2B | 0.205351781 | 2.61E-06 | 8.56E-06 |
| CHMP4A | 0.16343935 | 0.000195243 | 0.000473203 |
| CHMP4C | 0.327253586 | 2.55E-14 | 2.83E-13 |
| CHMP5 | 0.282596627 | 6.53E-11 | 4.31E-10 |
| CHMP6 | -0.199413875 | 5.11E-06 | 1.60E-05 |
| CHN2 | -0.239709082 | 3.65E-08 | 1.60E-07 |
| CHORDC1 | 0.404648215 | 1.04E-21 | 3.53E-20 |
| CHP2 | -0.135262548 | 0.002095967 | 0.004213436 |
| CHRAC1 | 0.290868055 | 1.69E-11 | 1.22E-10 |
| CHRDL1 | -0.376082449 | 9.52E-19 | 2.08E-17 |
| CHRM1 | -0.307609849 | 9.50E-13 | 8.35E-12 |
| CHRM2 | -0.146972619 | 0.000821583 | 0.001774563 |
| CHRNA10 | -0.268296839 | 6.11E-10 | 3.51E-09 |
| CHRNA2 | -0.291368509 | 1.55E-11 | 1.13E-10 |
| CHRNA4 | -0.101430999 | 0.021324875 | 0.035352211 |
| CHRNA5 | 0.302866802 | 2.19E-12 | 1.81E-11 |
| CHRNA6 | -0.230142292 | 1.28E-07 | 5.20E-07 |
| CHRNA9 | 0.306526612 | 1.15E-12 | 9.99E-12 |
| CHRNB3 | -0.288749375 | 2.40E-11 | 1.69E-10 |
| CHRNB4 | 0.117233883 | 0.007740495 | 0.01403329 |
| CHST10 | -0.229536746 | 1.39E-07 | 5.59E-07 |
| CHST11 | 0.161886217 | 0.000224944 | 0.000539046 |
| CHST12 | -0.152943757 | 0.000495968 | 0.001112242 |
| CHST14 | -0.107157644 | 0.014978699 | 0.025657231 |
| CHST1 | 0.145894114 | 0.000898232 | 0.001924522 |
| CHST2 | -0.179652801 | 4.12E-05 | 0.000111549 |
| CHST5 | -0.274652515 | 2.30E-10 | 1.40E-09 |
| CHST7 | -0.222807888 | 3.25E-07 | 1.24E-06 |
| CHST8 | -0.237849971 | 4.68E-08 | 2.02E-07 |
| CHST9 | -0.223322181 | 3.05E-07 | 1.16E-06 |
| CHSY3 | -0.095754004 | 0.02980151 | 0.047895199 |
| CHUK | 0.370983674 | 3.00E-18 | 6.05E-17 |
| CIAO1 | 0.196126754 | 7.34E-06 | 2.24E-05 |
| CIAPIN1 | 0.326288183 | 3.07E-14 | 3.36E-13 |
| CIB2 | 0.177344687 | 5.19E-05 | 0.000138114 |
| CIC | -0.24646461 | 1.45E-08 | 6.78E-08 |
| CIDEB | -0.141195704 | 0.001315564 | 0.002737973 |
| CIDEC | 0.163156927 | 0.000200354 | 0.000484653 |
| CIITA | -0.414285726 | 8.90E-23 | 3.51E-21 |
| CINP | 0.209867965 | 1.55E-06 | 5.27E-06 |
| CIR1 | -0.111376248 | 0.011430294 | 0.020048973 |
| CIRBP | -0.501824713 | 3.35E-34 | 8.27E-32 |
| CIRH1A | 0.324338038 | 4.44E-14 | 4.75E-13 |
| CISD1 | 0.359412894 | 3.78E-17 | 6.46E-16 |
| CISD2 | 0.397031408 | 6.83E-21 | 2.04E-19 |
| CISH | -0.373251918 | 1.81E-18 | 3.74E-17 |
| CITED1 | -0.105062019 | 0.017076934 | 0.028858112 |
| CITED2 | -0.35958904 | 3.64E-17 | 6.25E-16 |
| CKAP2L | 0.505558595 | 9.13E-35 | 2.53E-32 |
| CKAP2 | 0.46074296 | 1.99E-28 | 1.72E-26 |
| CKAP4 | 0.359248844 | 3.92E-17 | 6.67E-16 |
| CKAP5 | 0.245574953 | 1.64E-08 | 7.60E-08 |
| CKB | -0.132428362 | 0.002601764 | 0.005137218 |
| CKMT1B | 0.102371366 | 0.020144878 | 0.033552685 |
| CKMT2 | -0.261583358 | 1.67E-09 | 8.94E-09 |
| CKS1B | 0.499238535 | 8.17E-34 | 1.81E-31 |
| CKS2 | 0.483836776 | 1.41E-31 | 2.09E-29 |
| CLASP1 | -0.106276274 | 0.015831868 | 0.026936152 |
| CLCA2 | -0.136779295 | 0.001863871 | 0.003775723 |
| CLCA3P | 0.122743513 | 0.005282551 | 0.009880931 |
| CLCA4 | -0.104634699 | 0.01753504 | 0.029554801 |
| CLCC1 | -0.261461478 | 1.70E-09 | 9.09E-09 |
| CLCN4 | -0.108939588 | 0.013376265 | 0.023112447 |
| CLCN5 | -0.200480427 | 4.54E-06 | 1.43E-05 |
| CLCN6 | -0.386776464 | 8.01E-20 | 2.03E-18 |
| CLCN7 | -0.255549371 | 4.02E-09 | 2.03E-08 |
| CLCNKA | -0.270492495 | 4.37E-10 | 2.56E-09 |
| CLCNKB | -0.224179933 | 2.74E-07 | 1.05E-06 |
| CLDN10 | 0.160518702 | 0.000254546 | 0.000602971 |
| CLDN11 | -0.126131044 | 0.004145517 | 0.007894493 |
| CLDN12 | 0.255296308 | 4.17E-09 | 2.10E-08 |
| CLDN14 | 0.238927893 | 4.05E-08 | 1.77E-07 |
| CLDN15 | -0.227052735 | 1.91E-07 | 7.51E-07 |
| CLDN16 | -0.252396725 | 6.32E-09 | 3.10E-08 |
| CLDN18 | -0.292518204 | 1.28E-11 | 9.43E-11 |
| CLDN1 | -0.177648907 | 5.04E-05 | 0.000134381 |
| CLDN20 | -0.212550905 | 1.13E-06 | 3.92E-06 |
| CLDN23 | -0.291555109 | 1.50E-11 | 1.10E-10 |
| CLDN2 | -0.226553628 | 2.03E-07 | 7.97E-07 |
| CLDN3 | -0.112929679 | 0.010324597 | 0.018275598 |
| CLDN4 | -0.114980412 | 0.00901072 | 0.016118335 |
| CLDN5 | -0.130130438 | 0.003090969 | 0.006022253 |
| CLDN8 | -0.133844661 | 0.002336539 | 0.004653522 |
| CLDN9 | -0.198498902 | 5.65E-06 | 1.76E-05 |
| CLDND1 | 0.177264058 | 5.23E-05 | 0.000139099 |
| CLDND2 | -0.131884774 | 0.002710647 | 0.005340423 |
| CLEC10A | -0.280976985 | 8.46E-11 | 5.52E-10 |
| CLEC12A | -0.183022388 | 2.93E-05 | 8.14E-05 |
| CLEC12B | -0.313870226 | 3.09E-13 | 2.92E-12 |
| CLEC14A | -0.290297794 | 1.85E-11 | 1.33E-10 |
| CLEC16A | -0.380650002 | 3.34E-19 | 7.81E-18 |
| CLEC17A | -0.203054962 | 3.40E-06 | 1.09E-05 |
| CLEC18A | -0.15758266 | 0.000330826 | 0.000765634 |
| CLEC18B | -0.152594047 | 0.000511109 | 0.001143757 |
| CLEC1A | -0.270263611 | 4.53E-10 | 2.64E-09 |
| CLEC1B | -0.128333903 | 0.003530137 | 0.006803623 |
| CLEC2L | 0.125001991 | 0.004497168 | 0.00851548 |
| CLEC3A | -0.149982486 | 0.000638505 | 0.00140571 |
| CLEC3B | -0.312178891 | 4.19E-13 | 3.88E-12 |
| CLEC4A | -0.15810832 | 0.000315765 | 0.000734067 |
| CLEC4F | -0.464180745 | 7.00E-29 | 6.54E-27 |
| CLEC4GP1 | -0.200736977 | 4.41E-06 | 1.39E-05 |
| CLEC5A | -0.157910383 | 0.000321359 | 0.000745944 |
| CLEC7A | -0.180215055 | 3.90E-05 | 0.000105883 |
| CLEC9A | -0.310040043 | 6.16E-13 | 5.57E-12 |
| CLECL1 | -0.254457075 | 4.71E-09 | 2.35E-08 |
| CLGN | 0.183366288 | 2.83E-05 | 7.87E-05 |
| CLIC1 | 0.312574854 | 3.90E-13 | 3.63E-12 |
| CLIC2 | -0.159500685 | 0.000278907 | 0.000655935 |
| CLIC3 | -0.266937172 | 7.50E-10 | 4.25E-09 |
| CLIC4 | 0.145656731 | 0.000915967 | 0.001959789 |
| CLIC5 | -0.389214935 | 4.49E-20 | 1.18E-18 |
| CLIC6 | -0.322250512 | 6.57E-14 | 6.84E-13 |
| CLINT1 | -0.154649256 | 0.000427926 | 0.000971975 |
| CLIP1 | 0.172945864 | 7.98E-05 | 0.000205869 |
| CLIP2 | -0.118712151 | 0.006996641 | 0.012786766 |
| CLIP3 | -0.193564264 | 9.69E-06 | 2.91E-05 |
| CLK1 | -0.311916724 | 4.40E-13 | 4.05E-12 |
| CLK2 | -0.158762197 | 0.000297923 | 0.000696315 |
| CLK3 | -0.121857539 | 0.005622968 | 0.010467717 |
| CLK4 | -0.421444419 | 1.36E-23 | 6.13E-22 |
| CLN3 | -0.210729745 | 1.40E-06 | 4.80E-06 |
| CLN5 | -0.179357287 | 4.25E-05 | 0.000114683 |
| CLN8 | -0.158286419 | 0.000310809 | 0.00072364 |
| CLNK | -0.106651932 | 0.015463153 | 0.026374052 |
| CLNS1A | 0.396539312 | 7.70E-21 | 2.28E-19 |
| CLOCK | 0.11033612 | 0.012228021 | 0.021297812 |
| CLP1 | 0.218803243 | 5.32E-07 | 1.96E-06 |
| CLPB | 0.293228718 | 1.14E-11 | 8.42E-11 |
| CLPTM1L | -0.197596313 | 6.25E-06 | 1.93E-05 |
| CLPX | 0.209516178 | 1.61E-06 | 5.48E-06 |
| CLSPN | 0.360400831 | 3.06E-17 | 5.32E-16 |
| CLTA | 0.142786435 | 0.001157589 | 0.002434029 |
| CLUAP1 | -0.278201397 | 1.32E-10 | 8.32E-10 |
| CLUL1 | -0.400702388 | 2.77E-21 | 8.80E-20 |
| CLU | -0.312034468 | 4.30E-13 | 3.97E-12 |
| CLVS2 | 0.096713649 | 0.028192621 | 0.045536493 |
| CLYBL | -0.138937289 | 0.001574069 | 0.003228246 |
| CMA1 | -0.289918806 | 1.97E-11 | 1.41E-10 |
| CMAH | -0.325345214 | 3.67E-14 | 3.98E-13 |
| CMAS | 0.320335836 | 9.40E-14 | 9.55E-13 |
| CMBL | 0.170284275 | 0.000103017 | 0.000261674 |
| CMC1 | 0.27122323 | 3.91E-10 | 2.30E-09 |
| CMKLR1 | -0.153903917 | 0.000456516 | 0.001030823 |
| CMTM5 | -0.288707129 | 2.41E-11 | 1.70E-10 |
| CMTM7 | -0.302137779 | 2.48E-12 | 2.03E-11 |
| CMYA5 | -0.390507356 | 3.30E-20 | 8.90E-19 |
| CNBP | 0.190582493 | 1.33E-05 | 3.92E-05 |
| CNDP2 | -0.135913646 | 0.001993275 | 0.004022676 |
| CNFN | -0.098674653 | 0.025135732 | 0.041009959 |
| CNGA1 | -0.131657917 | 0.002757303 | 0.005421303 |
| CNGA4 | -0.297120888 | 5.89E-12 | 4.55E-11 |
| CNGB3 | 0.264545473 | 1.07E-09 | 5.95E-09 |
| CNIH | 0.518008801 | 1.06E-36 | 4.17E-34 |
| CNKSR1 | -0.345478791 | 6.98E-16 | 9.85E-15 |
| CNKSR2 | -0.365550385 | 9.99E-18 | 1.88E-16 |
| CNN1 | -0.107748659 | 0.014429464 | 0.024780147 |
| CNN2 | -0.10386733 | 0.018384705 | 0.030869748 |
| CNNM1 | -0.15548821 | 0.000397743 | 0.000907025 |
| CNNM2 | -0.129983159 | 0.003125008 | 0.006083827 |
| CNNM3 | -0.365367276 | 1.04E-17 | 1.94E-16 |
| CNOT10 | 0.177054467 | 5.34E-05 | 0.000141722 |
| CNOT2 | 0.173462113 | 7.59E-05 | 0.000196545 |
| CNOT6L | -0.160003652 | 0.000266611 | 0.000629239 |
| CNOT7 | 0.178028731 | 4.85E-05 | 0.000129795 |
| CNO | 0.310831796 | 5.34E-13 | 4.87E-12 |
| CNPY2 | 0.313427794 | 3.34E-13 | 3.15E-12 |
| CNP | 0.095408478 | 0.030399766 | 0.048731521 |
| CNR1 | -0.353512483 | 1.32E-16 | 2.06E-15 |
| CNR2 | -0.256258785 | 3.63E-09 | 1.84E-08 |
| CNRIP1 | -0.210431458 | 1.45E-06 | 4.95E-06 |
| CNTD1 | -0.269407567 | 5.16E-10 | 2.99E-09 |
| CNTD2 | 0.21560466 | 7.85E-07 | 2.80E-06 |
| CNTFR | -0.372745205 | 2.02E-18 | 4.17E-17 |
| CNTN2 | -0.287546924 | 2.92E-11 | 2.03E-10 |
| CNTN3 | -0.179484151 | 4.19E-05 | 0.000113312 |
| CNTN4 | -0.26822801 | 6.17E-10 | 3.54E-09 |
| CNTN6 | -0.336306722 | 4.40E-15 | 5.52E-14 |
| CNTNAP1 | -0.176601802 | 5.58E-05 | 0.000147612 |
| CNTNAP4 | 0.120943827 | 0.00599459 | 0.011092378 |
| CNTROB | -0.291538178 | 1.51E-11 | 1.10E-10 |
| COASY | 0.156457212 | 0.000365353 | 0.000838617 |
| COBLL1 | -0.302035812 | 2.53E-12 | 2.07E-11 |
| COBL | -0.136406759 | 0.001918587 | 0.003877917 |
| COCH | 0.247243539 | 1.30E-08 | 6.12E-08 |
| COG2 | 0.136251853 | 0.001941769 | 0.003923582 |
| COG4 | -0.105010706 | 0.017131384 | 0.028940341 |
| COG5 | 0.216913338 | 6.70E-07 | 2.42E-06 |
| COG6 | 0.173706167 | 7.41E-05 | 0.000192371 |
| COG7 | -0.21018729 | 1.49E-06 | 5.09E-06 |
| COIL | 0.186150963 | 2.12E-05 | 6.05E-05 |
| COL11A1 | 0.264462668 | 1.09E-09 | 6.02E-09 |
| COL11A2 | -0.244621976 | 1.87E-08 | 8.60E-08 |
| COL12A1 | 0.186096075 | 2.14E-05 | 6.08E-05 |
| COL13A1 | -0.314818432 | 2.60E-13 | 2.48E-12 |
| COL14A1 | -0.288346932 | 2.56E-11 | 1.79E-10 |
| COL15A1 | 0.110716093 | 0.011931072 | 0.020818705 |
| COL16A1 | -0.220037984 | 4.58E-07 | 1.70E-06 |
| COL17A1 | -0.111884751 | 0.011057332 | 0.019448606 |
| COL19A1 | -0.28479276 | 4.58E-11 | 3.09E-10 |
| COL1A1 | 0.115226688 | 0.008863398 | 0.015876118 |
| COL1A2 | 0.112928198 | 0.010325604 | 0.018275762 |
| COL21A1 | -0.38040045 | 3.54E-19 | 8.25E-18 |
| COL23A1 | -0.199701491 | 4.95E-06 | 1.55E-05 |
| COL24A1 | -0.128656556 | 0.003447316 | 0.006656841 |
| COL27A1 | -0.266830017 | 7.63E-10 | 4.32E-09 |
| COL28A1 | -0.208385774 | 1.84E-06 | 6.20E-06 |
| COL29A1 | -0.363753086 | 1.48E-17 | 2.68E-16 |
| COL3A1 | 0.157760882 | 0.000325646 | 0.000755281 |
| COL4A3BP | -0.312036078 | 4.30E-13 | 3.97E-12 |
| COL4A3 | -0.430687085 | 1.13E-24 | 5.92E-23 |
| COL4A4 | -0.381950099 | 2.48E-19 | 5.94E-18 |
| COL4A5 | -0.130083546 | 0.00310177 | 0.006040352 |
| COL4A6 | -0.129094663 | 0.003337683 | 0.006466377 |
| COL5A2 | 0.179820634 | 4.05E-05 | 0.000109865 |
| COL6A4P2 | -0.226976953 | 1.92E-07 | 7.58E-07 |
| COL6A6 | -0.362957092 | 1.76E-17 | 3.15E-16 |
| COL8A1 | -0.117627511 | 0.007535819 | 0.013692023 |
| COL8A2 | -0.189766743 | 1.45E-05 | 4.26E-05 |
| COL9A1 | -0.141746233 | 0.001258775 | 0.00263239 |
| COL9A2 | -0.337915499 | 3.20E-15 | 4.11E-14 |
| COLEC10 | -0.101715228 | 0.020962044 | 0.034802669 |
| COLEC12 | -0.350686678 | 2.39E-16 | 3.58E-15 |
| COLQ | -0.323668019 | 5.03E-14 | 5.33E-13 |
| COMMD10 | 0.114877619 | 0.009072853 | 0.016222712 |
| COMMD1 | 0.102799683 | 0.019626521 | 0.032767559 |
| COMMD2 | 0.159854143 | 0.000270212 | 0.000637211 |
| COMMD3 | 0.106123146 | 0.015984364 | 0.027167982 |
| COMMD5 | 0.185783423 | 2.21E-05 | 6.26E-05 |
| COMMD7 | 0.171856484 | 8.86E-05 | 0.000227189 |
| COMMD8 | 0.26204745 | 1.56E-09 | 8.40E-09 |
| COMTD1 | 0.154949344 | 0.000416893 | 0.000947884 |
| COPA | 0.161978346 | 0.00022307 | 0.000534813 |
| COPB1 | 0.283184338 | 5.94E-11 | 3.95E-10 |
| COPB2 | 0.280598539 | 8.99E-11 | 5.83E-10 |
| COPE | 0.113010433 | 0.010269806 | 0.0181915 |
| COPG2 | 0.108366429 | 0.013874371 | 0.023909038 |
| COPG | 0.192583418 | 1.08E-05 | 3.21E-05 |
| COPS2 | 0.252432657 | 6.28E-09 | 3.09E-08 |
| COPS3 | 0.300954763 | 3.05E-12 | 2.46E-11 |
| COPS4 | 0.169720015 | 0.000108695 | 0.000275361 |
| COPS5 | 0.370243703 | 3.54E-18 | 7.06E-17 |
| COPS6 | 0.285483309 | 4.09E-11 | 2.78E-10 |
| COPS7A | 0.13114758 | 0.002864944 | 0.005611417 |
| COPS8 | 0.275465092 | 2.02E-10 | 1.24E-09 |
| COPZ1 | 0.371798823 | 2.50E-18 | 5.10E-17 |
| COQ10A | -0.174822011 | 6.65E-05 | 0.000173922 |
| COQ10B | 0.133165358 | 0.002460506 | 0.004880479 |
| COQ2 | 0.240513804 | 3.27E-08 | 1.45E-07 |
| COQ3 | 0.223444169 | 3.00E-07 | 1.15E-06 |
| COQ4 | -0.19215412 | 1.13E-05 | 3.35E-05 |
| COQ5 | 0.286781447 | 3.31E-11 | 2.28E-10 |
| COQ6 | 0.138031778 | 0.001690227 | 0.003452657 |
| COQ7 | -0.155792 | 0.000387311 | 0.000885156 |
| COQ9 | 0.128030244 | 0.003609726 | 0.006942288 |
| CORO1A | -0.252767599 | 5.99E-09 | 2.96E-08 |
| CORO1C | 0.205408756 | 2.60E-06 | 8.51E-06 |
| CORO2A | -0.19360526 | 9.65E-06 | 2.90E-05 |
| CORO2B | -0.356259574 | 7.41E-17 | 1.21E-15 |
| CORO7 | -0.301309218 | 2.87E-12 | 2.32E-11 |
| CORT | -0.100410653 | 0.022672686 | 0.037348084 |
| COX10 | 0.156208575 | 0.000373421 | 0.000855526 |
| COX15 | 0.228473758 | 1.59E-07 | 6.33E-07 |
| COX16 | 0.206805661 | 2.21E-06 | 7.33E-06 |
| COX17 | 0.165440396 | 0.000162376 | 0.000399061 |
| COX4I1 | 0.105286575 | 0.016840423 | 0.028489748 |
| COX4I2 | -0.243534354 | 2.17E-08 | 9.87E-08 |
| COX4NB | 0.350319923 | 2.58E-16 | 3.86E-15 |
| COX5A | 0.293931572 | 1.01E-11 | 7.54E-11 |
| COX5B | 0.202134085 | 3.77E-06 | 1.20E-05 |
| COX6A1 | 0.262997012 | 1.35E-09 | 7.35E-09 |
| COX6B1 | 0.186513334 | 2.05E-05 | 5.83E-05 |
| COX6C | 0.227922473 | 1.71E-07 | 6.77E-07 |
| COX7A1 | -0.16009393 | 0.000264458 | 0.000624379 |
| COX7A2L | 0.29536463 | 7.93E-12 | 6.04E-11 |
| COX7A2 | 0.247149896 | 1.32E-08 | 6.19E-08 |
| COX7B2 | 0.187281581 | 1.89E-05 | 5.42E-05 |
| COX7B | 0.226171639 | 2.13E-07 | 8.32E-07 |
| COX8A | 0.19597809 | 7.46E-06 | 2.27E-05 |
| CP110 | -0.237308103 | 5.03E-08 | 2.16E-07 |
| CPA3 | -0.306349145 | 1.19E-12 | 1.03E-11 |
| CPA4 | 0.160278467 | 0.000260109 | 0.000615054 |
| CPAMD8 | -0.488388835 | 3.16E-32 | 5.23E-30 |
| CPB2 | -0.186822935 | 1.98E-05 | 5.66E-05 |
| CPD | 0.249054746 | 1.01E-08 | 4.85E-08 |
| CPEB1 | -0.174827592 | 6.65E-05 | 0.00017385 |
| CPEB3 | -0.326018696 | 3.23E-14 | 3.53E-13 |
| CPEB4 | -0.287025198 | 3.18E-11 | 2.20E-10 |
| CPE | 0.287635673 | 2.88E-11 | 2.00E-10 |
| CPLX2 | 0.228589693 | 1.57E-07 | 6.25E-07 |
| CPLX4 | 0.104112279 | 0.018109663 | 0.030453975 |
| CPM | -0.117902276 | 0.00739583 | 0.013463374 |
| CPN1 | 0.222260914 | 3.48E-07 | 1.32E-06 |
| CPNE1 | 0.096720788 | 0.028180937 | 0.04552498 |
| CPNE2 | -0.372454252 | 2.16E-18 | 4.44E-17 |
| CPNE3 | 0.182004 | 3.25E-05 | 8.95E-05 |
| CPNE7 | -0.124691259 | 0.004598566 | 0.008694299 |
| CPNE9 | -0.286184118 | 3.65E-11 | 2.50E-10 |
| CPOX | 0.148295609 | 0.000735829 | 0.001604424 |
| CPO | -0.200702258 | 4.42E-06 | 1.40E-05 |
| CPS1 | 0.309724095 | 6.52E-13 | 5.88E-12 |
| CPSF2 | 0.393804208 | 1.50E-20 | 4.19E-19 |
| CPSF3L | -0.168215403 | 0.000125307 | 0.000313865 |
| CPSF3 | 0.37027489 | 3.52E-18 | 7.03E-17 |
| CPSF4 | 0.203230819 | 3.33E-06 | 1.07E-05 |
| CPSF6 | 0.120185904 | 0.006319435 | 0.0116437 |
| CPT1B | -0.249775319 | 9.15E-09 | 4.40E-08 |
| CPT1C | 0.095498851 | 0.030242306 | 0.048529481 |
| CPT2 | -0.142182885 | 0.001215346 | 0.0025461 |
| CPVL | -0.218234208 | 5.71E-07 | 2.09E-06 |
| CPXM1 | 0.106121418 | 0.015986091 | 0.027168608 |
| CPXM2 | -0.161284031 | 0.000237559 | 0.000566153 |
| CPZ | -0.102889064 | 0.019519827 | 0.032603184 |
| CR1 | -0.179577094 | 4.15E-05 | 0.000112344 |
| CR2 | -0.176775706 | 5.49E-05 | 0.000145293 |
| CRABP1 | 0.144698403 | 0.000990903 | 0.002108835 |
| CRADD | 0.103222381 | 0.019126391 | 0.031999524 |
| CRAMP1L | -0.302448611 | 2.35E-12 | 1.93E-11 |
| CRAT | -0.132117925 | 0.002663447 | 0.005253304 |
| CRB1 | -0.122147368 | 0.005509495 | 0.010272742 |
| CRB2 | -0.232101154 | 9.97E-08 | 4.10E-07 |
| CRB3 | -0.148025403 | 0.000752639 | 0.001638039 |
| CRBN | -0.107860608 | 0.014327447 | 0.024621874 |
| CRCP | 0.16232181 | 0.000216213 | 0.000520056 |
| CREB3L1 | -0.142410568 | 0.001193253 | 0.002502964 |
| CREB3 | 0.115081406 | 0.008950044 | 0.016022703 |
| CREB5 | -0.103272204 | 0.01906818 | 0.031912817 |
| CREBBP | -0.259953296 | 2.12E-09 | 1.12E-08 |
| CREBL2 | -0.196884919 | 6.75E-06 | 2.07E-05 |
| CREBZF | -0.14092578 | 0.001344261 | 0.002792173 |
| CREG2 | 0.241848002 | 2.73E-08 | 1.22E-07 |
| CRELD1 | -0.179872289 | 4.03E-05 | 0.000109356 |
| CREM | 0.262007436 | 1.57E-09 | 8.44E-09 |
| CRHBP | -0.268102574 | 6.29E-10 | 3.60E-09 |
| CRIM1 | -0.164546042 | 0.000176366 | 0.000431375 |
| CRIP2 | -0.123343363 | 0.005062728 | 0.009503551 |
| CRIPAK | -0.175248296 | 6.38E-05 | 0.000167158 |
| CRIPT | 0.296262215 | 6.81E-12 | 5.23E-11 |
| CRISP2 | -0.095269581 | 0.030643141 | 0.049078134 |
| CRK | 0.122265305 | 0.005463914 | 0.010196314 |
| CRMP1 | -0.188367762 | 1.69E-05 | 4.88E-05 |
| CRNKL1 | 0.137648291 | 0.001741749 | 0.003547028 |
| CROCCL1 | -0.347518964 | 4.59E-16 | 6.69E-15 |
| CROCCL2 | -0.432612166 | 6.67E-25 | 3.67E-23 |
| CROCC | -0.412453036 | 1.43E-22 | 5.39E-21 |
| CROT | -0.179788938 | 4.07E-05 | 0.00011017 |
| CRP | 0.114651695 | 0.009210754 | 0.01645995 |
| CRTAC1 | -0.348051397 | 4.12E-16 | 6.03E-15 |
| CRTAM | -0.122674945 | 0.005308218 | 0.009924297 |
| CRTAP | -0.129910218 | 0.003141992 | 0.006112724 |
| CRTC1 | -0.327791816 | 2.30E-14 | 2.57E-13 |
| CRY1 | 0.175451779 | 6.25E-05 | 0.000164109 |
| CRY2 | -0.509665008 | 2.14E-35 | 6.70E-33 |
| CRYBA2 | 0.134965167 | 0.002144462 | 0.004303564 |
| CRYBA4 | -0.104336536 | 0.017861013 | 0.030058595 |
| CRYBB3 | -0.171264643 | 9.38E-05 | 0.000239721 |
| CRYBG3 | -0.134475438 | 0.002226562 | 0.00445357 |
| CRYGC | 0.098684513 | 0.025121116 | 0.040992805 |
| CRYGS | -0.310563668 | 5.61E-13 | 5.11E-12 |
| CRYM | -0.32826218 | 2.10E-14 | 2.36E-13 |
| CRYZL1 | -0.111227341 | 0.011541597 | 0.020216759 |
| CSAD | -0.346156193 | 6.07E-16 | 8.67E-15 |
| CSAG1 | 0.194891319 | 8.40E-06 | 2.54E-05 |
| CSAG2 | 0.148003324 | 0.000754028 | 0.001640348 |
| CSAG3 | 0.198665816 | 5.55E-06 | 1.73E-05 |
| CSDAP1 | 0.15418104 | 0.000445685 | 0.001008758 |
| CSDA | 0.105025427 | 0.017115748 | 0.02891637 |
| CSDC2 | -0.242444036 | 2.52E-08 | 1.14E-07 |
| CSE1L | 0.450491434 | 4.19E-27 | 3.11E-25 |
| CSF1R | -0.229035693 | 1.48E-07 | 5.93E-07 |
| CSF1 | -0.22932709 | 1.43E-07 | 5.73E-07 |
| CSF2RA | -0.179736057 | 4.09E-05 | 0.000110696 |
| CSF2RB | -0.222046701 | 3.57E-07 | 1.35E-06 |
| CSF2 | -0.133907704 | 0.002325328 | 0.004633965 |
| CSF3R | -0.246398637 | 1.46E-08 | 6.83E-08 |
| CSGALNACT1 | 0.143836205 | 0.001063118 | 0.002249826 |
| CSMD1 | -0.143160609 | 0.001123063 | 0.00236741 |
| CSN3 | -0.108354389 | 0.013885008 | 0.023925306 |
| CSNK1D | -0.143127334 | 0.001126094 | 0.00237305 |
| CSNK1E | 0.14321278 | 0.001118325 | 0.00235854 |
| CSNK2A1P | 0.206162535 | 2.38E-06 | 7.86E-06 |
| CSNK2A1 | 0.211459319 | 1.29E-06 | 4.43E-06 |
| CSNK2A2 | 0.103496509 | 0.018808018 | 0.031509066 |
| CSNK2B | 0.204072704 | 3.02E-06 | 9.80E-06 |
| CSPG5 | -0.214102136 | 9.39E-07 | 3.32E-06 |
| CSRNP1 | -0.280798046 | 8.71E-11 | 5.66E-10 |
| CSRNP2 | 0.097465329 | 0.026985061 | 0.043762873 |
| CSRP1 | -0.169970601 | 0.000106138 | 0.000269259 |
| CSRP2 | 0.200762916 | 4.39E-06 | 1.39E-05 |
| CST2 | -0.121449325 | 0.005786356 | 0.010736881 |
| CST3 | -0.249446392 | 9.58E-09 | 4.60E-08 |
| CST5 | -0.367651722 | 6.30E-18 | 1.21E-16 |
| CSTF1 | 0.22111811 | 4.01E-07 | 1.50E-06 |
| CSTF2 | 0.294035651 | 9.93E-12 | 7.42E-11 |
| CSTF3 | 0.329397084 | 1.69E-14 | 1.93E-13 |
| CS | 0.316786422 | 1.81E-13 | 1.77E-12 |
| CT45A4 | 0.121216239 | 0.005881551 | 0.010901377 |
| CT62 | -0.159162377 | 0.000287473 | 0.000674177 |
| CTAG1B | 0.134438491 | 0.002232871 | 0.004464793 |
| CTAG2 | 0.160604115 | 0.000252596 | 0.000598918 |
| CTAGE1 | 0.209454586 | 1.63E-06 | 5.51E-06 |
| CTAGE5 | 0.242941084 | 2.35E-08 | 1.06E-07 |
| CTBP1 | -0.101441215 | 0.021311739 | 0.035336296 |
| CTBP2 | 0.291199157 | 1.60E-11 | 1.16E-10 |
| CTBS | 0.157663364 | 0.000328471 | 0.000760949 |
| CTCFL | 0.170965328 | 9.65E-05 | 0.000246275 |
| CTDSP1 | -0.294940044 | 8.52E-12 | 6.45E-11 |
| CTDSP2 | -0.190630276 | 1.33E-05 | 3.90E-05 |
| CTDSPL2 | 0.192414659 | 1.10E-05 | 3.26E-05 |
| CTDSPL | -0.359853172 | 3.44E-17 | 5.94E-16 |
| CTF1 | -0.34002401 | 2.10E-15 | 2.77E-14 |
| CTGF | -0.099742172 | 0.023595199 | 0.038714565 |
| CTHRC1 | 0.332460447 | 9.34E-15 | 1.11E-13 |
| CTH | 0.160606384 | 0.000252544 | 0.000598867 |
| CTLA4 | -0.111039006 | 0.011683744 | 0.02043173 |
| CTNNAL1 | 0.101619443 | 0.021083714 | 0.034985349 |
| CTNNBIP1 | -0.309034677 | 7.37E-13 | 6.59E-12 |
| CTNNBL1 | 0.111834532 | 0.011093679 | 0.019503584 |
| CTNS | -0.120485157 | 0.006189334 | 0.011421012 |
| CTPS | 0.317473923 | 1.60E-13 | 1.57E-12 |
| CTRC | -0.148274716 | 0.000737116 | 0.00160688 |
| CTRL | -0.111428336 | 0.011391584 | 0.019990781 |
| CTSD | -0.338637571 | 2.77E-15 | 3.60E-14 |
| CTSE | -0.221310541 | 3.91E-07 | 1.47E-06 |
| CTSF | -0.292163171 | 1.36E-11 | 9.98E-11 |
| CTSG | -0.336835148 | 3.96E-15 | 5.01E-14 |
| CTSH | -0.478355877 | 8.30E-31 | 1.04E-28 |
| CTSL1 | 0.30389051 | 1.83E-12 | 1.53E-11 |
| CTSL2 | 0.338056481 | 3.11E-15 | 4.01E-14 |
| CTSO | -0.329606413 | 1.62E-14 | 1.86E-13 |
| CTSS | -0.180664204 | 3.72E-05 | 0.00010156 |
| CTSW | -0.139299645 | 0.001529675 | 0.003147534 |
| CTSZ | -0.204335175 | 2.94E-06 | 9.54E-06 |
| CTTNBP2NL | -0.099090396 | 0.024525749 | 0.040106412 |
| CTTNBP2 | -0.302663374 | 2.27E-12 | 1.87E-11 |
| CTU1 | 0.12817173 | 0.003572442 | 0.006875876 |
| CTU2 | 0.141053639 | 0.001330596 | 0.002766091 |
| CTXN1 | 0.104934775 | 0.017212238 | 0.029052373 |
| CTXN3 | -0.103593985 | 0.018695927 | 0.031352814 |
| CUBN | -0.305029517 | 1.50E-12 | 1.27E-11 |
| CUEDC1 | -0.192473255 | 1.09E-05 | 3.24E-05 |
| CUEDC2 | 0.097386223 | 0.027110013 | 0.043936993 |
| CUL1 | 0.125708805 | 0.004274005 | 0.008119838 |
| CUL2 | 0.349158722 | 3.28E-16 | 4.88E-15 |
| CUL4B | 0.223373547 | 3.03E-07 | 1.16E-06 |
| CUL9 | -0.370135739 | 3.63E-18 | 7.23E-17 |
| CUTA | -0.124287506 | 0.0047334 | 0.008933169 |
| CUTC | 0.11605346 | 0.00838439 | 0.015096584 |
| CUX1 | -0.154196969 | 0.000445069 | 0.001007594 |
| CUX2 | -0.156849126 | 0.000352965 | 0.00081289 |
| CWC15 | 0.160331643 | 0.000258868 | 0.000612482 |
| CWC22 | 0.097028266 | 0.027681659 | 0.044783569 |
| CWC25 | -0.259319935 | 2.33E-09 | 1.22E-08 |
| CWC27 | 0.120116941 | 0.006349762 | 0.011694374 |
| CWF19L1 | 0.266423276 | 8.11E-10 | 4.57E-09 |
| CWH43 | -0.108463742 | 0.013788668 | 0.0237798 |
| CX3CL1 | -0.319515891 | 1.09E-13 | 1.11E-12 |
| CX3CR1 | -0.415395852 | 6.68E-23 | 2.69E-21 |
| CXADR | -0.102147363 | 0.020420683 | 0.03396876 |
| CXCL10 | 0.120646583 | 0.006120157 | 0.011303797 |
| CXCL12 | -0.198204402 | 5.84E-06 | 1.81E-05 |
| CXCL14 | -0.210494354 | 1.44E-06 | 4.92E-06 |
| CXCL16 | -0.301339587 | 2.85E-12 | 2.31E-11 |
| CXCL17 | -0.299376613 | 4.00E-12 | 3.16E-11 |
| CXCL2 | -0.108288236 | 0.013943576 | 0.024017942 |
| CXCL5 | 0.252467765 | 6.25E-09 | 3.08E-08 |
| CXCR2P1 | -0.09671831 | 0.028184992 | 0.04552785 |
| CXCR2 | -0.15836201 | 0.000308727 | 0.000719212 |
| CXCR3 | -0.121630174 | 0.005713451 | 0.010614413 |
| CXCR4 | -0.177581473 | 5.07E-05 | 0.000135217 |
| CXCR5 | -0.212614032 | 1.12E-06 | 3.90E-06 |
| CXCR7 | 0.155611684 | 0.000393472 | 0.000898311 |
| CXXC1 | -0.127020127 | 0.003886326 | 0.007435412 |
| CXXC5 | -0.186118646 | 2.13E-05 | 6.07E-05 |
| CXorf1 | -0.17736154 | 5.18E-05 | 0.000137902 |
| CXorf21 | -0.248107922 | 1.15E-08 | 5.47E-08 |
| CXorf22 | -0.243613695 | 2.15E-08 | 9.77E-08 |
| CXorf23 | -0.300718908 | 3.17E-12 | 2.55E-11 |
| CXorf26 | 0.155382591 | 0.00040143 | 0.000914809 |
| CXorf30 | -0.276255827 | 1.79E-10 | 1.11E-09 |
| CXorf36 | -0.186582049 | 2.03E-05 | 5.80E-05 |
| CXorf40B | 0.152326723 | 0.000522971 | 0.001168601 |
| CXorf41 | -0.211045556 | 1.35E-06 | 4.64E-06 |
| CXorf42 | -0.293496766 | 1.09E-11 | 8.09E-11 |
| CXorf50B | -0.274973496 | 2.18E-10 | 1.34E-09 |
| CXorf57 | -0.260348057 | 2.00E-09 | 1.06E-08 |
| CXorf59 | -0.246528073 | 1.44E-08 | 6.72E-08 |
| CXorf61 | 0.204198068 | 2.98E-06 | 9.68E-06 |
| CXorf64 | -0.174101793 | 7.13E-05 | 0.000185646 |
| CXorf65 | -0.222282889 | 3.47E-07 | 1.31E-06 |
| CYB561D1 | -0.245029496 | 1.77E-08 | 8.15E-08 |
| CYB5A | -0.236866118 | 5.33E-08 | 2.28E-07 |
| CYB5B | 0.182604502 | 3.06E-05 | 8.46E-05 |
| CYB5D1 | -0.174007534 | 7.20E-05 | 0.000187064 |
| CYB5D2 | -0.212030623 | 1.20E-06 | 4.16E-06 |
| CYB5R1 | -0.241776881 | 2.76E-08 | 1.23E-07 |
| CYB5R2 | -0.139200526 | 0.001541703 | 0.003169346 |
| CYB5R3 | -0.272393073 | 3.26E-10 | 1.94E-09 |
| CYB5R4 | 0.152111397 | 0.000532711 | 0.001188506 |
| CYB5RL | -0.229213564 | 1.45E-07 | 5.80E-07 |
| CYBASC3 | -0.198497262 | 5.65E-06 | 1.76E-05 |
| CYBB | -0.160912318 | 0.000245672 | 0.000583819 |
| CYBRD1 | -0.378244483 | 5.82E-19 | 1.31E-17 |
| CYC1 | 0.366592155 | 7.95E-18 | 1.51E-16 |
| CYCSP52 | -0.118912154 | 0.006901079 | 0.01262779 |
| CYCS | 0.476622695 | 1.44E-30 | 1.76E-28 |
| CYFIP2 | -0.37118993 | 2.87E-18 | 5.80E-17 |
| CYGB | -0.207311855 | 2.09E-06 | 6.94E-06 |
| CYLD | -0.322692007 | 6.05E-14 | 6.32E-13 |
| CYMP | -0.187501595 | 1.85E-05 | 5.31E-05 |
| CYP17A1 | -0.22448689 | 2.63E-07 | 1.02E-06 |
| CYP1A2 | -0.183449853 | 2.81E-05 | 7.81E-05 |
| CYP20A1 | -0.261627302 | 1.66E-09 | 8.88E-09 |
| CYP21A2 | -0.399639923 | 3.60E-21 | 1.13E-19 |
| CYP24A1 | 0.307019148 | 1.05E-12 | 9.20E-12 |
| CYP27A1 | -0.365248149 | 1.07E-17 | 1.99E-16 |
| CYP2A13 | -0.174679584 | 6.74E-05 | 0.000176149 |
| CYP2A6 | -0.145693256 | 0.000913217 | 0.001954534 |
| CYP2B6 | -0.114540545 | 0.00927928 | 0.016575 |
| CYP2B7P1 | -0.375775713 | 1.02E-18 | 2.22E-17 |
| CYP2C8 | -0.174196248 | 7.07E-05 | 0.000184092 |
| CYP2C9 | -0.103955812 | 0.018284935 | 0.030720289 |
| CYP2D6 | -0.143646602 | 0.001079636 | 0.002283091 |
| CYP2D7P1 | -0.249365691 | 9.69E-09 | 4.65E-08 |
| CYP2F1 | -0.242287698 | 2.57E-08 | 1.16E-07 |
| CYP2R1 | -0.16699643 | 0.000140486 | 0.000348954 |
| CYP2S1 | -0.238285273 | 4.41E-08 | 1.91E-07 |
| CYP2U1 | -0.323135113 | 5.57E-14 | 5.84E-13 |
| CYP2W1 | -0.11029264 | 0.012262414 | 0.021348413 |
| CYP39A1 | -0.181705222 | 3.35E-05 | 9.21E-05 |
| CYP3A7 | -0.250226615 | 8.58E-09 | 4.15E-08 |
| CYP46A1 | -0.26071266 | 1.90E-09 | 1.01E-08 |
| CYP4A11 | -0.252737837 | 6.02E-09 | 2.97E-08 |
| CYP4A22 | -0.315789298 | 2.18E-13 | 2.10E-12 |
| CYP4B1 | -0.467196229 | 2.77E-29 | 2.72E-27 |
| CYP4F12 | -0.229465321 | 1.40E-07 | 5.64E-07 |
| CYP4F22 | -0.164513919 | 0.000176889 | 0.000432443 |
| CYP4F2 | 0.110923465 | 0.011771714 | 0.020562283 |
| CYP4F3 | 0.19259039 | 1.08E-05 | 3.21E-05 |
| CYP4V2 | -0.300295664 | 3.41E-12 | 2.73E-11 |
| CYP4X1 | -0.432754117 | 6.42E-25 | 3.54E-23 |
| CYP4Z1 | -0.436891062 | 2.04E-25 | 1.22E-23 |
| CYP4Z2P | -0.458587517 | 3.81E-28 | 3.16E-26 |
| CYP51A1 | 0.164027182 | 0.000184992 | 0.00045038 |
| CYP7A1 | -0.274927973 | 2.20E-10 | 1.35E-09 |
| CYP7B1 | -0.110294754 | 0.01226074 | 0.021347358 |
| CYR61 | -0.163844989 | 0.000188114 | 0.00045709 |
| CYS1 | -0.304502409 | 1.64E-12 | 1.39E-11 |
| CYSLTR1 | -0.269215104 | 5.31E-10 | 3.07E-09 |
| CYSLTR2 | -0.154155644 | 0.000446667 | 0.001010753 |
| CYTH1 | -0.366522735 | 8.07E-18 | 1.53E-16 |
| CYTH2 | -0.186110965 | 2.13E-05 | 6.07E-05 |
| CYTH3 | -0.2844605 | 4.83E-11 | 3.25E-10 |
| CYTH4 | -0.178624193 | 4.57E-05 | 0.000122801 |
| CYTIP | -0.14802846 | 0.000752447 | 0.001637799 |
| CYTSA | -0.186053204 | 2.15E-05 | 6.10E-05 |
| CYTSB | 0.17229548 | 8.50E-05 | 0.000218188 |
| CYYR1 | -0.238633125 | 4.21E-08 | 1.83E-07 |
| CYorf15A | 0.160915136 | 0.00024561 | 0.000583809 |
| CYorf15B | 0.109242956 | 0.013119054 | 0.02272499 |
| D2HGDH | -0.264159269 | 1.14E-09 | 6.27E-09 |
| D4S234E | -0.306250337 | 1.21E-12 | 1.04E-11 |
| DAAM2 | -0.3973622 | 6.30E-21 | 1.88E-19 |
| DAB2IP | -0.275826697 | 1.91E-10 | 1.18E-09 |
| DAB2 | -0.226626168 | 2.01E-07 | 7.90E-07 |
| DACH1 | -0.290765542 | 1.72E-11 | 1.24E-10 |
| DACH2 | -0.171971092 | 8.77E-05 | 0.000224874 |
| DACT2 | -0.111995904 | 0.010977257 | 0.019316262 |
| DACT3 | -0.177525682 | 5.10E-05 | 0.000135876 |
| DAD1L | -0.119594928 | 0.006583593 | 0.012093612 |
| DAD1 | 0.348488968 | 3.76E-16 | 5.54E-15 |
| DAGLB | -0.230856729 | 1.17E-07 | 4.77E-07 |
| DALRD3 | -0.1041137 | 0.018108078 | 0.030453871 |
| DAND5 | -0.106970499 | 0.015156401 | 0.025910549 |
| DAO | -0.152143609 | 0.000531243 | 0.001185629 |
| DAP3 | 0.300001686 | 3.59E-12 | 2.86E-11 |
| DAPK1 | -0.327549234 | 2.41E-14 | 2.68E-13 |
| DAPK2 | -0.496255256 | 2.26E-33 | 4.66E-31 |
| DAPP1 | -0.174400437 | 6.93E-05 | 0.00018075 |
| DARC | -0.355668756 | 8.39E-17 | 1.35E-15 |
| DARS2 | 0.471927003 | 6.36E-30 | 6.91E-28 |
| DARS | 0.422572465 | 1.01E-23 | 4.59E-22 |
| DAZAP2 | 0.110917075 | 0.011776596 | 0.02056891 |
| DBC1 | -0.234434298 | 7.35E-08 | 3.08E-07 |
| DBF4B | 0.173230721 | 7.76E-05 | 0.000200715 |
| DBF4 | 0.5230277 | 1.67E-37 | 7.61E-35 |
| DBH | -0.2924712 | 1.29E-11 | 9.50E-11 |
| DBN1 | 0.20448404 | 2.89E-06 | 9.40E-06 |
| DBNDD1 | 0.193202732 | 1.01E-05 | 3.02E-05 |
| DBNL | -0.112991856 | 0.010282388 | 0.018210559 |
| DBP | -0.368595672 | 5.11E-18 | 9.99E-17 |
| DBR1 | 0.095551569 | 0.030150777 | 0.048405922 |
| DCAF10 | 0.19275447 | 1.06E-05 | 3.16E-05 |
| DCAF11 | -0.180375354 | 3.83E-05 | 0.000104345 |
| DCAF13 | 0.479957475 | 4.96E-31 | 6.44E-29 |
| DCAF15 | -0.14750833 | 0.000785802 | 0.001702799 |
| DCAF17 | 0.178637572 | 4.56E-05 | 0.000122654 |
| DCAF4L1 | -0.098524741 | 0.025358875 | 0.041350398 |
| DCAF4L2 | 0.104168883 | 0.018046618 | 0.030358173 |
| DCAF5 | -0.195874162 | 7.54E-06 | 2.30E-05 |
| DCAF6 | -0.142949244 | 0.001142448 | 0.002405482 |
| DCAF8L2 | 0.121503725 | 0.005764339 | 0.010700444 |
| DCAF8 | -0.278561522 | 1.24E-10 | 7.89E-10 |
| DCAKD | -0.221597548 | 3.78E-07 | 1.42E-06 |
| DCC | -0.132479161 | 0.002591795 | 0.005119557 |
| DCDC2B | -0.268851653 | 5.61E-10 | 3.24E-09 |
| DCDC2 | -0.11693575 | 0.007898807 | 0.014300592 |
| DCHS1 | -0.157974671 | 0.000319532 | 0.000742048 |
| DCHS2 | -0.213507784 | 1.01E-06 | 3.54E-06 |
| DCK | 0.282902547 | 6.21E-11 | 4.12E-10 |
| DCLK2 | -0.291617937 | 1.49E-11 | 1.09E-10 |
| DCLK3 | 0.137392664 | 0.001776889 | 0.0036127 |
| DCLRE1A | 0.270895011 | 4.11E-10 | 2.42E-09 |
| DCLRE1B | 0.13590977 | 0.001993872 | 0.00402317 |
| DCN | -0.195815954 | 7.59E-06 | 2.31E-05 |
| DCP1B | -0.2290377 | 1.48E-07 | 5.93E-07 |
| DCP2 | -0.217444634 | 6.28E-07 | 2.28E-06 |
| DCPS | 0.102337756 | 0.020186053 | 0.033600835 |
| DCST2 | -0.177196879 | 5.27E-05 | 0.000139944 |
| DCTN1 | -0.169451679 | 0.000111497 | 0.000281674 |
| DCTN2 | 0.23543007 | 6.45E-08 | 2.73E-07 |
| DCTN4 | -0.097755249 | 0.026531353 | 0.043093505 |
| DCTN5 | 0.203545698 | 3.21E-06 | 1.04E-05 |
| DCTN6 | 0.12942672 | 0.003256708 | 0.006317458 |
| DCTPP1 | 0.28968582 | 2.05E-11 | 1.47E-10 |
| DCT | -0.211909496 | 1.22E-06 | 4.21E-06 |
| DCUN1D1 | 0.259860494 | 2.15E-09 | 1.13E-08 |
| DCUN1D2 | -0.202718872 | 3.53E-06 | 1.13E-05 |
| DCUN1D3 | -0.15909155 | 0.000289297 | 0.000678057 |
| DCUN1D5 | 0.449497254 | 5.59E-27 | 4.07E-25 |
| DDAH1 | -0.261519498 | 1.69E-09 | 9.02E-09 |
| DDB1 | 0.147339631 | 0.000796911 | 0.001724943 |
| DDB2 | -0.240027294 | 3.49E-08 | 1.54E-07 |
| DDC | 0.166533642 | 0.000146688 | 0.000363411 |
| DDI1 | -0.100085978 | 0.023116765 | 0.038026326 |
| DDIT3 | 0.139353756 | 0.001523145 | 0.003137004 |
| DDIT4L | 0.131481726 | 0.002794042 | 0.005485983 |
| DDIT4 | 0.105554471 | 0.016562019 | 0.028068648 |
| DDN | 0.238684842 | 4.18E-08 | 1.82E-07 |
| DDOST | 0.204540284 | 2.87E-06 | 9.34E-06 |
| DDO | -0.23914231 | 3.94E-08 | 1.72E-07 |
| DDR1 | -0.235818157 | 6.12E-08 | 2.60E-07 |
| DDR2 | -0.149316684 | 0.000675397 | 0.001482032 |
| DDRGK1 | -0.139108924 | 0.001552896 | 0.003190715 |
| DDX10 | 0.321253816 | 7.92E-14 | 8.13E-13 |
| DDX11L2 | -0.156532465 | 0.000362943 | 0.000833756 |
| DDX11 | 0.143052265 | 0.001132961 | 0.002386514 |
| DDX12 | 0.107736736 | 0.014440367 | 0.02479461 |
| DDX17 | -0.301686251 | 2.68E-12 | 2.18E-11 |
| DDX18 | 0.344464559 | 8.58E-16 | 1.19E-14 |
| DDX1 | 0.31845277 | 1.33E-13 | 1.33E-12 |
| DDX20 | 0.102337783 | 0.02018602 | 0.033600835 |
| DDX21 | 0.390618646 | 3.22E-20 | 8.67E-19 |
| DDX23 | 0.288113548 | 2.66E-11 | 1.86E-10 |
| DDX24 | -0.178869799 | 4.46E-05 | 0.000120006 |
| DDX26B | -0.297867108 | 5.18E-12 | 4.03E-11 |
| DDX27 | 0.170456908 | 0.000101337 | 0.000257667 |
| DDX39 | 0.336010299 | 4.66E-15 | 5.82E-14 |
| DDX3Y | 0.155522939 | 0.000396537 | 0.000904586 |
| DDX42 | -0.218280566 | 5.67E-07 | 2.08E-06 |
| DDX47 | 0.401653781 | 2.19E-21 | 7.10E-20 |
| DDX49 | 0.107115442 | 0.015018611 | 0.025718985 |
| DDX50 | 0.287957582 | 2.73E-11 | 1.91E-10 |
| DDX52 | 0.361065774 | 2.65E-17 | 4.64E-16 |
| DDX53 | 0.14280632 | 0.00115573 | 0.002430887 |
| DDX54 | 0.145038789 | 0.000963662 | 0.002055017 |
| DDX55 | 0.241039407 | 3.05E-08 | 1.35E-07 |
| DDX56 | 0.263618691 | 1.23E-09 | 6.74E-09 |
| DDX59 | 0.146200692 | 0.000875797 | 0.001880485 |
| DDX5 | -0.322361887 | 6.44E-14 | 6.71E-13 |
| DEAF1 | -0.18995484 | 1.43E-05 | 4.18E-05 |
| DECR1 | 0.19664674 | 6.93E-06 | 2.12E-05 |
| DEDD | 0.171247768 | 9.40E-05 | 0.000240078 |
| DEF6 | -0.308480782 | 8.14E-13 | 7.23E-12 |
| DEF8 | -0.121755783 | 0.005663303 | 0.010532994 |
| DEFA5 | 0.121516749 | 0.005759078 | 0.010694208 |
| DEFB103B | 0.191553193 | 1.20E-05 | 3.56E-05 |
| DEFB124 | -0.135264611 | 0.002095635 | 0.004213402 |
| DEFB126 | 0.120263977 | 0.006285258 | 0.011587321 |
| DEFB1 | 0.192217828 | 1.12E-05 | 3.33E-05 |
| DEGS2 | -0.157063389 | 0.00034636 | 0.000798873 |
| DEK | 0.265082131 | 9.92E-10 | 5.53E-09 |
| DENND1A | 0.149796748 | 0.000648604 | 0.001426686 |
| DENND1C | -0.344355521 | 8.77E-16 | 1.22E-14 |
| DENND2A | -0.31053147 | 5.64E-13 | 5.13E-12 |
| DENND2D | -0.174648212 | 6.76E-05 | 0.00017662 |
| DENND3 | -0.33811502 | 3.07E-15 | 3.97E-14 |
| DENND4B | -0.13842284 | 0.00163913 | 0.003352735 |
| DENND4C | -0.112745596 | 0.010450468 | 0.018475495 |
| DENND5A | -0.110214031 | 0.01232481 | 0.021445835 |
| DENR | 0.495073104 | 3.38E-33 | 6.75E-31 |
| DEPDC1B | 0.495981588 | 2.48E-33 | 5.01E-31 |
| DEPDC1 | 0.572998306 | 2.84E-46 | 6.30E-43 |
| DEPDC4 | 0.146912943 | 0.000825661 | 0.0017826 |
| DEPDC5 | -0.228204697 | 1.65E-07 | 6.54E-07 |
| DEPDC6 | -0.200186655 | 4.69E-06 | 1.48E-05 |
| DERA | 0.36802288 | 5.80E-18 | 1.13E-16 |
| DERL1 | 0.32096433 | 8.36E-14 | 8.55E-13 |
| DERL2 | 0.185022384 | 2.39E-05 | 6.72E-05 |
| DES | -0.334298489 | 6.53E-15 | 7.97E-14 |
| DET1 | -0.153393055 | 0.000477128 | 0.001073243 |
| DEXI | -0.251312747 | 7.36E-09 | 3.60E-08 |
| DFFA | 0.270222666 | 4.55E-10 | 2.66E-09 |
| DFFB | -0.115718551 | 0.008575573 | 0.015397821 |
| DFNA5 | -0.11269749 | 0.010483585 | 0.018527488 |
| DFNB31 | -0.266921922 | 7.52E-10 | 4.26E-09 |
| DFNB59 | -0.237484878 | 4.91E-08 | 2.11E-07 |
| DGAT1 | -0.106101087 | 0.016006438 | 0.027200606 |
| DGCR10 | -0.156262075 | 0.000371671 | 0.000851749 |
| DGCR14 | -0.117985386 | 0.007353947 | 0.013394449 |
| DGCR2 | -0.254042293 | 5.00E-09 | 2.49E-08 |
| DGCR6L | -0.173316179 | 7.70E-05 | 0.000199215 |
| DGCR6 | -0.223723548 | 2.90E-07 | 1.11E-06 |
| DGCR8 | -0.187301855 | 1.88E-05 | 5.41E-05 |
| DGCR9 | -0.131219731 | 0.002849497 | 0.005584993 |
| DGKA | -0.156365307 | 0.000368316 | 0.000844933 |
| DGKD | -0.223372933 | 3.03E-07 | 1.16E-06 |
| DGKE | -0.115249442 | 0.008849895 | 0.015854773 |
| DGKG | 0.122014877 | 0.005561109 | 0.010361246 |
| DGKI | 0.170689915 | 9.91E-05 | 0.000252389 |
| DGKQ | -0.149530341 | 0.000663348 | 0.001457514 |
| DGUOK | 0.247579417 | 1.24E-08 | 5.86E-08 |
| DHCR24 | -0.19866173 | 5.55E-06 | 1.73E-05 |
| DHCR7 | 0.184710672 | 2.47E-05 | 6.93E-05 |
| DHDDS | -0.144532326 | 0.001004451 | 0.002135848 |
| DHDH | -0.260587501 | 1.93E-09 | 1.02E-08 |
| DHFRL1 | -0.152804753 | 0.000501936 | 0.001124615 |
| DHFR | 0.229248669 | 1.44E-07 | 5.78E-07 |
| DHH | -0.216073508 | 7.41E-07 | 2.66E-06 |
| DHRS12 | -0.26814101 | 6.25E-10 | 3.59E-09 |
| DHRS1 | -0.294494518 | 9.19E-12 | 6.92E-11 |
| DHRS3 | -0.249974528 | 8.89E-09 | 4.29E-08 |
| DHRS7B | -0.135002473 | 0.002138322 | 0.004293399 |
| DHRS9 | -0.104365454 | 0.017829167 | 0.03001511 |
| DHRSX | 0.14340552 | 0.001100981 | 0.002324785 |
| DHTKD1 | 0.117212668 | 0.007751666 | 0.014050994 |
| DHX15 | 0.226538208 | 2.03E-07 | 7.99E-07 |
| DHX33 | 0.182984231 | 2.94E-05 | 8.17E-05 |
| DHX36 | 0.295955584 | 7.18E-12 | 5.50E-11 |
| DHX37 | 0.242445342 | 2.52E-08 | 1.14E-07 |
| DHX40 | -0.110857815 | 0.011821959 | 0.020640925 |
| DHX57 | 0.16200083 | 0.000222615 | 0.000534042 |
| DHX58 | -0.343728219 | 9.96E-16 | 1.37E-14 |
| DHX9 | 0.185214702 | 2.34E-05 | 6.60E-05 |
| DIABLO | 0.364022039 | 1.40E-17 | 2.55E-16 |
| DIAPH2 | -0.127316787 | 0.003803165 | 0.007284874 |
| DIAPH3 | 0.438154702 | 1.43E-25 | 8.77E-24 |
| DIDO1 | -0.26010969 | 2.07E-09 | 1.09E-08 |
| DIMT1L | 0.232620574 | 9.32E-08 | 3.85E-07 |
| DIO1 | -0.216996509 | 6.63E-07 | 2.40E-06 |
| DIO2 | 0.18873721 | 1.62E-05 | 4.70E-05 |
| DIO3OS | -0.246403351 | 1.46E-08 | 6.83E-08 |
| DIO3 | -0.181290523 | 3.50E-05 | 9.58E-05 |
| DIP2A | -0.267447599 | 6.95E-10 | 3.96E-09 |
| DIP2B | 0.192174565 | 1.13E-05 | 3.34E-05 |
| DIP2C | -0.166165776 | 0.000151801 | 0.000374916 |
| DIRAS3 | -0.325893477 | 3.30E-14 | 3.62E-13 |
| DIRC1 | 0.172857089 | 8.05E-05 | 0.000207589 |
| DIS3L2 | -0.151154563 | 0.000578066 | 0.001281684 |
| DIS3L | -0.121364164 | 0.005820975 | 0.010795106 |
| DIS3 | 0.09532672 | 0.030542822 | 0.048937068 |
| DISC1 | -0.150936062 | 0.000588914 | 0.001304 |
| DISP1 | -0.365300583 | 1.06E-17 | 1.97E-16 |
| DIXDC1 | -0.195763855 | 7.64E-06 | 2.32E-05 |
| DKC1 | 0.470635191 | 9.53E-30 | 1.00E-27 |
| DKFZP586I1420 | -0.263965532 | 1.17E-09 | 6.44E-09 |
| DKFZP686I15217 | -0.235742452 | 6.19E-08 | 2.62E-07 |
| DKFZp686A1627 | 0.113348771 | 0.010043041 | 0.017826152 |
| DKFZp761E198 | -0.112273843 | 0.010779273 | 0.019008038 |
| DKK1 | 0.144207623 | 0.001031434 | 0.002188799 |
| DKK2 | -0.152935826 | 0.000496307 | 0.001112752 |
| DKK3 | -0.167608106 | 0.000132666 | 0.000330721 |
| DLAT | 0.357898787 | 5.23E-17 | 8.74E-16 |
| DLC1 | -0.425266305 | 4.92E-24 | 2.37E-22 |
| DLD | 0.298837614 | 4.39E-12 | 3.44E-11 |
| DLEC1 | -0.427565266 | 2.65E-24 | 1.34E-22 |
| DLEU1 | 0.247603128 | 1.24E-08 | 5.84E-08 |
| DLEU2L | -0.164309832 | 0.000180245 | 0.000439895 |
| DLG2 | -0.112482861 | 0.010632476 | 0.018765733 |
| DLG3 | -0.151145044 | 0.000578535 | 0.001282581 |
| DLG4 | -0.2148008 | 8.64E-07 | 3.06E-06 |
| DLGAP1 | -0.161368392 | 0.000235752 | 0.000562317 |
| DLGAP2 | -0.265960819 | 8.69E-10 | 4.88E-09 |
| DLGAP3 | -0.115104352 | 0.008936309 | 0.016000981 |
| DLGAP5 | 0.598126416 | 2.78E-51 | 1.11E-47 |
| DLK1 | -0.097357161 | 0.027156041 | 0.04399732 |
| DLL1 | -0.155315256 | 0.000403798 | 0.000919889 |
| DLL3 | 0.229996546 | 1.31E-07 | 5.30E-07 |
| DLST | 0.225172394 | 2.42E-07 | 9.36E-07 |
| DLX2 | 0.144246806 | 0.001028143 | 0.002182974 |
| DLX3 | -0.302840561 | 2.20E-12 | 1.82E-11 |
| DLX4 | -0.196134574 | 7.33E-06 | 2.24E-05 |
| DMAP1 | -0.213246609 | 1.04E-06 | 3.64E-06 |
| DMBT1 | -0.307699646 | 9.35E-13 | 8.23E-12 |
| DMC1 | 0.148912085 | 0.000698771 | 0.001529795 |
| DMD | -0.260608617 | 1.93E-09 | 1.02E-08 |
| DMKN | -0.12701919 | 0.003886591 | 0.007435412 |
| DMP1 | 0.105048492 | 0.017091274 | 0.028879903 |
| DMPK | -0.115521335 | 0.008689963 | 0.01558781 |
| DMRT2 | -0.275827123 | 1.91E-10 | 1.18E-09 |
| DMRTA1 | -0.151050132 | 0.000583227 | 0.001292125 |
| DMRTA2 | -0.102821212 | 0.019600775 | 0.032730183 |
| DMRTC1B | -0.312713157 | 3.81E-13 | 3.55E-12 |
| DMRTC1 | -0.135091443 | 0.002123746 | 0.004267132 |
| DMTF1 | -0.227988286 | 1.69E-07 | 6.71E-07 |
| DMXL1 | -0.304866784 | 1.54E-12 | 1.31E-11 |
| DMXL2 | -0.140131172 | 0.001432114 | 0.002961421 |
| DNA2 | 0.393030552 | 1.80E-20 | 5.01E-19 |
| DNAH10 | -0.26690835 | 7.54E-10 | 4.27E-09 |
| DNAH11 | -0.282589301 | 6.53E-11 | 4.32E-10 |
| DNAH12 | -0.286251776 | 3.61E-11 | 2.48E-10 |
| DNAH14 | 0.217317722 | 6.38E-07 | 2.31E-06 |
| DNAH17 | -0.149620912 | 0.000658301 | 0.00144722 |
| DNAH1 | -0.385182104 | 1.16E-19 | 2.91E-18 |
| DNAH2 | -0.244601111 | 1.88E-08 | 8.62E-08 |
| DNAH3 | -0.125273233 | 0.004410313 | 0.008360522 |
| DNAH5 | -0.239969872 | 3.52E-08 | 1.55E-07 |
| DNAH6 | -0.374981697 | 1.22E-18 | 2.63E-17 |
| DNAH7 | -0.289402899 | 2.15E-11 | 1.53E-10 |
| DNAH8 | -0.258283834 | 2.71E-09 | 1.40E-08 |
| DNAH9 | -0.274133537 | 2.49E-10 | 1.51E-09 |
| DNAI1 | -0.218844373 | 5.30E-07 | 1.95E-06 |
| DNAI2 | -0.265042845 | 9.98E-10 | 5.55E-09 |
| DNAJA1 | 0.347314494 | 4.79E-16 | 6.96E-15 |
| DNAJA2 | 0.155019871 | 0.000414338 | 0.000942509 |
| DNAJA3 | 0.175745896 | 6.08E-05 | 0.000159716 |
| DNAJA4 | -0.128915619 | 0.003382099 | 0.006540383 |
| DNAJB11 | 0.26082205 | 1.87E-09 | 9.91E-09 |
| DNAJB12 | -0.102563136 | 0.01991134 | 0.03318778 |
| DNAJB13 | -0.202194177 | 3.74E-06 | 1.20E-05 |
| DNAJB2 | -0.266491221 | 8.03E-10 | 4.53E-09 |
| DNAJB3 | 0.102436303 | 0.020065531 | 0.033428058 |
| DNAJB4 | 0.227392405 | 1.83E-07 | 7.21E-07 |
| DNAJB6 | 0.186006353 | 2.16E-05 | 6.12E-05 |
| DNAJB7 | -0.129948242 | 0.003133128 | 0.00609726 |
| DNAJC10 | 0.353118706 | 1.44E-16 | 2.23E-15 |
| DNAJC12 | 0.135440608 | 0.002067413 | 0.004162314 |
| DNAJC14 | 0.28700027 | 3.19E-11 | 2.21E-10 |
| DNAJC15 | 0.133228047 | 0.002448819 | 0.004859227 |
| DNAJC17 | -0.151108353 | 0.000580345 | 0.001286165 |
| DNAJC18 | -0.096520953 | 0.02850956 | 0.045985239 |
| DNAJC1 | 0.156085669 | 0.00037747 | 0.000863652 |
| DNAJC22 | 0.193033134 | 1.03E-05 | 3.07E-05 |
| DNAJC25-GNG10 | 0.109090284 | 0.013247948 | 0.022910537 |
| DNAJC25 | 0.173626802 | 7.47E-05 | 0.000193697 |
| DNAJC27 | -0.344556145 | 8.42E-16 | 1.18E-14 |
| DNAJC28 | -0.282881283 | 6.23E-11 | 4.13E-10 |
| DNAJC2 | 0.278493949 | 1.26E-10 | 7.97E-10 |
| DNAJC4 | -0.212218862 | 1.18E-06 | 4.07E-06 |
| DNAJC5B | -0.141663397 | 0.001267173 | 0.002646906 |
| DNAJC5G | -0.123124784 | 0.005141856 | 0.00964303 |
| DNAJC6 | 0.102048784 | 0.020543097 | 0.034158175 |
| DNAJC7 | 0.12260637 | 0.005334001 | 0.009971568 |
| DNAJC9 | 0.423435455 | 8.03E-24 | 3.72E-22 |
| DNAL4 | -0.130861454 | 0.002926955 | 0.005726756 |
| DNALI1 | -0.268228642 | 6.17E-10 | 3.54E-09 |
| DNASE1L3 | -0.281582829 | 7.68E-11 | 5.03E-10 |
| DNASE2B | -0.27972069 | 1.03E-10 | 6.65E-10 |
| DNASE2 | -0.189801856 | 1.45E-05 | 4.24E-05 |
| DND1 | -0.144669351 | 0.000993261 | 0.002113402 |
| DNER | 0.158328457 | 0.00030965 | 0.000721277 |
| DNHD1 | -0.326372789 | 3.02E-14 | 3.31E-13 |
| DNM1L | 0.365191479 | 1.08E-17 | 2.01E-16 |
| DNM1P35 | -0.369268382 | 4.40E-18 | 8.69E-17 |
| DNM2 | -0.28339946 | 5.73E-11 | 3.82E-10 |
| DNM3 | -0.214473797 | 8.99E-07 | 3.18E-06 |
| DNMT3A | 0.163318658 | 0.000197412 | 0.000478287 |
| DNMT3B | 0.289067998 | 2.27E-11 | 1.61E-10 |
| DNTTIP1 | 0.135728988 | 0.002021922 | 0.004076475 |
| DNTTIP2 | 0.321173526 | 8.04E-14 | 8.24E-13 |
| DOC2A | -0.19153877 | 1.20E-05 | 3.57E-05 |
| DOCK10 | -0.294101441 | 9.82E-12 | 7.35E-11 |
| DOCK11 | -0.160118483 | 0.000263876 | 0.000623225 |
| DOCK1 | -0.153640217 | 0.00046705 | 0.001052469 |
| DOCK2 | -0.272450933 | 3.23E-10 | 1.93E-09 |
| DOCK3 | -0.151905473 | 0.000542183 | 0.001207392 |
| DOCK4 | -0.23702823 | 5.22E-08 | 2.24E-07 |
| DOCK6 | -0.177630437 | 5.04E-05 | 0.00013458 |
| DOCK7 | 0.151134961 | 0.000579031 | 0.00128354 |
| DOCK8 | -0.336385315 | 4.33E-15 | 5.44E-14 |
| DOCK9 | -0.346304844 | 5.89E-16 | 8.44E-15 |
| DOK1 | -0.30819437 | 8.56E-13 | 7.57E-12 |
| DOK2 | -0.23843265 | 4.33E-08 | 1.88E-07 |
| DOK3 | -0.203929429 | 3.07E-06 | 9.96E-06 |
| DOK4 | -0.326354321 | 3.03E-14 | 3.32E-13 |
| DOK5 | -0.098946294 | 0.024735713 | 0.040423304 |
| DOK6 | -0.195189759 | 8.13E-06 | 2.47E-05 |
| DOK7 | -0.218756344 | 5.35E-07 | 1.97E-06 |
| DOLK | 0.107191014 | 0.014947205 | 0.025607674 |
| DOLPP1 | 0.157694179 | 0.000327576 | 0.000759228 |
| DOM3Z | -0.107933253 | 0.014261588 | 0.024525563 |
| DONSON | 0.27681989 | 1.64E-10 | 1.02E-09 |
| DOPEY1 | -0.304599985 | 1.62E-12 | 1.37E-11 |
| DOT1L | -0.108460763 | 0.013791284 | 0.023782261 |
| DPAGT1 | 0.141277814 | 0.001306947 | 0.002722307 |
| DPCD | 0.134567339 | 0.00221094 | 0.00442365 |
| DPCR1 | -0.319635097 | 1.07E-13 | 1.08E-12 |
| DPEP2 | -0.381010467 | 3.08E-19 | 7.25E-18 |
| DPF1 | 0.097946367 | 0.02623587 | 0.042665574 |
| DPF3 | -0.102038397 | 0.020556033 | 0.034176841 |
| DPH1 | -0.25866303 | 2.56E-09 | 1.33E-08 |
| DPH2 | 0.210245693 | 1.48E-06 | 5.05E-06 |
| DPH3 | 0.218024204 | 5.85E-07 | 2.14E-06 |
| DPH5 | 0.225063124 | 2.45E-07 | 9.48E-07 |
| DPM1 | 0.275105759 | 2.14E-10 | 1.31E-09 |
| DPP10 | -0.212221258 | 1.17E-06 | 4.07E-06 |
| DPP3 | 0.238156998 | 4.49E-08 | 1.95E-07 |
| DPP4 | -0.225866156 | 2.21E-07 | 8.63E-07 |
| DPP6 | -0.25808137 | 2.79E-09 | 1.44E-08 |
| DPP7 | -0.261896703 | 1.59E-09 | 8.56E-09 |
| DPPA2 | 0.142573069 | 0.001177712 | 0.002473479 |
| DPRXP4 | -0.221212652 | 3.96E-07 | 1.48E-06 |
| DPRX | -0.097839305 | 0.026401045 | 0.042902772 |
| DPT | -0.101617588 | 0.021086076 | 0.034985349 |
| DPY19L1 | 0.143940452 | 0.001054136 | 0.002231762 |
| DPY19L2P2 | -0.207264339 | 2.10E-06 | 6.98E-06 |
| DPY19L2P4 | -0.178284597 | 4.73E-05 | 0.000126669 |
| DPY19L3 | -0.147404341 | 0.000792633 | 0.00171667 |
| DPY19L4 | 0.215987268 | 7.49E-07 | 2.68E-06 |
| DPY30 | 0.222363301 | 3.43E-07 | 1.30E-06 |
| DPYD | -0.209950194 | 1.53E-06 | 5.22E-06 |
| DPYSL2 | -0.479680503 | 5.42E-31 | 6.99E-29 |
| DPYS | -0.183446584 | 2.81E-05 | 7.81E-05 |
| DR1 | 0.234207414 | 7.57E-08 | 3.17E-07 |
| DRAM1 | -0.270856269 | 4.13E-10 | 2.43E-09 |
| DRAP1 | 0.166504497 | 0.000147087 | 0.00036431 |
| DRD1 | -0.2358176 | 6.13E-08 | 2.60E-07 |
| DRD2 | -0.141256949 | 0.001309132 | 0.002726005 |
| DRD5 | -0.208092182 | 1.91E-06 | 6.40E-06 |
| DRG1 | 0.336051412 | 4.62E-15 | 5.78E-14 |
| DRGX | -0.143450723 | 0.00109695 | 0.002317251 |
| DRP2 | 0.274335636 | 2.41E-10 | 1.47E-09 |
| DSC2 | 0.189208922 | 1.54E-05 | 4.49E-05 |
| DSCAML1 | -0.23134156 | 1.10E-07 | 4.51E-07 |
| DSCAM | 0.177977297 | 4.87E-05 | 0.000130338 |
| DSCC1 | 0.515632656 | 2.52E-36 | 9.33E-34 |
| DSCR3 | -0.131712912 | 0.002745926 | 0.00540159 |
| DSCR8 | 0.10606131 | 0.016046309 | 0.027259359 |
| DSG1 | 0.156292795 | 0.00037067 | 0.000849747 |
| DSG2 | 0.286740991 | 3.33E-11 | 2.30E-10 |
| DSG4 | 0.205623855 | 2.53E-06 | 8.32E-06 |
| DSN1 | 0.270360399 | 4.46E-10 | 2.61E-09 |
| DSP | 0.228347631 | 1.62E-07 | 6.43E-07 |
| DSTYK | -0.17457673 | 6.81E-05 | 0.00017781 |
| DST | -0.15056726 | 0.000607654 | 0.00134252 |
| DTD1 | 0.183472014 | 2.80E-05 | 7.80E-05 |
| DTHD1 | -0.238423825 | 4.33E-08 | 1.88E-07 |
| DTL | 0.450687816 | 3.95E-27 | 2.96E-25 |
| DTNA | -0.097894255 | 0.026316157 | 0.042775259 |
| DTNBP1 | -0.216653546 | 6.91E-07 | 2.49E-06 |
| DTNB | -0.203389592 | 3.27E-06 | 1.05E-05 |
| DTX1 | -0.106677737 | 0.015438103 | 0.026333573 |
| DTX3 | -0.327067124 | 2.64E-14 | 2.93E-13 |
| DTX4 | -0.318178824 | 1.40E-13 | 1.39E-12 |
| DTYMK | 0.401147208 | 2.48E-21 | 7.99E-20 |
| DUOX1 | -0.361922425 | 2.20E-17 | 3.88E-16 |
| DUOX2 | -0.19271977 | 1.06E-05 | 3.17E-05 |
| DUOXA1 | -0.363964526 | 1.41E-17 | 2.57E-16 |
| DUOXA2 | -0.206873763 | 2.19E-06 | 7.28E-06 |
| DUPD1 | -0.097600673 | 0.026772432 | 0.043470949 |
| DUS4L | 0.301678956 | 2.69E-12 | 2.18E-11 |
| DUSP11 | 0.295714262 | 7.48E-12 | 5.72E-11 |
| DUSP12 | 0.153937358 | 0.000455196 | 0.001028307 |
| DUSP13 | 0.277253286 | 1.53E-10 | 9.57E-10 |
| DUSP14 | 0.289445394 | 2.14E-11 | 1.52E-10 |
| DUSP15 | -0.223935027 | 2.82E-07 | 1.08E-06 |
| DUSP18 | -0.206782036 | 2.22E-06 | 7.35E-06 |
| DUSP19 | -0.152645981 | 0.000508833 | 0.001139048 |
| DUSP1 | -0.199464197 | 5.08E-06 | 1.59E-05 |
| DUSP22 | -0.243187777 | 2.28E-08 | 1.03E-07 |
| DUSP26 | -0.351143848 | 2.17E-16 | 3.28E-15 |
| DUSP27 | -0.293093914 | 1.16E-11 | 8.61E-11 |
| DUSP28 | -0.212880638 | 1.09E-06 | 3.79E-06 |
| DUSP2 | -0.140211496 | 0.001422999 | 0.002944096 |
| DUSP3 | -0.129958517 | 0.003130736 | 0.006093199 |
| DUSP4 | 0.265281021 | 9.63E-10 | 5.37E-09 |
| DUSP5P | 0.133145545 | 0.00246421 | 0.004886856 |
| DUSP6 | -0.113557722 | 0.009905226 | 0.017609681 |
| DUSP7 | -0.12775454 | 0.003683394 | 0.007072399 |
| DUSP8 | -0.249269017 | 9.82E-09 | 4.71E-08 |
| DUT | 0.219228656 | 5.05E-07 | 1.87E-06 |
| DUXA | -0.095777936 | 0.029760451 | 0.047836904 |
| DVL2 | 0.096485682 | 0.0285679 | 0.046071905 |
| DVWA | 0.166486753 | 0.000147331 | 0.000364867 |
| DYDC1 | -0.14254176 | 0.001180691 | 0.002479216 |
| DYM | 0.187319172 | 1.88E-05 | 5.40E-05 |
| DYNC1I2 | 0.279857504 | 1.01E-10 | 6.52E-10 |
| DYNC1LI1 | 0.18557049 | 2.26E-05 | 6.38E-05 |
| DYNC1LI2 | -0.191571694 | 1.20E-05 | 3.55E-05 |
| DYNC2H1 | -0.335731161 | 4.93E-15 | 6.12E-14 |
| DYNC2LI1 | -0.189655517 | 1.47E-05 | 4.30E-05 |
| DYNLL1 | 0.46116841 | 1.75E-28 | 1.55E-26 |
| DYNLL2 | -0.203391624 | 3.27E-06 | 1.05E-05 |
| DYNLRB2 | -0.242446344 | 2.52E-08 | 1.14E-07 |
| DYNLT1 | 0.107704707 | 0.014469691 | 0.024838559 |
| DYNLT3 | 0.115854492 | 0.008497507 | 0.015272742 |
| DYRK1A | -0.345836913 | 6.48E-16 | 9.20E-15 |
| DYRK1B | -0.142957893 | 0.001141648 | 0.002404053 |
| DYSFIP1 | 0.097231409 | 0.027355986 | 0.044292544 |
| DZIP1L | -0.193136159 | 1.02E-05 | 3.04E-05 |
| DZIP3 | -0.220676089 | 4.23E-07 | 1.58E-06 |
| E2F1 | 0.257041663 | 3.24E-09 | 1.65E-08 |
| E2F2 | 0.289908397 | 1.98E-11 | 1.42E-10 |
| E2F3 | 0.266492688 | 8.02E-10 | 4.53E-09 |
| E2F4 | 0.119233141 | 0.006750152 | 0.012378169 |
| E2F5 | 0.348600068 | 3.68E-16 | 5.42E-15 |
| E2F6 | 0.233203773 | 8.63E-08 | 3.59E-07 |
| E2F7 | 0.336741695 | 4.03E-15 | 5.09E-14 |
| E2F8 | 0.358804906 | 4.31E-17 | 7.28E-16 |
| E4F1 | -0.132932556 | 0.002504351 | 0.004960556 |
| EAF1 | 0.165408302 | 0.000162859 | 0.000400151 |
| EAPP | 0.121546846 | 0.005746939 | 0.01067365 |
| EBAG9 | 0.123018499 | 0.005180733 | 0.009705923 |
| EBF1 | -0.133358001 | 0.002424754 | 0.004815299 |
| EBF2 | -0.139286738 | 0.001531236 | 0.003149774 |
| EBF3 | -0.203572223 | 3.20E-06 | 1.03E-05 |
| EBF4 | -0.413039269 | 1.23E-22 | 4.73E-21 |
| EBI3 | -0.147998057 | 0.00075436 | 0.001640891 |
| EBNA1BP2 | 0.320058692 | 9.90E-14 | 1.00E-12 |
| EBPL | 0.211805637 | 1.23E-06 | 4.26E-06 |
| EBP | 0.188348203 | 1.69E-05 | 4.89E-05 |
| ECD | 0.185829895 | 2.20E-05 | 6.23E-05 |
| ECE1 | -0.09512771 | 0.030893446 | 0.049439407 |
| ECE2 | 0.425628501 | 4.46E-24 | 2.16E-22 |
| ECHDC1 | -0.254265569 | 4.84E-09 | 2.42E-08 |
| ECHDC2 | -0.443370676 | 3.27E-26 | 2.19E-24 |
| ECHDC3 | -0.119779837 | 0.006499896 | 0.011947758 |
| ECHS1 | 0.168645031 | 0.000120335 | 0.000302168 |
| ECM1 | -0.14218353 | 0.001215283 | 0.0025461 |
| ECM2 | -0.170341726 | 0.000102455 | 0.000260378 |
| ECSCR | -0.128290068 | 0.003541527 | 0.006822943 |
| ECT2L | -0.216183974 | 7.32E-07 | 2.62E-06 |
| ECT2 | 0.514063159 | 4.45E-36 | 1.53E-33 |
| EDA2R | -0.276126853 | 1.82E-10 | 1.13E-09 |
| EDARADD | 0.242368137 | 2.55E-08 | 1.15E-07 |
| EDA | -0.267196339 | 7.22E-10 | 4.10E-09 |
| EDC3 | 0.153442063 | 0.000475114 | 0.001068953 |
| EDC4 | -0.198165521 | 5.87E-06 | 1.82E-05 |
| EDDM3B | -0.129019517 | 0.00335626 | 0.006497959 |
| EDEM1 | -0.157965798 | 0.000319784 | 0.000742546 |
| EDEM3 | 0.111089909 | 0.011645173 | 0.020374032 |
| EDN1 | -0.115101525 | 0.008938 | 0.016002575 |
| EDN3 | -0.305834757 | 1.30E-12 | 1.12E-11 |
| EDNRB | -0.26701154 | 7.42E-10 | 4.21E-09 |
| EED | 0.266502661 | 8.01E-10 | 4.52E-09 |
| EEF1B2 | 0.205397022 | 2.60E-06 | 8.52E-06 |
| EEF1E1 | 0.445659452 | 1.70E-26 | 1.17E-24 |
| EEF1G | 0.216397136 | 7.13E-07 | 2.56E-06 |
| EEF2K | -0.214170322 | 9.32E-07 | 3.29E-06 |
| EEFSEC | -0.141327476 | 0.00130176 | 0.002712752 |
| EFCAB10 | -0.14588976 | 0.000898555 | 0.001925006 |
| EFCAB1 | -0.22636006 | 2.08E-07 | 8.15E-07 |
| EFCAB3 | 0.099765867 | 0.023561952 | 0.038672712 |
| EFCAB4A | -0.182141761 | 3.21E-05 | 8.83E-05 |
| EFCAB4B | 0.100210296 | 0.022945845 | 0.037770036 |
| EFCAB5 | -0.176998364 | 5.37E-05 | 0.000142397 |
| EFCAB6 | -0.42462894 | 5.84E-24 | 2.78E-22 |
| EFEMP1 | -0.285452249 | 4.11E-11 | 2.80E-10 |
| EFEMP2 | -0.148135129 | 0.00074577 | 0.001624505 |
| EFHA1 | 0.129107389 | 0.003334547 | 0.006460926 |
| EFHA2 | -0.288969126 | 2.31E-11 | 1.63E-10 |
| EFHB | -0.266726713 | 7.75E-10 | 4.38E-09 |
| EFHC1 | -0.296070784 | 7.04E-12 | 5.39E-11 |
| EFHC2 | -0.279116955 | 1.14E-10 | 7.25E-10 |
| EFHD1 | -0.164021754 | 0.000185084 | 0.00045055 |
| EFNA2 | 0.17481013 | 6.66E-05 | 0.000174078 |
| EFNA3 | 0.176373956 | 5.71E-05 | 0.000150646 |
| EFNB1 | -0.158585813 | 0.000302641 | 0.000706268 |
| EFNB3 | -0.112035244 | 0.010949039 | 0.01927679 |
| EFR3A | 0.103130273 | 0.019234415 | 0.032169483 |
| EFR3B | -0.109068353 | 0.013266554 | 0.022940729 |
| EFS | -0.194832574 | 8.45E-06 | 2.56E-05 |
| EFTUD2 | 0.2913242 | 1.56E-11 | 1.14E-10 |
| EGFL6 | -0.110165256 | 0.012363667 | 0.021509702 |
| EGFL8 | -0.111161242 | 0.011591311 | 0.020291393 |
| EGFLAM | 0.133012365 | 0.00248924 | 0.004931602 |
| EGFR | -0.109413025 | 0.012976778 | 0.022488281 |
| EGLN1 | 0.308327713 | 8.36E-13 | 7.41E-12 |
| EGLN2 | -0.242409766 | 2.53E-08 | 1.14E-07 |
| EGLN3 | 0.291320748 | 1.56E-11 | 1.14E-10 |
| EGOT | -0.214415487 | 9.05E-07 | 3.20E-06 |
| EGR1 | -0.243776386 | 2.10E-08 | 9.57E-08 |
| EGR2 | -0.201833193 | 3.90E-06 | 1.24E-05 |
| EGR3 | -0.24281627 | 2.40E-08 | 1.08E-07 |
| EHBP1L1 | -0.205693631 | 2.51E-06 | 8.27E-06 |
| EHBP1 | 0.293136528 | 1.15E-11 | 8.55E-11 |
| EHD2 | -0.274307051 | 2.42E-10 | 1.48E-09 |
| EHD4 | -0.110333895 | 0.012229779 | 0.021299018 |
| EHF | -0.194798797 | 8.48E-06 | 2.57E-05 |
| EI24 | 0.190503247 | 1.35E-05 | 3.95E-05 |
| EID1 | -0.123410337 | 0.005038703 | 0.009461118 |
| EID2B | -0.233794506 | 7.99E-08 | 3.34E-07 |
| EID3 | 0.119331805 | 0.006704359 | 0.012298709 |
| EIF1AD | 0.211212081 | 1.32E-06 | 4.55E-06 |
| EIF1AX | 0.115739206 | 0.00856367 | 0.015379213 |
| EIF1AY | 0.158700028 | 0.000299578 | 0.000699611 |
| EIF1 | 0.141070884 | 0.001328763 | 0.002763142 |
| EIF2AK1 | 0.248416885 | 1.11E-08 | 5.25E-08 |
| EIF2AK2 | 0.231360635 | 1.10E-07 | 4.50E-07 |
| EIF2AK3 | 0.113121335 | 0.01019498 | 0.018068564 |
| EIF2AK4 | 0.108441171 | 0.013808505 | 0.023805796 |
| EIF2A | 0.220263887 | 4.45E-07 | 1.66E-06 |
| EIF2B1 | 0.29899457 | 4.27E-12 | 3.36E-11 |
| EIF2B2 | 0.321536884 | 7.51E-14 | 7.76E-13 |
| EIF2B3 | 0.195440036 | 7.91E-06 | 2.40E-05 |
| EIF2C1 | -0.250983519 | 7.72E-09 | 3.76E-08 |
| EIF2C2 | 0.340009835 | 2.11E-15 | 2.78E-14 |
| EIF2C4 | -0.367287054 | 6.82E-18 | 1.31E-16 |
| EIF2S1 | 0.538536446 | 4.55E-40 | 3.95E-37 |
| EIF2S2 | 0.431001608 | 1.04E-24 | 5.49E-23 |
| EIF2S3 | 0.242081356 | 2.65E-08 | 1.19E-07 |
| EIF3A | 0.181618164 | 3.38E-05 | 9.28E-05 |
| EIF3B | 0.289151932 | 2.24E-11 | 1.59E-10 |
| EIF3C | 0.18651281 | 2.05E-05 | 5.83E-05 |
| EIF3D | 0.190705894 | 1.32E-05 | 3.87E-05 |
| EIF3E | 0.28093566 | 8.52E-11 | 5.55E-10 |
| EIF3H | 0.322514199 | 6.25E-14 | 6.53E-13 |
| EIF3IP1 | 0.152575 | 0.000511946 | 0.001145502 |
| EIF3I | 0.263709775 | 1.22E-09 | 6.67E-09 |
| EIF3J | 0.430779146 | 1.10E-24 | 5.80E-23 |
| EIF3M | 0.329583523 | 1.63E-14 | 1.87E-13 |
| EIF4A1 | 0.418789887 | 2.75E-23 | 1.18E-21 |
| EIF4A3 | 0.460713859 | 2.01E-28 | 1.73E-26 |
| EIF4B | 0.153047529 | 0.000491557 | 0.001103215 |
| EIF4E3 | -0.263563891 | 1.24E-09 | 6.79E-09 |
| EIF4EBP1 | 0.328625232 | 1.96E-14 | 2.22E-13 |
| EIF4EBP2 | -0.158367835 | 0.000308568 | 0.000718924 |
| EIF4EBP3 | -0.253181239 | 5.65E-09 | 2.80E-08 |
| EIF4ENIF1 | -0.179734288 | 4.09E-05 | 0.000110701 |
| EIF4E | 0.401027825 | 2.56E-21 | 8.19E-20 |
| EIF4G1 | 0.134412521 | 0.002237316 | 0.004471726 |
| EIF4G2 | 0.153589307 | 0.00046911 | 0.001056276 |
| EIF4H | 0.161108617 | 0.000241356 | 0.000574312 |
| EIF5A2 | 0.188971015 | 1.58E-05 | 4.60E-05 |
| EIF5AL1 | 0.477113422 | 1.23E-30 | 1.51E-28 |
| EIF5A | 0.429817403 | 1.43E-24 | 7.39E-23 |
| EIF5B | 0.27830042 | 1.30E-10 | 8.20E-10 |
| EIF5 | 0.149081808 | 0.000688876 | 0.001509952 |
| EIF6 | 0.264238277 | 1.13E-09 | 6.20E-09 |
| ELAC1 | -0.149142814 | 0.000685351 | 0.00150305 |
| ELANE | -0.34240018 | 1.30E-15 | 1.77E-14 |
| ELAVL1 | 0.205109981 | 2.69E-06 | 8.78E-06 |
| ELF2 | -0.191573005 | 1.20E-05 | 3.55E-05 |
| ELF3 | -0.194919396 | 8.37E-06 | 2.53E-05 |
| ELF4 | -0.119488518 | 0.006632196 | 0.012175269 |
| ELF5 | -0.224906264 | 2.50E-07 | 9.66E-07 |
| ELFN2 | -0.201007871 | 4.28E-06 | 1.36E-05 |
| ELK1 | 0.198217833 | 5.83E-06 | 1.81E-05 |
| ELL2 | 0.129415817 | 0.003259339 | 0.006321333 |
| ELL | -0.241255003 | 2.96E-08 | 1.32E-07 |
| ELMO1 | -0.237759308 | 4.73E-08 | 2.04E-07 |
| ELMO2 | -0.190100514 | 1.40E-05 | 4.12E-05 |
| ELMO3 | -0.1755868 | 6.17E-05 | 0.000161973 |
| ELMOD1 | -0.12218477 | 0.005495003 | 0.010249544 |
| ELMOD2 | 0.252588212 | 6.15E-09 | 3.03E-08 |
| ELMOD3 | -0.301122302 | 2.96E-12 | 2.39E-11 |
| ELN | -0.406575934 | 6.39E-22 | 2.23E-20 |
| ELOVL4 | 0.15360277 | 0.000468564 | 0.001055286 |
| ELOVL5 | 0.130303071 | 0.0030515 | 0.005951735 |
| ELOVL6 | 0.285552001 | 4.04E-11 | 2.76E-10 |
| ELP2P | -0.123519716 | 0.004999688 | 0.009393155 |
| ELP2 | 0.121784501 | 0.005651893 | 0.010514709 |
| ELP4 | 0.107267081 | 0.014875634 | 0.025498171 |
| EMB | -0.267262398 | 7.14E-10 | 4.06E-09 |
| EMCN | -0.28995186 | 1.96E-11 | 1.41E-10 |
| EME1 | 0.360130624 | 3.24E-17 | 5.62E-16 |
| EME2 | -0.206296167 | 2.35E-06 | 7.75E-06 |
| EMG1 | 0.362160471 | 2.09E-17 | 3.70E-16 |
| EMID1 | -0.207891791 | 1.95E-06 | 6.53E-06 |
| EMILIN2 | -0.160095895 | 0.000264412 | 0.000624379 |
| EMILIN3 | -0.140934146 | 0.001343363 | 0.002790821 |
| EML1 | -0.132603609 | 0.00256752 | 0.005075118 |
| EML3 | -0.301190677 | 2.93E-12 | 2.36E-11 |
| EML4 | -0.16531013 | 0.000164346 | 0.000403508 |
| EML5 | -0.205266435 | 2.64E-06 | 8.64E-06 |
| EML6 | -0.243566094 | 2.16E-08 | 9.83E-08 |
| EMP1 | -0.115140349 | 0.0089148 | 0.015965328 |
| EMP2 | -0.33428993 | 6.54E-15 | 7.98E-14 |
| EMR3 | -0.206188426 | 2.37E-06 | 7.84E-06 |
| EMR4P | -0.40799428 | 4.46E-22 | 1.59E-20 |
| EN2 | -0.133492133 | 0.002400142 | 0.004772111 |
| ENAH | 0.157455334 | 0.000334573 | 0.000773652 |
| ENAM | -0.134031916 | 0.002303384 | 0.004593439 |
| ENC1 | -0.253638342 | 5.29E-09 | 2.63E-08 |
| ENDOD1 | -0.228840932 | 1.52E-07 | 6.06E-07 |
| ENDOU | -0.315328612 | 2.37E-13 | 2.27E-12 |
| ENGASE | -0.377833424 | 6.39E-19 | 1.43E-17 |
| ENG | -0.357983084 | 5.13E-17 | 8.60E-16 |
| ENHO | -0.241199365 | 2.98E-08 | 1.33E-07 |
| ENKUR | -0.152358609 | 0.000521543 | 0.001165671 |
| ENO1 | 0.260895958 | 1.85E-09 | 9.82E-09 |
| ENOPH1 | 0.399801201 | 3.46E-21 | 1.09E-19 |
| ENOSF1 | -0.190631453 | 1.33E-05 | 3.90E-05 |
| ENOX1 | 0.160020017 | 0.00026622 | 0.000628389 |
| ENOX2 | 0.141407003 | 0.001293494 | 0.002697096 |
| ENPEP | 0.139296282 | 0.001530082 | 0.003148046 |
| ENPP1 | 0.217822277 | 6.00E-07 | 2.19E-06 |
| ENPP2 | -0.129263923 | 0.003296182 | 0.00638907 |
| ENPP3 | -0.203511729 | 3.22E-06 | 1.04E-05 |
| ENPP4 | -0.261938292 | 1.58E-09 | 8.52E-09 |
| ENPP5 | -0.353847813 | 1.23E-16 | 1.93E-15 |
| ENPP6 | -0.218163174 | 5.76E-07 | 2.10E-06 |
| ENTPD1 | -0.139439869 | 0.001512806 | 0.003116995 |
| ENTPD3 | -0.275968109 | 1.87E-10 | 1.16E-09 |
| ENTPD5 | 0.121203154 | 0.005886937 | 0.010909457 |
| ENTPD7 | 0.37761524 | 6.72E-19 | 1.49E-17 |
| ENY2 | 0.339047309 | 2.55E-15 | 3.33E-14 |
| EP300 | -0.156118313 | 0.00037639 | 0.00086138 |
| EPAS1 | -0.131740373 | 0.002740261 | 0.005390977 |
| EPB41L1 | -0.317464946 | 1.60E-13 | 1.57E-12 |
| EPB41L2 | -0.178026572 | 4.85E-05 | 0.000129806 |
| EPB41L3 | -0.118979767 | 0.006869038 | 0.012578868 |
| EPB41L4A | -0.460943376 | 1.87E-28 | 1.65E-26 |
| EPB41L5 | -0.169778816 | 0.00010809 | 0.000273897 |
| EPB41 | -0.271150624 | 3.95E-10 | 2.33E-09 |
| EPB49 | -0.264491965 | 1.08E-09 | 5.99E-09 |
| EPC1 | -0.119052772 | 0.006834593 | 0.01251923 |
| EPC2 | -0.099053109 | 0.02457993 | 0.04018515 |
| EPCAM | 0.137904691 | 0.001707144 | 0.003482944 |
| EPDR1 | -0.238859591 | 4.09E-08 | 1.78E-07 |
| EPHA10 | -0.199176675 | 5.24E-06 | 1.64E-05 |
| EPHA1 | -0.123770194 | 0.004911368 | 0.009240251 |
| EPHA2 | -0.166541443 | 0.000146581 | 0.000363192 |
| EPHA3 | -0.170535381 | 0.000100582 | 0.000255909 |
| EPHA4 | -0.335808068 | 4.85E-15 | 6.04E-14 |
| EPHA5 | -0.110927698 | 0.011768481 | 0.020562283 |
| EPHA6 | 0.162705552 | 0.000208785 | 0.000503778 |
| EPHB3 | -0.1621473 | 0.000219672 | 0.000527678 |
| EPHB6 | -0.315091753 | 2.47E-13 | 2.37E-12 |
| EPHX1 | -0.15458064 | 0.000430486 | 0.000977458 |
| EPHX2 | -0.299598056 | 3.85E-12 | 3.05E-11 |
| EPHX4 | 0.161286824 | 0.000237499 | 0.000566077 |
| EPM2AIP1 | -0.328646545 | 1.95E-14 | 2.21E-13 |
| EPM2A | -0.243307437 | 2.24E-08 | 1.02E-07 |
| EPN2 | -0.215760796 | 7.70E-07 | 2.75E-06 |
| EPOR | -0.316136586 | 2.04E-13 | 1.98E-12 |
| EPR1 | 0.492763911 | 7.35E-33 | 1.31E-30 |
| EPRS | 0.264210702 | 1.13E-09 | 6.23E-09 |
| EPS15 | -0.188944354 | 1.59E-05 | 4.61E-05 |
| EPS8L1 | -0.180261024 | 3.88E-05 | 0.000105438 |
| EPS8L2 | -0.274957243 | 2.19E-10 | 1.34E-09 |
| EPS8L3 | 0.199376752 | 5.13E-06 | 1.61E-05 |
| EPT1 | 0.471395811 | 7.52E-30 | 8.03E-28 |
| EPX | -0.289392204 | 2.15E-11 | 1.53E-10 |
| EPYC | 0.14283347 | 0.001153196 | 0.002426324 |
| ERAL1 | 0.200959606 | 4.30E-06 | 1.36E-05 |
| ERAS | -0.162998734 | 0.000203272 | 0.000491413 |
| ERBB2 | -0.162429211 | 0.00021411 | 0.000515618 |
| ERBB3 | -0.17072307 | 9.88E-05 | 0.000251656 |
| ERBB4 | -0.405768352 | 7.83E-22 | 2.70E-20 |
| ERC2 | -0.11657947 | 0.008091784 | 0.014623741 |
| ERCC5 | -0.345333335 | 7.19E-16 | 1.01E-14 |
| ERCC6L | 0.522392183 | 2.12E-37 | 9.42E-35 |
| ERCC8 | 0.210505677 | 1.44E-06 | 4.92E-06 |
| EREG | 0.13279322 | 0.002530933 | 0.005009238 |
| ERGIC2 | 0.413131692 | 1.20E-22 | 4.67E-21 |
| ERGIC3 | 0.114514346 | 0.009295499 | 0.016598037 |
| ERG | -0.28336498 | 5.77E-11 | 3.84E-10 |
| ERH | 0.432509813 | 6.86E-25 | 3.76E-23 |
| ERI1 | 0.212951521 | 1.08E-06 | 3.76E-06 |
| ERICH1 | -0.252132407 | 6.56E-09 | 3.22E-08 |
| ERLIN1 | 0.401209211 | 2.44E-21 | 7.88E-20 |
| ERMAP | -0.342555123 | 1.26E-15 | 1.72E-14 |
| ERMN | -0.159342529 | 0.000282881 | 0.000664656 |
| ERMP1 | 0.167774345 | 0.000130612 | 0.000326252 |
| ERN1 | -0.177014372 | 5.36E-05 | 0.000142209 |
| ERN2 | -0.119930073 | 0.006432597 | 0.011836026 |
| ERO1L | 0.540691952 | 1.95E-40 | 1.77E-37 |
| ERP27 | -0.265055802 | 9.96E-10 | 5.54E-09 |
| ERP44 | 0.114099368 | 0.00955577 | 0.017032347 |
| ERRFI1 | 0.1883327 | 1.69E-05 | 4.89E-05 |
| ERVFRDE1 | -0.421316228 | 1.41E-23 | 6.31E-22 |
| ESAM | -0.302704835 | 2.25E-12 | 1.86E-11 |
| ESCO2 | 0.493342547 | 6.05E-33 | 1.14E-30 |
| ESF1 | 0.194683109 | 8.59E-06 | 2.60E-05 |
| ESM1 | 0.259711235 | 2.20E-09 | 1.15E-08 |
| ESPL1 | 0.446949181 | 1.17E-26 | 8.24E-25 |
| ESPNL | -0.099498417 | 0.023939572 | 0.039218439 |
| ESPNP | -0.202810504 | 3.49E-06 | 1.12E-05 |
| ESR1 | -0.099950533 | 0.023304235 | 0.038303187 |
| ESR2 | -0.141087645 | 0.001326984 | 0.002760303 |
| ESRP1 | 0.113252625 | 0.010107024 | 0.017926986 |
| ESRP2 | -0.186490713 | 2.05E-05 | 5.85E-05 |
| ESRRB | -0.13283662 | 0.002522626 | 0.004993785 |
| ESRRG | -0.204979717 | 2.73E-06 | 8.90E-06 |
| ESX1 | 0.143991503 | 0.001049763 | 0.002223681 |
| ESYT3 | -0.436978769 | 1.99E-25 | 1.19E-23 |
| ETAA1 | -0.172532112 | 8.31E-05 | 0.000213708 |
| ETF1 | 0.152082861 | 0.000534014 | 0.001191148 |
| ETFA | 0.295392089 | 7.89E-12 | 6.01E-11 |
| ETFDH | -0.101493022 | 0.021245238 | 0.0352348 |
| ETHE1 | 0.149805706 | 0.000648114 | 0.001425764 |
| ETNK1 | 0.140181089 | 0.001426443 | 0.00295061 |
| ETNK2 | -0.134612353 | 0.002203325 | 0.004409692 |
| ETS1 | -0.104971606 | 0.017172978 | 0.028995901 |
| ETS2 | -0.119706156 | 0.006533132 | 0.012006642 |
| ETV1 | -0.284316266 | 4.94E-11 | 3.32E-10 |
| ETV3 | -0.098431094 | 0.025499133 | 0.041562152 |
| ETV5 | -0.246683668 | 1.41E-08 | 6.58E-08 |
| EVC2 | -0.128772179 | 0.00341807 | 0.006606109 |
| EVC | -0.279525864 | 1.07E-10 | 6.84E-10 |
| EVI2A | -0.110980089 | 0.01172853 | 0.020499287 |
| EVI2B | -0.226894474 | 1.94E-07 | 7.65E-07 |
| EVI5L | -0.28623763 | 3.62E-11 | 2.48E-10 |
| EVI5 | -0.179983081 | 3.99E-05 | 0.00010819 |
| EVL | -0.340218422 | 2.02E-15 | 2.67E-14 |
| EVPLL | -0.284562965 | 4.75E-11 | 3.20E-10 |
| EVPL | -0.318463434 | 1.33E-13 | 1.33E-12 |
| EWSR1 | -0.137548496 | 0.001755391 | 0.003571535 |
| EXD2 | 0.190703795 | 1.32E-05 | 3.87E-05 |
| EXD3 | -0.219597374 | 4.83E-07 | 1.79E-06 |
| EXO1 | 0.527189126 | 3.53E-38 | 1.91E-35 |
| EXOC3L | -0.2650452 | 9.97E-10 | 5.55E-09 |
| EXOC3 | -0.101951949 | 0.020663965 | 0.034342009 |
| EXOC4 | 0.132989371 | 0.002493585 | 0.004939721 |
| EXOC5 | 0.394731077 | 1.20E-20 | 3.42E-19 |
| EXOC6 | 0.169894546 | 0.000106908 | 0.000271143 |
| EXOC7 | -0.233340705 | 8.48E-08 | 3.53E-07 |
| EXOC8 | -0.150112996 | 0.000631496 | 0.001391506 |
| EXOG | -0.113801958 | 0.00974627 | 0.017347145 |
| EXOSC1 | 0.218239055 | 5.70E-07 | 2.09E-06 |
| EXOSC2 | 0.335520914 | 5.13E-15 | 6.37E-14 |
| EXOSC3 | 0.368484009 | 5.24E-18 | 1.02E-16 |
| EXOSC4 | 0.263443414 | 1.27E-09 | 6.90E-09 |
| EXOSC5 | 0.140553538 | 0.00138478 | 0.00287186 |
| EXOSC6 | 0.115936097 | 0.00845095 | 0.015199457 |
| EXOSC8 | 0.254996605 | 4.36E-09 | 2.19E-08 |
| EXOSC9 | 0.164322396 | 0.000180037 | 0.000439494 |
| EXPH5 | -0.295611974 | 7.61E-12 | 5.81E-11 |
| EXT2 | 0.099893348 | 0.023383779 | 0.038408659 |
| EXTL1 | -0.112964929 | 0.010300649 | 0.018241283 |
| EYA1 | -0.097742964 | 0.026550445 | 0.043117508 |
| EYS | 0.121673265 | 0.005696203 | 0.010587288 |
| EZH1 | -0.514775243 | 3.44E-36 | 1.23E-33 |
| EZH2 | 0.327523882 | 2.42E-14 | 2.69E-13 |
| EZR | -0.174085571 | 7.15E-05 | 0.000185867 |
| F10 | -0.21401751 | 9.49E-07 | 3.35E-06 |
| F11R | -0.189713627 | 1.46E-05 | 4.28E-05 |
| F11 | -0.390948555 | 2.97E-20 | 8.05E-19 |
| F12 | 0.319188531 | 1.16E-13 | 1.17E-12 |
| F13A1 | -0.139750846 | 0.001476004 | 0.003045878 |
| F13B | 0.180096079 | 3.94E-05 | 0.00010707 |
| F2 | 0.267476869 | 6.92E-10 | 3.94E-09 |
| F7 | 0.174569388 | 6.82E-05 | 0.000177914 |
| F8 | -0.355659812 | 8.41E-17 | 1.36E-15 |
| FAAH | -0.412745999 | 1.33E-22 | 5.06E-21 |
| FABP3 | -0.213242473 | 1.04E-06 | 3.64E-06 |
| FABP4 | -0.15702921 | 0.000347405 | 0.000801008 |
| FADD | 0.356199039 | 7.50E-17 | 1.22E-15 |
| FADS1 | 0.241824291 | 2.74E-08 | 1.23E-07 |
| FADS2 | 0.12206762 | 0.005540511 | 0.010327681 |
| FADS3 | -0.154181076 | 0.000445683 | 0.001008758 |
| FADS6 | -0.248694254 | 1.06E-08 | 5.07E-08 |
| FAF1 | 0.133253794 | 0.002444034 | 0.004850695 |
| FAHD1 | 0.11855381 | 0.007073135 | 0.012917116 |
| FAHD2B | -0.197414987 | 6.37E-06 | 1.96E-05 |
| FAH | -0.122204881 | 0.005487225 | 0.010236947 |
| FAIM2 | -0.181565914 | 3.40E-05 | 9.33E-05 |
| FAIM3 | -0.319502226 | 1.10E-13 | 1.11E-12 |
| FAM100A | -0.184192227 | 2.60E-05 | 7.29E-05 |
| FAM101A | 0.204300157 | 2.95E-06 | 9.57E-06 |
| FAM103A1 | 0.29912873 | 4.17E-12 | 3.29E-11 |
| FAM104A | 0.215490296 | 7.95E-07 | 2.83E-06 |
| FAM105A | -0.157861044 | 0.000322768 | 0.000749041 |
| FAM106C | -0.134404078 | 0.002238762 | 0.004473943 |
| FAM107A | -0.388411899 | 5.44E-20 | 1.41E-18 |
| FAM108A1 | -0.150571862 | 0.000607416 | 0.001342293 |
| FAM108B1 | 0.139345751 | 0.00152411 | 0.003138344 |
| FAM10A4 | 0.118362249 | 0.007166679 | 0.01307242 |
| FAM110A | -0.141045087 | 0.001331506 | 0.002767406 |
| FAM110B | -0.272887043 | 3.02E-10 | 1.81E-09 |
| FAM110C | -0.097771291 | 0.026506442 | 0.043056543 |
| FAM111A | -0.283831376 | 5.35E-11 | 3.58E-10 |
| FAM111B | 0.284532186 | 4.77E-11 | 3.22E-10 |
| FAM113A | -0.261719265 | 1.64E-09 | 8.77E-09 |
| FAM114A1 | 0.268620048 | 5.81E-10 | 3.35E-09 |
| FAM116B | -0.40217865 | 1.92E-21 | 6.30E-20 |
| FAM117A | -0.462552238 | 1.15E-28 | 1.04E-26 |
| FAM117B | -0.109099255 | 0.013240343 | 0.022899367 |
| FAM118A | -0.232158036 | 9.89E-08 | 4.08E-07 |
| FAM118B | -0.146856613 | 0.000829527 | 0.001789015 |
| FAM119A | 0.288835709 | 2.36E-11 | 1.67E-10 |
| FAM120B | -0.164090679 | 0.000183916 | 0.000447923 |
| FAM120C | -0.099912824 | 0.023356662 | 0.03837323 |
| FAM122A | -0.242292666 | 2.57E-08 | 1.16E-07 |
| FAM122C | -0.185628055 | 2.24E-05 | 6.35E-05 |
| FAM124B | -0.185454661 | 2.28E-05 | 6.45E-05 |
| FAM125B | -0.341201509 | 1.66E-15 | 2.23E-14 |
| FAM126A | 0.099948309 | 0.023307324 | 0.038305113 |
| FAM126B | -0.182894745 | 2.97E-05 | 8.23E-05 |
| FAM128A | 0.188119549 | 1.73E-05 | 5.00E-05 |
| FAM128B | 0.111311871 | 0.011478296 | 0.020127045 |
| FAM129A | -0.279582651 | 1.06E-10 | 6.78E-10 |
| FAM129B | -0.128199058 | 0.003565281 | 0.006863415 |
| FAM129C | -0.291856039 | 1.43E-11 | 1.05E-10 |
| FAM131B | -0.138702531 | 0.001603458 | 0.003284476 |
| FAM131C | 0.103309323 | 0.019024911 | 0.031853738 |
| FAM133A | 0.18393052 | 2.67E-05 | 7.47E-05 |
| FAM134A | -0.120014912 | 0.00639487 | 0.011770943 |
| FAM134B | -0.158487511 | 0.0003053 | 0.000711976 |
| FAM134C | -0.118672531 | 0.007015712 | 0.012819274 |
| FAM135B | -0.176642169 | 5.56E-05 | 0.000147104 |
| FAM136A | 0.430644165 | 1.14E-24 | 5.97E-23 |
| FAM136B | 0.134871798 | 0.002159898 | 0.004331404 |
| FAM138B | -0.166274599 | 0.000150271 | 0.000371597 |
| FAM138D | -0.115721829 | 0.008573683 | 0.015395811 |
| FAM138F | -0.278445289 | 1.27E-10 | 8.03E-10 |
| FAM13AOS | -0.44585256 | 1.61E-26 | 1.11E-24 |
| FAM13A | -0.176974714 | 5.38E-05 | 0.000142673 |
| FAM13B | -0.38382423 | 1.60E-19 | 3.94E-18 |
| FAM13C | -0.235823198 | 6.12E-08 | 2.60E-07 |
| FAM149A | -0.301848253 | 2.61E-12 | 2.12E-11 |
| FAM150A | -0.197499155 | 6.31E-06 | 1.95E-05 |
| FAM150B | -0.336899028 | 3.91E-15 | 4.95E-14 |
| FAM151A | -0.158749509 | 0.00029826 | 0.000696959 |
| FAM153A | -0.262527878 | 1.45E-09 | 7.85E-09 |
| FAM153B | -0.296098176 | 7.00E-12 | 5.37E-11 |
| FAM153C | -0.287892489 | 2.76E-11 | 1.92E-10 |
| FAM154A | -0.174287687 | 7.01E-05 | 0.000182603 |
| FAM154B | -0.239752192 | 3.63E-08 | 1.59E-07 |
| FAM156A | -0.344203438 | 9.04E-16 | 1.26E-14 |
| FAM157A | -0.124945577 | 0.004515426 | 0.008546002 |
| FAM157B | -0.172578502 | 8.27E-05 | 0.000212781 |
| FAM158A | 0.273237194 | 2.86E-10 | 1.72E-09 |
| FAM159A | -0.248542024 | 1.09E-08 | 5.17E-08 |
| FAM160A1 | -0.141345055 | 0.001299929 | 0.002709666 |
| FAM160A2 | -0.355780553 | 8.20E-17 | 1.32E-15 |
| FAM160B2 | -0.273634817 | 2.69E-10 | 1.62E-09 |
| FAM161B | -0.252584344 | 6.15E-09 | 3.03E-08 |
| FAM162A | 0.27711346 | 1.56E-10 | 9.76E-10 |
| FAM162B | -0.225362124 | 2.36E-07 | 9.15E-07 |
| FAM163A | 0.116762672 | 0.007992035 | 0.014459159 |
| FAM164C | -0.137299778 | 0.001789817 | 0.003637136 |
| FAM166A | -0.226762055 | 1.98E-07 | 7.77E-07 |
| FAM166B | -0.156650628 | 0.00035919 | 0.000825808 |
| FAM167A | -0.156266712 | 0.00037152 | 0.0008515 |
| FAM167B | -0.178569639 | 4.59E-05 | 0.000123421 |
| FAM170A | -0.126332526 | 0.004085447 | 0.00778826 |
| FAM171A1 | -0.121366978 | 0.005819828 | 0.01079398 |
| FAM172A | -0.162858867 | 0.000205885 | 0.000497249 |
| FAM174A | -0.158063966 | 0.00031701 | 0.000736534 |
| FAM174B | -0.231098004 | 1.14E-07 | 4.64E-07 |
| FAM175B | 0.294729832 | 8.83E-12 | 6.66E-11 |
| FAM176A | -0.163519954 | 0.000193806 | 0.00046995 |
| FAM177A1 | 0.100599597 | 0.022417673 | 0.036969862 |
| FAM178A | -0.165332072 | 0.000164013 | 0.000402837 |
| FAM179A | -0.320478505 | 9.15E-14 | 9.32E-13 |
| FAM180A | -0.153221239 | 0.000484253 | 0.001088167 |
| FAM180B | -0.237892109 | 4.65E-08 | 2.01E-07 |
| FAM181A | -0.184486086 | 2.52E-05 | 7.08E-05 |
| FAM181B | -0.201680358 | 3.96E-06 | 1.26E-05 |
| FAM182A | -0.274444952 | 2.37E-10 | 1.45E-09 |
| FAM182B | -0.367248818 | 6.88E-18 | 1.32E-16 |
| FAM183A | -0.20161202 | 4.00E-06 | 1.27E-05 |
| FAM183B | -0.275092044 | 2.14E-10 | 1.31E-09 |
| FAM184A | -0.469241586 | 1.47E-29 | 1.53E-27 |
| FAM186A | -0.205201791 | 2.66E-06 | 8.70E-06 |
| FAM186B | -0.122398071 | 0.005413008 | 0.010106035 |
| FAM187B | -0.17502107 | 6.52E-05 | 0.000170795 |
| FAM188B | -0.19894006 | 5.38E-06 | 1.68E-05 |
| FAM189A1 | -0.138102546 | 0.001680873 | 0.003434253 |
| FAM189A2 | -0.460585274 | 2.09E-28 | 1.79E-26 |
| FAM189B | 0.234489836 | 7.30E-08 | 3.06E-07 |
| FAM18A | -0.178565669 | 4.60E-05 | 0.000123454 |
| FAM18B | 0.192574141 | 1.08E-05 | 3.21E-05 |
| FAM190A | -0.206214508 | 2.37E-06 | 7.82E-06 |
| FAM193A | -0.212505284 | 1.14E-06 | 3.94E-06 |
| FAM193B | -0.422241201 | 1.10E-23 | 5.00E-22 |
| FAM196A | 0.230193622 | 1.28E-07 | 5.17E-07 |
| FAM198A | -0.353562208 | 1.31E-16 | 2.05E-15 |
| FAM199X | 0.278230633 | 1.31E-10 | 8.29E-10 |
| FAM19A1 | -0.143317233 | 0.001108895 | 0.002341 |
| FAM19A2 | -0.213862915 | 9.67E-07 | 3.41E-06 |
| FAM200A | 0.098819426 | 0.02492185 | 0.040697544 |
| FAM200B | -0.102546179 | 0.019931894 | 0.033219269 |
| FAM20A | -0.237364104 | 4.99E-08 | 2.15E-07 |
| FAM20B | 0.170294881 | 0.000102913 | 0.000261509 |
| FAM21B | -0.146786422 | 0.000834368 | 0.001798679 |
| FAM21C | -0.145979726 | 0.000891914 | 0.00191201 |
| FAM22A | -0.271215075 | 3.91E-10 | 2.31E-09 |
| FAM22D | -0.214266746 | 9.21E-07 | 3.26E-06 |
| FAM22F | -0.157690601 | 0.000327679 | 0.000759292 |
| FAM22G | -0.142520007 | 0.001182766 | 0.00248305 |
| FAM23A | -0.179151333 | 4.34E-05 | 0.000116882 |
| FAM24B | 0.247308225 | 1.29E-08 | 6.07E-08 |
| FAM25A | 0.176802808 | 5.47E-05 | 0.000144924 |
| FAM25B | 0.113464376 | 0.009966583 | 0.017704592 |
| FAM35A | 0.25717805 | 3.18E-09 | 1.62E-08 |
| FAM35B2 | 0.162147401 | 0.00021967 | 0.000527678 |
| FAM35B | 0.218438834 | 5.57E-07 | 2.04E-06 |
| FAM38B | -0.232563016 | 9.39E-08 | 3.88E-07 |
| FAM3B | -0.161861505 | 0.000225449 | 0.000540127 |
| FAM3C | 0.148795065 | 0.000705669 | 0.001544102 |
| FAM3D | -0.234566012 | 7.22E-08 | 3.03E-07 |
| FAM40A | -0.197137126 | 6.57E-06 | 2.02E-05 |
| FAM40B | 0.362453357 | 1.96E-17 | 3.49E-16 |
| FAM41C | -0.144006564 | 0.001048476 | 0.002221661 |
| FAM43A | -0.179178309 | 4.32E-05 | 0.000116582 |
| FAM43B | -0.097302423 | 0.02724292 | 0.044124484 |
| FAM45A | 0.284814809 | 4.56E-11 | 3.08E-10 |
| FAM45B | 0.242472926 | 2.51E-08 | 1.13E-07 |
| FAM46B | -0.175871297 | 6.00E-05 | 0.000157914 |
| FAM46C | -0.236479755 | 5.61E-08 | 2.40E-07 |
| FAM46D | 0.103210414 | 0.019140396 | 0.032017595 |
| FAM47C | -0.22934674 | 1.42E-07 | 5.71E-07 |
| FAM47E | -0.288744857 | 2.40E-11 | 1.69E-10 |
| FAM48A | -0.162970974 | 0.000203788 | 0.000492482 |
| FAM49A | -0.247208587 | 1.31E-08 | 6.15E-08 |
| FAM49B | 0.317752993 | 1.52E-13 | 1.50E-12 |
| FAM53A | -0.120231158 | 0.006299604 | 0.011610556 |
| FAM53B | -0.22657751 | 2.02E-07 | 7.95E-07 |
| FAM54A | 0.461517721 | 1.57E-28 | 1.40E-26 |
| FAM54B | -0.191206743 | 1.25E-05 | 3.68E-05 |
| FAM55D | -0.116715468 | 0.008017631 | 0.014502842 |
| FAM58A | 0.151169676 | 0.000577322 | 0.001280604 |
| FAM58B | 0.152043472 | 0.000535818 | 0.001194772 |
| FAM59A | -0.118909614 | 0.006902285 | 0.012628181 |
| FAM5C | -0.164007472 | 0.000185328 | 0.000451087 |
| FAM60A | 0.39904708 | 4.17E-21 | 1.29E-19 |
| FAM63A | -0.112510652 | 0.010613092 | 0.018736484 |
| FAM64A | 0.48354001 | 1.55E-31 | 2.25E-29 |
| FAM65A | -0.175369446 | 6.30E-05 | 0.00016533 |
| FAM65B | -0.31725723 | 1.66E-13 | 1.63E-12 |
| FAM66A | -0.26811411 | 6.28E-10 | 3.60E-09 |
| FAM66C | -0.223724502 | 2.90E-07 | 1.11E-06 |
| FAM66D | -0.227902762 | 1.71E-07 | 6.78E-07 |
| FAM66E | -0.167590722 | 0.000132882 | 0.000331167 |
| FAM69A | 0.14179299 | 0.001254057 | 0.002623073 |
| FAM70A | -0.202638283 | 3.56E-06 | 1.14E-05 |
| FAM70B | -0.125976528 | 0.004192126 | 0.007974135 |
| FAM71A | -0.162751527 | 0.000207912 | 0.00050184 |
| FAM71C | -0.099221272 | 0.024336392 | 0.039813046 |
| FAM71D | 0.155816607 | 0.000386477 | 0.000883554 |
| FAM71F2 | -0.165783564 | 0.000157289 | 0.000387324 |
| FAM72A | 0.469351365 | 1.42E-29 | 1.49E-27 |
| FAM72B | 0.481125717 | 3.40E-31 | 4.66E-29 |
| FAM72D | 0.487095973 | 4.85E-32 | 7.69E-30 |
| FAM73B | -0.262522344 | 1.45E-09 | 7.86E-09 |
| FAM74A1 | -0.191436066 | 1.22E-05 | 3.60E-05 |
| FAM74A3 | -0.197229464 | 6.50E-06 | 2.00E-05 |
| FAM74A4 | -0.114019131 | 0.009606835 | 0.017120312 |
| FAM76A | -0.29151574 | 1.51E-11 | 1.10E-10 |
| FAM78A | -0.260962498 | 1.83E-09 | 9.73E-09 |
| FAM7A3 | -0.150309845 | 0.00062106 | 0.001370322 |
| FAM81B | -0.258369063 | 2.67E-09 | 1.38E-08 |
| FAM82A1 | -0.346377985 | 5.80E-16 | 8.32E-15 |
| FAM82A2 | -0.281555749 | 7.71E-11 | 5.05E-10 |
| FAM82B | 0.142591533 | 0.001175958 | 0.002470314 |
| FAM83A | 0.342187081 | 1.36E-15 | 1.85E-14 |
| FAM83B | 0.132300681 | 0.002626974 | 0.005184436 |
| FAM83D | 0.513632189 | 5.19E-36 | 1.73E-33 |
| FAM83E | -0.204419242 | 2.91E-06 | 9.46E-06 |
| FAM83F | 0.191070108 | 1.27E-05 | 3.74E-05 |
| FAM83G | 0.143956491 | 0.00105276 | 0.002229286 |
| FAM83H | 0.141538733 | 0.001279909 | 0.002671555 |
| FAM86A | 0.115843142 | 0.008504001 | 0.015281665 |
| FAM86B1 | -0.129670783 | 0.003198334 | 0.006213266 |
| FAM86C | 0.164551356 | 0.000176279 | 0.000431217 |
| FAM89A | 0.096627505 | 0.028333931 | 0.045742558 |
| FAM8A1 | -0.142434469 | 0.001190955 | 0.002499456 |
| FAM91A1 | 0.370281394 | 3.51E-18 | 7.02E-17 |
| FAM92A1 | 0.150903884 | 0.000590527 | 0.001307139 |
| FAM92A3 | 0.099499137 | 0.02393855 | 0.039218439 |
| FAM92B | -0.177788533 | 4.97E-05 | 0.000132716 |
| FAM95B1 | -0.362320898 | 2.02E-17 | 3.58E-16 |
| FAM96A | 0.233165445 | 8.68E-08 | 3.61E-07 |
| FAM96B | 0.101424947 | 0.02133266 | 0.035360537 |
| FAM98A | 0.328023391 | 2.20E-14 | 2.46E-13 |
| FAM98B | 0.181136857 | 3.55E-05 | 9.72E-05 |
| FAM98C | -0.142416978 | 0.001192636 | 0.002502458 |
| FAM9A | 0.113929991 | 0.009663851 | 0.017215781 |
| FAM9B | 0.227635807 | 1.77E-07 | 7.00E-07 |
| FANCA | 0.231000444 | 1.15E-07 | 4.69E-07 |
| FANCB | 0.399072191 | 4.14E-21 | 1.29E-19 |
| FANCC | 0.09692001 | 0.027856569 | 0.045033735 |
| FANCD2 | 0.314652509 | 2.68E-13 | 2.55E-12 |
| FANCF | 0.233007835 | 8.86E-08 | 3.68E-07 |
| FANCG | 0.35600584 | 7.81E-17 | 1.27E-15 |
| FANCI | 0.434978353 | 3.47E-25 | 1.99E-23 |
| FANCM | 0.396113904 | 8.54E-21 | 2.50E-19 |
| FANK1 | -0.259844948 | 2.16E-09 | 1.13E-08 |
| FAP | 0.148688273 | 0.00071202 | 0.001556244 |
| FARP1 | -0.154333087 | 0.000439844 | 0.000996893 |
| FARP2 | -0.319052052 | 1.19E-13 | 1.20E-12 |
| FARSA | 0.198578779 | 5.60E-06 | 1.74E-05 |
| FARSB | 0.398104872 | 5.25E-21 | 1.60E-19 |
| FASTKD1 | 0.225811918 | 2.23E-07 | 8.68E-07 |
| FASTKD2 | 0.237908643 | 4.64E-08 | 2.01E-07 |
| FAS | -0.166645981 | 0.000145159 | 0.000359847 |
| FAT1 | 0.119199817 | 0.006765683 | 0.012403234 |
| FAT2 | -0.18835623 | 1.69E-05 | 4.88E-05 |
| FAT3 | -0.219814928 | 4.70E-07 | 1.75E-06 |
| FAT4 | -0.373966951 | 1.54E-18 | 3.25E-17 |
| FATE1 | 0.227388024 | 1.83E-07 | 7.21E-07 |
| FBF1 | -0.225886226 | 2.21E-07 | 8.61E-07 |
| FBLN1 | -0.241886454 | 2.72E-08 | 1.22E-07 |
| FBLN2 | -0.133623033 | 0.002376343 | 0.004728555 |
| FBLN5 | -0.283467043 | 5.67E-11 | 3.78E-10 |
| FBL | 0.238068122 | 4.54E-08 | 1.97E-07 |
| FBN3 | -0.227131136 | 1.89E-07 | 7.44E-07 |
| FBP1 | -0.282220123 | 6.93E-11 | 4.57E-10 |
| FBP2 | -0.161839153 | 0.000225907 | 0.000541159 |
| FBRS | -0.196312168 | 7.19E-06 | 2.20E-05 |
| FBXL12 | -0.220337558 | 4.41E-07 | 1.64E-06 |
| FBXL14 | 0.185325611 | 2.31E-05 | 6.53E-05 |
| FBXL15 | -0.152475972 | 0.000516317 | 0.001154895 |
| FBXL16 | -0.214865319 | 8.57E-07 | 3.04E-06 |
| FBXL17 | -0.112935647 | 0.010320539 | 0.018271651 |
| FBXL18 | 0.130623708 | 0.002979406 | 0.005821916 |
| FBXL3 | -0.127526863 | 0.003745254 | 0.007184963 |
| FBXL4 | 0.133405502 | 0.002416012 | 0.004801754 |
| FBXL7 | -0.258129041 | 2.77E-09 | 1.43E-08 |
| FBXL8 | -0.290273542 | 1.86E-11 | 1.34E-10 |
| FBXO15 | -0.250106995 | 8.73E-09 | 4.21E-08 |
| FBXO22OS | 0.344433455 | 8.63E-16 | 1.20E-14 |
| FBXO22 | 0.254907704 | 4.41E-09 | 2.22E-08 |
| FBXO24 | -0.291762338 | 1.45E-11 | 1.06E-10 |
| FBXO25 | -0.119633612 | 0.006566004 | 0.012063726 |
| FBXO28 | 0.203834533 | 3.11E-06 | 1.01E-05 |
| FBXO2 | -0.184531913 | 2.51E-05 | 7.05E-05 |
| FBXO30 | 0.285649426 | 3.98E-11 | 2.72E-10 |
| FBXO31 | -0.227412582 | 1.82E-07 | 7.19E-07 |
| FBXO32 | 0.229611033 | 1.37E-07 | 5.54E-07 |
| FBXO33 | 0.15296784 | 0.000494941 | 0.001110313 |
| FBXO38 | -0.291965377 | 1.40E-11 | 1.03E-10 |
| FBXO3 | -0.152233317 | 0.000527175 | 0.00117747 |
| FBXO42 | -0.24788784 | 1.19E-08 | 5.63E-08 |
| FBXO43 | 0.204667379 | 2.83E-06 | 9.21E-06 |
| FBXO44 | -0.383083018 | 1.90E-19 | 4.62E-18 |
| FBXO45 | 0.406070543 | 7.26E-22 | 2.52E-20 |
| FBXO48 | -0.129039748 | 0.00335125 | 0.006490773 |
| FBXO5 | 0.409374217 | 3.14E-22 | 1.14E-20 |
| FBXO7 | -0.14661901 | 0.000846021 | 0.001822619 |
| FBXO9 | -0.12598604 | 0.004189243 | 0.00796941 |
| FBXW11 | -0.185262892 | 2.33E-05 | 6.57E-05 |
| FBXW2 | -0.112517848 | 0.010608077 | 0.018730941 |
| FBXW4 | -0.420022478 | 1.99E-23 | 8.67E-22 |
| FBXW7 | -0.146588534 | 0.000848158 | 0.001826239 |
| FCAMR | -0.301818027 | 2.62E-12 | 2.13E-11 |
| FCER1A | -0.401069 | 2.53E-21 | 8.12E-20 |
| FCER2 | -0.269754706 | 4.89E-10 | 2.84E-09 |
| FCF1 | 0.249643941 | 9.32E-09 | 4.48E-08 |
| FCGBP | -0.427280046 | 2.86E-24 | 1.44E-22 |
| FCGR2A | -0.099590452 | 0.02380904 | 0.039030189 |
| FCGR2C | -0.131863499 | 0.002714992 | 0.005347054 |
| FCGRT | -0.387674837 | 6.48E-20 | 1.66E-18 |
| FCHO2 | -0.144137391 | 0.001037358 | 0.002199735 |
| FCHSD1 | -0.379449899 | 4.41E-19 | 1.01E-17 |
| FCHSD2 | -0.318269199 | 1.38E-13 | 1.37E-12 |
| FCN1 | -0.26732242 | 7.08E-10 | 4.03E-09 |
| FCN2 | -0.09897811 | 0.024689222 | 0.040350628 |
| FCN3 | -0.181643228 | 3.37E-05 | 9.27E-05 |
| FCRL1 | -0.289499829 | 2.12E-11 | 1.51E-10 |
| FCRL2 | -0.165511208 | 0.000161314 | 0.000396597 |
| FCRL3 | -0.247109817 | 1.33E-08 | 6.22E-08 |
| FCRL4 | -0.11907467 | 0.006824291 | 0.012503797 |
| FCRL6 | -0.259179946 | 2.38E-09 | 1.24E-08 |
| FCRLA | -0.110029599 | 0.012472307 | 0.021674184 |
| FDPSL2A | -0.169332808 | 0.00011276 | 0.000284505 |
| FDPS | 0.207846218 | 1.96E-06 | 6.57E-06 |
| FDXR | -0.229486917 | 1.40E-07 | 5.62E-07 |
| FECH | 0.214452023 | 9.01E-07 | 3.19E-06 |
| FEM1A | -0.112463033 | 0.010646325 | 0.018786858 |
| FEM1B | 0.163603345 | 0.000192331 | 0.000466598 |
| FEM1C | -0.226251644 | 2.11E-07 | 8.25E-07 |
| FEN1 | 0.413377705 | 1.13E-22 | 4.40E-21 |
| FER1L5 | -0.255609423 | 3.99E-09 | 2.01E-08 |
| FERMT1 | 0.136694741 | 0.001876163 | 0.003798314 |
| FERMT2 | 0.205422472 | 2.59E-06 | 8.50E-06 |
| FERMT3 | -0.215564555 | 7.88E-07 | 2.81E-06 |
| FES | -0.197213692 | 6.51E-06 | 2.01E-05 |
| FETUB | 0.126204672 | 0.004123474 | 0.007856257 |
| FEZ1 | -0.201837038 | 3.90E-06 | 1.24E-05 |
| FEZF1 | -0.123604223 | 0.004969732 | 0.009341264 |
| FFAR1 | -0.105505484 | 0.016612624 | 0.02814038 |
| FGA | 0.288352414 | 2.56E-11 | 1.79E-10 |
| FGB | 0.331748788 | 1.07E-14 | 1.26E-13 |
| FGD1 | 0.166404287 | 0.000148467 | 0.0003675 |
| FGD2 | -0.306901304 | 1.08E-12 | 9.39E-12 |
| FGD3 | -0.361936974 | 2.19E-17 | 3.87E-16 |
| FGD4 | -0.190382184 | 1.36E-05 | 4.00E-05 |
| FGD5 | -0.308987524 | 7.43E-13 | 6.64E-12 |
| FGF10 | -0.196188894 | 7.29E-06 | 2.23E-05 |
| FGF12 | 0.190584853 | 1.33E-05 | 3.92E-05 |
| FGF13 | -0.117447112 | 0.007629013 | 0.013848762 |
| FGF14 | -0.346038695 | 6.22E-16 | 8.87E-15 |
| FGF17 | -0.280523854 | 9.10E-11 | 5.90E-10 |
| FGF18 | -0.236711436 | 5.44E-08 | 2.32E-07 |
| FGF20 | -0.21503358 | 8.40E-07 | 2.99E-06 |
| FGF21 | 0.184002849 | 2.65E-05 | 7.42E-05 |
| FGF22 | -0.16640282 | 0.000148487 | 0.000367505 |
| FGF23 | 0.167740416 | 0.000131029 | 0.000327211 |
| FGF2 | -0.142410996 | 0.001193212 | 0.002502964 |
| FGF3 | 0.102585164 | 0.019884665 | 0.033154378 |
| FGF5 | 0.303473965 | 1.97E-12 | 1.64E-11 |
| FGF9 | -0.244051507 | 2.02E-08 | 9.25E-08 |
| FGFBP2 | -0.218736301 | 5.37E-07 | 1.97E-06 |
| FGFBP3 | -0.098202271 | 0.025844668 | 0.042077318 |
| FGFR1OP2 | 0.18532785 | 2.31E-05 | 6.53E-05 |
| FGFR1OP | 0.131177297 | 0.002858573 | 0.005600768 |
| FGFR1 | -0.112911381 | 0.010337048 | 0.018292778 |
| FGFR2 | -0.431722699 | 8.52E-25 | 4.59E-23 |
| FGFR3 | -0.356531461 | 6.99E-17 | 1.15E-15 |
| FGFR4 | -0.124989187 | 0.004501307 | 0.008521701 |
| FGGY | -0.201312924 | 4.13E-06 | 1.31E-05 |
| FGG | 0.23605134 | 5.94E-08 | 2.53E-07 |
| FGL1 | 0.25668273 | 3.42E-09 | 1.74E-08 |
| FGL2 | -0.213054548 | 1.06E-06 | 3.72E-06 |
| FGR | -0.267676116 | 6.71E-10 | 3.83E-09 |
| FHAD1 | -0.23643657 | 5.64E-08 | 2.41E-07 |
| FHDC1 | -0.360726621 | 2.85E-17 | 4.97E-16 |
| FHIT | -0.213011617 | 1.07E-06 | 3.74E-06 |
| FHL1 | -0.340614604 | 1.87E-15 | 2.48E-14 |
| FHL2 | 0.197621818 | 6.23E-06 | 1.92E-05 |
| FHL5 | -0.27295771 | 2.99E-10 | 1.79E-09 |
| FHOD1 | -0.294890272 | 8.59E-12 | 6.50E-11 |
| FH | 0.279119638 | 1.14E-10 | 7.25E-10 |
| FIBIN | -0.09796983 | 0.026199791 | 0.042610369 |
| FIG4 | -0.27087862 | 4.12E-10 | 2.42E-09 |
| FIGF | -0.407826498 | 4.66E-22 | 1.64E-20 |
| FIGNL1 | 0.208936912 | 1.73E-06 | 5.84E-06 |
| FIGNL2 | -0.167691827 | 0.000131628 | 0.000328502 |
| FIGN | 0.212261463 | 1.17E-06 | 4.05E-06 |
| FILIP1L | -0.193720744 | 9.53E-06 | 2.86E-05 |
| FILIP1 | -0.176401536 | 5.70E-05 | 0.000150297 |
| FIP1L1 | 0.223208575 | 3.09E-07 | 1.18E-06 |
| FITM1 | -0.305529298 | 1.37E-12 | 1.18E-11 |
| FIZ1 | -0.166901069 | 0.000141743 | 0.000351815 |
| FJX1 | 0.124317002 | 0.00472343 | 0.00891688 |
| FKBP10 | 0.276967085 | 1.60E-10 | 9.96E-10 |
| FKBP11 | 0.115962234 | 0.008436086 | 0.015176279 |
| FKBP14 | 0.231245519 | 1.11E-07 | 4.56E-07 |
| FKBP15 | -0.263547495 | 1.25E-09 | 6.81E-09 |
| FKBP1A | 0.131167164 | 0.002860744 | 0.005603739 |
| FKBP3 | 0.460520871 | 2.13E-28 | 1.81E-26 |
| FKBP4 | 0.391406823 | 2.66E-20 | 7.27E-19 |
| FKBP6 | -0.257221437 | 3.16E-09 | 1.62E-08 |
| FKBP8 | -0.181098072 | 3.56E-05 | 9.76E-05 |
| FKBP9L | 0.121289128 | 0.005851632 | 0.010849947 |
| FKBP9 | 0.131377113 | 0.002816066 | 0.005526193 |
| FKBPL | 0.20607814 | 2.41E-06 | 7.93E-06 |
| FKRP | -0.168313462 | 0.000124156 | 0.000311136 |
| FKSG83 | -0.155514401 | 0.000396833 | 0.000905158 |
| FKTN | 0.103061985 | 0.019314847 | 0.032285094 |
| FLAD1 | 0.309649152 | 6.60E-13 | 5.95E-12 |
| FLCN | -0.278362187 | 1.28E-10 | 8.13E-10 |
| FLI1 | -0.296148834 | 6.94E-12 | 5.33E-11 |
| FLII | -0.126718017 | 0.003972705 | 0.007591437 |
| FLJ10038 | -0.388076681 | 5.89E-20 | 1.52E-18 |
| FLJ10213 | -0.226098 | 2.15E-07 | 8.40E-07 |
| FLJ10357 | -0.317928281 | 1.47E-13 | 1.45E-12 |
| FLJ10661 | -0.279846418 | 1.01E-10 | 6.53E-10 |
| FLJ11235 | -0.389577025 | 4.12E-20 | 1.10E-18 |
| FLJ13197 | -0.412465761 | 1.42E-22 | 5.38E-21 |
| FLJ14107 | -0.222552499 | 3.35E-07 | 1.27E-06 |
| FLJ22536 | 0.246294333 | 1.49E-08 | 6.92E-08 |
| FLJ23867 | -0.123783479 | 0.004906723 | 0.009232381 |
| FLJ25363 | 0.155599779 | 0.000393881 | 0.000899144 |
| FLJ25758 | 0.178178545 | 4.78E-05 | 0.000127979 |
| FLJ26850 | -0.220313414 | 4.42E-07 | 1.65E-06 |
| FLJ33360 | -0.411634315 | 1.76E-22 | 6.60E-21 |
| FLJ33630 | -0.165239049 | 0.000165431 | 0.000406022 |
| FLJ34503 | -0.250689974 | 8.04E-09 | 3.90E-08 |
| FLJ35220 | -0.139508833 | 0.001504573 | 0.00310099 |
| FLJ35390 | -0.151229891 | 0.000574369 | 0.001274336 |
| FLJ36031 | 0.19118162 | 1.25E-05 | 3.69E-05 |
| FLJ36777 | -0.338724871 | 2.72E-15 | 3.54E-14 |
| FLJ37307 | -0.216989938 | 6.64E-07 | 2.40E-06 |
| FLJ37453 | -0.231276881 | 1.11E-07 | 4.54E-07 |
| FLJ37543 | -0.153598388 | 0.000468742 | 0.001055567 |
| FLJ39609 | -0.143148227 | 0.00112419 | 0.002369536 |
| FLJ39739 | 0.149579613 | 0.000660598 | 0.00145195 |
| FLJ40292 | -0.112855573 | 0.010375106 | 0.018353626 |
| FLJ40852 | -0.186048665 | 2.15E-05 | 6.10E-05 |
| FLJ42289 | -0.251876843 | 6.80E-09 | 3.33E-08 |
| FLJ42627 | -0.185673633 | 2.23E-05 | 6.32E-05 |
| FLJ42709 | -0.158603697 | 0.000302159 | 0.00070534 |
| FLJ42875 | -0.463683085 | 8.15E-29 | 7.47E-27 |
| FLJ43390 | -0.235856226 | 6.09E-08 | 2.59E-07 |
| FLJ43663 | -0.232601303 | 9.34E-08 | 3.86E-07 |
| FLJ43859 | 0.126579068 | 0.004013014 | 0.007661009 |
| FLJ43860 | -0.13268456 | 0.002551842 | 0.005048121 |
| FLJ44606 | -0.27345802 | 2.77E-10 | 1.67E-09 |
| FLJ45079 | -0.166660192 | 0.000144967 | 0.000359415 |
| FLJ45244 | -0.276018582 | 1.86E-10 | 1.15E-09 |
| FLJ45340 | -0.339395371 | 2.38E-15 | 3.13E-14 |
| FLJ45445 | -0.182652684 | 3.04E-05 | 8.43E-05 |
| FLJ46111 | 0.134248922 | 0.002265499 | 0.004523304 |
| FLJ90757 | -0.373571302 | 1.68E-18 | 3.51E-17 |
| FLNA | -0.219771461 | 4.73E-07 | 1.76E-06 |
| FLNC | 0.228884704 | 1.51E-07 | 6.03E-07 |
| FLOT2 | -0.116712644 | 0.008019164 | 0.014504303 |
| FLRT3 | -0.128506803 | 0.003485534 | 0.006725442 |
| FLT3LG | -0.311588954 | 4.66E-13 | 4.28E-12 |
| FLT3 | -0.291932102 | 1.41E-11 | 1.04E-10 |
| FLT4 | -0.140471592 | 0.00139385 | 0.002888271 |
| FLVCR1 | 0.237093656 | 5.17E-08 | 2.22E-07 |
| FLYWCH1 | -0.331303745 | 1.17E-14 | 1.36E-13 |
| FLYWCH2 | -0.14013747 | 0.001431397 | 0.002960245 |
| FMN1 | -0.21379453 | 9.75E-07 | 3.43E-06 |
| FMN2 | -0.148610718 | 0.000716665 | 0.00156537 |
| FMNL1 | -0.098702933 | 0.025093828 | 0.040951619 |
| FMNL2 | -0.197544281 | 6.28E-06 | 1.94E-05 |
| FMNL3 | -0.259116706 | 2.40E-09 | 1.25E-08 |
| FMO2 | -0.302605786 | 2.29E-12 | 1.88E-11 |
| FMO3 | -0.264575309 | 1.07E-09 | 5.92E-09 |
| FMO4 | -0.442869093 | 3.77E-26 | 2.48E-24 |
| FMO5 | -0.237298151 | 5.03E-08 | 2.16E-07 |
| FMO6P | -0.133314982 | 0.002432696 | 0.004830112 |
| FMR1 | -0.116941229 | 0.007895872 | 0.014296828 |
| FN3K | -0.121893135 | 0.005608919 | 0.010444482 |
| FNBP1 | -0.219465296 | 4.91E-07 | 1.82E-06 |
| FNBP4 | -0.289388286 | 2.16E-11 | 1.53E-10 |
| FNDC1 | 0.096223388 | 0.029004991 | 0.046697685 |
| FNDC3B | 0.203254149 | 3.32E-06 | 1.07E-05 |
| FNDC4 | 0.133065357 | 0.002479253 | 0.004913259 |
| FNDC5 | -0.308259425 | 8.46E-13 | 7.49E-12 |
| FNIP1 | -0.132499022 | 0.002587907 | 0.005112382 |
| FNIP2 | -0.324424178 | 4.37E-14 | 4.68E-13 |
| FNTA | 0.230978227 | 1.15E-07 | 4.70E-07 |
| FOLH1B | 0.104354911 | 0.017840772 | 0.030027059 |
| FOLH1 | 0.113249221 | 0.010109296 | 0.017929425 |
| FOLR1 | -0.332638699 | 9.03E-15 | 1.07E-13 |
| FOLR2 | -0.19183085 | 1.17E-05 | 3.46E-05 |
| FOLR4 | -0.140145201 | 0.001430518 | 0.002958733 |
| FOSB | -0.263059339 | 1.34E-09 | 7.29E-09 |
| FOSL1 | 0.294143775 | 9.75E-12 | 7.31E-11 |
| FOS | -0.257194624 | 3.17E-09 | 1.62E-08 |
| FOXA1 | 0.128525658 | 0.003480701 | 0.006717414 |
| FOXA2 | -0.297825847 | 5.22E-12 | 4.06E-11 |
| FOXA3 | 0.096612668 | 0.02835833 | 0.04577825 |
| FOXC1 | -0.137331845 | 0.001785344 | 0.003628784 |
| FOXD4L1 | -0.197811148 | 6.10E-06 | 1.89E-05 |
| FOXD4L2 | -0.209457652 | 1.63E-06 | 5.51E-06 |
| FOXD4L3 | -0.154009655 | 0.000452355 | 0.001022235 |
| FOXD4L5 | -0.134074463 | 0.002295911 | 0.004580364 |
| FOXD4L6 | -0.237272133 | 5.05E-08 | 2.17E-07 |
| FOXD4 | -0.177366499 | 5.18E-05 | 0.000137852 |
| FOXF1 | -0.334116357 | 6.76E-15 | 8.23E-14 |
| FOXF2 | -0.308508427 | 8.10E-13 | 7.20E-12 |
| FOXG1 | 0.149968033 | 0.000639286 | 0.001407273 |
| FOXH1 | 0.096794345 | 0.028060798 | 0.045341894 |
| FOXI1 | -0.192781567 | 1.05E-05 | 3.15E-05 |
| FOXI2 | -0.315358952 | 2.35E-13 | 2.26E-12 |
| FOXJ1 | -0.255743677 | 3.91E-09 | 1.98E-08 |
| FOXJ2 | -0.12637815 | 0.004071954 | 0.007764019 |
| FOXK2 | 0.142277779 | 0.001206093 | 0.00252804 |
| FOXL2 | 0.103525296 | 0.018774854 | 0.031466693 |
| FOXM1 | 0.508047836 | 3.80E-35 | 1.12E-32 |
| FOXN1 | -0.183429976 | 2.81E-05 | 7.82E-05 |
| FOXN2 | 0.156487449 | 0.000364383 | 0.000836582 |
| FOXN3 | -0.30782974 | 9.13E-13 | 8.05E-12 |
| FOXO1 | -0.16979033 | 0.000107972 | 0.000273667 |
| FOXO3B | -0.231896447 | 1.02E-07 | 4.21E-07 |
| FOXO3 | -0.27167714 | 3.64E-10 | 2.16E-09 |
| FOXO4 | -0.275957821 | 1.87E-10 | 1.16E-09 |
| FOXP1 | -0.117896961 | 0.007398515 | 0.013467036 |
| FOXP2 | 0.112833745 | 0.010390025 | 0.018376765 |
| FOXP3 | -0.131856537 | 0.002716415 | 0.005348803 |
| FOXP4 | -0.189513521 | 1.49E-05 | 4.36E-05 |
| FOXQ1 | -0.22512602 | 2.43E-07 | 9.41E-07 |
| FOXR2 | 0.131618467 | 0.00276549 | 0.00543366 |
| FOXS1 | -0.16818904 | 0.000125619 | 0.000314605 |
| FPR3 | -0.114182499 | 0.009503118 | 0.016941519 |
| FRAS1 | -0.196873921 | 6.76E-06 | 2.08E-05 |
| FRAT1 | -0.240488877 | 3.28E-08 | 1.45E-07 |
| FRAT2 | 0.095979417 | 0.029416686 | 0.047314776 |
| FREM1 | -0.232173579 | 9.87E-08 | 4.07E-07 |
| FREM2 | -0.299529563 | 3.90E-12 | 3.08E-11 |
| FRMD4A | -0.285924959 | 3.81E-11 | 2.60E-10 |
| FRMD4B | -0.273710511 | 2.66E-10 | 1.61E-09 |
| FRMD5 | 0.13461265 | 0.002203274 | 0.004409692 |
| FRMD6 | 0.157609769 | 0.000330033 | 0.000763949 |
| FRMPD1 | -0.144223992 | 0.001030058 | 0.002186111 |
| FRMPD4 | -0.194270485 | 8.98E-06 | 2.71E-05 |
| FRS3 | -0.270421185 | 4.42E-10 | 2.59E-09 |
| FRYL | -0.1471881 | 0.000807013 | 0.001745168 |
| FRY | -0.418060752 | 3.33E-23 | 1.41E-21 |
| FRZB | -0.308493838 | 8.12E-13 | 7.22E-12 |
| FSCN1 | 0.181169507 | 3.54E-05 | 9.69E-05 |
| FSCN2 | -0.204058781 | 3.03E-06 | 9.82E-06 |
| FSCN3 | -0.140321964 | 0.001410551 | 0.00292076 |
| FSD1L | 0.11805867 | 0.007317192 | 0.013336011 |
| FSD1 | 0.115986497 | 0.008422308 | 0.015153938 |
| FSD2 | -0.194913103 | 8.38E-06 | 2.54E-05 |
| FSHR | -0.128114537 | 0.003587471 | 0.006902143 |
| FST | 0.207767153 | 1.98E-06 | 6.62E-06 |
| FTHL3 | 0.09845524 | 0.025462905 | 0.041506487 |
| FTO | -0.177732661 | 4.99E-05 | 0.00013334 |
| FTSJ1 | 0.263725949 | 1.21E-09 | 6.66E-09 |
| FTSJD2 | -0.142140327 | 0.001219518 | 0.002554303 |
| FUBP1 | 0.23935857 | 3.82E-08 | 1.67E-07 |
| FUBP3 | 0.222725273 | 3.28E-07 | 1.25E-06 |
| FUCA1 | -0.402191707 | 1.92E-21 | 6.29E-20 |
| FUCA2 | 0.336342131 | 4.37E-15 | 5.48E-14 |
| FUK | -0.259040751 | 2.43E-09 | 1.26E-08 |
| FUNDC2P2 | 0.184640549 | 2.48E-05 | 6.98E-05 |
| FUNDC2 | 0.163984949 | 0.000185711 | 0.000451856 |
| FURIN | 0.234943444 | 6.87E-08 | 2.90E-07 |
| FUT1 | -0.135770793 | 0.002015403 | 0.004063743 |
| FUT3 | -0.145669983 | 0.000914969 | 0.001958034 |
| FUT4 | 0.096232139 | 0.028990316 | 0.04668534 |
| FUT7 | -0.259394751 | 2.30E-09 | 1.21E-08 |
| FUZ | -0.126441472 | 0.004053294 | 0.007731391 |
| FXC1 | -0.120781357 | 0.006062934 | 0.011203283 |
| FXN | 0.122434443 | 0.005399137 | 0.010085789 |
| FXR1 | 0.201911467 | 3.86E-06 | 1.23E-05 |
| FXYD1 | -0.426324511 | 3.70E-24 | 1.83E-22 |
| FXYD4 | 0.129051899 | 0.003348244 | 0.006485579 |
| FXYD5 | -0.162499519 | 0.000212743 | 0.000512711 |
| FXYD6 | -0.262936068 | 1.37E-09 | 7.41E-09 |
| FXYD7 | -0.301855206 | 2.61E-12 | 2.12E-11 |
| FYB | -0.103059225 | 0.019318103 | 0.032287837 |
| FYCO1 | -0.371643778 | 2.59E-18 | 5.26E-17 |
| FYN | -0.266051987 | 8.57E-10 | 4.81E-09 |
| FYTTD1 | 0.223981608 | 2.81E-07 | 1.08E-06 |
| FZD10 | 0.178534704 | 4.61E-05 | 0.000123802 |
| FZD1 | -0.205200134 | 2.66E-06 | 8.70E-06 |
| FZD2 | -0.134760608 | 0.002178412 | 0.004365561 |
| FZD3 | 0.097918942 | 0.026278096 | 0.042720341 |
| FZD4 | -0.095797919 | 0.029726203 | 0.047789539 |
| FZD6 | 0.108100142 | 0.014111294 | 0.024290092 |
| FZD7 | -0.122048945 | 0.005547796 | 0.01033837 |
| FZD8 | -0.218650703 | 5.42E-07 | 1.99E-06 |
| G2E3 | 0.415905779 | 5.85E-23 | 2.38E-21 |
| G3BP1 | 0.329380577 | 1.70E-14 | 1.94E-13 |
| G3BP2 | 0.186030585 | 2.15E-05 | 6.11E-05 |
| G6PC | 0.159056495 | 0.000290204 | 0.000680024 |
| G6PD | 0.215427338 | 8.01E-07 | 2.85E-06 |
| GAA | -0.135815082 | 0.002008519 | 0.004051497 |
| GAB1 | -0.331535495 | 1.12E-14 | 1.31E-13 |
| GAB2 | -0.162315752 | 0.000216332 | 0.000520281 |
| GAB3 | -0.390987941 | 2.94E-20 | 8.00E-19 |
| GABBR1 | -0.339712294 | 2.24E-15 | 2.94E-14 |
| GABBR2 | -0.192711152 | 1.06E-05 | 3.17E-05 |
| GABPB1 | 0.418225479 | 3.19E-23 | 1.36E-21 |
| GABRA2 | 0.104587698 | 0.017586078 | 0.029630828 |
| GABRA3 | 0.163040805 | 0.000202492 | 0.000489647 |
| GABRA4 | -0.133129146 | 0.002467279 | 0.004891865 |
| GABRA5 | 0.130574767 | 0.002990309 | 0.005840937 |
| GABRB2 | -0.123747304 | 0.00491938 | 0.009251842 |
| GABRE | -0.309462103 | 6.83E-13 | 6.13E-12 |
| GABRG2 | 0.153927263 | 0.000455594 | 0.001028974 |
| GABRP | -0.167080584 | 0.000139385 | 0.000346434 |
| GABRQ | 0.174194743 | 7.07E-05 | 0.000184095 |
| GABRR2 | -0.207912835 | 1.95E-06 | 6.52E-06 |
| GADD45B | -0.149348081 | 0.000673614 | 0.001478282 |
| GADD45GIP1 | 0.175685506 | 6.11E-05 | 0.000160517 |
| GADD45G | -0.172397112 | 8.41E-05 | 0.000216284 |
| GADL1 | -0.154065348 | 0.000450177 | 0.001017889 |
| GAL3ST1 | -0.106301704 | 0.015806667 | 0.026897978 |
| GALC | -0.15365645 | 0.000466395 | 0.001051111 |
| GALK2 | 0.168867055 | 0.000117839 | 0.000296384 |
| GALNT10 | -0.201528554 | 4.03E-06 | 1.28E-05 |
| GALNT11 | -0.254465748 | 4.70E-09 | 2.35E-08 |
| GALNT12 | -0.175801328 | 6.04E-05 | 0.000158911 |
| GALNT14 | 0.143254357 | 0.001114563 | 0.00235157 |
| GALNT1 | 0.133871621 | 0.002331739 | 0.004644887 |
| GALNT2 | 0.215940751 | 7.53E-07 | 2.69E-06 |
| GALNT4 | 0.284356564 | 4.91E-11 | 3.30E-10 |
| GALNT5 | -0.146859262 | 0.000829345 | 0.001789008 |
| GALNT6 | 0.106819614 | 0.015301017 | 0.02612203 |
| GALNT7 | 0.151933064 | 0.000540905 | 0.001205039 |
| GALNTL1 | -0.362282168 | 2.04E-17 | 3.61E-16 |
| GALNTL4 | -0.321174375 | 8.04E-14 | 8.24E-13 |
| GALP | 0.099335681 | 0.024171896 | 0.039576331 |
| GALR3 | 0.120382839 | 0.006233544 | 0.011497284 |
| GALT | -0.21441477 | 9.05E-07 | 3.20E-06 |
| GAL | 0.414777967 | 7.84E-23 | 3.11E-21 |
| GANAB | 0.192414977 | 1.10E-05 | 3.26E-05 |
| GANC | -0.384836249 | 1.26E-19 | 3.15E-18 |
| GAN | 0.224827588 | 2.52E-07 | 9.75E-07 |
| GAP43 | 0.131682448 | 0.002752223 | 0.005412912 |
| GAPDHS | -0.15715367 | 0.000343611 | 0.000793266 |
| GAPDH | 0.463787079 | 7.90E-29 | 7.27E-27 |
| GAPT | -0.341246063 | 1.64E-15 | 2.21E-14 |
| GAPVD1 | -0.144941898 | 0.000971345 | 0.002070076 |
| GAR1 | 0.308372716 | 8.29E-13 | 7.36E-12 |
| GARNL3 | -0.329780444 | 1.57E-14 | 1.81E-13 |
| GARS | 0.43502657 | 3.42E-25 | 1.97E-23 |
| GART | 0.360500583 | 2.99E-17 | 5.21E-16 |
| GAS2L2 | -0.397394081 | 6.25E-21 | 1.87E-19 |
| GAS2L3 | 0.274545769 | 2.34E-10 | 1.43E-09 |
| GAS5 | 0.120395275 | 0.006228155 | 0.011488405 |
| GAS6 | -0.339064366 | 2.54E-15 | 3.32E-14 |
| GAS7 | -0.299662891 | 3.81E-12 | 3.02E-11 |
| GAS8 | -0.222855222 | 3.23E-07 | 1.23E-06 |
| GAST | 0.222753178 | 3.27E-07 | 1.24E-06 |
| GATA1 | -0.377950727 | 6.22E-19 | 1.39E-17 |
| GATA2 | -0.204413219 | 2.91E-06 | 9.46E-06 |
| GATA3 | -0.111086106 | 0.011648051 | 0.020376443 |
| GATA5 | -0.380920855 | 3.14E-19 | 7.38E-18 |
| GATA6 | -0.328140533 | 2.15E-14 | 2.41E-13 |
| GATAD2B | -0.164289039 | 0.00018059 | 0.000440522 |
| GATC | 0.313801428 | 3.13E-13 | 2.95E-12 |
| GATM | -0.110837155 | 0.01183781 | 0.020664991 |
| GATSL1 | 0.204441277 | 2.90E-06 | 9.44E-06 |
| GATSL2 | 0.11991944 | 0.00643734 | 0.011843662 |
| GATSL3 | -0.204517728 | 2.88E-06 | 9.36E-06 |
| GBA2 | -0.164539948 | 0.000176465 | 0.000431565 |
| GBA3 | -0.09542187 | 0.03037639 | 0.048705542 |
| GBAS | 0.162492819 | 0.000212873 | 0.000512948 |
| GBE1 | 0.179535628 | 4.17E-05 | 0.000112775 |
| GBGT1 | -0.123122328 | 0.005142751 | 0.009643804 |
| GBP1 | 0.106869537 | 0.015253035 | 0.026050289 |
| GBX2 | 0.272575889 | 3.17E-10 | 1.89E-09 |
| GCC2 | -0.234112205 | 7.67E-08 | 3.21E-07 |
| GCDH | -0.176919016 | 5.41E-05 | 0.000143346 |
| GCET2 | -0.363948625 | 1.42E-17 | 2.58E-16 |
| GCFC1 | -0.194398614 | 8.86E-06 | 2.67E-05 |
| GCH1 | 0.337439719 | 3.51E-15 | 4.48E-14 |
| GCHFR | -0.098092804 | 0.026011391 | 0.042324623 |
| GCK | -0.104831409 | 0.017322841 | 0.029211925 |
| GCLC | 0.289093488 | 2.26E-11 | 1.60E-10 |
| GCLM | 0.330570659 | 1.35E-14 | 1.56E-13 |
| GCN1L1 | 0.194010991 | 9.24E-06 | 2.78E-05 |
| GCNT4 | -0.307351568 | 9.94E-13 | 8.72E-12 |
| GCNT7 | -0.236780366 | 5.39E-08 | 2.30E-07 |
| GCOM1 | -0.180400738 | 3.82E-05 | 0.000104103 |
| GCSH | 0.300759383 | 3.15E-12 | 2.54E-11 |
| GDAP1L1 | -0.17830721 | 4.72E-05 | 0.000126401 |
| GDE1 | -0.187076853 | 1.93E-05 | 5.53E-05 |
| GDF10 | -0.30253472 | 2.32E-12 | 1.90E-11 |
| GDF15 | -0.327813676 | 2.29E-14 | 2.56E-13 |
| GDF1 | 0.104357483 | 0.01783794 | 0.030024821 |
| GDF2 | -0.147780489 | 0.000768182 | 0.001668052 |
| GDF3 | -0.106990699 | 0.015137131 | 0.025882479 |
| GDF5 | -0.239604451 | 3.70E-08 | 1.62E-07 |
| GDF7 | -0.257528962 | 3.02E-09 | 1.55E-08 |
| GDF9 | -0.169238362 | 0.000113773 | 0.000286843 |
| GDI2 | 0.34419358 | 9.06E-16 | 1.26E-14 |
| GDPD5 | -0.260368376 | 2.00E-09 | 1.05E-08 |
| GEFT | -0.117759011 | 0.007468528 | 0.013577166 |
| GEMIN6 | 0.352241921 | 1.73E-16 | 2.66E-15 |
| GEMIN7 | 0.306494189 | 1.16E-12 | 1.00E-11 |
| GEMIN8P4 | 0.11223055 | 0.010809902 | 0.019058686 |
| GEMIN8 | -0.177340973 | 5.19E-05 | 0.000138146 |
| GEN1 | 0.212416415 | 1.15E-06 | 3.98E-06 |
| GFI1B | -0.3181882 | 1.40E-13 | 1.39E-12 |
| GFM1 | 0.282147022 | 7.02E-11 | 4.62E-10 |
| GFM2 | 0.171869308 | 8.85E-05 | 0.000226939 |
| GFOD1 | -0.153723426 | 0.000463702 | 0.001046103 |
| GFPT1 | 0.162447083 | 0.000213762 | 0.000514903 |
| GFPT2 | 0.1780379 | 4.84E-05 | 0.000129694 |
| GFRA1 | -0.472871294 | 4.73E-30 | 5.31E-28 |
| GFRA2 | -0.303294606 | 2.03E-12 | 1.69E-11 |
| GFRA3 | -0.145776148 | 0.000907005 | 0.001941862 |
| GGA1 | -0.232655711 | 9.27E-08 | 3.84E-07 |
| GGA2 | -0.536533662 | 9.92E-40 | 7.94E-37 |
| GGA3 | -0.296298728 | 6.77E-12 | 5.20E-11 |
| GGCT | 0.105510085 | 0.016607865 | 0.028136803 |
| GGCX | -0.099725111 | 0.023619162 | 0.038747521 |
| GGH | 0.414387934 | 8.67E-23 | 3.43E-21 |
| GGNBP2 | -0.109191683 | 0.013162218 | 0.022786411 |
| GGPS1 | 0.120043768 | 0.006382084 | 0.011748488 |
| GGT1 | -0.241334648 | 2.93E-08 | 1.30E-07 |
| GGT3P | -0.399356069 | 3.86E-21 | 1.21E-19 |
| GGT5 | -0.279607051 | 1.05E-10 | 6.76E-10 |
| GGT6 | -0.367716734 | 6.21E-18 | 1.20E-16 |
| GGT7 | -0.303821839 | 1.85E-12 | 1.55E-11 |
| GGT8P | -0.276735989 | 1.66E-10 | 1.03E-09 |
| GGTA1 | -0.262276197 | 1.51E-09 | 8.14E-09 |
| GGTLC1 | -0.444300964 | 2.51E-26 | 1.71E-24 |
| GGTLC2 | -0.256843586 | 3.34E-09 | 1.70E-08 |
| GH2 | 0.127676633 | 0.003704456 | 0.007111474 |
| GHDC | -0.310620713 | 5.55E-13 | 5.06E-12 |
| GHITM | 0.313127039 | 3.53E-13 | 3.31E-12 |
| GHRLOS | -0.201706324 | 3.95E-06 | 1.26E-05 |
| GHRL | -0.357632095 | 5.53E-17 | 9.21E-16 |
| GHR | -0.340512703 | 1.90E-15 | 2.53E-14 |
| GIGYF1 | -0.288738565 | 2.40E-11 | 1.69E-10 |
| GIGYF2 | -0.146274053 | 0.000870506 | 0.00187033 |
| GIMAP1 | -0.294049355 | 9.90E-12 | 7.41E-11 |
| GIMAP2 | -0.161611788 | 0.000230613 | 0.000551507 |
| GIMAP4 | -0.149546639 | 0.000662437 | 0.001455673 |
| GIMAP5 | -0.145351018 | 0.000939283 | 0.002006026 |
| GIMAP6 | -0.184555326 | 2.51E-05 | 7.03E-05 |
| GIMAP7 | -0.212977492 | 1.07E-06 | 3.75E-06 |
| GIMAP8 | -0.26595025 | 8.71E-10 | 4.88E-09 |
| GINS1 | 0.45954514 | 2.86E-28 | 2.41E-26 |
| GINS2 | 0.431433411 | 9.22E-25 | 4.94E-23 |
| GINS3 | 0.419859805 | 2.07E-23 | 9.03E-22 |
| GINS4 | 0.391549734 | 2.57E-20 | 7.06E-19 |
| GIPC2 | -0.134261513 | 0.002263319 | 0.004519402 |
| GIPR | -0.395629997 | 9.61E-21 | 2.80E-19 |
| GIP | 0.25070901 | 8.02E-09 | 3.89E-08 |
| GIT2 | -0.172960064 | 7.97E-05 | 0.000205667 |
| GJA4 | -0.129773834 | 0.003173973 | 0.006171339 |
| GJB1 | -0.204328391 | 2.94E-06 | 9.55E-06 |
| GJB2 | 0.240561091 | 3.25E-08 | 1.44E-07 |
| GJB3 | 0.184406724 | 2.54E-05 | 7.14E-05 |
| GJC1 | 0.122525566 | 0.005364525 | 0.010025818 |
| GJC2 | -0.233167726 | 8.67E-08 | 3.61E-07 |
| GK5 | -0.11142299 | 0.011395552 | 0.019995987 |
| GKAP1 | -0.095529738 | 0.030188652 | 0.048458945 |
| GKN2 | -0.171735603 | 8.97E-05 | 0.000229724 |
| GLA | 0.289321259 | 2.18E-11 | 1.55E-10 |
| GLB1L2 | -0.285606474 | 4.01E-11 | 2.74E-10 |
| GLB1L3 | -0.356827403 | 6.56E-17 | 1.08E-15 |
| GLB1L | -0.252846939 | 5.92E-09 | 2.93E-08 |
| GLCCI1 | -0.278262844 | 1.30E-10 | 8.25E-10 |
| GLCE | 0.18468204 | 2.47E-05 | 6.95E-05 |
| GLDC | 0.309844829 | 6.38E-13 | 5.76E-12 |
| GLDN | -0.235624853 | 6.28E-08 | 2.66E-07 |
| GLI1 | -0.244036488 | 2.03E-08 | 9.27E-08 |
| GLI3 | -0.148577239 | 0.000718679 | 0.001569598 |
| GLIPR1L2 | -0.253037081 | 5.77E-09 | 2.85E-08 |
| GLIPR2 | -0.158825384 | 0.00029625 | 0.000692566 |
| GLIS2 | -0.239889739 | 3.56E-08 | 1.57E-07 |
| GLIS3 | -0.095749822 | 0.029808691 | 0.047899037 |
| GLMN | 0.225660891 | 2.27E-07 | 8.84E-07 |
| GLO1 | 0.113370748 | 0.010028466 | 0.017804111 |
| GLOD4 | 0.16398692 | 0.000185678 | 0.00045183 |
| GLOD5 | -0.266619716 | 7.87E-10 | 4.44E-09 |
| GLP1R | -0.288081222 | 2.67E-11 | 1.87E-10 |
| GLRX2 | 0.328599583 | 1.97E-14 | 2.23E-13 |
| GLRX3 | 0.426212902 | 3.81E-24 | 1.88E-22 |
| GLRX5 | 0.357688324 | 5.47E-17 | 9.11E-16 |
| GLS2 | -0.316491602 | 1.91E-13 | 1.86E-12 |
| GLT25D1 | 0.142938859 | 0.001143408 | 0.002406997 |
| GLT25D2 | -0.293474977 | 1.09E-11 | 8.11E-11 |
| GLTSCR1 | -0.127649525 | 0.00371181 | 0.00712354 |
| GLTSCR2 | -0.237988219 | 4.59E-08 | 1.99E-07 |
| GLUD1 | 0.21452027 | 8.94E-07 | 3.16E-06 |
| GLUD2 | 0.187309286 | 1.88E-05 | 5.40E-05 |
| GLUL | -0.166017082 | 0.000153914 | 0.000379667 |
| GLYATL1 | 0.094972638 | 0.031169043 | 0.049828587 |
| GLYATL2 | -0.15190505 | 0.000542203 | 0.001207392 |
| GLYR1 | -0.237710349 | 4.77E-08 | 2.06E-07 |
| GM2A | -0.137552845 | 0.001754794 | 0.003570684 |
| GMCL1 | 0.150223607 | 0.000625612 | 0.001379452 |
| GMDS | 0.20893759 | 1.73E-06 | 5.84E-06 |
| GMEB1 | 0.124135169 | 0.004785193 | 0.009024377 |
| GMFB | 0.494124447 | 4.65E-33 | 8.86E-31 |
| GMFG | -0.188810304 | 1.61E-05 | 4.67E-05 |
| GMIP | -0.295047485 | 8.37E-12 | 6.35E-11 |
| GMNN | 0.394629572 | 1.23E-20 | 3.49E-19 |
| GMPPA | 0.152064313 | 0.000534863 | 0.001192908 |
| GMPR2 | 0.11071849 | 0.011929219 | 0.020817289 |
| GMPR | -0.33226974 | 9.70E-15 | 1.14E-13 |
| GMPS | 0.380503424 | 3.46E-19 | 8.07E-18 |
| GNA11 | -0.102586751 | 0.019882745 | 0.033154378 |
| GNA13 | 0.142562985 | 0.001178671 | 0.002475233 |
| GNA14 | -0.258686872 | 2.55E-09 | 1.33E-08 |
| GNAI2 | -0.226789404 | 1.97E-07 | 7.75E-07 |
| GNAI3 | 0.297028595 | 5.98E-12 | 4.62E-11 |
| GNAO1 | -0.341418613 | 1.59E-15 | 2.14E-14 |
| GNAQ | -0.252624824 | 6.11E-09 | 3.01E-08 |
| GNAT1 | 0.126013483 | 0.004180936 | 0.007955121 |
| GNAZ | -0.188001627 | 1.75E-05 | 5.06E-05 |
| GNB1L | 0.103452403 | 0.018858928 | 0.031583766 |
| GNB1 | 0.174095361 | 7.14E-05 | 0.000185714 |
| GNB3 | -0.152035459 | 0.000536186 | 0.001195325 |
| GNG10 | 0.129877779 | 0.003149572 | 0.006126279 |
| GNG2 | -0.207386016 | 2.07E-06 | 6.89E-06 |
| GNG4 | 0.27397606 | 2.55E-10 | 1.55E-09 |
| GNG5 | 0.128013586 | 0.003614139 | 0.006948101 |
| GNG7 | -0.45576775 | 8.84E-28 | 7.16E-26 |
| GNGT1 | 0.177745698 | 4.99E-05 | 0.00013321 |
| GNGT2 | -0.142537164 | 0.00118113 | 0.002479876 |
| GNL2 | 0.095688968 | 0.029913339 | 0.048059467 |
| GNL3L | 0.105632589 | 0.016481599 | 0.027939463 |
| GNL3 | 0.380018828 | 3.87E-19 | 8.97E-18 |
| GNMT | -0.333883853 | 7.08E-15 | 8.57E-14 |
| GNPAT | 0.1657959 | 0.000157109 | 0.000387118 |
| GNPNAT1 | 1 | 1.00E-54 | 1.00E-50 |
| GNPTAB | -0.203423427 | 3.26E-06 | 1.05E-05 |
| GNPTG | -0.239903901 | 3.55E-08 | 1.56E-07 |
| GNRH1 | -0.317212676 | 1.68E-13 | 1.64E-12 |
| GNRHR2 | -0.299142 | 4.17E-12 | 3.28E-11 |
| GNRHR | -0.179851808 | 4.04E-05 | 0.000109551 |
| GOLGA1 | -0.152646372 | 0.000508816 | 0.001139048 |
| GOLGA2B | -0.404177885 | 1.17E-21 | 3.93E-20 |
| GOLGA5 | 0.364794624 | 1.18E-17 | 2.17E-16 |
| GOLGA6B | -0.119702934 | 0.006534589 | 0.012008215 |
| GOLGA6L10 | -0.328460193 | 2.02E-14 | 2.28E-13 |
| GOLGA6L5 | -0.252711542 | 6.04E-09 | 2.98E-08 |
| GOLGA6L9 | -0.358572659 | 4.53E-17 | 7.62E-16 |
| GOLGA7 | 0.206831136 | 2.21E-06 | 7.31E-06 |
| GOLGA8A | -0.340411663 | 1.94E-15 | 2.58E-14 |
| GOLGA8B | -0.30626226 | 1.21E-12 | 1.04E-11 |
| GOLGA8C | 0.117913447 | 0.007390188 | 0.013454997 |
| GOLGA9P | -0.192031298 | 1.14E-05 | 3.39E-05 |
| GOLGB1 | -0.233180151 | 8.66E-08 | 3.60E-07 |
| GOLM1 | 0.232768953 | 9.14E-08 | 3.79E-07 |
| GOLT1A | -0.115365812 | 0.008781126 | 0.015740036 |
| GOLT1B | 0.423937729 | 7.02E-24 | 3.29E-22 |
| GON4L | -0.142376903 | 0.001196496 | 0.00250924 |
| GORASP1 | -0.227464408 | 1.81E-07 | 7.15E-07 |
| GORASP2 | 0.225856857 | 2.22E-07 | 8.63E-07 |
| GOSR2 | 0.135513564 | 0.002055817 | 0.004140218 |
| GOT1L1 | 0.102991144 | 0.019398593 | 0.032408816 |
| GOT1 | 0.246932558 | 1.36E-08 | 6.37E-08 |
| GOT2 | 0.29692438 | 6.09E-12 | 4.69E-11 |
| GP1BA | -0.350855315 | 2.30E-16 | 3.47E-15 |
| GP5 | -0.207065543 | 2.15E-06 | 7.13E-06 |
| GP6 | -0.182559433 | 3.07E-05 | 8.50E-05 |
| GP9 | -0.151238238 | 0.000573961 | 0.001273572 |
| GPA33 | -0.147367138 | 0.00079509 | 0.001721432 |
| GPATCH2 | 0.165569062 | 0.000160451 | 0.00039467 |
| GPATCH3 | -0.118534219 | 0.007082652 | 0.01293095 |
| GPATCH4 | 0.198135669 | 5.88E-06 | 1.83E-05 |
| GPATCH8 | -0.138291454 | 0.001656137 | 0.003385789 |
| GPBAR1 | -0.211294543 | 1.31E-06 | 4.51E-06 |
| GPBP1L1 | -0.213137116 | 1.05E-06 | 3.68E-06 |
| GPC3 | -0.299024521 | 4.25E-12 | 3.35E-11 |
| GPC4 | -0.430189701 | 1.30E-24 | 6.71E-23 |
| GPC5 | -0.194783275 | 8.50E-06 | 2.57E-05 |
| GPC6 | 0.350842119 | 2.31E-16 | 3.47E-15 |
| GPCPD1 | -0.154399295 | 0.000437323 | 0.000991741 |
| GPD1L | -0.376174226 | 9.33E-19 | 2.04E-17 |
| GPD1 | -0.285151207 | 4.32E-11 | 2.93E-10 |
| GPD2 | 0.207641904 | 2.01E-06 | 6.70E-06 |
| GPER | -0.225169181 | 2.42E-07 | 9.36E-07 |
| GPHN | 0.182387218 | 3.13E-05 | 8.63E-05 |
| GPIHBP1 | -0.328839596 | 1.88E-14 | 2.14E-13 |
| GPI | 0.313785725 | 3.13E-13 | 2.96E-12 |
| GPLD1 | -0.297816375 | 5.23E-12 | 4.07E-11 |
| GPM6A | -0.232954173 | 8.92E-08 | 3.70E-07 |
| GPM6B | -0.103895958 | 0.018352372 | 0.030820454 |
| GPN1 | 0.388165746 | 5.76E-20 | 1.49E-18 |
| GPN3 | 0.358387814 | 4.71E-17 | 7.90E-16 |
| GPNMB | -0.199948123 | 4.81E-06 | 1.51E-05 |
| GPR108 | -0.284326903 | 4.94E-11 | 3.32E-10 |
| GPR110 | -0.254803806 | 4.48E-09 | 2.24E-08 |
| GPR112 | -0.236895823 | 5.31E-08 | 2.27E-07 |
| GPR113 | -0.212878223 | 1.09E-06 | 3.79E-06 |
| GPR114 | -0.149411673 | 0.000670015 | 0.001471354 |
| GPR115 | 0.273833754 | 2.61E-10 | 1.58E-09 |
| GPR116 | -0.481223781 | 3.30E-31 | 4.55E-29 |
| GPR119 | 0.108344371 | 0.013893864 | 0.023936438 |
| GPR120 | -0.258157432 | 2.76E-09 | 1.42E-08 |
| GPR123 | -0.218035344 | 5.85E-07 | 2.14E-06 |
| GPR124 | -0.146377859 | 0.00086307 | 0.001855749 |
| GPR12 | -0.261017846 | 1.81E-09 | 9.66E-09 |
| GPR132 | -0.265341239 | 9.54E-10 | 5.33E-09 |
| GPR133 | -0.288862122 | 2.35E-11 | 1.66E-10 |
| GPR137C | 0.156126784 | 0.000376111 | 0.000860839 |
| GPR137 | -0.176957929 | 5.39E-05 | 0.000142853 |
| GPR143 | -0.209448354 | 1.63E-06 | 5.52E-06 |
| GPR146 | -0.420308405 | 1.84E-23 | 8.08E-22 |
| GPR155 | -0.223440217 | 3.00E-07 | 1.15E-06 |
| GPR160 | -0.226215739 | 2.12E-07 | 8.28E-07 |
| GPR162 | -0.314830757 | 2.59E-13 | 2.48E-12 |
| GPR171 | -0.153396639 | 0.000476981 | 0.001073032 |
| GPR172A | 0.258816046 | 2.51E-09 | 1.30E-08 |
| GPR172B | -0.337043572 | 3.80E-15 | 4.82E-14 |
| GPR173 | -0.149474985 | 0.000666451 | 0.001464009 |
| GPR174 | -0.154529213 | 0.000432415 | 0.000981391 |
| GPR176 | 0.139031633 | 0.001562398 | 0.0032073 |
| GPR179 | -0.220301589 | 4.43E-07 | 1.65E-06 |
| GPR17 | -0.334671588 | 6.07E-15 | 7.45E-14 |
| GPR180 | 0.305548574 | 1.37E-12 | 1.17E-11 |
| GPR182 | -0.133452536 | 0.002407384 | 0.004785558 |
| GPR183 | -0.168369289 | 0.000123505 | 0.000309621 |
| GPR18 | -0.160161643 | 0.000262855 | 0.000620959 |
| GPR19 | 0.299843239 | 3.69E-12 | 2.94E-11 |
| GPR1 | 0.167241073 | 0.000137308 | 0.000341483 |
| GPR20 | -0.161991528 | 0.000222804 | 0.000534429 |
| GPR25 | -0.14901765 | 0.000692601 | 0.001517451 |
| GPR27 | -0.139638765 | 0.001489172 | 0.003070515 |
| GPR31 | -0.131902203 | 0.002707092 | 0.005334651 |
| GPR34 | -0.144967053 | 0.000969345 | 0.002066474 |
| GPR35 | 0.178311989 | 4.71E-05 | 0.000126358 |
| GPR37L1 | 0.218550409 | 5.49E-07 | 2.02E-06 |
| GPR37 | 0.114990892 | 0.009004407 | 0.016108483 |
| GPR44 | -0.35242511 | 1.66E-16 | 2.56E-15 |
| GPR45 | -0.123458406 | 0.005021524 | 0.009432405 |
| GPR55 | -0.29091395 | 1.67E-11 | 1.21E-10 |
| GPR62 | -0.12083421 | 0.006040624 | 0.011168255 |
| GPR63 | 0.115647424 | 0.008616672 | 0.015463282 |
| GPR64 | -0.229896631 | 1.33E-07 | 5.36E-07 |
| GPR65 | -0.153569855 | 0.000469899 | 0.001057934 |
| GPR68 | -0.141299739 | 0.001304655 | 0.002717816 |
| GPR75 | -0.193992829 | 9.25E-06 | 2.78E-05 |
| GPR78 | 0.181034529 | 3.59E-05 | 9.81E-05 |
| GPR82 | -0.160912366 | 0.000245671 | 0.000583819 |
| GPR85 | -0.220037823 | 4.58E-07 | 1.70E-06 |
| GPR89A | 0.151255827 | 0.000573102 | 0.001271806 |
| GPR97 | 0.264867848 | 1.02E-09 | 5.68E-09 |
| GPR98 | -0.213983965 | 9.53E-07 | 3.36E-06 |
| GPRASP1 | -0.483345619 | 1.66E-31 | 2.38E-29 |
| GPRASP2 | -0.254824455 | 4.47E-09 | 2.24E-08 |
| GPRC5C | -0.271885644 | 3.53E-10 | 2.09E-09 |
| GPRIN1 | 0.144900889 | 0.000974614 | 0.002076378 |
| GPRIN2 | -0.304409784 | 1.67E-12 | 1.41E-11 |
| GPS1 | 0.107080577 | 0.015051654 | 0.025764534 |
| GPSM1 | -0.178833167 | 4.48E-05 | 0.000120413 |
| GPSM2 | 0.161053993 | 0.00024255 | 0.000576878 |
| GPSM3 | -0.237089187 | 5.18E-08 | 2.22E-07 |
| GPT2 | 0.207711595 | 1.99E-06 | 6.66E-06 |
| GPX2 | 0.160535258 | 0.000254167 | 0.000602216 |
| GPX3 | -0.1606725 | 0.000251044 | 0.000595663 |
| GPX8 | 0.214407816 | 9.06E-07 | 3.20E-06 |
| GRAMD1B | 0.128129632 | 0.003583499 | 0.006895829 |
| GRAMD2 | -0.299386907 | 3.99E-12 | 3.16E-11 |
| GRAMD3 | -0.203356845 | 3.28E-06 | 1.06E-05 |
| GRAMD4 | -0.12871201 | 0.003433261 | 0.006631762 |
| GRAP2 | -0.222026469 | 3.58E-07 | 1.35E-06 |
| GRAPL | -0.161425736 | 0.000234532 | 0.000559874 |
| GRAP | -0.143544997 | 0.001088585 | 0.002300798 |
| GRASP | -0.353454381 | 1.34E-16 | 2.09E-15 |
| GRB10 | 0.102743181 | 0.019694229 | 0.032872505 |
| GRB7 | -0.162659301 | 0.000209668 | 0.00050553 |
| GREB1L | 0.235206254 | 6.64E-08 | 2.81E-07 |
| GREB1 | -0.162353605 | 0.000215588 | 0.000518741 |
| GREM1 | 0.273740557 | 2.65E-10 | 1.60E-09 |
| GREM2 | -0.148425757 | 0.000727857 | 0.001588428 |
| GRHL1 | -0.186093111 | 2.14E-05 | 6.08E-05 |
| GRHL3 | -0.115995892 | 0.008416979 | 0.015147943 |
| GRHPR | 0.174427474 | 6.91E-05 | 0.000180308 |
| GRIA1 | -0.362912449 | 1.78E-17 | 3.18E-16 |
| GRIA3 | -0.128533742 | 0.003478631 | 0.006714066 |
| GRIA4 | -0.109666211 | 0.012767487 | 0.022148631 |
| GRID1 | -0.321579444 | 7.45E-14 | 7.71E-13 |
| GRID2IP | -0.297918455 | 5.14E-12 | 4.00E-11 |
| GRIK3 | -0.217566688 | 6.19E-07 | 2.25E-06 |
| GRIK4 | -0.275780604 | 1.93E-10 | 1.19E-09 |
| GRIN2A | -0.19492818 | 8.36E-06 | 2.53E-05 |
| GRIN2C | -0.193579033 | 9.68E-06 | 2.90E-05 |
| GRIN2D | 0.217217197 | 6.46E-07 | 2.34E-06 |
| GRIN3B | -0.095491074 | 0.030255829 | 0.048547284 |
| GRINL1A | 0.184942621 | 2.41E-05 | 6.78E-05 |
| GRIP1 | -0.110123043 | 0.012397383 | 0.021558977 |
| GRIPAP1 | -0.205432942 | 2.59E-06 | 8.49E-06 |
| GRK4 | 0.120376772 | 0.006236174 | 0.011501075 |
| GRK5 | -0.151363683 | 0.000567858 | 0.001260868 |
| GRK6 | -0.105879549 | 0.016229609 | 0.027544997 |
| GRLF1 | -0.20587369 | 2.46E-06 | 8.11E-06 |
| GRM2 | -0.156494511 | 0.000364157 | 0.000836159 |
| GRM3 | -0.251906763 | 6.77E-09 | 3.31E-08 |
| GRM6 | -0.296995953 | 6.01E-12 | 4.64E-11 |
| GRM7 | -0.184403834 | 2.54E-05 | 7.14E-05 |
| GRM8 | 0.163594352 | 0.000192489 | 0.00046687 |
| GRN | -0.30905795 | 7.34E-13 | 6.57E-12 |
| GRPEL1 | 0.318200943 | 1.40E-13 | 1.39E-12 |
| GRPEL2 | 0.197263775 | 6.48E-06 | 2.00E-05 |
| GRP | -0.124927065 | 0.004521432 | 0.008554939 |
| GRRP1 | -0.308746268 | 7.76E-13 | 6.91E-12 |
| GRSF1 | 0.395177428 | 1.07E-20 | 3.09E-19 |
| GRWD1 | 0.190291876 | 1.38E-05 | 4.04E-05 |
| GSDMB | -0.257714922 | 2.94E-09 | 1.51E-08 |
| GSDMD | -0.140212228 | 0.001422916 | 0.002944096 |
| GSG1L | -0.28868743 | 2.42E-11 | 1.70E-10 |
| GSG2 | 0.412679055 | 1.35E-22 | 5.14E-21 |
| GSK3A | 0.184017475 | 2.65E-05 | 7.41E-05 |
| GSN | -0.392826328 | 1.89E-20 | 5.25E-19 |
| GSPT2 | -0.22544042 | 2.34E-07 | 9.07E-07 |
| GSR | 0.231029645 | 1.15E-07 | 4.67E-07 |
| GSS | 0.182651632 | 3.04E-05 | 8.43E-05 |
| GSTA3 | -0.22286768 | 3.23E-07 | 1.23E-06 |
| GSTA4 | -0.13778999 | 0.001722546 | 0.003512216 |
| GSTA5 | -0.129755402 | 0.003178318 | 0.006178586 |
| GSTCD | 0.35543833 | 8.81E-17 | 1.41E-15 |
| GSTK1 | -0.117346143 | 0.007681622 | 0.013935403 |
| GSTM2 | -0.293604671 | 1.07E-11 | 7.95E-11 |
| GSTM4 | -0.186040054 | 2.15E-05 | 6.11E-05 |
| GSTM5 | -0.355212183 | 9.24E-17 | 1.47E-15 |
| GSTO1 | 0.213745754 | 9.80E-07 | 3.45E-06 |
| GSTT2 | -0.09494732 | 0.031214236 | 0.049896846 |
| GTF2A1 | 0.147223211 | 0.000804662 | 0.00174046 |
| GTF2A2 | 0.289403325 | 2.15E-11 | 1.53E-10 |
| GTF2B | 0.168388075 | 0.000123286 | 0.00030919 |
| GTF2E1 | 0.295888899 | 7.26E-12 | 5.56E-11 |
| GTF2E2 | 0.297303604 | 5.71E-12 | 4.43E-11 |
| GTF2F2 | 0.280029187 | 9.85E-11 | 6.35E-10 |
| GTF2H1 | 0.157795874 | 0.000324638 | 0.000753117 |
| GTF2H2 | 0.113758947 | 0.009774097 | 0.017393577 |
| GTF2H3 | 0.312664068 | 3.84E-13 | 3.58E-12 |
| GTF2H4 | 0.113296684 | 0.010077659 | 0.017881248 |
| GTF2H5 | 0.146435923 | 0.000858936 | 0.001847655 |
| GTF2IP1 | -0.185618325 | 2.25E-05 | 6.35E-05 |
| GTF2IRD1 | 0.158150828 | 0.000314575 | 0.000731706 |
| GTF2IRD2B | -0.326089899 | 3.18E-14 | 3.49E-13 |
| GTF2IRD2P1 | -0.374993043 | 1.22E-18 | 2.63E-17 |
| GTF2IRD2 | -0.353774711 | 1.25E-16 | 1.96E-15 |
| GTF3A | 0.309137913 | 7.24E-13 | 6.49E-12 |
| GTF3C1 | -0.108607625 | 0.013662804 | 0.023572906 |
| GTF3C2 | 0.212021773 | 1.20E-06 | 4.16E-06 |
| GTF3C3 | 0.27182986 | 3.56E-10 | 2.11E-09 |
| GTF3C4 | 0.147902724 | 0.000760388 | 0.001652384 |
| GTF3C6 | 0.261592326 | 1.67E-09 | 8.93E-09 |
| GTPBP10 | 0.242493469 | 2.50E-08 | 1.13E-07 |
| GTPBP1 | -0.257449388 | 3.06E-09 | 1.57E-08 |
| GTPBP4 | 0.417873978 | 3.50E-23 | 1.48E-21 |
| GTPBP8 | 0.20364539 | 3.18E-06 | 1.03E-05 |
| GTSE1 | 0.43686213 | 2.05E-25 | 1.23E-23 |
| GTSF1L | -0.119365594 | 0.006688741 | 0.012274564 |
| GUCA1A | 0.336286355 | 4.41E-15 | 5.54E-14 |
| GUCA2B | 0.134431636 | 0.002234044 | 0.0044663 |
| GUCY1A2 | -0.183927958 | 2.67E-05 | 7.47E-05 |
| GUCY1A3 | -0.274240349 | 2.45E-10 | 1.49E-09 |
| GUCY1B3 | -0.144880732 | 0.000976224 | 0.002079365 |
| GUCY2D | -0.134023057 | 0.002304943 | 0.004596089 |
| GUCY2E | 0.095352259 | 0.030498073 | 0.048877124 |
| GUCY2F | -0.173030102 | 7.92E-05 | 0.000204332 |
| GUCY2GP | 0.11587277 | 0.008487059 | 0.015256708 |
| GUF1 | 0.146226217 | 0.000873953 | 0.001876928 |
| GULP1 | 0.181045235 | 3.58E-05 | 9.80E-05 |
| GUSBL1 | -0.23374944 | 8.04E-08 | 3.35E-07 |
| GUSBP1 | -0.200533642 | 4.51E-06 | 1.42E-05 |
| GUSBP3 | -0.16195188 | 0.000223607 | 0.000536035 |
| GUSB | -0.161263383 | 0.000238003 | 0.000566938 |
| GVIN1 | -0.216971137 | 6.65E-07 | 2.40E-06 |
| GYG1 | 0.196389078 | 7.13E-06 | 2.18E-05 |
| GYPB | -0.120973798 | 0.005982059 | 0.01107245 |
| GYPC | -0.250129731 | 8.70E-09 | 4.20E-08 |
| GYPE | -0.289832635 | 2.00E-11 | 1.43E-10 |
| GYS1 | -0.097557972 | 0.026839361 | 0.043561928 |
| GYS2 | 0.11102353 | 0.011695494 | 0.02044691 |
| GZMB | 0.222715429 | 3.29E-07 | 1.25E-06 |
| GZMK | -0.124097654 | 0.004798026 | 0.009045741 |
| GZMM | -0.125945818 | 0.004201445 | 0.007989581 |
| H1F0 | 0.176831881 | 5.46E-05 | 0.000144528 |
| H1FNT | -0.366534442 | 8.05E-18 | 1.53E-16 |
| H2AFV | 0.178142788 | 4.79E-05 | 0.000128383 |
| H2AFX | 0.381048322 | 3.05E-19 | 7.19E-18 |
| H2AFY | 0.138847438 | 0.001585258 | 0.003249861 |
| H2AFZ | 0.494655628 | 3.89E-33 | 7.62E-31 |
| H2BFXP | -0.157142019 | 0.000343964 | 0.00079399 |
| H3F3A | 0.100497519 | 0.022555135 | 0.037172827 |
| H6PD | -0.268783492 | 5.67E-10 | 3.27E-09 |
| HAAO | -0.359714662 | 3.54E-17 | 6.10E-16 |
| HABP2 | -0.315335956 | 2.36E-13 | 2.27E-12 |
| HABP4 | -0.175849562 | 6.01E-05 | 0.000158223 |
| HACE1 | -0.127334549 | 0.003798237 | 0.007276829 |
| HADHA | 0.168631966 | 0.000120484 | 0.000302503 |
| HADH | -0.128865243 | 0.003394693 | 0.006562832 |
| HAGH | -0.265151374 | 9.82E-10 | 5.47E-09 |
| HAL | 0.145155008 | 0.00095452 | 0.002036391 |
| HAND1 | -0.148058802 | 0.000750542 | 0.001634365 |
| HAND2 | -0.138240194 | 0.001662816 | 0.003398401 |
| HAO1 | 0.203122012 | 3.37E-06 | 1.08E-05 |
| HAPLN1 | 0.099213944 | 0.02434696 | 0.039827076 |
| HAPLN4 | -0.124922688 | 0.004522853 | 0.008556818 |
| HAR1A | -0.13131211 | 0.002829831 | 0.0055508 |
| HARBI1 | 0.158714699 | 0.000299187 | 0.000698778 |
| HAS2AS | 0.119903219 | 0.006444581 | 0.011854802 |
| HAS3 | -0.317035475 | 1.73E-13 | 1.69E-12 |
| HAT1 | 0.266443536 | 8.08E-10 | 4.56E-09 |
| HAUS1 | 0.249264707 | 9.83E-09 | 4.72E-08 |
| HAUS2 | 0.236360176 | 5.70E-08 | 2.43E-07 |
| HAUS5 | -0.162973758 | 0.000203737 | 0.000492417 |
| HAUS6 | 0.33292522 | 8.54E-15 | 1.02E-13 |
| HAUS7 | 0.15973795 | 0.000273042 | 0.000642898 |
| HAUS8 | 0.127893473 | 0.003646102 | 0.007006181 |
| HAVCR1 | 0.141647468 | 0.001268793 | 0.002650015 |
| HAX1 | 0.210265956 | 1.48E-06 | 5.04E-06 |
| HBA1 | -0.201319792 | 4.13E-06 | 1.31E-05 |
| HBA2 | -0.201081727 | 4.24E-06 | 1.35E-05 |
| HBBP1 | 0.11100715 | 0.01170794 | 0.02046688 |
| HBB | -0.16020227 | 0.000261897 | 0.000618916 |
| HBEGF | -0.143246615 | 0.001115262 | 0.002352703 |
| HBQ1 | 0.192216427 | 1.12E-05 | 3.33E-05 |
| HBS1L | 0.249896955 | 8.99E-09 | 4.34E-08 |
| HBXIP | 0.185348424 | 2.31E-05 | 6.52E-05 |
| HBZ | -0.215161815 | 8.27E-07 | 2.94E-06 |
| HCCS | 0.340081073 | 2.08E-15 | 2.74E-14 |
| HCG26 | -0.324530516 | 4.28E-14 | 4.60E-13 |
| HCG27 | -0.361934328 | 2.20E-17 | 3.87E-16 |
| HCG2P7 | -0.269248274 | 5.28E-10 | 3.06E-09 |
| HCG4P6 | -0.132542328 | 0.002579448 | 0.005097687 |
| HCG4 | -0.097468489 | 0.026980082 | 0.043761898 |
| HCK | -0.214609904 | 8.84E-07 | 3.13E-06 |
| HCLS1 | -0.217134586 | 6.52E-07 | 2.36E-06 |
| HCN1 | -0.167077531 | 0.000139425 | 0.00034649 |
| HCN3 | -0.098236121 | 0.0257933 | 0.04200395 |
| HCN4 | -0.189831227 | 1.44E-05 | 4.23E-05 |
| HCRTR1 | -0.23240039 | 9.59E-08 | 3.96E-07 |
| HCRTR2 | -0.155892997 | 0.0003839 | 0.000877862 |
| HCST | -0.162334065 | 0.000215972 | 0.000519539 |
| HDAC10 | -0.357215041 | 6.04E-17 | 1.00E-15 |
| HDAC11 | -0.316014877 | 2.09E-13 | 2.02E-12 |
| HDAC1 | 0.147911566 | 0.000759827 | 0.001651345 |
| HDAC2 | 0.344147685 | 9.15E-16 | 1.27E-14 |
| HDAC5 | -0.432779029 | 6.37E-25 | 3.53E-23 |
| HDAC6 | -0.276164789 | 1.81E-10 | 1.12E-09 |
| HDAC7 | -0.275865485 | 1.90E-10 | 1.17E-09 |
| HDAC9 | -0.140340143 | 0.001408512 | 0.002917142 |
| HDC | -0.387836845 | 6.23E-20 | 1.60E-18 |
| HDDC2 | 0.144325491 | 0.001021563 | 0.002170848 |
| HDDC3 | 0.141677469 | 0.001265742 | 0.002644471 |
| HDGF | 0.375871501 | 9.99E-19 | 2.17E-17 |
| HDHD2 | -0.182866465 | 2.98E-05 | 8.26E-05 |
| HDLBP | 0.108132775 | 0.014082069 | 0.024243944 |
| HEATR1 | 0.300082165 | 3.54E-12 | 2.83E-11 |
| HEATR2 | 0.33706327 | 3.79E-15 | 4.80E-14 |
| HEATR3 | 0.176533639 | 5.62E-05 | 0.00014851 |
| HEATR5A | 0.208218444 | 1.88E-06 | 6.31E-06 |
| HEATR5B | -0.229847658 | 1.33E-07 | 5.38E-07 |
| HEATR7A | -0.177028899 | 5.35E-05 | 0.000142043 |
| HEBP2 | 0.240108607 | 3.46E-08 | 1.52E-07 |
| HECA | -0.23400996 | 7.77E-08 | 3.25E-07 |
| HECTD3 | -0.193122294 | 1.02E-05 | 3.04E-05 |
| HECW2 | -0.112853426 | 0.010376573 | 0.018354596 |
| HEG1 | -0.126576669 | 0.004013713 | 0.007661009 |
| HELB | 0.117808473 | 0.007443357 | 0.013536332 |
| HELLS | 0.374102624 | 1.49E-18 | 3.16E-17 |
| HELQ | -0.158147628 | 0.000314665 | 0.000731765 |
| HELT | -0.173235246 | 7.76E-05 | 0.000200667 |
| HELZ | -0.240171637 | 3.43E-08 | 1.51E-07 |
| HEMGN | -0.269115254 | 5.39E-10 | 3.12E-09 |
| HEMK1 | -0.321114013 | 8.13E-14 | 8.32E-13 |
| HEPACAM | -0.231355662 | 1.10E-07 | 4.50E-07 |
| HEPN1 | -0.114529951 | 0.009285835 | 0.016583745 |
| HERC1 | -0.373338376 | 1.77E-18 | 3.69E-17 |
| HERC2P2 | -0.306126895 | 1.23E-12 | 1.07E-11 |
| HERC2 | -0.163274075 | 0.000198219 | 0.000480068 |
| HERC3 | -0.251201479 | 7.48E-09 | 3.65E-08 |
| HERC4 | 0.160128117 | 0.000263648 | 0.000622759 |
| HERC5 | -0.13041257 | 0.003026703 | 0.005905676 |
| HERC6 | -0.213060034 | 1.06E-06 | 3.72E-06 |
| HERPUD1 | -0.150252198 | 0.000624099 | 0.001376572 |
| HERPUD2 | 0.125559887 | 0.004320173 | 0.008200534 |
| HES4 | 0.113865235 | 0.009705458 | 0.017282201 |
| HES6 | 0.104603462 | 0.017568945 | 0.029604457 |
| HES7 | 0.137667952 | 0.001739073 | 0.003542016 |
| HESRG | 0.103520654 | 0.018780199 | 0.031473012 |
| HESX1 | -0.21895054 | 5.23E-07 | 1.93E-06 |
| HEXDC | -0.268991255 | 5.50E-10 | 3.18E-09 |
| HEY2 | -0.1707041 | 9.90E-05 | 0.00025208 |
| HEYL | -0.169510061 | 0.000110882 | 0.000280226 |
| HFM1 | -0.130843368 | 0.002930915 | 0.005733888 |
| HGD | 0.160197731 | 0.000262003 | 0.000619022 |
| HGF | -0.154590844 | 0.000430105 | 0.000976723 |
| HHATL | -0.212568289 | 1.13E-06 | 3.92E-06 |
| HHAT | -0.279870975 | 1.01E-10 | 6.51E-10 |
| HHEX | -0.138959533 | 0.00157131 | 0.003223865 |
| HHIPL2 | 0.28435831 | 4.91E-11 | 3.30E-10 |
| HHIP | -0.285611326 | 4.01E-11 | 2.73E-10 |
| HHLA2 | -0.217409419 | 6.31E-07 | 2.29E-06 |
| HHLA3 | -0.116210899 | 0.008295836 | 0.014951955 |
| HIAT1 | 0.110519245 | 0.012084103 | 0.021065503 |
| HIBADH | 0.211858035 | 1.23E-06 | 4.24E-06 |
| HIF1A | 0.295543331 | 7.69E-12 | 5.87E-11 |
| HIF3A | -0.20136007 | 4.11E-06 | 1.31E-05 |
| HIGD1A | 0.207632119 | 2.01E-06 | 6.71E-06 |
| HIGD1B | -0.277203716 | 1.54E-10 | 9.64E-10 |
| HINT1 | 0.173249079 | 7.75E-05 | 0.000200436 |
| HINT3 | -0.139232625 | 0.001537798 | 0.003162946 |
| HIP1R | -0.10919123 | 0.0131626 | 0.022786411 |
| HIP1 | -0.329301743 | 1.72E-14 | 1.96E-13 |
| HIPK1 | -0.228783924 | 1.53E-07 | 6.10E-07 |
| HIRIP3 | -0.175806262 | 6.04E-05 | 0.000158855 |
| HIST1H1B | 0.259510222 | 2.26E-09 | 1.19E-08 |
| HIST1H1D | 0.153707722 | 0.000464332 | 0.001047052 |
| HIST1H1E | 0.206288554 | 2.35E-06 | 7.76E-06 |
| HIST1H2AC | 0.116356086 | 0.008214914 | 0.014820806 |
| HIST1H2AD | 0.09635023 | 0.028792908 | 0.046397344 |
| HIST1H2AE | 0.163503104 | 0.000194106 | 0.000470619 |
| HIST1H2AG | 0.228903966 | 1.51E-07 | 6.02E-07 |
| HIST1H2AH | 0.163215803 | 0.000199279 | 0.000482342 |
| HIST1H2AJ | 0.177505394 | 5.11E-05 | 0.000136132 |
| HIST1H2AL | 0.176722327 | 5.52E-05 | 0.000146022 |
| HIST1H2AM | 0.210001935 | 1.53E-06 | 5.19E-06 |
| HIST1H2BC | 0.190239706 | 1.38E-05 | 4.06E-05 |
| HIST1H2BD | 0.200742468 | 4.40E-06 | 1.39E-05 |
| HIST1H2BE | 0.140077798 | 0.0014382 | 0.002973084 |
| HIST1H2BF | 0.172323517 | 8.47E-05 | 0.000217655 |
| HIST1H2BG | 0.152947612 | 0.000495804 | 0.001112123 |
| HIST1H2BH | 0.198298583 | 5.78E-06 | 1.80E-05 |
| HIST1H2BJ | 0.220165 | 4.51E-07 | 1.68E-06 |
| HIST1H2BK | 0.167829986 | 0.000129931 | 0.000324715 |
| HIST1H2BL | 0.173066525 | 7.89E-05 | 0.000203666 |
| HIST1H2BM | 0.134191094 | 0.002275539 | 0.004541988 |
| HIST1H2BN | 0.187926821 | 1.77E-05 | 5.09E-05 |
| HIST1H2BO | 0.281092076 | 8.31E-11 | 5.42E-10 |
| HIST1H3B | 0.269286583 | 5.25E-10 | 3.04E-09 |
| HIST1H3C | 0.221702993 | 3.73E-07 | 1.40E-06 |
| HIST1H3D | 0.175719827 | 6.09E-05 | 0.00016002 |
| HIST1H3F | 0.137358958 | 0.00178157 | 0.00362185 |
| HIST1H3G | 0.169114195 | 0.000115118 | 0.000289941 |
| HIST1H3H | 0.152741787 | 0.000504661 | 0.001130467 |
| HIST1H3I | 0.137688453 | 0.001736287 | 0.003536985 |
| HIST1H3J | 0.171141351 | 9.49E-05 | 0.000242381 |
| HIST1H4A | 0.112505291 | 0.010616829 | 0.018741427 |
| HIST1H4B | 0.105502374 | 0.016615842 | 0.028143162 |
| HIST1H4C | 0.122358537 | 0.005428121 | 0.010131705 |
| HIST1H4D | 0.136524479 | 0.00190114 | 0.003845375 |
| HIST1H4H | 0.117016892 | 0.007855436 | 0.014227479 |
| HIST1H4L | 0.105643214 | 0.016470688 | 0.027930441 |
| HIST2H2AA3 | 0.167482434 | 0.000134238 | 0.000334266 |
| HIST2H2AB | 0.197695912 | 6.18E-06 | 1.91E-05 |
| HIST2H2AC | 0.178559331 | 4.60E-05 | 0.000123515 |
| HIST2H3C | 0.177263457 | 5.23E-05 | 0.000139099 |
| HIST2H4A | 0.180329292 | 3.85E-05 | 0.000104801 |
| HIST3H2A | 0.146563787 | 0.000849897 | 0.00182959 |
| HIST3H3 | -0.139874345 | 0.001461618 | 0.003017438 |
| HIST4H4 | -0.159393971 | 0.000281583 | 0.000661917 |
| HIVEP1 | -0.144002554 | 0.001048818 | 0.002222152 |
| HIVEP3 | -0.267554519 | 6.84E-10 | 3.90E-09 |
| HJURP | 0.496461794 | 2.11E-33 | 4.39E-31 |
| HK2 | 0.112386355 | 0.010700032 | 0.018876632 |
| HK3 | -0.158746838 | 0.000298331 | 0.000697024 |
| HKR1 | -0.201354819 | 4.11E-06 | 1.31E-05 |
| HLA-A | -0.117785134 | 0.007455225 | 0.013554215 |
| HLA-B | -0.189681401 | 1.47E-05 | 4.29E-05 |
| HLA-C | -0.146548455 | 0.000850976 | 0.001831519 |
| HLA-DMA | -0.397056384 | 6.79E-21 | 2.03E-19 |
| HLA-DMB | -0.297235878 | 5.77E-12 | 4.47E-11 |
| HLA-DOA | -0.381470191 | 2.77E-19 | 6.59E-18 |
| HLA-DOB | -0.302175478 | 2.47E-12 | 2.02E-11 |
| HLA-DPA1 | -0.341191613 | 1.66E-15 | 2.23E-14 |
| HLA-DPB1 | -0.384109672 | 1.50E-19 | 3.70E-18 |
| HLA-DPB2 | -0.25260649 | 6.13E-09 | 3.02E-08 |
| HLA-DQA1 | -0.313268612 | 3.44E-13 | 3.23E-12 |
| HLA-DQA2 | -0.218127789 | 5.78E-07 | 2.11E-06 |
| HLA-DQB1 | -0.341991793 | 1.41E-15 | 1.91E-14 |
| HLA-DQB2 | -0.401749098 | 2.14E-21 | 6.95E-20 |
| HLA-DRA | -0.303970099 | 1.80E-12 | 1.52E-11 |
| HLA-DRB1 | -0.357938856 | 5.18E-17 | 8.67E-16 |
| HLA-DRB5 | -0.324363842 | 4.42E-14 | 4.73E-13 |
| HLA-DRB6 | -0.178473272 | 4.64E-05 | 0.000124495 |
| HLA-E | -0.26619083 | 8.40E-10 | 4.72E-09 |
| HLA-F | -0.238140255 | 4.50E-08 | 1.95E-07 |
| HLA-J | -0.309536258 | 6.74E-13 | 6.05E-12 |
| HLA-L | -0.295181748 | 8.18E-12 | 6.21E-11 |
| HLCS | -0.128356518 | 0.003524273 | 0.006793632 |
| HLF | -0.490880877 | 1.38E-32 | 2.42E-30 |
| HLTF | 0.205018221 | 2.72E-06 | 8.87E-06 |
| HLX | -0.252302204 | 6.40E-09 | 3.14E-08 |
| HM13 | 0.16846128 | 0.000122439 | 0.000307296 |
| HMBS | 0.3019134 | 2.58E-12 | 2.10E-11 |
| HMCN1 | -0.33207697 | 1.01E-14 | 1.19E-13 |
| HMG20A | -0.185709747 | 2.22E-05 | 6.30E-05 |
| HMGA1 | 0.504551229 | 1.30E-34 | 3.41E-32 |
| HMGA2 | 0.107902158 | 0.014289745 | 0.024567644 |
| HMGB1 | 0.161682593 | 0.000229138 | 0.000548307 |
| HMGB2 | 0.277384111 | 1.50E-10 | 9.39E-10 |
| HMGB3L1 | 0.134630662 | 0.002200234 | 0.004405078 |
| HMGB3 | 0.264207392 | 1.13E-09 | 6.23E-09 |
| HMGCLL1 | -0.318750821 | 1.26E-13 | 1.26E-12 |
| HMGCL | -0.158958251 | 0.00029276 | 0.000685209 |
| HMGCR | 0.126574067 | 0.004014471 | 0.007661726 |
| HMGCS2 | -0.100147252 | 0.023032384 | 0.037898586 |
| HMGN2 | 0.09677218 | 0.028096953 | 0.045396644 |
| HMGN3 | -0.359697799 | 3.56E-17 | 6.11E-16 |
| HMGN4 | 0.189114246 | 1.56E-05 | 4.54E-05 |
| HMGN5 | -0.103664122 | 0.018615637 | 0.031220788 |
| HMGXB4 | 0.317666116 | 1.54E-13 | 1.52E-12 |
| HMHA1 | -0.355897987 | 7.99E-17 | 1.30E-15 |
| HMMR | 0.52311291 | 1.62E-37 | 7.54E-35 |
| HMOX2 | -0.119536777 | 0.006610114 | 0.012138076 |
| HMX2 | 0.188569858 | 1.65E-05 | 4.78E-05 |
| HN1L | 0.194490446 | 8.77E-06 | 2.65E-05 |
| HN1 | 0.365105165 | 1.10E-17 | 2.04E-16 |
| HNF1A | 0.121917808 | 0.0055992 | 0.010427356 |
| HNF1B | -0.238837163 | 4.10E-08 | 1.79E-07 |
| HNF4A | 0.129455722 | 0.003249721 | 0.00630574 |
| HNF4G | 0.130198183 | 0.003075425 | 0.00599489 |
| HNMT | -0.220935032 | 4.10E-07 | 1.53E-06 |
| HNRNPA1L2 | 0.166220759 | 0.000151026 | 0.000373233 |
| HNRNPA1 | 0.162681508 | 0.000209244 | 0.00050469 |
| HNRNPA2B1 | 0.121843933 | 0.005628346 | 0.010475778 |
| HNRNPA3P1 | 0.168597438 | 0.000120877 | 0.000303452 |
| HNRNPAB | 0.218237705 | 5.70E-07 | 2.09E-06 |
| HNRNPC | 0.485508359 | 8.17E-32 | 1.27E-29 |
| HNRNPD | 0.124683296 | 0.004601191 | 0.00869844 |
| HNRNPF | 0.38414315 | 1.49E-19 | 3.68E-18 |
| HNRNPH1 | -0.196591001 | 6.97E-06 | 2.14E-05 |
| HNRNPK | 0.31501661 | 2.50E-13 | 2.40E-12 |
| HNRNPL | 0.310074065 | 6.12E-13 | 5.54E-12 |
| HNRNPR | 0.300000917 | 3.59E-12 | 2.86E-11 |
| HNRNPU | 0.100641863 | 0.02236097 | 0.03689237 |
| HNRPDL | -0.114521649 | 0.009290975 | 0.016591442 |
| HNRPLL | 0.233318986 | 8.50E-08 | 3.54E-07 |
| HOMER1 | 0.322823938 | 5.90E-14 | 6.17E-13 |
| HOMER2 | -0.197076366 | 6.61E-06 | 2.03E-05 |
| HOOK2 | -0.267922856 | 6.46E-10 | 3.70E-09 |
| HOPX | -0.249440319 | 9.59E-09 | 4.61E-08 |
| HORMAD1 | 0.11709817 | 0.007812206 | 0.014154312 |
| HOXA10 | 0.14410375 | 0.001040206 | 0.002205074 |
| HOXA11AS | 0.142693406 | 0.001166323 | 0.002451364 |
| HOXA11 | 0.207370642 | 2.07E-06 | 6.90E-06 |
| HOXA13 | 0.096060196 | 0.029279822 | 0.047109803 |
| HOXA1 | 0.15630818 | 0.000370169 | 0.000848794 |
| HOXA5 | -0.190349113 | 1.37E-05 | 4.01E-05 |
| HOXA7 | -0.166112883 | 0.000152549 | 0.000376439 |
| HOXB13 | 0.098560421 | 0.025305611 | 0.041270281 |
| HOXB7 | 0.156155745 | 0.000375156 | 0.000858949 |
| HOXB8 | 0.107498605 | 0.014659642 | 0.025149509 |
| HOXB9 | 0.183531076 | 2.78E-05 | 7.75E-05 |
| HOXC10 | 0.124920263 | 0.00452364 | 0.008557497 |
| HOXC11 | 0.105616909 | 0.016497714 | 0.027964408 |
| HOXC12 | 0.099257047 | 0.024284851 | 0.039741739 |
| HOXC13 | 0.103010889 | 0.019375219 | 0.032375178 |
| HOXC5 | 0.138519303 | 0.001626745 | 0.003329448 |
| HOXC6 | 0.18369309 | 2.74E-05 | 7.64E-05 |
| HOXC8 | 0.188880583 | 1.60E-05 | 4.64E-05 |
| HOXC9 | 0.185471555 | 2.28E-05 | 6.44E-05 |
| HOXD10 | 0.150577636 | 0.000607119 | 0.001341784 |
| HOXD11 | 0.175950993 | 5.95E-05 | 0.000156757 |
| HOXD12 | 0.135187447 | 0.002108119 | 0.004236585 |
| HOXD13 | 0.146857327 | 0.000829478 | 0.001789015 |
| HOXD1 | -0.365496618 | 1.01E-17 | 1.89E-16 |
| HOXD3 | -0.159311161 | 0.000283676 | 0.000666288 |
| HOXD4 | -0.12297252 | 0.005197633 | 0.00973576 |
| HOXD8 | 0.115457733 | 0.008727142 | 0.015648884 |
| HP1BP3 | -0.180136748 | 3.93E-05 | 0.000106677 |
| HPCAL1 | -0.18154635 | 3.41E-05 | 9.35E-05 |
| HPCAL4 | -0.266976481 | 7.46E-10 | 4.23E-09 |
| HPCA | -0.205485239 | 2.57E-06 | 8.45E-06 |
| HPDL | 0.347895657 | 4.25E-16 | 6.21E-15 |
| HPGDS | -0.374841134 | 1.26E-18 | 2.70E-17 |
| HPGD | -0.218306398 | 5.66E-07 | 2.07E-06 |
| HPN | -0.189888939 | 1.44E-05 | 4.20E-05 |
| HPRT1 | 0.353079551 | 1.45E-16 | 2.25E-15 |
| HPR | -0.134905587 | 0.0021543 | 0.004321147 |
| HPS1 | -0.316698689 | 1.84E-13 | 1.80E-12 |
| HPS3 | 0.125295159 | 0.004403359 | 0.008348923 |
| HPS4 | -0.217111924 | 6.54E-07 | 2.37E-06 |
| HPSE2 | -0.332465622 | 9.34E-15 | 1.11E-13 |
| HPYR1 | -0.11164913 | 0.011228787 | 0.019725874 |
| HRASLS2 | -0.198312051 | 5.77E-06 | 1.79E-05 |
| HRASLS5 | -0.21319928 | 1.05E-06 | 3.66E-06 |
| HRCT1 | -0.106354262 | 0.015754693 | 0.026827808 |
| HRC | -0.154464037 | 0.000434871 | 0.000986628 |
| HRG | 0.222659716 | 3.31E-07 | 1.26E-06 |
| HRH4 | -0.258308448 | 2.70E-09 | 1.39E-08 |
| HRNBP3 | -0.095287746 | 0.030611218 | 0.049038793 |
| HRNR | -0.115463761 | 0.008723612 | 0.015643958 |
| HRSP12 | 0.256861722 | 3.33E-09 | 1.70E-08 |
| HR | -0.279479281 | 1.07E-10 | 6.88E-10 |
| HS2ST1 | 0.215386317 | 8.05E-07 | 2.87E-06 |
| HS3ST1 | -0.109143729 | 0.0132027 | 0.022844133 |
| HS3ST2 | -0.189276225 | 1.53E-05 | 4.47E-05 |
| HS3ST4 | -0.102367448 | 0.020149674 | 0.033557047 |
| HS3ST5 | -0.229373982 | 1.42E-07 | 5.70E-07 |
| HS3ST6 | -0.138973611 | 0.001569566 | 0.003220664 |
| HS6ST1 | -0.118010122 | 0.007341522 | 0.013374362 |
| HSBP1 | 0.159925512 | 0.000268487 | 0.000633443 |
| HSCB | 0.152864062 | 0.000499382 | 0.001119143 |
| HSD11B1L | -0.174948119 | 6.57E-05 | 0.000171972 |
| HSD11B2 | -0.152395409 | 0.000519899 | 0.001162127 |
| HSD17B10 | 0.227929859 | 1.70E-07 | 6.76E-07 |
| HSD17B11 | -0.14002787 | 0.001443915 | 0.002983972 |
| HSD17B13 | -0.273789652 | 2.63E-10 | 1.59E-09 |
| HSD17B14 | -0.176338009 | 5.73E-05 | 0.00015112 |
| HSD17B2 | -0.138806725 | 0.001590352 | 0.003259301 |
| HSD17B3 | -0.258492091 | 2.63E-09 | 1.36E-08 |
| HSD17B4 | -0.296571796 | 6.46E-12 | 4.97E-11 |
| HSD17B6 | -0.374335531 | 1.42E-18 | 3.02E-17 |
| HSD17B7P2 | -0.202425275 | 3.65E-06 | 1.17E-05 |
| HSD17B8 | -0.106868759 | 0.015253782 | 0.026050289 |
| HSDL2 | -0.182460809 | 3.10E-05 | 8.58E-05 |
| HSF1 | 0.220974997 | 4.08E-07 | 1.53E-06 |
| HSF2BP | 0.324154761 | 4.59E-14 | 4.89E-13 |
| HSF4 | -0.247569129 | 1.24E-08 | 5.86E-08 |
| HSF5 | -0.202874175 | 3.47E-06 | 1.11E-05 |
| HSFX2 | -0.388062918 | 5.91E-20 | 1.53E-18 |
| HSH2D | -0.150359547 | 0.00061845 | 0.001365016 |
| HSN2 | 0.096371518 | 0.028757446 | 0.046343935 |
| HSP90AA1 | 0.367878825 | 5.99E-18 | 1.16E-16 |
| HSP90AB1 | 0.240950546 | 3.09E-08 | 1.37E-07 |
| HSP90AB2P | 0.192177337 | 1.13E-05 | 3.34E-05 |
| HSP90B1 | 0.308330974 | 8.36E-13 | 7.41E-12 |
| HSP90B3P | 0.18575044 | 2.21E-05 | 6.28E-05 |
| HSPA12A | -0.113709412 | 0.009806233 | 0.017446104 |
| HSPA12B | -0.262070175 | 1.55E-09 | 8.37E-09 |
| HSPA13 | 0.337610065 | 3.40E-15 | 4.34E-14 |
| HSPA14 | 0.334740591 | 5.98E-15 | 7.37E-14 |
| HSPA1A | 0.132867546 | 0.002516722 | 0.004983084 |
| HSPA1B | 0.17767681 | 5.02E-05 | 0.000134052 |
| HSPA1L | -0.136958781 | 0.001838023 | 0.003728187 |
| HSPA4L | 0.20911509 | 1.69E-06 | 5.73E-06 |
| HSPA4 | 0.308212228 | 8.53E-13 | 7.55E-12 |
| HSPA5 | 0.219939969 | 4.63E-07 | 1.72E-06 |
| HSPA7 | -0.227648812 | 1.77E-07 | 6.99E-07 |
| HSPA8 | 0.27402582 | 2.53E-10 | 1.54E-09 |
| HSPA9 | 0.311597487 | 4.66E-13 | 4.28E-12 |
| HSPB2 | -0.160251511 | 0.00026074 | 0.000616328 |
| HSPB3 | -0.229485145 | 1.40E-07 | 5.62E-07 |
| HSPB6 | -0.266743897 | 7.73E-10 | 4.37E-09 |
| HSPB7 | -0.326979329 | 2.69E-14 | 2.98E-13 |
| HSPB8 | -0.274189129 | 2.47E-10 | 1.50E-09 |
| HSPB9 | -0.140230766 | 0.00142082 | 0.002940197 |
| HSPBAP1 | -0.12176229 | 0.005660716 | 0.010529163 |
| HSPC157 | -0.147888332 | 0.000761302 | 0.001654191 |
| HSPC159 | -0.221215485 | 3.96E-07 | 1.48E-06 |
| HSPD1 | 0.519779065 | 5.56E-37 | 2.26E-34 |
| HSPE1 | 0.421004316 | 1.53E-23 | 6.81E-22 |
| HSPG2 | -0.294169341 | 9.70E-12 | 7.29E-11 |
| HSPH1 | 0.173508289 | 7.56E-05 | 0.000195729 |
| HTATIP2 | 0.317310119 | 1.65E-13 | 1.61E-12 |
| HTR1B | 0.098233343 | 0.025797511 | 0.042007385 |
| HTR1D | 0.222108897 | 3.54E-07 | 1.34E-06 |
| HTR2C | 0.16922925 | 0.000113871 | 0.000287019 |
| HTR3B | 0.107554165 | 0.014608221 | 0.025067747 |
| HTR3C | -0.22766307 | 1.76E-07 | 6.98E-07 |
| HTR4 | -0.261946712 | 1.58E-09 | 8.51E-09 |
| HTR7P1 | -0.159634743 | 0.000275579 | 0.000648642 |
| HTRA2 | 0.164277966 | 0.000180774 | 0.000440918 |
| HTRA4 | -0.199397376 | 5.12E-06 | 1.60E-05 |
| HTT | -0.190753946 | 1.31E-05 | 3.86E-05 |
| HUNK | -0.246873177 | 1.37E-08 | 6.42E-08 |
| HUS1B | 0.099563949 | 0.023846565 | 0.039081303 |
| HUS1 | 0.141303526 | 0.001304259 | 0.002717275 |
| HVCN1 | -0.247917033 | 1.19E-08 | 5.61E-08 |
| HYDIN | -0.201448976 | 4.07E-06 | 1.29E-05 |
| HYI | -0.30649828 | 1.16E-12 | 1.00E-11 |
| HYLS1 | 0.386521033 | 8.50E-20 | 2.14E-18 |
| HYMAI | -0.137483501 | 0.001764328 | 0.003587892 |
| HYOU1 | 0.164770201 | 0.000172757 | 0.000422962 |
| IAH1 | 0.181190412 | 3.53E-05 | 9.68E-05 |
| IAPP | 0.120133276 | 0.006342567 | 0.011682199 |
| IARS2 | 0.283644434 | 5.51E-11 | 3.68E-10 |
| IARS | 0.402306581 | 1.86E-21 | 6.13E-20 |
| IBSP | 0.180854234 | 3.65E-05 | 9.98E-05 |
| IBTK | 0.158244861 | 0.000311959 | 0.000726063 |
| ICA1L | -0.271605253 | 3.68E-10 | 2.18E-09 |
| ICAM1 | -0.283675311 | 5.48E-11 | 3.66E-10 |
| ICAM2 | -0.145652284 | 0.000916302 | 0.001960297 |
| ICAM3 | -0.160756052 | 0.00024916 | 0.000591474 |
| ICAM4 | -0.363657845 | 1.51E-17 | 2.74E-16 |
| ICAM5 | -0.4480585 | 8.50E-27 | 6.04E-25 |
| ICMT | 0.118792825 | 0.006957953 | 0.012719551 |
| ICOSLG | -0.223085293 | 3.14E-07 | 1.20E-06 |
| ICOS | -0.115534269 | 0.008682419 | 0.015575676 |
| ICT1 | 0.255027187 | 4.34E-09 | 2.18E-08 |
| ID4 | -0.197012621 | 6.66E-06 | 2.05E-05 |
| IDE | 0.171072803 | 9.55E-05 | 0.000243851 |
| IDH1 | 0.192679371 | 1.07E-05 | 3.18E-05 |
| IDH2 | 0.112679011 | 0.010496331 | 0.018546735 |
| IDH3A | 0.129423604 | 0.00325746 | 0.006318303 |
| IDI1 | 0.157326412 | 0.000338408 | 0.000781796 |
| IDI2 | 0.099748297 | 0.023586601 | 0.038703634 |
| IDO2 | -0.309015753 | 7.40E-13 | 6.61E-12 |
| IDS | -0.132643279 | 0.002559826 | 0.005061912 |
| IDUA | -0.339371462 | 2.39E-15 | 3.14E-14 |
| IER2 | -0.192816806 | 1.05E-05 | 3.14E-05 |
| IER3IP1 | 0.169716005 | 0.000108736 | 0.000275414 |
| IER3 | 0.11235357 | 0.010723069 | 0.018912265 |
| IER5L | 0.136093579 | 0.001965719 | 0.003969569 |
| IER5 | 0.119345635 | 0.006697962 | 0.01228923 |
| IFFO1 | -0.255984324 | 3.78E-09 | 1.91E-08 |
| IFFO2 | -0.24891058 | 1.03E-08 | 4.93E-08 |
| IFI27L2 | -0.13932147 | 0.001527038 | 0.003142755 |
| IFI44L | -0.096244412 | 0.028969747 | 0.046659734 |
| IFI44 | -0.126612706 | 0.004003222 | 0.007643174 |
| IFI6 | -0.096583307 | 0.028406669 | 0.04585258 |
| IFIT1 | -0.147694655 | 0.000773699 | 0.001679121 |
| IFIT2 | -0.147141486 | 0.000810145 | 0.00175156 |
| IFIT5 | -0.155142136 | 0.000409945 | 0.000933467 |
| IFLTD1 | -0.201014196 | 4.27E-06 | 1.36E-05 |
| IFNA1 | 0.101422466 | 0.021335851 | 0.035361609 |
| IFNA2 | 0.102248181 | 0.020296146 | 0.033767219 |
| IFNAR1 | -0.215370298 | 8.07E-07 | 2.87E-06 |
| IFNAR2 | -0.182885936 | 2.97E-05 | 8.24E-05 |
| IFNE | 0.097929313 | 0.026262121 | 0.042697842 |
| IFNGR1 | -0.113360272 | 0.010035411 | 0.017814191 |
| IFNG | 0.133428197 | 0.002411845 | 0.00479395 |
| IFRD1 | 0.229159015 | 1.46E-07 | 5.84E-07 |
| IFRD2 | 0.123020613 | 0.005179957 | 0.00970538 |
| IFT122 | -0.236371141 | 5.69E-08 | 2.43E-07 |
| IFT140 | -0.426355428 | 3.67E-24 | 1.82E-22 |
| IFT172 | -0.431139943 | 1.00E-24 | 5.32E-23 |
| IFT27 | -0.150924843 | 0.000589476 | 0.0013051 |
| IFT46 | -0.116358136 | 0.008213777 | 0.014820502 |
| IFT57 | -0.333747086 | 7.27E-15 | 8.79E-14 |
| IFT74 | -0.105635661 | 0.016478444 | 0.027936483 |
| IFT80 | -0.30760958 | 9.50E-13 | 8.35E-12 |
| IFT81 | 0.104082396 | 0.018143023 | 0.030502376 |
| IFT88 | -0.108572144 | 0.013693746 | 0.023624254 |
| IGF2BP1 | 0.280839003 | 8.65E-11 | 5.63E-10 |
| IGF2BP2 | 0.139630574 | 0.001490139 | 0.003072191 |
| IGF2BP3 | 0.319002646 | 1.20E-13 | 1.21E-12 |
| IGF2R | -0.096710571 | 0.028197659 | 0.04554095 |
| IGF2 | -0.144206072 | 0.001031564 | 0.002188844 |
| IGFALS | -0.306153014 | 1.23E-12 | 1.06E-11 |
| IGFBP1 | 0.260770209 | 1.88E-09 | 9.98E-09 |
| IGFBP2 | -0.098674987 | 0.025135237 | 0.041009959 |
| IGFBP3 | 0.16069713 | 0.000250487 | 0.000594413 |
| IGFBP4 | -0.207359419 | 2.07E-06 | 6.91E-06 |
| IGFBP6 | -0.11391257 | 0.009675029 | 0.017234158 |
| IGFBP7 | -0.161057256 | 0.000242478 | 0.000576821 |
| IGFN1 | -0.307758337 | 9.25E-13 | 8.15E-12 |
| IGLL3 | -0.208544172 | 1.81E-06 | 6.10E-06 |
| IGLON5 | 0.173053573 | 7.90E-05 | 0.000203895 |
| IGSF10 | -0.377942815 | 6.23E-19 | 1.39E-17 |
| IGSF21 | -0.123928664 | 0.00485622 | 0.00914855 |
| IGSF22 | -0.144413037 | 0.001014287 | 0.002156305 |
| IGSF3 | -0.276086419 | 1.84E-10 | 1.14E-09 |
| IGSF6 | -0.18419187 | 2.60E-05 | 7.29E-05 |
| IGSF8 | -0.097073847 | 0.027608295 | 0.044675729 |
| IGSF9B | -0.358423441 | 4.67E-17 | 7.85E-16 |
| IHH | -0.200227317 | 4.67E-06 | 1.47E-05 |
| IKBIP | 0.293303767 | 1.12E-11 | 8.33E-11 |
| IKBKB | -0.314333063 | 2.84E-13 | 2.69E-12 |
| IKBKE | -0.141679095 | 0.001265577 | 0.002644471 |
| IKZF1 | -0.265619702 | 9.15E-10 | 5.12E-09 |
| IKZF2 | -0.27233244 | 3.29E-10 | 1.96E-09 |
| IKZF3 | -0.172208363 | 8.57E-05 | 0.000219941 |
| IKZF4 | -0.219534932 | 4.87E-07 | 1.80E-06 |
| IK | -0.197242973 | 6.49E-06 | 2.00E-05 |
| IL10RA | -0.234449159 | 7.33E-08 | 3.08E-07 |
| IL11RA | -0.484229602 | 1.24E-31 | 1.85E-29 |
| IL11 | 0.203010812 | 3.41E-06 | 1.10E-05 |
| IL12B | -0.333587866 | 7.50E-15 | 9.03E-14 |
| IL12RB1 | -0.132074943 | 0.002672092 | 0.005269314 |
| IL12RB2 | 0.187778825 | 1.79E-05 | 5.17E-05 |
| IL13RA2 | -0.155741541 | 0.000389026 | 0.000888668 |
| IL16 | -0.328180562 | 2.14E-14 | 2.40E-13 |
| IL17C | 0.203316681 | 3.30E-06 | 1.06E-05 |
| IL17F | -0.125866668 | 0.004225552 | 0.008032366 |
| IL17RA | -0.216756334 | 6.83E-07 | 2.46E-06 |
| IL17RC | -0.227491474 | 1.80E-07 | 7.13E-07 |
| IL17REL | -0.161757486 | 0.000227587 | 0.000544922 |
| IL17RE | -0.182437539 | 3.11E-05 | 8.60E-05 |
| IL18BP | -0.100991609 | 0.021896506 | 0.036197781 |
| IL1F5 | 0.112715614 | 0.010471097 | 0.018508691 |
| IL1F6 | -0.100241647 | 0.022902914 | 0.037708686 |
| IL1F7 | -0.278851426 | 1.19E-10 | 7.56E-10 |
| IL1F8 | 0.103687473 | 0.018588972 | 0.031184435 |
| IL1R1 | -0.212956894 | 1.08E-06 | 3.75E-06 |
| IL1R2 | 0.346195755 | 6.03E-16 | 8.61E-15 |
| IL20RB | 0.187525363 | 1.84E-05 | 5.29E-05 |
| IL20 | -0.13388378 | 0.002329577 | 0.004641043 |
| IL21R | -0.109924395 | 0.012557137 | 0.02181212 |
| IL22RA2 | -0.130530317 | 0.003000243 | 0.005857845 |
| IL23R | -0.112191016 | 0.010837939 | 0.019103063 |
| IL25 | -0.113429791 | 0.009989403 | 0.017740398 |
| IL27RA | -0.17115244 | 9.48E-05 | 0.000242154 |
| IL28RA | -0.345678655 | 6.70E-16 | 9.48E-15 |
| IL2RG | -0.154200485 | 0.000444934 | 0.001007401 |
| IL2 | -0.218773616 | 5.34E-07 | 1.96E-06 |
| IL31RA | 0.132843335 | 0.002521343 | 0.00499174 |
| IL33 | -0.308118825 | 8.68E-13 | 7.66E-12 |
| IL34 | -0.395484954 | 9.95E-21 | 2.89E-19 |
| IL3RA | -0.268877816 | 5.59E-10 | 3.23E-09 |
| IL4R | -0.1376988 | 0.001734882 | 0.003534483 |
| IL5RA | -0.224321481 | 2.69E-07 | 1.03E-06 |
| IL6R | -0.289440461 | 2.14E-11 | 1.52E-10 |
| IL6ST | -0.134970129 | 0.002143644 | 0.004302356 |
| IL6 | 0.167986796 | 0.000128031 | 0.000320247 |
| IL7R | -0.176210333 | 5.80E-05 | 0.000152951 |
| IL7 | -0.103299763 | 0.019036047 | 0.031867044 |
| IL8 | 0.326628658 | 2.87E-14 | 3.17E-13 |
| IL9R | -0.155122188 | 0.000410659 | 0.000934941 |
| ILDR1 | -0.328451447 | 2.03E-14 | 2.28E-13 |
| ILDR2 | -0.231905237 | 1.02E-07 | 4.20E-07 |
| ILF2 | 0.397728217 | 5.76E-21 | 1.74E-19 |
| IMMP1L | 0.102467101 | 0.020027995 | 0.033368306 |
| IMMT | 0.325022926 | 3.90E-14 | 4.22E-13 |
| IMP4 | 0.22776785 | 1.74E-07 | 6.89E-07 |
| IMPA1 | 0.310357304 | 5.82E-13 | 5.29E-12 |
| IMPA2 | 0.097146864 | 0.027491124 | 0.044500534 |
| IMPAD1 | 0.268120412 | 6.27E-10 | 3.60E-09 |
| IMPDH2 | 0.227316707 | 1.84E-07 | 7.27E-07 |
| IMPG1 | -0.303624571 | 1.92E-12 | 1.60E-11 |
| IMPG2 | -0.109930942 | 0.012551844 | 0.02180482 |
| INADL | -0.152723253 | 0.000505466 | 0.001132016 |
| INCA1 | -0.310264057 | 5.92E-13 | 5.37E-12 |
| INCENP | 0.314591843 | 2.71E-13 | 2.58E-12 |
| INE1 | -0.274582999 | 2.32E-10 | 1.42E-09 |
| INE2 | -0.230116911 | 1.29E-07 | 5.22E-07 |
| ING4 | -0.135378749 | 0.002077292 | 0.004180099 |
| ING5 | -0.24573577 | 1.61E-08 | 7.44E-08 |
| INGX | 0.107779335 | 0.014401446 | 0.024738407 |
| INHA | 0.20225635 | 3.72E-06 | 1.19E-05 |
| INHBA | 0.189458106 | 1.50E-05 | 4.38E-05 |
| INHBB | -0.121235886 | 0.005873473 | 0.010887414 |
| INHBE | 0.277333785 | 1.51E-10 | 9.46E-10 |
| INMT | -0.417778957 | 3.58E-23 | 1.51E-21 |
| INO80D | -0.210476468 | 1.44E-06 | 4.93E-06 |
| INO80 | -0.211919857 | 1.22E-06 | 4.21E-06 |
| INPP1 | -0.152207954 | 0.000528323 | 0.001179637 |
| INPP4A | -0.280806959 | 8.70E-11 | 5.66E-10 |
| INPP4B | 0.182914855 | 2.96E-05 | 8.22E-05 |
| INPP5A | -0.191366456 | 1.23E-05 | 3.63E-05 |
| INPP5B | -0.451209691 | 3.39E-27 | 2.57E-25 |
| INPP5D | -0.280246879 | 9.51E-11 | 6.15E-10 |
| INPP5E | -0.228207427 | 1.65E-07 | 6.54E-07 |
| INPP5J | -0.379908938 | 3.97E-19 | 9.19E-18 |
| INPP5K | -0.340710676 | 1.83E-15 | 2.44E-14 |
| INPPL1 | -0.137004539 | 0.001831486 | 0.003715767 |
| INSL4 | 0.288489853 | 2.50E-11 | 1.76E-10 |
| INSL5 | -0.126678711 | 0.00398407 | 0.007611699 |
| INSM2 | -0.128502473 | 0.003486645 | 0.006726936 |
| INSR | -0.09591325 | 0.029529202 | 0.047488108 |
| INTS2 | 0.18017946 | 3.91E-05 | 0.000106248 |
| INTS3 | -0.198244541 | 5.81E-06 | 1.81E-05 |
| INTS4 | 0.106230505 | 0.015877314 | 0.027004404 |
| INTS6 | 0.2540385 | 5.00E-09 | 2.49E-08 |
| INTS7 | 0.365511491 | 1.01E-17 | 1.89E-16 |
| INTS8 | 0.417398689 | 3.96E-23 | 1.66E-21 |
| INTS9 | -0.137927067 | 0.001704154 | 0.003477909 |
| INTU | -0.155060526 | 0.000412872 | 0.000939599 |
| IP6K1 | -0.149589771 | 0.000660032 | 0.001450866 |
| IP6K2 | -0.139037234 | 0.001561707 | 0.003206513 |
| IPCEF1 | -0.287127632 | 3.13E-11 | 2.17E-10 |
| IPMK | 0.211338865 | 1.30E-06 | 4.49E-06 |
| IPO11 | 0.186793216 | 1.99E-05 | 5.68E-05 |
| IPO13 | -0.185446546 | 2.29E-05 | 6.46E-05 |
| IPO4 | 0.337363577 | 3.57E-15 | 4.54E-14 |
| IPO5 | 0.340230695 | 2.02E-15 | 2.67E-14 |
| IPO7 | 0.286688503 | 3.36E-11 | 2.32E-10 |
| IPO8 | 0.226524496 | 2.04E-07 | 8.00E-07 |
| IPO9 | 0.133567774 | 0.002386363 | 0.004746604 |
| IPPK | 0.17367636 | 7.44E-05 | 0.000192903 |
| IPW | -0.170844279 | 9.77E-05 | 0.000249013 |
| IQCA1 | -0.257524458 | 3.02E-09 | 1.55E-08 |
| IQCC | -0.15049409 | 0.000611437 | 0.001350431 |
| IQCE | -0.102570101 | 0.019902902 | 0.033176482 |
| IQCF1 | -0.113561094 | 0.009903015 | 0.017607318 |
| IQCF5 | 0.119154762 | 0.00678673 | 0.012438398 |
| IQCG | -0.213354172 | 1.03E-06 | 3.60E-06 |
| IQCJ | 0.111166309 | 0.011587493 | 0.020288052 |
| IQCK | -0.193144418 | 1.01E-05 | 3.03E-05 |
| IQGAP1 | -0.153545764 | 0.000470878 | 0.00105978 |
| IQGAP2 | -0.288969917 | 2.31E-11 | 1.63E-10 |
| IQGAP3 | 0.319523736 | 1.09E-13 | 1.10E-12 |
| IQSEC1 | -0.355613464 | 8.49E-17 | 1.37E-15 |
| IQSEC2 | -0.273544076 | 2.73E-10 | 1.64E-09 |
| IQSEC3 | -0.187643193 | 1.82E-05 | 5.24E-05 |
| IQUB | -0.15851064 | 0.000304673 | 0.000710678 |
| IRAK1BP1 | -0.098604381 | 0.025240119 | 0.04117691 |
| IRAK1 | 0.278694094 | 1.22E-10 | 7.74E-10 |
| IRAK3 | -0.164414503 | 0.000178516 | 0.000435835 |
| IRF2BP2 | -0.165740833 | 0.000157915 | 0.000388766 |
| IRF2 | -0.229927095 | 1.32E-07 | 5.34E-07 |
| IRF3 | -0.22064211 | 4.25E-07 | 1.59E-06 |
| IRF4 | -0.12799363 | 0.003619431 | 0.006957607 |
| IRF5 | -0.192278818 | 1.11E-05 | 3.31E-05 |
| IRF7 | -0.101144026 | 0.021696729 | 0.035897212 |
| IRF8 | -0.249109659 | 1.00E-08 | 4.81E-08 |
| IRF9 | -0.101128691 | 0.021716757 | 0.035924401 |
| IRGM | -0.107483729 | 0.014673436 | 0.025171013 |
| IRGQ | -0.097940056 | 0.026245583 | 0.042674425 |
| IRX1 | -0.343130841 | 1.12E-15 | 1.54E-14 |
| IRX2 | -0.387646465 | 6.52E-20 | 1.67E-18 |
| IRX3 | -0.281781493 | 7.44E-11 | 4.88E-10 |
| IRX5 | -0.309572675 | 6.70E-13 | 6.02E-12 |
| IRX6 | -0.330254289 | 1.43E-14 | 1.65E-13 |
| ISCA1P1 | 0.278180123 | 1.32E-10 | 8.35E-10 |
| ISCA1 | 0.182705816 | 3.03E-05 | 8.39E-05 |
| ISCA2 | 0.163717452 | 0.000190329 | 0.000462134 |
| ISCU | -0.287981105 | 2.72E-11 | 1.90E-10 |
| ISG20L2 | 0.283714918 | 5.45E-11 | 3.64E-10 |
| ISG20 | 0.100993353 | 0.021894211 | 0.03619698 |
| ISL2 | 0.162201907 | 0.000218584 | 0.00052538 |
| ISLR2 | -0.337203309 | 3.68E-15 | 4.68E-14 |
| ISM1 | -0.221781798 | 3.69E-07 | 1.39E-06 |
| ISM2 | 0.17133245 | 9.32E-05 | 0.000238291 |
| ISY1 | 0.157738828 | 0.000326283 | 0.000756582 |
| ISYNA1 | -0.120582774 | 0.006147418 | 0.011351001 |
| ITCH | 0.220134734 | 4.52E-07 | 1.68E-06 |
| ITFG2 | -0.183519564 | 2.79E-05 | 7.76E-05 |
| ITFG3 | -0.155153529 | 0.000409537 | 0.000932646 |
| ITGA10 | -0.308862452 | 7.60E-13 | 6.78E-12 |
| ITGA11 | 0.104628115 | 0.017542181 | 0.029561852 |
| ITGA2B | -0.243866214 | 2.08E-08 | 9.47E-08 |
| ITGA3 | -0.128983166 | 0.00336528 | 0.006512899 |
| ITGA4 | -0.213483036 | 1.01E-06 | 3.55E-06 |
| ITGA5 | 0.225657663 | 2.27E-07 | 8.84E-07 |
| ITGA7 | -0.301897871 | 2.59E-12 | 2.11E-11 |
| ITGA8 | -0.332866539 | 8.63E-15 | 1.03E-13 |
| ITGA9 | -0.369024734 | 4.65E-18 | 9.14E-17 |
| ITGAD | -0.209042668 | 1.71E-06 | 5.77E-06 |
| ITGAE | 0.153877447 | 0.000457564 | 0.001032838 |
| ITGAL | -0.27940875 | 1.09E-10 | 6.94E-10 |
| ITGAM | -0.264627083 | 1.06E-09 | 5.88E-09 |
| ITGAV | 0.116123225 | 0.008345046 | 0.015034575 |
| ITGAX | -0.286533839 | 3.45E-11 | 2.38E-10 |
| ITGB1BP1 | 0.221197609 | 3.97E-07 | 1.49E-06 |
| ITGB1BP2 | -0.155111763 | 0.000411032 | 0.000935623 |
| ITGB1 | 0.377400146 | 7.05E-19 | 1.57E-17 |
| ITGB2 | -0.25931025 | 2.33E-09 | 1.22E-08 |
| ITGB3BP | 0.187478427 | 1.85E-05 | 5.32E-05 |
| ITGB3 | -0.097294509 | 0.0272555 | 0.044140571 |
| ITGB4 | -0.097789807 | 0.026477712 | 0.043016867 |
| ITGB6 | -0.185306749 | 2.32E-05 | 6.54E-05 |
| ITGB7 | -0.107123024 | 0.015011433 | 0.025708896 |
| ITGB8 | -0.145816551 | 0.000903992 | 0.001935617 |
| ITGBL1 | -0.182236406 | 3.18E-05 | 8.75E-05 |
| ITIH3 | -0.225109414 | 2.44E-07 | 9.43E-07 |
| ITIH4 | -0.184336501 | 2.56E-05 | 7.19E-05 |
| ITIH5 | -0.396595308 | 7.60E-21 | 2.26E-19 |
| ITK | -0.196120833 | 7.34E-06 | 2.24E-05 |
| ITLN1 | -0.196892404 | 6.75E-06 | 2.07E-05 |
| ITLN2 | -0.1906335 | 1.33E-05 | 3.90E-05 |
| ITM2A | -0.217363419 | 6.34E-07 | 2.30E-06 |
| ITM2B | -0.182394191 | 3.13E-05 | 8.63E-05 |
| ITPA | 0.116171456 | 0.008317943 | 0.014989094 |
| ITPKA | 0.120661703 | 0.006113713 | 0.011293982 |
| ITPKB | -0.263653066 | 1.23E-09 | 6.71E-09 |
| ITPR1 | -0.148171601 | 0.0007435 | 0.001619913 |
| ITPR2 | -0.255975174 | 3.78E-09 | 1.92E-08 |
| ITPR3 | -0.2658484 | 8.84E-10 | 4.95E-09 |
| ITPRIP | -0.160815039 | 0.000247838 | 0.000588616 |
| ITSN2 | -0.298635258 | 4.54E-12 | 3.56E-11 |
| IVD | -0.33825436 | 2.99E-15 | 3.86E-14 |
| IVL | -0.204835098 | 2.77E-06 | 9.05E-06 |
| IWS1 | 0.121106003 | 0.005927062 | 0.010976821 |
| IYD | -0.15457107 | 0.000430845 | 0.00097816 |
| IZUMO1 | -0.168149255 | 0.00012609 | 0.000315706 |
| JAGN1 | 0.222045853 | 3.57E-07 | 1.35E-06 |
| JAK1 | -0.181851896 | 3.30E-05 | 9.08E-05 |
| JAK2 | -0.120635633 | 0.006124827 | 0.011311378 |
| JAK3 | -0.147605834 | 0.000779447 | 0.001690493 |
| JAM2 | -0.324348708 | 4.43E-14 | 4.74E-13 |
| JAM3 | -0.141074559 | 0.001328373 | 0.002762617 |
| JAZF1 | -0.206039023 | 2.42E-06 | 7.96E-06 |
| JDP2 | -0.205702025 | 2.51E-06 | 8.26E-06 |
| JHDM1D | -0.193829834 | 9.42E-06 | 2.83E-05 |
| JKAMP | 0.222267671 | 3.47E-07 | 1.31E-06 |
| JMJD4 | 0.15879789 | 0.000296977 | 0.000694185 |
| JMJD5 | -0.261964838 | 1.58E-09 | 8.49E-09 |
| JMJD6 | 0.180002265 | 3.98E-05 | 0.000107996 |
| JMJD7-PLA2G4B | -0.499615384 | 7.18E-34 | 1.63E-31 |
| JMJD8 | -0.241705955 | 2.79E-08 | 1.25E-07 |
| JMY | -0.248027234 | 1.17E-08 | 5.52E-08 |
| JPH1 | -0.18685296 | 1.97E-05 | 5.65E-05 |
| JPH2 | -0.161263295 | 0.000238005 | 0.000566938 |
| JPH4 | -0.413190341 | 1.18E-22 | 4.60E-21 |
| JTB | 0.134614794 | 0.002202912 | 0.004409692 |
| JUNB | -0.174313499 | 6.99E-05 | 0.000182216 |
| JUND | -0.252279058 | 6.42E-09 | 3.15E-08 |
| JUN | -0.296194289 | 6.89E-12 | 5.29E-11 |
| KAL1 | -0.346550973 | 5.60E-16 | 8.05E-15 |
| KALRN | -0.186451781 | 2.06E-05 | 5.87E-05 |
| KANK1 | -0.249044176 | 1.01E-08 | 4.85E-08 |
| KANK2 | -0.389170836 | 4.54E-20 | 1.19E-18 |
| KANK3 | -0.400876905 | 2.65E-21 | 8.47E-20 |
| KARS | 0.279433171 | 1.08E-10 | 6.92E-10 |
| KAT2B | -0.379327818 | 4.54E-19 | 1.04E-17 |
| KATNA1 | 0.105268684 | 0.016859161 | 0.02851662 |
| KATNAL2 | -0.248950159 | 1.03E-08 | 4.91E-08 |
| KATNB1 | -0.128407801 | 0.00351101 | 0.006770678 |
| KAZALD1 | 0.185042744 | 2.38E-05 | 6.71E-05 |
| KAZ | -0.35496705 | 9.73E-17 | 1.55E-15 |
| KBTBD10 | -0.217305622 | 6.39E-07 | 2.32E-06 |
| KBTBD11 | -0.099880367 | 0.023401868 | 0.038435213 |
| KBTBD13 | -0.183787819 | 2.71E-05 | 7.57E-05 |
| KBTBD2 | 0.118919883 | 0.006897409 | 0.012625038 |
| KBTBD3 | -0.147673533 | 0.000775063 | 0.001681532 |
| KBTBD4 | -0.14143473 | 0.001290624 | 0.002691673 |
| KBTBD8 | -0.13240445 | 0.002606468 | 0.00514549 |
| KCMF1 | 0.428665297 | 1.96E-24 | 1.00E-22 |
| KCNA10 | -0.199828395 | 4.88E-06 | 1.53E-05 |
| KCNA1 | -0.123827303 | 0.004891429 | 0.009205337 |
| KCNA2 | -0.212152329 | 1.18E-06 | 4.10E-06 |
| KCNA3 | -0.301499473 | 2.77E-12 | 2.25E-11 |
| KCNA4 | -0.31646268 | 1.92E-13 | 1.87E-12 |
| KCNA5 | -0.344543875 | 8.44E-16 | 1.18E-14 |
| KCNA6 | -0.234087248 | 7.69E-08 | 3.22E-07 |
| KCNAB1 | -0.218786507 | 5.33E-07 | 1.96E-06 |
| KCNAB2 | -0.318098947 | 1.42E-13 | 1.41E-12 |
| KCNAB3 | -0.28726871 | 3.06E-11 | 2.12E-10 |
| KCNB1 | -0.128493045 | 0.003489064 | 0.006730304 |
| KCNB2 | -0.110437471 | 0.012148184 | 0.021166134 |
| KCNC1 | -0.101021725 | 0.021856906 | 0.036141283 |
| KCNC2 | 0.165146757 | 0.00016685 | 0.000409302 |
| KCNC3 | -0.359782891 | 3.49E-17 | 6.02E-16 |
| KCNC4 | -0.267388798 | 7.01E-10 | 3.99E-09 |
| KCND1 | -0.187823776 | 1.78E-05 | 5.15E-05 |
| KCND2 | 0.336147786 | 4.54E-15 | 5.67E-14 |
| KCND3 | -0.277139758 | 1.56E-10 | 9.72E-10 |
| KCNE1L | -0.117136279 | 0.007792009 | 0.014121559 |
| KCNE1 | -0.24753531 | 1.25E-08 | 5.89E-08 |
| KCNE2 | -0.109853252 | 0.012614793 | 0.021904654 |
| KCNE3 | 0.131886034 | 0.00271039 | 0.005340423 |
| KCNF1 | 0.293863385 | 1.02E-11 | 7.63E-11 |
| KCNH1 | -0.20295273 | 3.43E-06 | 1.10E-05 |
| KCNH2 | -0.115902544 | 0.008470064 | 0.015230267 |
| KCNH3 | -0.112803098 | 0.010411004 | 0.018408984 |
| KCNH4 | -0.164101048 | 0.00018374 | 0.000447551 |
| KCNH5 | 0.111197874 | 0.011563736 | 0.020250215 |
| KCNIP1 | -0.145488859 | 0.000928703 | 0.001985127 |
| KCNIP2 | -0.307305291 | 1.00E-12 | 8.78E-12 |
| KCNIP3 | -0.195588424 | 7.78E-06 | 2.37E-05 |
| KCNJ11 | -0.216674201 | 6.90E-07 | 2.48E-06 |
| KCNJ13 | -0.174294841 | 7.00E-05 | 0.0001825 |
| KCNJ15 | -0.322255411 | 6.57E-14 | 6.84E-13 |
| KCNJ1 | -0.101276102 | 0.021524894 | 0.035648321 |
| KCNJ2 | -0.15910257 | 0.000289013 | 0.00067755 |
| KCNJ5 | -0.32118142 | 8.03E-14 | 8.23E-13 |
| KCNK12 | 0.106992836 | 0.015135094 | 0.025882479 |
| KCNK13 | -0.123379474 | 0.005049762 | 0.009480992 |
| KCNK15 | -0.099040714 | 0.024597964 | 0.040211344 |
| KCNK17 | -0.279562299 | 1.06E-10 | 6.80E-10 |
| KCNK3 | -0.189520708 | 1.49E-05 | 4.36E-05 |
| KCNK4 | -0.245112521 | 1.75E-08 | 8.07E-08 |
| KCNK5 | -0.342712247 | 1.22E-15 | 1.67E-14 |
| KCNK9 | 0.134443547 | 0.002232007 | 0.004463567 |
| KCNMA1 | -0.118536232 | 0.007081673 | 0.012930345 |
| KCNMB1 | -0.210164075 | 1.50E-06 | 5.10E-06 |
| KCNMB2 | -0.154042494 | 0.00045107 | 0.001019792 |
| KCNMB4 | 0.297088429 | 5.92E-12 | 4.58E-11 |
| KCNN2 | -0.108466524 | 0.013786225 | 0.023777639 |
| KCNQ1OT1 | -0.194491763 | 8.77E-06 | 2.65E-05 |
| KCNQ1 | -0.337376227 | 3.56E-15 | 4.53E-14 |
| KCNQ3 | -0.117358545 | 0.007675143 | 0.013926176 |
| KCNRG | -0.232450835 | 9.52E-08 | 3.94E-07 |
| KCNS1 | -0.168448529 | 0.000122586 | 0.000307627 |
| KCNS2 | -0.195106658 | 8.20E-06 | 2.49E-05 |
| KCNS3 | -0.155036677 | 0.000413732 | 0.00094134 |
| KCNT2 | -0.126309621 | 0.004092236 | 0.007800459 |
| KCNU1 | 0.243105194 | 2.30E-08 | 1.04E-07 |
| KCNV1 | 0.155375921 | 0.000401664 | 0.000915237 |
| KCTD10 | -0.143467802 | 0.00109543 | 0.002314775 |
| KCTD12 | -0.233772576 | 8.01E-08 | 3.35E-07 |
| KCTD14 | -0.146046983 | 0.00088698 | 0.001902714 |
| KCTD16 | -0.099486847 | 0.023956025 | 0.039238961 |
| KCTD18 | -0.178721939 | 4.53E-05 | 0.000121692 |
| KCTD19 | -0.295060298 | 8.35E-12 | 6.33E-11 |
| KCTD1 | -0.101242912 | 0.021567964 | 0.035710774 |
| KCTD20 | 0.166715119 | 0.000144226 | 0.000357666 |
| KCTD2 | -0.276028575 | 1.85E-10 | 1.15E-09 |
| KCTD3 | 0.260691659 | 1.90E-09 | 1.01E-08 |
| KCTD5 | 0.164242791 | 0.000181361 | 0.000442293 |
| KCTD6 | 0.186066361 | 2.14E-05 | 6.09E-05 |
| KCTD7 | -0.337117179 | 3.75E-15 | 4.75E-14 |
| KCTD8 | -0.099086011 | 0.024532115 | 0.040113541 |
| KCTD9 | 0.103005379 | 0.019381739 | 0.032383366 |
| KDELC1 | 0.147771937 | 0.00076873 | 0.00166888 |
| KDELC2 | 0.137960888 | 0.001699644 | 0.003470122 |
| KDELR1 | 0.16100322 | 0.000243665 | 0.000579392 |
| KDELR2 | 0.237443798 | 4.94E-08 | 2.13E-07 |
| KDELR3 | 0.125589647 | 0.004310911 | 0.008185284 |
| KDM1A | 0.282779131 | 6.34E-11 | 4.19E-10 |
| KDM1B | 0.102818775 | 0.019603688 | 0.032732312 |
| KDM3B | -0.158465852 | 0.000305889 | 0.000713266 |
| KDM4A | -0.212770389 | 1.10E-06 | 3.83E-06 |
| KDM4B | -0.154917206 | 0.000418061 | 0.000950325 |
| KDM4C | -0.221679201 | 3.74E-07 | 1.40E-06 |
| KDM4D | -0.119075445 | 0.006823926 | 0.012503797 |
| KDM5A | 0.106857141 | 0.015264936 | 0.026067112 |
| KDM5C | -0.200924703 | 4.32E-06 | 1.37E-05 |
| KDM5D | 0.139803303 | 0.001469878 | 0.003033862 |
| KDM6A | -0.237129893 | 5.15E-08 | 2.21E-07 |
| KDM6B | -0.233952486 | 7.83E-08 | 3.27E-07 |
| KDR | -0.175706904 | 6.10E-05 | 0.000160202 |
| KEL | -0.232006107 | 1.01E-07 | 4.15E-07 |
| KERA | -0.105316048 | 0.016809594 | 0.028442409 |
| KGFLP2 | -0.222481962 | 3.38E-07 | 1.28E-06 |
| KHDC1L | 0.271357273 | 3.83E-10 | 2.26E-09 |
| KHDRBS1 | 0.258029496 | 2.81E-09 | 1.45E-08 |
| KHDRBS2 | -0.2578243 | 2.90E-09 | 1.49E-08 |
| KHK | 0.097906616 | 0.026297094 | 0.042747748 |
| KHSRP | 0.131176857 | 0.002858667 | 0.005600768 |
| KIAA0020 | 0.273184932 | 2.89E-10 | 1.73E-09 |
| KIAA0040 | -0.237781654 | 4.72E-08 | 2.04E-07 |
| KIAA0087 | -0.238324661 | 4.39E-08 | 1.91E-07 |
| KIAA0090 | 0.239233092 | 3.89E-08 | 1.70E-07 |
| KIAA0101 | 0.530378916 | 1.06E-38 | 6.21E-36 |
| KIAA0114 | 0.124175944 | 0.00477128 | 0.00900041 |
| KIAA0125 | -0.127335384 | 0.003798006 | 0.007276829 |
| KIAA0141 | -0.309585786 | 6.68E-13 | 6.01E-12 |
| KIAA0182 | -0.100971377 | 0.021923145 | 0.03623882 |
| KIAA0195 | -0.246497684 | 1.44E-08 | 6.75E-08 |
| KIAA0196 | 0.293472936 | 1.09E-11 | 8.11E-11 |
| KIAA0226 | -0.148706635 | 0.000710924 | 0.001554189 |
| KIAA0232 | -0.165327998 | 0.000164075 | 0.00040294 |
| KIAA0240 | -0.238130263 | 4.51E-08 | 1.95E-07 |
| KIAA0247 | -0.325605225 | 3.49E-14 | 3.80E-13 |
| KIAA0284 | -0.0973958 | 0.027094859 | 0.043919556 |
| KIAA0317 | 0.189152517 | 1.55E-05 | 4.52E-05 |
| KIAA0319L | -0.390184196 | 3.57E-20 | 9.58E-19 |
| KIAA0355 | -0.242125588 | 2.63E-08 | 1.18E-07 |
| KIAA0391 | 0.293006551 | 1.18E-11 | 8.73E-11 |
| KIAA0406 | 0.267512414 | 6.88E-10 | 3.92E-09 |
| KIAA0408 | -0.41092329 | 2.12E-22 | 7.79E-21 |
| KIAA0427 | -0.284448295 | 4.84E-11 | 3.26E-10 |
| KIAA0430 | -0.331760339 | 1.07E-14 | 1.26E-13 |
| KIAA0467 | -0.41515381 | 7.11E-23 | 2.85E-21 |
| KIAA0494 | -0.394556296 | 1.25E-20 | 3.55E-19 |
| KIAA0495 | -0.408827558 | 3.61E-22 | 1.30E-20 |
| KIAA0513 | -0.355442298 | 8.80E-17 | 1.41E-15 |
| KIAA0556 | -0.235301917 | 6.56E-08 | 2.77E-07 |
| KIAA0562 | -0.223656411 | 2.92E-07 | 1.12E-06 |
| KIAA0586 | 0.219137112 | 5.11E-07 | 1.89E-06 |
| KIAA0649 | -0.185891276 | 2.18E-05 | 6.19E-05 |
| KIAA0664P3 | -0.352069809 | 1.79E-16 | 2.74E-15 |
| KIAA0748 | -0.327690956 | 2.35E-14 | 2.62E-13 |
| KIAA0753 | -0.134456302 | 0.002229828 | 0.004459656 |
| KIAA0754 | -0.095359397 | 0.030485577 | 0.048861014 |
| KIAA0892 | -0.370822654 | 3.11E-18 | 6.26E-17 |
| KIAA0895L | -0.283819195 | 5.36E-11 | 3.58E-10 |
| KIAA0907 | -0.164460619 | 0.00017776 | 0.000434147 |
| KIAA0913 | -0.323573332 | 5.13E-14 | 5.42E-13 |
| KIAA0922 | -0.13658243 | 0.001892605 | 0.003830049 |
| KIAA1009 | -0.27302895 | 2.96E-10 | 1.77E-09 |
| KIAA1033 | 0.184939314 | 2.41E-05 | 6.78E-05 |
| KIAA1045 | -0.146596305 | 0.000847612 | 0.001825458 |
| KIAA1109 | -0.33957964 | 2.30E-15 | 3.02E-14 |
| KIAA1147 | -0.146336433 | 0.000866031 | 0.001861514 |
| KIAA1161 | 0.148935487 | 0.000697398 | 0.001526958 |
| KIAA1191 | -0.117353917 | 0.00767756 | 0.013929298 |
| KIAA1199 | 0.109013272 | 0.013313387 | 0.02301574 |
| KIAA1217 | -0.165614973 | 0.000159769 | 0.00039309 |
| KIAA1239 | 0.103279089 | 0.019060148 | 0.031902046 |
| KIAA1244 | -0.25282268 | 5.94E-09 | 2.94E-08 |
| KIAA1267 | -0.274401715 | 2.39E-10 | 1.45E-09 |
| KIAA1274 | -0.335288969 | 5.37E-15 | 6.64E-14 |
| KIAA1279 | 0.179316904 | 4.26E-05 | 0.00011507 |
| KIAA1310 | -0.214288093 | 9.19E-07 | 3.25E-06 |
| KIAA1324L | -0.261959207 | 1.58E-09 | 8.50E-09 |
| KIAA1324 | -0.169511864 | 0.000110863 | 0.000280213 |
| KIAA1328 | -0.282086347 | 7.08E-11 | 4.66E-10 |
| KIAA1370 | -0.276526887 | 1.71E-10 | 1.06E-09 |
| KIAA1377 | -0.169448505 | 0.000111531 | 0.000281723 |
| KIAA1407 | -0.396245069 | 8.27E-21 | 2.44E-19 |
| KIAA1409 | -0.111102593 | 0.01163558 | 0.020359973 |
| KIAA1429 | 0.338329702 | 2.94E-15 | 3.81E-14 |
| KIAA1467 | -0.106966352 | 0.015160359 | 0.025915099 |
| KIAA1522 | -0.152120346 | 0.000532303 | 0.001187728 |
| KIAA1524 | 0.457081939 | 5.98E-28 | 4.88E-26 |
| KIAA1529 | -0.437996382 | 1.50E-25 | 9.14E-24 |
| KIAA1530 | -0.181499699 | 3.42E-05 | 9.39E-05 |
| KIAA1543 | -0.301260007 | 2.89E-12 | 2.34E-11 |
| KIAA1549 | 0.113802867 | 0.009745683 | 0.017347145 |
| KIAA1586 | 0.115299734 | 0.008820117 | 0.015804257 |
| KIAA1598 | 0.154527084 | 0.000432495 | 0.000981461 |
| KIAA1609 | 0.24809322 | 1.16E-08 | 5.48E-08 |
| KIAA1614 | -0.229940671 | 1.32E-07 | 5.33E-07 |
| KIAA1632 | -0.187342955 | 1.88E-05 | 5.39E-05 |
| KIAA1671 | -0.307278047 | 1.01E-12 | 8.82E-12 |
| KIAA1683 | -0.407969437 | 4.49E-22 | 1.59E-20 |
| KIAA1715 | 0.23755627 | 4.86E-08 | 2.10E-07 |
| KIAA1751 | -0.157420292 | 0.000335612 | 0.000775964 |
| KIAA1755 | -0.133124292 | 0.002468189 | 0.004892805 |
| KIAA1826 | -0.13720894 | 0.001802544 | 0.00366151 |
| KIAA1875 | -0.170801684 | 9.81E-05 | 0.000249965 |
| KIAA1908 | -0.326905886 | 2.73E-14 | 3.01E-13 |
| KIAA1984 | -0.156529885 | 0.000363026 | 0.000833849 |
| KIAA2018 | -0.235839527 | 6.11E-08 | 2.59E-07 |
| KIAA2022 | -0.422691687 | 9.79E-24 | 4.46E-22 |
| KIAA2026 | -0.20715143 | 2.13E-06 | 7.06E-06 |
| KIDINS220 | -0.280779743 | 8.73E-11 | 5.68E-10 |
| KIF11 | 0.508612653 | 3.11E-35 | 9.58E-33 |
| KIF12 | -0.182000119 | 3.25E-05 | 8.95E-05 |
| KIF13A | -0.283284524 | 5.84E-11 | 3.88E-10 |
| KIF13B | -0.144104229 | 0.001040165 | 0.002205074 |
| KIF14 | 0.499789493 | 6.76E-34 | 1.55E-31 |
| KIF15 | 0.451525626 | 3.09E-27 | 2.36E-25 |
| KIF16B | -0.282249175 | 6.90E-11 | 4.55E-10 |
| KIF17 | -0.230189876 | 1.28E-07 | 5.17E-07 |
| KIF18A | 0.472179744 | 5.88E-30 | 6.42E-28 |
| KIF18B | 0.399579009 | 3.66E-21 | 1.15E-19 |
| KIF19 | -0.137929372 | 0.001703847 | 0.003477636 |
| KIF1C | -0.228353574 | 1.61E-07 | 6.42E-07 |
| KIF20A | 0.496506246 | 2.08E-33 | 4.37E-31 |
| KIF20B | 0.428694583 | 1.95E-24 | 9.96E-23 |
| KIF21A | 0.206715402 | 2.24E-06 | 7.40E-06 |
| KIF21B | -0.157633745 | 0.000329333 | 0.000762594 |
| KIF22 | 0.112933806 | 0.010321791 | 0.018272249 |
| KIF23 | 0.523721014 | 1.29E-37 | 6.16E-35 |
| KIF24 | 0.232625682 | 9.31E-08 | 3.85E-07 |
| KIF25 | -0.106978106 | 0.015149141 | 0.025900353 |
| KIF26A | -0.229911331 | 1.32E-07 | 5.35E-07 |
| KIF27 | -0.213835144 | 9.70E-07 | 3.42E-06 |
| KIF2A | 0.153031804 | 0.000492223 | 0.001104586 |
| KIF2C | 0.491127567 | 1.27E-32 | 2.25E-30 |
| KIF3A | -0.150788664 | 0.000596338 | 0.001319563 |
| KIF3C | 0.200520704 | 4.52E-06 | 1.43E-05 |
| KIF4A | 0.504745721 | 1.21E-34 | 3.23E-32 |
| KIF4B | 0.423538038 | 7.81E-24 | 3.63E-22 |
| KIF5B | 0.212177662 | 1.18E-06 | 4.09E-06 |
| KIF6 | -0.276999207 | 1.59E-10 | 9.93E-10 |
| KIF7 | -0.229949649 | 1.32E-07 | 5.33E-07 |
| KIF9 | -0.099449098 | 0.024009776 | 0.03932378 |
| KIFAP3 | -0.099232073 | 0.024320821 | 0.039794087 |
| KIFC1 | 0.453454953 | 1.75E-27 | 1.36E-25 |
| KIFC3 | -0.127366762 | 0.003789315 | 0.007263217 |
| KILLIN | -0.18690217 | 1.96E-05 | 5.62E-05 |
| KIN | 0.213710613 | 9.84E-07 | 3.46E-06 |
| KIR2DL3 | 0.097362094 | 0.027148224 | 0.043988221 |
| KIR2DL4 | 0.206718561 | 2.23E-06 | 7.40E-06 |
| KIR3DL3 | 0.113828972 | 0.009728828 | 0.017320728 |
| KIR3DP1 | 0.116865701 | 0.007936421 | 0.014365044 |
| KIR3DX1 | 0.137265964 | 0.001794545 | 0.003646002 |
| KIRREL2 | -0.110304692 | 0.012252872 | 0.021337376 |
| KIRREL3 | -0.114404303 | 0.009363895 | 0.016709717 |
| KIRREL | -0.161114881 | 0.000241219 | 0.000574124 |
| KISS1R | 0.115178934 | 0.008891796 | 0.015925557 |
| KLC2 | 0.125200758 | 0.004433371 | 0.008401045 |
| KLC4 | -0.275068268 | 2.15E-10 | 1.32E-09 |
| KLF11 | -0.252398088 | 6.31E-09 | 3.10E-08 |
| KLF12 | -0.191323663 | 1.23E-05 | 3.64E-05 |
| KLF13 | -0.267150381 | 7.27E-10 | 4.13E-09 |
| KLF15 | -0.341532658 | 1.55E-15 | 2.09E-14 |
| KLF16 | 0.185253774 | 2.33E-05 | 6.58E-05 |
| KLF1 | 0.158439007 | 0.000306621 | 0.000714721 |
| KLF2 | -0.264679581 | 1.05E-09 | 5.84E-09 |
| KLF8 | -0.328313541 | 2.08E-14 | 2.34E-13 |
| KLF9 | -0.179624602 | 4.13E-05 | 0.000111849 |
| KLHDC1 | -0.333622421 | 7.45E-15 | 8.98E-14 |
| KLHDC5 | 0.153893109 | 0.000456944 | 0.001031672 |
| KLHDC7A | -0.395272678 | 1.05E-20 | 3.03E-19 |
| KLHDC8A | -0.132512372 | 0.002585297 | 0.00510773 |
| KLHDC8B | -0.289429796 | 2.14E-11 | 1.52E-10 |
| KLHDC9 | -0.172140262 | 8.62E-05 | 0.00022133 |
| KLHL13 | -0.116536296 | 0.008115453 | 0.014662539 |
| KLHL14 | -0.184063458 | 2.64E-05 | 7.37E-05 |
| KLHL17 | -0.156104799 | 0.000376837 | 0.000862303 |
| KLHL1 | 0.188730649 | 1.62E-05 | 4.71E-05 |
| KLHL20 | -0.206771417 | 2.22E-06 | 7.36E-06 |
| KLHL21 | -0.144244334 | 0.00102835 | 0.002183182 |
| KLHL22 | -0.201343973 | 4.12E-06 | 1.31E-05 |
| KLHL23 | 0.123186183 | 0.005119517 | 0.009603839 |
| KLHL24 | -0.178718315 | 4.53E-05 | 0.000121719 |
| KLHL25 | 0.133064099 | 0.00247949 | 0.004913259 |
| KLHL26 | -0.276700768 | 1.67E-10 | 1.04E-09 |
| KLHL28 | 0.170658743 | 9.94E-05 | 0.000253077 |
| KLHL29 | -0.306001407 | 1.26E-12 | 1.09E-11 |
| KLHL31 | 0.114244099 | 0.009464269 | 0.016876778 |
| KLHL32 | -0.196625415 | 6.95E-06 | 2.13E-05 |
| KLHL33 | -0.374398365 | 1.40E-18 | 2.98E-17 |
| KLHL34 | -0.146046617 | 0.000887007 | 0.001902714 |
| KLHL38 | -0.095850824 | 0.029635695 | 0.047651698 |
| KLHL3 | -0.222542627 | 3.36E-07 | 1.27E-06 |
| KLHL5 | 0.251045258 | 7.65E-09 | 3.73E-08 |
| KLHL6 | -0.243917894 | 2.06E-08 | 9.41E-08 |
| KLHL7 | 0.169842556 | 0.000107438 | 0.000272451 |
| KLK12 | 0.121862546 | 0.00562099 | 0.01046501 |
| KLK14 | 0.124185596 | 0.004767992 | 0.008995057 |
| KLK2 | 0.111089275 | 0.011645653 | 0.020374032 |
| KLK6 | 0.24105329 | 3.04E-08 | 1.35E-07 |
| KLK8 | 0.233124132 | 8.72E-08 | 3.63E-07 |
| KLK9 | 0.170641292 | 9.96E-05 | 0.000253466 |
| KLKB1 | -0.253839001 | 5.14E-09 | 2.56E-08 |
| KLKP1 | 0.11804366 | 0.007324707 | 0.013347273 |
| KLRA1 | -0.196891742 | 6.75E-06 | 2.07E-05 |
| KLRAQ1 | -0.162792774 | 0.000207131 | 0.000500016 |
| KLRB1 | -0.198292876 | 5.78E-06 | 1.80E-05 |
| KLRC1 | 0.129995306 | 0.003122188 | 0.006079521 |
| KLRC2 | 0.180068169 | 3.95E-05 | 0.000107327 |
| KLRC3 | 0.169527589 | 0.000110698 | 0.000279937 |
| KLRD1 | 0.114583497 | 0.009252746 | 0.016532035 |
| KLRG1 | -0.171727844 | 8.97E-05 | 0.000229807 |
| KL | -0.25749591 | 3.04E-09 | 1.56E-08 |
| KNDC1 | -0.250896744 | 7.81E-09 | 3.80E-08 |
| KNG1 | 0.130585846 | 0.002987838 | 0.00583725 |
| KNTC1 | 0.253580919 | 5.34E-09 | 2.65E-08 |
| KPNA1 | 0.23205642 | 1.00E-07 | 4.12E-07 |
| KPNA2 | 0.538140764 | 5.31E-40 | 4.42E-37 |
| KPNA3 | 0.328916265 | 1.85E-14 | 2.11E-13 |
| KPNA4 | 0.393205623 | 1.73E-20 | 4.82E-19 |
| KPNA5 | -0.127486184 | 0.003756405 | 0.00720359 |
| KPNA7 | 0.107520887 | 0.014639001 | 0.025116253 |
| KPNB1 | 0.328427256 | 2.04E-14 | 2.29E-13 |
| KRAS | 0.209941757 | 1.54E-06 | 5.23E-06 |
| KRBA1 | -0.261365448 | 1.72E-09 | 9.20E-09 |
| KRBA2 | -0.173556722 | 7.52E-05 | 0.000194902 |
| KREMEN2 | 0.158078454 | 0.000316603 | 0.000735844 |
| KRR1 | 0.39360463 | 1.57E-20 | 4.39E-19 |
| KRT13 | -0.131487829 | 0.002792762 | 0.005484009 |
| KRT15 | -0.183635843 | 2.75E-05 | 7.68E-05 |
| KRT16 | 0.11426043 | 0.009453994 | 0.016861464 |
| KRT18 | 0.321825932 | 7.12E-14 | 7.38E-13 |
| KRT1 | -0.24574982 | 1.60E-08 | 7.43E-08 |
| KRT222 | -0.108663255 | 0.013614412 | 0.023493471 |
| KRT25 | 0.099738645 | 0.023600151 | 0.038719513 |
| KRT27 | -0.217748134 | 6.05E-07 | 2.20E-06 |
| KRT2 | -0.105085394 | 0.017052179 | 0.028821154 |
| KRT32 | -0.184860567 | 2.43E-05 | 6.83E-05 |
| KRT39 | -0.161697571 | 0.000228827 | 0.000547759 |
| KRT4 | -0.174820077 | 6.65E-05 | 0.000173932 |
| KRT5 | -0.116460961 | 0.0081569 | 0.014729436 |
| KRT6A | 0.231248073 | 1.11E-07 | 4.55E-07 |
| KRT6B | 0.197763461 | 6.13E-06 | 1.90E-05 |
| KRT6C | 0.195617368 | 7.76E-06 | 2.36E-05 |
| KRT72 | -0.221592814 | 3.78E-07 | 1.42E-06 |
| KRT73 | -0.205932578 | 2.45E-06 | 8.05E-06 |
| KRT78 | 0.143465895 | 0.0010956 | 0.002314888 |
| KRT79 | -0.191640281 | 1.19E-05 | 3.53E-05 |
| KRT7 | 0.137923337 | 0.001704653 | 0.00347857 |
| KRT80 | 0.184019392 | 2.65E-05 | 7.41E-05 |
| KRT81 | 0.115824441 | 0.008514709 | 0.015299533 |
| KRT83 | 0.210374675 | 1.46E-06 | 4.98E-06 |
| KRT8 | 0.240278544 | 3.38E-08 | 1.49E-07 |
| KRTAP10-3 | -0.140874921 | 0.001349732 | 0.002802954 |
| KRTAP4-1 | 0.159660977 | 0.000274932 | 0.000647196 |
| KRTAP5-10 | -0.257195665 | 3.17E-09 | 1.62E-08 |
| KRTAP5-11 | -0.110911176 | 0.011781105 | 0.020574987 |
| KRTAP5-1 | -0.10257558 | 0.019896266 | 0.033168188 |
| KRTAP5-2 | -0.136556364 | 0.00189644 | 0.003837032 |
| KRTAP5-7 | -0.148670697 | 0.00071307 | 0.00155837 |
| KRTAP5-8 | -0.279158229 | 1.13E-10 | 7.21E-10 |
| KRTAP5-9 | -0.350489671 | 2.49E-16 | 3.73E-15 |
| KRTCAP2 | 0.12307605 | 0.005159649 | 0.009673676 |
| KSR2 | -0.2164967 | 7.05E-07 | 2.53E-06 |
| KTI12 | 0.113689634 | 0.00981909 | 0.017467423 |
| KTN1 | 0.2456782 | 1.62E-08 | 7.50E-08 |
| KYNU | 0.265175404 | 9.78E-10 | 5.46E-09 |
| KY | -0.122945992 | 0.005207407 | 0.009752239 |
| L1TD1 | -0.20844605 | 1.83E-06 | 6.16E-06 |
| L2HGDH | 0.468814587 | 1.68E-29 | 1.70E-27 |
| L3MBTL2 | -0.277611287 | 1.44E-10 | 9.08E-10 |
| L3MBTL4 | -0.242078614 | 2.65E-08 | 1.19E-07 |
| L3MBTL | -0.304160935 | 1.74E-12 | 1.47E-11 |
| LACTB2 | 0.132430319 | 0.002601379 | 0.005136966 |
| LAGE3 | 0.172176243 | 8.60E-05 | 0.000220593 |
| LAIR1 | -0.162431023 | 0.000214074 | 0.000515595 |
| LAMA2 | -0.324849944 | 4.03E-14 | 4.34E-13 |
| LAMA5 | -0.10181999 | 0.02082967 | 0.034597262 |
| LAMB2L | -0.316364683 | 1.96E-13 | 1.90E-12 |
| LAMB2 | -0.349977093 | 2.77E-16 | 4.13E-15 |
| LAMB3 | 0.118974354 | 0.006871599 | 0.012582404 |
| LAMC1 | 0.179278363 | 4.28E-05 | 0.000115499 |
| LAMC2 | 0.198973831 | 5.36E-06 | 1.67E-05 |
| LAMC3 | -0.172321757 | 8.48E-05 | 0.000217664 |
| LAMP1 | -0.134593148 | 0.002206571 | 0.00441535 |
| LAMP3 | -0.330877023 | 1.27E-14 | 1.47E-13 |
| LANCL3 | -0.271985258 | 3.47E-10 | 2.06E-09 |
| LAPTM4A | -0.097529316 | 0.026884356 | 0.043627873 |
| LAPTM4B | 0.211565952 | 1.27E-06 | 4.38E-06 |
| LAPTM5 | -0.140349792 | 0.001407431 | 0.002915507 |
| LARGE | -0.268448897 | 5.97E-10 | 3.43E-09 |
| LARP1B | 0.262709613 | 1.41E-09 | 7.65E-09 |
| LARP1 | 0.110901062 | 0.011788839 | 0.020586695 |
| LARP4 | 0.393690272 | 1.54E-20 | 4.30E-19 |
| LARS2 | 0.159345846 | 0.000282797 | 0.000664537 |
| LASS4 | -0.284911562 | 4.49E-11 | 3.04E-10 |
| LAT2 | -0.110521192 | 0.012082582 | 0.021064687 |
| LAT | -0.203423048 | 3.26E-06 | 1.05E-05 |
| LBH | -0.256075876 | 3.73E-09 | 1.89E-08 |
| LBP | 0.197859604 | 6.07E-06 | 1.88E-05 |
| LBR | 0.306445747 | 1.17E-12 | 1.01E-11 |
| LBXCOR1 | -0.315189343 | 2.43E-13 | 2.33E-12 |
| LCA5L | -0.261924493 | 1.59E-09 | 8.53E-09 |
| LCA5 | -0.375598507 | 1.06E-18 | 2.30E-17 |
| LCAT | -0.426219379 | 3.81E-24 | 1.88E-22 |
| LCE3D | 0.120790834 | 0.006058928 | 0.011197951 |
| LCE3E | 0.095846769 | 0.029642623 | 0.047659005 |
| LCK | -0.119670674 | 0.006549192 | 0.012033944 |
| LCLAT1 | 0.30372502 | 1.88E-12 | 1.58E-11 |
| LCMT2 | -0.098338681 | 0.025638199 | 0.041764981 |
| LCN10 | -0.215540167 | 7.91E-07 | 2.82E-06 |
| LCN12 | -0.181002611 | 3.60E-05 | 9.84E-05 |
| LCN15 | 0.146961851 | 0.000822318 | 0.001775957 |
| LCN1 | 0.194552107 | 8.71E-06 | 2.63E-05 |
| LCN6 | -0.208913368 | 1.73E-06 | 5.85E-06 |
| LCNL1 | -0.304950217 | 1.52E-12 | 1.29E-11 |
| LCP1 | -0.176525289 | 5.63E-05 | 0.000148593 |
| LCP2 | -0.146591937 | 0.000847919 | 0.001825922 |
| LCTL | 0.169395455 | 0.000112093 | 0.000283036 |
| LCT | -0.173672618 | 7.44E-05 | 0.000192948 |
| LDB1 | -0.385348612 | 1.12E-19 | 2.81E-18 |
| LDB2 | -0.257461595 | 3.05E-09 | 1.56E-08 |
| LDB3 | -0.334388117 | 6.41E-15 | 7.85E-14 |
| LDHA | 0.441452813 | 5.64E-26 | 3.64E-24 |
| LDHB | 0.254322102 | 4.80E-09 | 2.40E-08 |
| LDHD | -0.208929642 | 1.73E-06 | 5.84E-06 |
| LDLRAD2 | -0.283845984 | 5.34E-11 | 3.57E-10 |
| LDLRAP1 | -0.408159242 | 4.28E-22 | 1.53E-20 |
| LDLR | -0.102798383 | 0.019628076 | 0.032767559 |
| LDOC1 | -0.173598291 | 7.49E-05 | 0.000194194 |
| LEAP2 | -0.120506837 | 0.006180003 | 0.011405899 |
| LECT1 | -0.112005808 | 0.010970147 | 0.019309985 |
| LEFTY2 | -0.398580174 | 4.67E-21 | 1.43E-19 |
| LEKR1 | -0.180471408 | 3.80E-05 | 0.000103439 |
| LEMD1 | -0.161205626 | 0.000239249 | 0.000569705 |
| LEMD3 | 0.144135668 | 0.001037503 | 0.002199811 |
| LENG1 | -0.179354906 | 4.25E-05 | 0.000114695 |
| LENG8 | -0.367564658 | 6.42E-18 | 1.24E-16 |
| LEO1 | 0.236010204 | 5.97E-08 | 2.54E-07 |
| LEPREL1 | -0.235848486 | 6.10E-08 | 2.59E-07 |
| LEP | 0.156699617 | 0.000357644 | 0.000822907 |
| LETM1 | 0.212474086 | 1.14E-06 | 3.96E-06 |
| LETM2 | 0.101611755 | 0.021093506 | 0.034994772 |
| LGALS12 | -0.121257722 | 0.005864507 | 0.010871801 |
| LGALS1 | 0.146334478 | 0.00086617 | 0.001861614 |
| LGALS2 | -0.241497497 | 2.87E-08 | 1.28E-07 |
| LGALS3BP | -0.165687236 | 0.000158702 | 0.00039056 |
| LGALS4 | -0.162951606 | 0.000204149 | 0.000493235 |
| LGALS7B | 0.124344757 | 0.004714066 | 0.008900043 |
| LGALS9B | -0.149383625 | 0.0006716 | 0.001474349 |
| LGALS9C | -0.287015012 | 3.19E-11 | 2.21E-10 |
| LGALS9 | -0.319514912 | 1.09E-13 | 1.11E-12 |
| LGI3 | -0.309066642 | 7.33E-13 | 6.56E-12 |
| LGI4 | -0.279200163 | 1.12E-10 | 7.17E-10 |
| LGMN | -0.126893595 | 0.003922295 | 0.007500845 |
| LGR4 | 0.162801646 | 0.000206963 | 0.000499749 |
| LGR5 | -0.106580021 | 0.015533146 | 0.026477616 |
| LGR6 | -0.298607077 | 4.57E-12 | 3.57E-11 |
| LGSN | 0.129505731 | 0.003237705 | 0.006284255 |
| LHCGR | -0.180445629 | 3.81E-05 | 0.00010368 |
| LHFPL1 | -0.15689859 | 0.00035143 | 0.00080954 |
| LHFPL3 | -0.323254036 | 5.44E-14 | 5.72E-13 |
| LHFPL4 | -0.150348852 | 0.000619011 | 0.001366102 |
| LHFPL5 | 0.174108738 | 7.13E-05 | 0.000185545 |
| LHFP | -0.311224393 | 4.98E-13 | 4.55E-12 |
| LHX1 | 0.205772829 | 2.49E-06 | 8.20E-06 |
| LHX2 | 0.102131611 | 0.020440201 | 0.033993339 |
| LHX4 | -0.178781743 | 4.50E-05 | 0.000121017 |
| LHX5 | 0.116358906 | 0.00821335 | 0.014820502 |
| LHX6 | -0.17311866 | 7.85E-05 | 0.000202772 |
| LHX9 | -0.230509932 | 1.22E-07 | 4.98E-07 |
| LIFR | -0.232837343 | 9.06E-08 | 3.76E-07 |
| LIG3 | 0.214911804 | 8.53E-07 | 3.03E-06 |
| LIG4 | -0.12413474 | 0.00478534 | 0.009024377 |
| LILRA1 | -0.156133674 | 0.000375883 | 0.000860417 |
| LILRA2 | -0.147099827 | 0.000812953 | 0.001757251 |
| LILRA3 | 0.123627709 | 0.004961435 | 0.009326546 |
| LILRA4 | -0.216064222 | 7.42E-07 | 2.66E-06 |
| LILRB1 | -0.128295023 | 0.003540237 | 0.006821117 |
| LILRB5 | -0.165080603 | 0.000167874 | 0.000411662 |
| LILRP2 | 0.181233863 | 3.52E-05 | 9.64E-05 |
| LIMCH1 | -0.203956989 | 3.07E-06 | 9.93E-06 |
| LIMD1 | -0.292308287 | 1.33E-11 | 9.75E-11 |
| LIMD2 | -0.137589619 | 0.001749758 | 0.00356116 |
| LIME1 | -0.123962215 | 0.004844616 | 0.009129271 |
| LIMK2 | -0.168137895 | 0.000126225 | 0.000316004 |
| LIMS1 | 0.114557149 | 0.009269015 | 0.016558144 |
| LIMS2 | -0.408076164 | 4.37E-22 | 1.56E-20 |
| LIMS3 | 0.100483324 | 0.022574308 | 0.037198291 |
| LIN28A | 0.182024208 | 3.25E-05 | 8.93E-05 |
| LIN28B | 0.25023191 | 8.58E-09 | 4.15E-08 |
| LIN52 | 0.159111247 | 0.000288789 | 0.000677184 |
| LIN54 | 0.300411306 | 3.35E-12 | 2.68E-11 |
| LIN7C | 0.146829461 | 0.000831397 | 0.00179266 |
| LIN9 | 0.417217731 | 4.15E-23 | 1.73E-21 |
| LINGO2 | 0.159821468 | 0.000271005 | 0.000638704 |
| LINGO3 | -0.19898014 | 5.36E-06 | 1.67E-05 |
| LINGO4 | -0.185789718 | 2.21E-05 | 6.25E-05 |
| LINS1 | -0.113611464 | 0.009870053 | 0.017553396 |
| LIPA | -0.211046553 | 1.35E-06 | 4.64E-06 |
| LIPE | -0.201928981 | 3.86E-06 | 1.23E-05 |
| LIPF | -0.147690487 | 0.000773968 | 0.001679522 |
| LIPH | -0.202741853 | 3.52E-06 | 1.13E-05 |
| LIPJ | -0.131630633 | 0.002762963 | 0.005429228 |
| LIPK | 0.121994383 | 0.005569132 | 0.010375227 |
| LIPN | -0.126454635 | 0.004049425 | 0.007724748 |
| LIPT1 | -0.100165387 | 0.023007461 | 0.037862106 |
| LIPT2 | 0.115063358 | 0.00896086 | 0.016039193 |
| LITAF | -0.212733882 | 1.11E-06 | 3.85E-06 |
| LIX1L | -0.182343957 | 3.14E-05 | 8.67E-05 |
| LLGL2 | -0.20665651 | 2.25E-06 | 7.45E-06 |
| LLPH | 0.373774292 | 1.61E-18 | 3.37E-17 |
| LMAN1L | -0.164676639 | 0.000174255 | 0.00042642 |
| LMAN1 | 0.27698791 | 1.59E-10 | 9.94E-10 |
| LMBR1 | 0.243668942 | 2.13E-08 | 9.71E-08 |
| LMBRD1 | -0.170201801 | 0.000103829 | 0.000263636 |
| LMCD1 | -0.124011691 | 0.004827549 | 0.009098826 |
| LMF1 | -0.383671736 | 1.66E-19 | 4.06E-18 |
| LMF2 | -0.278380093 | 1.28E-10 | 8.11E-10 |
| LMLN | -0.142592294 | 0.001175885 | 0.002470314 |
| LMNA | -0.103313502 | 0.019020045 | 0.031848258 |
| LMNB1 | 0.383317539 | 1.80E-19 | 4.38E-18 |
| LMNB2 | 0.324556604 | 4.26E-14 | 4.58E-13 |
| LMO1 | 0.148689909 | 0.000711922 | 0.001556201 |
| LMO2 | -0.305442122 | 1.39E-12 | 1.19E-11 |
| LMO3 | -0.309232806 | 7.11E-13 | 6.38E-12 |
| LMO7 | -0.18234798 | 3.14E-05 | 8.67E-05 |
| LMOD1 | -0.316629732 | 1.86E-13 | 1.82E-12 |
| LMOD3 | -0.31098366 | 5.20E-13 | 4.75E-12 |
| LMTK3 | -0.167658979 | 0.000132034 | 0.000329269 |
| LMX1A | 0.095195902 | 0.030772916 | 0.049258771 |
| LNX1 | -0.123884279 | 0.004871609 | 0.009174948 |
| LNX2 | -0.14849275 | 0.000723785 | 0.001580231 |
| LOC100009676 | -0.189037521 | 1.57E-05 | 4.57E-05 |
| LOC100101266 | -0.216907559 | 6.70E-07 | 2.42E-06 |
| LOC100124692 | -0.10047896 | 0.022580206 | 0.037201876 |
| LOC100125556 | 0.121585293 | 0.005731466 | 0.010646891 |
| LOC100128076 | -0.109972793 | 0.012518049 | 0.021749892 |
| LOC100128164 | -0.198797299 | 5.47E-06 | 1.71E-05 |
| LOC100128191 | 0.289707585 | 2.04E-11 | 1.46E-10 |
| LOC100128239 | -0.30674165 | 1.11E-12 | 9.64E-12 |
| LOC100128288 | -0.283560516 | 5.59E-11 | 3.72E-10 |
| LOC100128554 | -0.160524731 | 0.000254408 | 0.000602716 |
| LOC100128573 | -0.189363254 | 1.52E-05 | 4.43E-05 |
| LOC100128640 | -0.095040959 | 0.031047363 | 0.049657893 |
| LOC100128675 | -0.205556067 | 2.55E-06 | 8.38E-06 |
| LOC100128788 | -0.1407661 | 0.001361507 | 0.002826526 |
| LOC100128842 | -0.277467541 | 1.48E-10 | 9.28E-10 |
| LOC100129034 | -0.284825011 | 4.55E-11 | 3.08E-10 |
| LOC100129387 | -0.217854639 | 5.98E-07 | 2.18E-06 |
| LOC100129534 | -0.321731939 | 7.24E-14 | 7.50E-13 |
| LOC100129550 | -0.346117815 | 6.12E-16 | 8.73E-15 |
| LOC100129637 | -0.135526609 | 0.00205375 | 0.004136472 |
| LOC100129716 | -0.206838247 | 2.20E-06 | 7.31E-06 |
| LOC100129726 | -0.245531168 | 1.65E-08 | 7.64E-08 |
| LOC100130015 | -0.205215297 | 2.66E-06 | 8.69E-06 |
| LOC100130093 | -0.250254163 | 8.55E-09 | 4.14E-08 |
| LOC100130331 | 0.125253071 | 0.004416717 | 0.008371073 |
| LOC100130522 | -0.14344389 | 0.001097558 | 0.002318047 |
| LOC100130557 | -0.343892861 | 9.63E-16 | 1.33E-14 |
| LOC100130581 | -0.182268167 | 3.17E-05 | 8.73E-05 |
| LOC100130872 | -0.115040563 | 0.008974538 | 0.016059362 |
| LOC100130932 | 0.206257513 | 2.36E-06 | 7.78E-06 |
| LOC100130933 | -0.174225955 | 7.05E-05 | 0.000183608 |
| LOC100130987 | -0.147760792 | 0.000769445 | 0.00167025 |
| LOC100131193 | -0.247085113 | 1.33E-08 | 6.24E-08 |
| LOC100131434 | -0.393904012 | 1.46E-20 | 4.10E-19 |
| LOC100131551 | 0.257686471 | 2.95E-09 | 1.52E-08 |
| LOC100131691 | -0.225966497 | 2.19E-07 | 8.53E-07 |
| LOC100131726 | 0.308024886 | 8.82E-13 | 7.79E-12 |
| LOC100132111 | 0.159903184 | 0.000269026 | 0.000634638 |
| LOC100132215 | -0.131834132 | 0.002720999 | 0.005356775 |
| LOC100132247 | -0.286481423 | 3.48E-11 | 2.39E-10 |
| LOC100132287 | -0.266923773 | 7.52E-10 | 4.26E-09 |
| LOC100132288 | -0.107230398 | 0.014910111 | 0.025548504 |
| LOC100132354 | 0.154538976 | 0.000432048 | 0.000980781 |
| LOC100132707 | -0.381614539 | 2.68E-19 | 6.39E-18 |
| LOC100132724 | 0.139957362 | 0.001452021 | 0.002999199 |
| LOC100132831 | 0.122694268 | 0.005300973 | 0.00991168 |
| LOC100132832 | -0.301236239 | 2.90E-12 | 2.35E-11 |
| LOC100133050 | -0.125318382 | 0.004396004 | 0.00833735 |
| LOC100133161 | -0.246049474 | 1.54E-08 | 7.14E-08 |
| LOC100133331 | -0.318440752 | 1.34E-13 | 1.33E-12 |
| LOC100133469 | 0.111856172 | 0.011078003 | 0.019479822 |
| LOC100133545 | -0.111248375 | 0.011525817 | 0.020192779 |
| LOC100133669 | -0.110051654 | 0.012454587 | 0.021651647 |
| LOC100133985 | 0.177188505 | 5.27E-05 | 0.000140023 |
| LOC100134368 | -0.146051662 | 0.000886638 | 0.001902331 |
| LOC100144604 | -0.2676893 | 6.70E-10 | 3.82E-09 |
| LOC100170939 | -0.298766838 | 4.44E-12 | 3.48E-11 |
| LOC100188949 | -0.227590811 | 1.78E-07 | 7.04E-07 |
| LOC100189589 | -0.182672131 | 3.04E-05 | 8.41E-05 |
| LOC100190938 | -0.15413866 | 0.000447325 | 0.001012128 |
| LOC100190939 | -0.164806157 | 0.000172184 | 0.000421768 |
| LOC100190940 | 0.205597154 | 2.54E-06 | 8.35E-06 |
| LOC100190986 | -0.316666189 | 1.85E-13 | 1.81E-12 |
| LOC100216001 | 0.186153516 | 2.12E-05 | 6.05E-05 |
| LOC100216545 | -0.118604442 | 0.007048594 | 0.012873474 |
| LOC100233209 | -0.121677337 | 0.005694575 | 0.010585248 |
| LOC100240726 | -0.225134179 | 2.43E-07 | 9.40E-07 |
| LOC100270746 | -0.140478419 | 0.001393092 | 0.002887001 |
| LOC100270804 | -0.323969521 | 4.76E-14 | 5.06E-13 |
| LOC100271722 | -0.283780687 | 5.39E-11 | 3.60E-10 |
| LOC100271836 | -0.162334096 | 0.000215972 | 0.000519539 |
| LOC100272146 | -0.142960458 | 0.001141411 | 0.002403807 |
| LOC100272216 | -0.269451444 | 5.12E-10 | 2.97E-09 |
| LOC100272217 | -0.170728527 | 9.87E-05 | 0.000251557 |
| LOC100272228 | -0.34370318 | 1.00E-15 | 1.38E-14 |
| LOC100286793 | 0.107662981 | 0.014507971 | 0.024902132 |
| LOC100286844 | -0.14355349 | 0.001087835 | 0.002299454 |
| LOC100287718 | -0.205609467 | 2.54E-06 | 8.34E-06 |
| LOC100288778 | -0.226090643 | 2.15E-07 | 8.40E-07 |
| LOC100302401 | 0.18380142 | 2.71E-05 | 7.56E-05 |
| LOC100302640 | -0.161264407 | 0.000237981 | 0.000566938 |
| LOC100302650 | -0.26605463 | 8.57E-10 | 4.81E-09 |
| LOC100303728 | -0.316040864 | 2.08E-13 | 2.01E-12 |
| LOC113230 | -0.210919789 | 1.37E-06 | 4.70E-06 |
| LOC115110 | -0.450891302 | 3.72E-27 | 2.81E-25 |
| LOC116437 | 0.14126519 | 0.001308268 | 0.002724775 |
| LOC121838 | -0.208502055 | 1.82E-06 | 6.12E-06 |
| LOC121952 | -0.35299713 | 1.47E-16 | 2.28E-15 |
| LOC127841 | 0.163641121 | 0.000191666 | 0.000465268 |
| LOC134466 | -0.105133911 | 0.017000901 | 0.028741774 |
| LOC143188 | -0.201974369 | 3.84E-06 | 1.22E-05 |
| LOC143666 | -0.172484197 | 8.34E-05 | 0.000214587 |
| LOC144438 | 0.212082258 | 1.19E-06 | 4.13E-06 |
| LOC144571 | -0.237186452 | 5.11E-08 | 2.19E-07 |
| LOC144776 | 0.102104141 | 0.020474278 | 0.034046577 |
| LOC145783 | 0.153556516 | 0.000470441 | 0.001058915 |
| LOC145820 | -0.21464783 | 8.80E-07 | 3.12E-06 |
| LOC146880 | -0.399474112 | 3.75E-21 | 1.17E-19 |
| LOC147727 | -0.269939027 | 4.76E-10 | 2.77E-09 |
| LOC147804 | 0.110502172 | 0.012097457 | 0.021086943 |
| LOC148145 | -0.247202469 | 1.31E-08 | 6.15E-08 |
| LOC148413 | -0.150638129 | 0.00060401 | 0.001335652 |
| LOC148696 | -0.388512476 | 5.31E-20 | 1.38E-18 |
| LOC148709 | 0.095482966 | 0.030269933 | 0.048566015 |
| LOC149620 | -0.386645808 | 8.26E-20 | 2.09E-18 |
| LOC150197 | -0.148967359 | 0.000695534 | 0.001523209 |
| LOC150622 | -0.20613332 | 2.39E-06 | 7.88E-06 |
| LOC150776 | -0.247381656 | 1.28E-08 | 6.01E-08 |
| LOC150786 | 0.291735168 | 1.46E-11 | 1.07E-10 |
| LOC151009 | -0.246062125 | 1.53E-08 | 7.13E-08 |
| LOC151162 | -0.098149809 | 0.025924454 | 0.04219298 |
| LOC151174 | -0.121979469 | 0.005574977 | 0.010385148 |
| LOC152217 | 0.182202125 | 3.19E-05 | 8.78E-05 |
| LOC152225 | 0.231080674 | 1.14E-07 | 4.65E-07 |
| LOC153684 | -0.356545243 | 6.97E-17 | 1.14E-15 |
| LOC157381 | -0.183871816 | 2.69E-05 | 7.51E-05 |
| LOC158376 | -0.167515432 | 0.000133823 | 0.000333275 |
| LOC158572 | -0.245772082 | 1.60E-08 | 7.41E-08 |
| LOC158696 | -0.229858723 | 1.33E-07 | 5.38E-07 |
| LOC162632 | -0.133902986 | 0.002326166 | 0.004635171 |
| LOC168474 | -0.211250194 | 1.32E-06 | 4.53E-06 |
| LOC200030 | -0.29526324 | 8.07E-12 | 6.13E-11 |
| LOC202181 | -0.4368295 | 2.07E-25 | 1.23E-23 |
| LOC202781 | -0.15607944 | 0.000377676 | 0.000864026 |
| LOC220429 | 0.207528721 | 2.03E-06 | 6.79E-06 |
| LOC220594 | -0.116223085 | 0.008289017 | 0.014941011 |
| LOC221442 | -0.283704673 | 5.46E-11 | 3.64E-10 |
| LOC221710 | 0.374959265 | 1.23E-18 | 2.64E-17 |
| LOC222699 | 0.117398102 | 0.007654509 | 0.01389126 |
| LOC253039 | -0.265079657 | 9.92E-10 | 5.53E-09 |
| LOC255167 | -0.185339049 | 2.31E-05 | 6.52E-05 |
| LOC256880 | -0.1470251 | 0.000818012 | 0.001767232 |
| LOC257358 | -0.120809232 | 0.006051158 | 0.011185661 |
| LOC26102 | -0.157710206 | 0.000327111 | 0.000758239 |
| LOC282997 | -0.147309541 | 0.000798908 | 0.001728762 |
| LOC283050 | -0.288471905 | 2.51E-11 | 1.76E-10 |
| LOC283070 | -0.414257762 | 8.97E-23 | 3.53E-21 |
| LOC283174 | -0.419443529 | 2.31E-23 | 1.00E-21 |
| LOC283267 | -0.097016394 | 0.027700793 | 0.044807272 |
| LOC283314 | -0.234559495 | 7.23E-08 | 3.04E-07 |
| LOC283404 | 0.206141736 | 2.39E-06 | 7.88E-06 |
| LOC283663 | -0.306621279 | 1.13E-12 | 9.83E-12 |
| LOC283731 | -0.216267814 | 7.24E-07 | 2.60E-06 |
| LOC283856 | -0.253407748 | 5.47E-09 | 2.71E-08 |
| LOC283867 | -0.105602849 | 0.016512175 | 0.027986548 |
| LOC283922 | -0.335391655 | 5.27E-15 | 6.52E-14 |
| LOC284009 | -0.139426964 | 0.001514352 | 0.003119536 |
| LOC284023 | -0.205430079 | 2.59E-06 | 8.49E-06 |
| LOC284233 | -0.254022145 | 5.01E-09 | 2.50E-08 |
| LOC284276 | -0.271354231 | 3.83E-10 | 2.26E-09 |
| LOC284440 | -0.464862236 | 5.68E-29 | 5.33E-27 |
| LOC284441 | 0.10703857 | 0.015091551 | 0.02582177 |
| LOC284578 | -0.328151003 | 2.15E-14 | 2.41E-13 |
| LOC284632 | -0.105920649 | 0.016188001 | 0.027483714 |
| LOC284688 | 0.101165754 | 0.021668378 | 0.03585921 |
| LOC284749 | -0.245561888 | 1.64E-08 | 7.61E-08 |
| LOC284837 | -0.401108138 | 2.51E-21 | 8.05E-20 |
| LOC284900 | -0.340738588 | 1.82E-15 | 2.43E-14 |
| LOC285359 | -0.164564675 | 0.000176063 | 0.00043074 |
| LOC285419 | -0.23531979 | 6.54E-08 | 2.77E-07 |
| LOC285456 | -0.128958859 | 0.003371324 | 0.006521438 |
| LOC285501 | 0.111281071 | 0.011501325 | 0.020158584 |
| LOC285593 | -0.317710628 | 1.53E-13 | 1.51E-12 |
| LOC285629 | -0.138958413 | 0.001571449 | 0.003223865 |
| LOC285696 | 0.163926142 | 0.000186717 | 0.000454028 |
| LOC285735 | -0.098773219 | 0.024989942 | 0.040795407 |
| LOC285768 | -0.19431866 | 8.93E-06 | 2.69E-05 |
| LOC285780 | -0.124508164 | 0.004659275 | 0.00880325 |
| LOC285796 | -0.174037254 | 7.18E-05 | 0.000186645 |
| LOC285830 | -0.229910647 | 1.32E-07 | 5.35E-07 |
| LOC285847 | -0.101994331 | 0.020610989 | 0.034259663 |
| LOC286367 | -0.358299518 | 4.80E-17 | 8.04E-16 |
| LOC286467 | 0.24747563 | 1.26E-08 | 5.94E-08 |
| LOC29034 | 0.247704275 | 1.22E-08 | 5.77E-08 |
| LOC338651 | -0.165178982 | 0.000166353 | 0.000408134 |
| LOC338758 | -0.188397423 | 1.68E-05 | 4.86E-05 |
| LOC338799 | -0.243795507 | 2.10E-08 | 9.55E-08 |
| LOC339047 | -0.36720796 | 6.94E-18 | 1.33E-16 |
| LOC339240 | -0.102139934 | 0.020429887 | 0.033981241 |
| LOC339290 | -0.25548221 | 4.06E-09 | 2.05E-08 |
| LOC339524 | -0.277782356 | 1.41E-10 | 8.86E-10 |
| LOC339568 | 0.097310563 | 0.027229985 | 0.044109971 |
| LOC339674 | 0.19163665 | 1.19E-05 | 3.53E-05 |
| LOC339788 | -0.108351335 | 0.013887707 | 0.023927893 |
| LOC340357 | -0.144311159 | 0.001022758 | 0.002172927 |
| LOC340508 | -0.202359797 | 3.67E-06 | 1.18E-05 |
| LOC341056 | 0.134742108 | 0.002181507 | 0.004371324 |
| LOC342346 | -0.345952737 | 6.33E-16 | 9.01E-15 |
| LOC344595 | -0.160846082 | 0.000247145 | 0.000587179 |
| LOC349114 | -0.261586261 | 1.67E-09 | 8.93E-09 |
| LOC349196 | -0.280205427 | 9.57E-11 | 6.18E-10 |
| LOC375190 | -0.177771493 | 4.97E-05 | 0.000132902 |
| LOC388152 | -0.359312566 | 3.86E-17 | 6.59E-16 |
| LOC388387 | -0.260841758 | 1.86E-09 | 9.89E-09 |
| LOC388588 | -0.234122863 | 7.66E-08 | 3.20E-07 |
| LOC388692 | -0.127849752 | 0.0036578 | 0.007025283 |
| LOC388789 | 0.1312558 | 0.002841804 | 0.005572809 |
| LOC388796 | 0.159198737 | 0.000286541 | 0.000672306 |
| LOC388955 | 0.130436273 | 0.00302136 | 0.005896977 |
| LOC389033 | -0.167072886 | 0.000139485 | 0.000346597 |
| LOC389634 | -0.216185371 | 7.32E-07 | 2.62E-06 |
| LOC389705 | -0.129977528 | 0.003126316 | 0.006085189 |
| LOC389791 | -0.120657859 | 0.00611535 | 0.011295964 |
| LOC390595 | -0.280013804 | 9.87E-11 | 6.37E-10 |
| LOC391322 | -0.168420261 | 0.000122913 | 0.00030837 |
| LOC399744 | -0.22567241 | 2.27E-07 | 8.83E-07 |
| LOC399815 | 0.307577037 | 9.55E-13 | 8.39E-12 |
| LOC400027 | -0.295844926 | 7.31E-12 | 5.59E-11 |
| LOC400657 | -0.195814951 | 7.59E-06 | 2.31E-05 |
| LOC400752 | -0.28490361 | 4.49E-11 | 3.04E-10 |
| LOC400794 | -0.327354488 | 2.50E-14 | 2.78E-13 |
| LOC400804 | -0.15247042 | 0.000516563 | 0.001155316 |
| LOC400891 | -0.283749771 | 5.42E-11 | 3.62E-10 |
| LOC400927 | -0.261633573 | 1.66E-09 | 8.88E-09 |
| LOC400931 | -0.273182409 | 2.89E-10 | 1.73E-09 |
| LOC400940 | -0.117601663 | 0.007549109 | 0.013714924 |
| LOC401010 | 0.187623099 | 1.82E-05 | 5.25E-05 |
| LOC401052 | -0.14043488 | 0.00139793 | 0.002896127 |
| LOC401093 | -0.356600698 | 6.89E-17 | 1.13E-15 |
| LOC401127 | -0.120949472 | 0.005992228 | 0.011089034 |
| LOC401387 | -0.098250752 | 0.025771124 | 0.041971255 |
| LOC401431 | -0.168113906 | 0.00012651 | 0.000316678 |
| LOC401463 | -0.210490307 | 1.44E-06 | 4.93E-06 |
| LOC401588 | -0.117100677 | 0.007810875 | 0.014153184 |
| LOC407835 | 0.164854263 | 0.000171421 | 0.000420001 |
| LOC440173 | 0.095184596 | 0.030792872 | 0.049286348 |
| LOC440461 | -0.200560403 | 4.50E-06 | 1.42E-05 |
| LOC440563 | 0.104866112 | 0.017285639 | 0.029161491 |
| LOC440896 | -0.291542551 | 1.51E-11 | 1.10E-10 |
| LOC440944 | -0.198980201 | 5.36E-06 | 1.67E-05 |
| LOC441089 | 0.315167068 | 2.44E-13 | 2.34E-12 |
| LOC441177 | 0.276969356 | 1.60E-10 | 9.96E-10 |
| LOC441204 | -0.306189132 | 1.22E-12 | 1.05E-11 |
| LOC441208 | 0.098326454 | 0.025656647 | 0.041791627 |
| LOC441454 | -0.137569301 | 0.001752539 | 0.003566458 |
| LOC441666 | 0.123561757 | 0.004984765 | 0.009367759 |
| LOC441869 | -0.323186835 | 5.51E-14 | 5.79E-13 |
| LOC442308 | 0.182269234 | 3.17E-05 | 8.73E-05 |
| LOC442454 | 0.259183008 | 2.38E-09 | 1.24E-08 |
| LOC442459 | 0.096691309 | 0.028229209 | 0.045588222 |
| LOC541471 | 0.331358173 | 1.16E-14 | 1.35E-13 |
| LOC541473 | -0.242202074 | 2.60E-08 | 1.17E-07 |
| LOC550643 | 0.150633763 | 0.000604234 | 0.001335999 |
| LOC554202 | 0.187592623 | 1.83E-05 | 5.26E-05 |
| LOC572558 | -0.230019138 | 1.30E-07 | 5.28E-07 |
| LOC606724 | -0.19168549 | 1.19E-05 | 3.52E-05 |
| LOC619207 | -0.250495015 | 8.27E-09 | 4.00E-08 |
| LOC641298 | -0.148658355 | 0.000713809 | 0.001559813 |
| LOC642826 | -0.205667138 | 2.52E-06 | 8.29E-06 |
| LOC643008 | -0.242334602 | 2.56E-08 | 1.15E-07 |
| LOC643486 | 0.14327024 | 0.001113128 | 0.002349194 |
| LOC643677 | -0.103037645 | 0.019343585 | 0.032327724 |
| LOC643719 | -0.262131835 | 1.54E-09 | 8.31E-09 |
| LOC644145 | 0.11978438 | 0.006497852 | 0.011945099 |
| LOC644165 | -0.41249422 | 1.41E-22 | 5.35E-21 |
| LOC644172 | -0.191527107 | 1.21E-05 | 3.57E-05 |
| LOC645166 | 0.318279919 | 1.38E-13 | 1.37E-12 |
| LOC645323 | -0.130621174 | 0.00297997 | 0.005822448 |
| LOC645431 | -0.119573689 | 0.006593268 | 0.012108255 |
| LOC645676 | -0.16621459 | 0.000151113 | 0.000373401 |
| LOC645752 | -0.12163648 | 0.005710924 | 0.010610704 |
| LOC646471 | -0.329559339 | 1.64E-14 | 1.88E-13 |
| LOC646762 | -0.120844972 | 0.006036091 | 0.011162971 |
| LOC646851 | -0.241895331 | 2.71E-08 | 1.22E-07 |
| LOC646982 | -0.157923415 | 0.000320988 | 0.000745256 |
| LOC646999 | 0.107992427 | 0.014208137 | 0.024444573 |
| LOC647121 | -0.253451779 | 5.44E-09 | 2.70E-08 |
| LOC647288 | 0.15896269 | 0.000292644 | 0.000685098 |
| LOC647946 | 0.290599836 | 1.76E-11 | 1.27E-10 |
| LOC647979 | -0.126529456 | 0.004027495 | 0.007685848 |
| LOC648691 | 0.164303096 | 0.000180357 | 0.000440114 |
| LOC648740 | -0.442285275 | 4.46E-26 | 2.91E-24 |
| LOC649330 | 0.303032018 | 2.12E-12 | 1.76E-11 |
| LOC650368 | -0.132073187 | 0.002672446 | 0.005269492 |
| LOC650623 | -0.200280641 | 4.64E-06 | 1.46E-05 |
| LOC651250 | -0.303874605 | 1.83E-12 | 1.54E-11 |
| LOC653113 | -0.133466943 | 0.002404747 | 0.004780791 |
| LOC653501 | -0.364440013 | 1.27E-17 | 2.33E-16 |
| LOC653566 | 0.248773177 | 1.05E-08 | 5.02E-08 |
| LOC653786 | -0.161582175 | 0.000231233 | 0.000552735 |
| LOC654342 | 0.203741395 | 3.14E-06 | 1.02E-05 |
| LOC678655 | -0.109318936 | 0.013055321 | 0.022618511 |
| LOC723809 | -0.401593442 | 2.22E-21 | 7.20E-20 |
| LOC723972 | 0.136057694 | 0.001971187 | 0.003980208 |
| LOC727896 | 0.275232021 | 2.10E-10 | 1.29E-09 |
| LOC728264 | -0.290869294 | 1.69E-11 | 1.22E-10 |
| LOC728392 | -0.424397681 | 6.21E-24 | 2.95E-22 |
| LOC728554 | 0.219149093 | 5.10E-07 | 1.88E-06 |
| LOC728643 | 0.15749277 | 0.000333467 | 0.000771184 |
| LOC728723 | -0.149813842 | 0.000647669 | 0.001425099 |
| LOC728743 | -0.141224626 | 0.001312523 | 0.002732782 |
| LOC728758 | 0.156676573 | 0.00035837 | 0.000824388 |
| LOC728875 | 0.145412812 | 0.000934527 | 0.001996507 |
| LOC728989 | -0.312325497 | 4.08E-13 | 3.79E-12 |
| LOC729020 | 0.306337436 | 1.19E-12 | 1.03E-11 |
| LOC729082 | 0.190627715 | 1.33E-05 | 3.90E-05 |
| LOC729176 | 0.234429821 | 7.35E-08 | 3.09E-07 |
| LOC729234 | -0.317004147 | 1.74E-13 | 1.70E-12 |
| LOC729375 | -0.098902687 | 0.024799556 | 0.040511076 |
| LOC729603 | -0.254900495 | 4.42E-09 | 2.22E-08 |
| LOC729609 | -0.115961956 | 0.008436244 | 0.015176279 |
| LOC729678 | -0.151097217 | 0.000580895 | 0.001287243 |
| LOC729799 | -0.29654522 | 6.49E-12 | 4.99E-11 |
| LOC730668 | -0.152909882 | 0.000497417 | 0.001114864 |
| LOC80054 | -0.345174431 | 7.42E-16 | 1.04E-14 |
| LOC80154 | -0.394207022 | 1.36E-20 | 3.83E-19 |
| LOC81691 | 0.119898882 | 0.006446518 | 0.011857275 |
| LOC90110 | -0.337444542 | 3.51E-15 | 4.48E-14 |
| LOC90246 | -0.095526935 | 0.030193518 | 0.048458971 |
| LOC90586 | -0.221420345 | 3.86E-07 | 1.45E-06 |
| LOC90834 | -0.348630455 | 3.65E-16 | 5.40E-15 |
| LOC91316 | -0.374324637 | 1.42E-18 | 3.02E-17 |
| LOC91450 | -0.278011924 | 1.36E-10 | 8.56E-10 |
| LOC92249 | -0.240106174 | 3.46E-08 | 1.52E-07 |
| LOC92659 | 0.118797132 | 0.006955893 | 0.012716948 |
| LOC92973 | -0.505502543 | 9.31E-35 | 2.55E-32 |
| LOH12CR1 | 0.246521497 | 1.44E-08 | 6.73E-08 |
| LOH3CR2A | -0.191182184 | 1.25E-05 | 3.69E-05 |
| LONRF1 | -0.162414227 | 0.000214402 | 0.000516259 |
| LONRF2 | -0.23209522 | 9.97E-08 | 4.10E-07 |
| LOR | -0.280601365 | 8.99E-11 | 5.83E-10 |
| LOXHD1 | -0.17093356 | 9.68E-05 | 0.000246961 |
| LOXL1 | -0.106999434 | 0.015128805 | 0.025874438 |
| LOXL2 | 0.281926899 | 7.27E-11 | 4.77E-10 |
| LOXL3 | -0.119593849 | 0.006584085 | 0.012093612 |
| LOXL4 | -0.294516925 | 9.15E-12 | 6.90E-11 |
| LOX | 0.208073434 | 1.91E-06 | 6.41E-06 |
| LPAL2 | -0.185616139 | 2.25E-05 | 6.35E-05 |
| LPAR1 | 0.128223316 | 0.003558935 | 0.006851859 |
| LPAR2 | -0.095527035 | 0.030193344 | 0.048458971 |
| LPAR3 | -0.130699058 | 0.002962691 | 0.005792084 |
| LPAR4 | 0.117844464 | 0.00742509 | 0.013510486 |
| LPAR6 | -0.172130795 | 8.63E-05 | 0.000221503 |
| LPA | -0.15641871 | 0.000366592 | 0.000841073 |
| LPCAT1 | -0.235936436 | 6.03E-08 | 2.56E-07 |
| LPCAT2 | -0.290264803 | 1.86E-11 | 1.34E-10 |
| LPCAT3 | -0.188100259 | 1.73E-05 | 5.01E-05 |
| LPGAT1 | 0.231256239 | 1.11E-07 | 4.55E-07 |
| LPHN1 | -0.252818022 | 5.95E-09 | 2.94E-08 |
| LPIN1 | -0.244052577 | 2.02E-08 | 9.25E-08 |
| LPIN2 | -0.163199607 | 0.000199574 | 0.000482999 |
| LPIN3 | -0.260353324 | 2.00E-09 | 1.06E-08 |
| LPL | -0.240839792 | 3.13E-08 | 1.39E-07 |
| LPPR1 | -0.207505745 | 2.04E-06 | 6.80E-06 |
| LPPR2 | -0.230235409 | 1.27E-07 | 5.15E-07 |
| LPPR3 | -0.111375618 | 0.011430763 | 0.020048973 |
| LPPR4 | -0.337631899 | 3.38E-15 | 4.33E-14 |
| LPPR5 | -0.109873213 | 0.012598592 | 0.021878424 |
| LPXN | -0.208862947 | 1.74E-06 | 5.88E-06 |
| LRAT | -0.191530316 | 1.21E-05 | 3.57E-05 |
| LRCH1 | -0.10230238 | 0.020229469 | 0.0336703 |
| LRCH2 | -0.106631326 | 0.015483181 | 0.026401453 |
| LRCH4 | -0.366096062 | 8.87E-18 | 1.68E-16 |
| LRDD | -0.247272239 | 1.30E-08 | 6.10E-08 |
| LRFN1 | -0.114341783 | 0.009402953 | 0.016776419 |
| LRFN3 | -0.096560448 | 0.028444351 | 0.045902284 |
| LRFN4 | 0.226311259 | 2.09E-07 | 8.20E-07 |
| LRGUK | -0.229664674 | 1.37E-07 | 5.51E-07 |
| LRIG1 | -0.32553282 | 3.54E-14 | 3.85E-13 |
| LRIG2 | -0.193643376 | 9.61E-06 | 2.89E-05 |
| LRIG3 | -0.331046737 | 1.23E-14 | 1.43E-13 |
| LRIT1 | 0.131376575 | 0.00281618 | 0.005526193 |
| LRIT3 | -0.31027633 | 5.90E-13 | 5.36E-12 |
| LRMP | -0.247351853 | 1.28E-08 | 6.03E-08 |
| LRP11 | 0.118911394 | 0.00690144 | 0.01262779 |
| LRP12 | 0.231937295 | 1.02E-07 | 4.19E-07 |
| LRP1 | -0.240226632 | 3.40E-08 | 1.50E-07 |
| LRP2BP | -0.463814523 | 7.83E-29 | 7.25E-27 |
| LRP2 | -0.190015237 | 1.42E-05 | 4.15E-05 |
| LRP3 | -0.204219169 | 2.97E-06 | 9.66E-06 |
| LRP5L | -0.296409485 | 6.64E-12 | 5.11E-11 |
| LRP6 | -0.146897557 | 0.000826715 | 0.001784491 |
| LRP8 | 0.203266769 | 3.31E-06 | 1.07E-05 |
| LRPAP1 | -0.108808667 | 0.013488633 | 0.023290497 |
| LRPPRC | 0.364413132 | 1.28E-17 | 2.35E-16 |
| LRRC10B | -0.119602746 | 0.006580035 | 0.012088395 |
| LRRC16B | -0.138729848 | 0.001600013 | 0.003278426 |
| LRRC17 | -0.109769851 | 0.012682678 | 0.022009148 |
| LRRC18 | -0.254123961 | 4.94E-09 | 2.46E-08 |
| LRRC1 | 0.213104589 | 1.06E-06 | 3.70E-06 |
| LRRC23 | -0.168708975 | 0.000119611 | 0.000300502 |
| LRRC25 | -0.122418435 | 0.005405238 | 0.010094356 |
| LRRC27 | -0.299690458 | 3.79E-12 | 3.01E-11 |
| LRRC28 | 0.132750464 | 0.002539142 | 0.005024987 |
| LRRC29 | -0.264413066 | 1.10E-09 | 6.06E-09 |
| LRRC2 | -0.17777035 | 4.97E-05 | 0.000132902 |
| LRRC31 | -0.286768342 | 3.32E-11 | 2.29E-10 |
| LRRC32 | -0.105637979 | 0.016476064 | 0.027934817 |
| LRRC33 | -0.263247628 | 1.30E-09 | 7.10E-09 |
| LRRC36 | -0.422983799 | 9.06E-24 | 4.15E-22 |
| LRRC37A2 | -0.27690851 | 1.61E-10 | 1.01E-09 |
| LRRC37A3 | -0.101162791 | 0.021672242 | 0.035862636 |
| LRRC37A4 | -0.315379608 | 2.34E-13 | 2.26E-12 |
| LRRC37A | -0.317884509 | 1.48E-13 | 1.47E-12 |
| LRRC37B | -0.314437257 | 2.78E-13 | 2.64E-12 |
| LRRC39 | -0.27303094 | 2.96E-10 | 1.77E-09 |
| LRRC3B | -0.129152943 | 0.00332334 | 0.006440462 |
| LRRC3 | -0.244716305 | 1.85E-08 | 8.49E-08 |
| LRRC40 | 0.305694092 | 1.33E-12 | 1.14E-11 |
| LRRC42 | 0.359761428 | 3.51E-17 | 6.04E-16 |
| LRRC43 | -0.127609911 | 0.003722581 | 0.007142839 |
| LRRC46 | -0.204598805 | 2.85E-06 | 9.28E-06 |
| LRRC48 | -0.309244318 | 7.10E-13 | 6.37E-12 |
| LRRC49 | -0.124405973 | 0.004693472 | 0.008864512 |
| LRRC4B | -0.313263357 | 3.45E-13 | 3.24E-12 |
| LRRC4C | -0.205501779 | 2.57E-06 | 8.43E-06 |
| LRRC4 | -0.258101157 | 2.78E-09 | 1.43E-08 |
| LRRC50 | -0.241039343 | 3.05E-08 | 1.35E-07 |
| LRRC52 | -0.25569365 | 3.94E-09 | 1.99E-08 |
| LRRC55 | -0.302716833 | 2.24E-12 | 1.85E-11 |
| LRRC56 | -0.28448236 | 4.81E-11 | 3.24E-10 |
| LRRC59 | 0.473467467 | 3.92E-30 | 4.45E-28 |
| LRRC61 | 0.245107955 | 1.75E-08 | 8.07E-08 |
| LRRC67 | -0.197582854 | 6.25E-06 | 1.93E-05 |
| LRRC70 | -0.213144296 | 1.05E-06 | 3.68E-06 |
| LRRC8C | -0.164692534 | 0.000173999 | 0.0004259 |
| LRRC8E | -0.112890547 | 0.010351242 | 0.018316273 |
| LRRFIP1 | -0.099699647 | 0.023654967 | 0.038799893 |
| LRRIQ1 | -0.147555788 | 0.000782703 | 0.001697003 |
| LRRIQ4 | 0.185186748 | 2.35E-05 | 6.62E-05 |
| LRRK1 | -0.25238262 | 6.33E-09 | 3.11E-08 |
| LRRK2 | -0.406713539 | 6.17E-22 | 2.16E-20 |
| LRRN2 | -0.135791869 | 0.002012125 | 0.004057951 |
| LRRN3 | -0.331571006 | 1.11E-14 | 1.30E-13 |
| LRRN4CL | -0.158321309 | 0.000309846 | 0.000721651 |
| LRRN4 | -0.234884889 | 6.93E-08 | 2.92E-07 |
| LRRTM1 | -0.225014381 | 2.47E-07 | 9.53E-07 |
| LRRTM2 | -0.122867432 | 0.005236447 | 0.009803869 |
| LRRTM3 | 0.104153815 | 0.018063382 | 0.030383816 |
| LRSAM1 | -0.246353627 | 1.47E-08 | 6.87E-08 |
| LRTOMT | -0.144195867 | 0.001032423 | 0.002190201 |
| LRWD1 | 0.128959508 | 0.003371162 | 0.006521438 |
| LSAMP | -0.304310948 | 1.70E-12 | 1.43E-11 |
| LSG1 | 0.195172386 | 8.14E-06 | 2.47E-05 |
| LSM12 | 0.388970421 | 4.76E-20 | 1.24E-18 |
| LSM14A | 0.119213813 | 0.006759156 | 0.012392406 |
| LSM1 | 0.238668354 | 4.19E-08 | 1.83E-07 |
| LSM2 | 0.325273643 | 3.72E-14 | 4.03E-13 |
| LSM3 | 0.25079246 | 7.93E-09 | 3.85E-08 |
| LSM4 | 0.245242961 | 1.72E-08 | 7.93E-08 |
| LSM5 | 0.35575488 | 8.24E-17 | 1.33E-15 |
| LSM6 | 0.17730653 | 5.21E-05 | 0.000138563 |
| LSM7 | 0.099512696 | 0.02391928 | 0.03919162 |
| LSP1 | -0.281481246 | 7.81E-11 | 5.11E-10 |
| LSS | -0.260879317 | 1.85E-09 | 9.84E-09 |
| LST1 | -0.215720742 | 7.74E-07 | 2.76E-06 |
| LTA4H | -0.18074888 | 3.69E-05 | 0.000100751 |
| LTA | -0.184971382 | 2.40E-05 | 6.76E-05 |
| LTB4R2 | -0.250070198 | 8.77E-09 | 4.23E-08 |
| LTB4R | -0.21209558 | 1.19E-06 | 4.13E-06 |
| LTBP2 | -0.311397039 | 4.83E-13 | 4.42E-12 |
| LTBP3 | -0.328558119 | 1.99E-14 | 2.24E-13 |
| LTBP4 | -0.360767647 | 2.83E-17 | 4.93E-16 |
| LTBR | 0.274427698 | 2.38E-10 | 1.45E-09 |
| LTB | -0.251832727 | 6.84E-09 | 3.35E-08 |
| LTC4S | -0.419288317 | 2.41E-23 | 1.04E-21 |
| LTF | -0.170179897 | 0.000104046 | 0.000264153 |
| LTV1 | 0.280480574 | 9.16E-11 | 5.93E-10 |
| LUC7L3 | -0.294900504 | 8.58E-12 | 6.49E-11 |
| LUC7L | -0.276111257 | 1.83E-10 | 1.13E-09 |
| LUZP2 | -0.292560548 | 1.27E-11 | 9.37E-11 |
| LUZP6 | 0.299466461 | 3.94E-12 | 3.11E-11 |
| LY6E | -0.160411005 | 0.000257026 | 0.000608557 |
| LY6G5B | -0.236155408 | 5.86E-08 | 2.49E-07 |
| LY6G5C | -0.170221883 | 0.000103631 | 0.000263166 |
| LY6K | 0.158960083 | 0.000292712 | 0.000685177 |
| LY75 | -0.155288164 | 0.000404754 | 0.000921963 |
| LY86 | -0.302458546 | 2.35E-12 | 1.93E-11 |
| LY9 | -0.174295027 | 7.00E-05 | 0.0001825 |
| LYAR | 0.472299638 | 5.66E-30 | 6.25E-28 |
| LYG2 | 0.104831878 | 0.017322337 | 0.029211925 |
| LYL1 | -0.320428672 | 9.24E-14 | 9.40E-13 |
| LYNX1 | -0.313335227 | 3.40E-13 | 3.20E-12 |
| LYPD1 | 0.13538142 | 0.002076865 | 0.00417966 |
| LYPD2 | -0.149716266 | 0.000653026 | 0.001436097 |
| LYPD3 | 0.246693692 | 1.41E-08 | 6.57E-08 |
| LYPLA1 | 0.375269766 | 1.15E-18 | 2.47E-17 |
| LYPLA2 | -0.126043016 | 0.004172013 | 0.00794041 |
| LYRM4 | 0.251290861 | 7.39E-09 | 3.61E-08 |
| LYRM7 | -0.095763304 | 0.02978555 | 0.047873397 |
| LYSMD1 | 0.143196519 | 0.0011198 | 0.00236103 |
| LYSMD2 | -0.110488578 | 0.0121081 | 0.021099974 |
| LYSMD4 | -0.282016584 | 7.16E-11 | 4.71E-10 |
| LYST | -0.330810436 | 1.29E-14 | 1.49E-13 |
| LYZL2 | 0.18950599 | 1.50E-05 | 4.36E-05 |
| LYZ | -0.192505788 | 1.09E-05 | 3.23E-05 |
| LZIC | 0.248806554 | 1.05E-08 | 5.00E-08 |
| LZTFL1 | -0.187338121 | 1.88E-05 | 5.39E-05 |
| LZTR1 | -0.200385878 | 4.58E-06 | 1.45E-05 |
| LZTS2 | -0.121641214 | 0.005709028 | 0.010608166 |
| M6PR | 0.134644301 | 0.002197934 | 0.004401594 |
| MAB21L1 | -0.262869314 | 1.38E-09 | 7.48E-09 |
| MAB21L2 | -0.174738709 | 6.70E-05 | 0.000175227 |
| MACC1 | -0.265738838 | 8.99E-10 | 5.04E-09 |
| MACF1 | -0.382230007 | 2.32E-19 | 5.58E-18 |
| MACROD2 | -0.343223231 | 1.10E-15 | 1.52E-14 |
| MAD2L1BP | 0.209995991 | 1.53E-06 | 5.19E-06 |
| MAD2L1 | 0.578696263 | 2.26E-47 | 6.46E-44 |
| MAD2L2 | 0.160777526 | 0.000248678 | 0.00059047 |
| MADCAM1 | -0.102663785 | 0.019789715 | 0.033018098 |
| MADD | -0.333629725 | 7.44E-15 | 8.97E-14 |
| MAEA | 0.111105391 | 0.011633464 | 0.020358053 |
| MAEL | -0.101782771 | 0.020876615 | 0.034672355 |
| MAF1 | 0.149091998 | 0.000688286 | 0.001508824 |
| MAFA | 0.156701573 | 0.000357582 | 0.000822907 |
| MAFB | -0.107806803 | 0.014376399 | 0.024699627 |
| MAF | -0.1587492 | 0.000298268 | 0.000696959 |
| MAGEA11 | 0.164828443 | 0.000171831 | 0.000420952 |
| MAGEA12 | 0.197573375 | 6.26E-06 | 1.93E-05 |
| MAGEA1 | 0.239903759 | 3.55E-08 | 1.56E-07 |
| MAGEA2 | 0.228108727 | 1.67E-07 | 6.61E-07 |
| MAGEA3 | 0.25098437 | 7.71E-09 | 3.76E-08 |
| MAGEA4 | 0.099911753 | 0.023358152 | 0.03837323 |
| MAGEA6 | 0.243045925 | 2.32E-08 | 1.05E-07 |
| MAGEA8 | 0.111739204 | 0.011162966 | 0.019615417 |
| MAGEA9B | 0.107064646 | 0.015066774 | 0.02578379 |
| MAGEB18 | 0.11838153 | 0.007157214 | 0.013056346 |
| MAGEB1 | 0.1251675 | 0.004443989 | 0.00841877 |
| MAGEB2 | 0.213716227 | 9.84E-07 | 3.46E-06 |
| MAGEB6 | 0.2015401 | 4.03E-06 | 1.28E-05 |
| MAGEC1 | 0.181666957 | 3.37E-05 | 9.25E-05 |
| MAGEC2 | 0.190555046 | 1.34E-05 | 3.93E-05 |
| MAGEE1 | -0.302930668 | 2.16E-12 | 1.79E-11 |
| MAGEE2 | -0.323520016 | 5.18E-14 | 5.47E-13 |
| MAGEH1 | -0.116128348 | 0.008342163 | 0.015031385 |
| MAGI1 | -0.303014532 | 2.13E-12 | 1.77E-11 |
| MAGI2 | -0.305535795 | 1.37E-12 | 1.17E-11 |
| MAGI3 | -0.330606887 | 1.34E-14 | 1.55E-13 |
| MAGIX | -0.122066206 | 0.005541062 | 0.010327746 |
| MAGOHB | 0.413877244 | 9.90E-23 | 3.88E-21 |
| MAGOH | 0.244201869 | 1.98E-08 | 9.08E-08 |
| MAGT1 | 0.151765381 | 0.000548717 | 0.001221217 |
| MAG | -0.193413122 | 9.85E-06 | 2.95E-05 |
| MAK16 | 0.200848474 | 4.35E-06 | 1.38E-05 |
| MAK | -0.213998176 | 9.51E-07 | 3.35E-06 |
| MALAT1 | -0.33350911 | 7.62E-15 | 9.15E-14 |
| MALL | -0.30487344 | 1.54E-12 | 1.31E-11 |
| MAL | -0.331397803 | 1.15E-14 | 1.34E-13 |
| MAMDC2 | -0.406214348 | 7.00E-22 | 2.43E-20 |
| MAMDC4 | -0.261913425 | 1.59E-09 | 8.54E-09 |
| MAML1 | -0.207233956 | 2.11E-06 | 7.00E-06 |
| MAML2 | -0.301295208 | 2.87E-12 | 2.33E-11 |
| MAML3 | -0.157124339 | 0.000344502 | 0.000795047 |
| MAMSTR | 0.145853127 | 0.000901271 | 0.001930206 |
| MAN1C1 | -0.424200774 | 6.54E-24 | 3.09E-22 |
| MAN2A1 | 0.148761136 | 0.000707681 | 0.001547946 |
| MAN2A2 | -0.211624175 | 1.26E-06 | 4.35E-06 |
| MAN2B1 | -0.296609919 | 6.42E-12 | 4.94E-11 |
| MAN2B2 | -0.21294439 | 1.08E-06 | 3.76E-06 |
| MAN2C1 | -0.332151018 | 9.92E-15 | 1.17E-13 |
| MANBAL | 0.150615047 | 0.000605195 | 0.001337975 |
| MANBA | -0.179083524 | 4.36E-05 | 0.000117613 |
| MANEAL | -0.114111837 | 0.009547856 | 0.017019759 |
| MANF | 0.331011463 | 1.24E-14 | 1.44E-13 |
| MAOA | -0.196316891 | 7.19E-06 | 2.20E-05 |
| MAOB | -0.323496738 | 5.20E-14 | 5.49E-13 |
| MAP1A | -0.109142386 | 0.013203835 | 0.022844133 |
| MAP1LC3A | -0.276289745 | 1.78E-10 | 1.10E-09 |
| MAP1LC3B2 | 0.17828285 | 4.73E-05 | 0.000126674 |
| MAP1LC3C | -0.217383634 | 6.33E-07 | 2.30E-06 |
| MAP1S | -0.188017953 | 1.75E-05 | 5.05E-05 |
| MAP2K1 | 0.196668415 | 6.92E-06 | 2.12E-05 |
| MAP2K4 | 0.13209731 | 0.00266759 | 0.005260956 |
| MAP2K5 | -0.123322834 | 0.005070113 | 0.009516519 |
| MAP2K6 | 0.111199465 | 0.01156254 | 0.020249895 |
| MAP2K7 | -0.262075169 | 1.55E-09 | 8.37E-09 |
| MAP3K11 | -0.160281196 | 0.000260045 | 0.000614976 |
| MAP3K12 | -0.283955211 | 5.24E-11 | 3.51E-10 |
| MAP3K13 | -0.273560296 | 2.72E-10 | 1.64E-09 |
| MAP3K14 | -0.321345563 | 7.78E-14 | 8.02E-13 |
| MAP3K15 | -0.221320147 | 3.91E-07 | 1.46E-06 |
| MAP3K1 | -0.28606234 | 3.72E-11 | 2.55E-10 |
| MAP3K2 | 0.100499517 | 0.022552437 | 0.037171445 |
| MAP3K3 | -0.496085933 | 2.40E-33 | 4.89E-31 |
| MAP3K4 | -0.14427064 | 0.001026145 | 0.002179659 |
| MAP3K5 | -0.227716453 | 1.75E-07 | 6.94E-07 |
| MAP3K6 | -0.351891819 | 1.86E-16 | 2.84E-15 |
| MAP3K7 | 0.108692862 | 0.013588719 | 0.023455208 |
| MAP3K8 | -0.153247302 | 0.000483166 | 0.001085969 |
| MAP4K1 | -0.2637164 | 1.22E-09 | 6.66E-09 |
| MAP4K2 | -0.133374654 | 0.002421686 | 0.004811118 |
| MAP4K3 | 0.216383782 | 7.14E-07 | 2.56E-06 |
| MAP4K4 | 0.219120264 | 5.12E-07 | 1.89E-06 |
| MAP4K5 | 0.188630516 | 1.64E-05 | 4.75E-05 |
| MAP6D1 | 0.256326464 | 3.60E-09 | 1.83E-08 |
| MAP6 | -0.363468091 | 1.57E-17 | 2.84E-16 |
| MAP7D1 | -0.138626348 | 0.001613103 | 0.003303217 |
| MAP7D2 | -0.161609303 | 0.000230665 | 0.000551565 |
| MAP9 | -0.186370984 | 2.08E-05 | 5.92E-05 |
| MAPK10 | -0.466547673 | 3.39E-29 | 3.25E-27 |
| MAPK11 | -0.15925534 | 0.000285095 | 0.000669385 |
| MAPK12 | 0.135419071 | 0.002070848 | 0.00416839 |
| MAPK15 | -0.194363852 | 8.89E-06 | 2.68E-05 |
| MAPK1IP1L | 0.348827126 | 3.51E-16 | 5.19E-15 |
| MAPK3 | -0.130388184 | 0.00303221 | 0.005915265 |
| MAPK4 | -0.119276255 | 0.006730107 | 0.012344809 |
| MAPK6 | 0.406908795 | 5.87E-22 | 2.06E-20 |
| MAPK7 | -0.141832125 | 0.001250121 | 0.002615388 |
| MAPK8IP1 | -0.259381513 | 2.31E-09 | 1.21E-08 |
| MAPK8IP3 | -0.348138553 | 4.04E-16 | 5.93E-15 |
| MAPK8 | 0.309597393 | 6.67E-13 | 6.00E-12 |
| MAPKAP1 | 0.137613614 | 0.001746478 | 0.003555572 |
| MAPKAPK3 | -0.136706517 | 0.001874447 | 0.003795608 |
| MAPKAPK5 | 0.410499094 | 2.36E-22 | 8.65E-21 |
| MAPKBP1 | -0.358758424 | 4.35E-17 | 7.34E-16 |
| MAPRE1 | 0.374293504 | 1.43E-18 | 3.03E-17 |
| MAPRE2 | -0.139472111 | 0.001508952 | 0.003109695 |
| MAPRE3 | -0.355968546 | 7.88E-17 | 1.28E-15 |
| MAPT | -0.204748807 | 2.80E-06 | 9.13E-06 |
| 1-Mar | -0.135512194 | 0.002056034 | 0.004140239 |
| 2-Mar | -0.187736387 | 1.80E-05 | 5.19E-05 |
| 5-Mar | 0.37217807 | 2.30E-18 | 4.71E-17 |
| 7-Mar | 0.202398018 | 3.66E-06 | 1.17E-05 |
| 9-Mar | -0.207183827 | 2.12E-06 | 7.04E-06 |
| MARCKSL1 | 0.21935428 | 4.98E-07 | 1.84E-06 |
| MARCKS | 0.164968889 | 0.000169616 | 0.00041568 |
| MARCO | -0.215151672 | 8.28E-07 | 2.94E-06 |
| MARS2 | 0.29108187 | 1.63E-11 | 1.18E-10 |
| MARS | 0.378393928 | 5.62E-19 | 1.27E-17 |
| MARVELD2 | -0.163869268 | 0.000187695 | 0.000456183 |
| MAS1L | -0.323551567 | 5.15E-14 | 5.44E-13 |
| MASP1 | -0.258878232 | 2.48E-09 | 1.29E-08 |
| MASP2 | -0.391408746 | 2.66E-20 | 7.27E-19 |
| MAST1 | 0.130579222 | 0.002989315 | 0.005839566 |
| MAST3 | -0.384462793 | 1.38E-19 | 3.43E-18 |
| MAST4 | -0.285483935 | 4.09E-11 | 2.78E-10 |
| MASTL | 0.351268155 | 2.11E-16 | 3.20E-15 |
| MAT2A | -0.247169953 | 1.32E-08 | 6.18E-08 |
| MAT2B | -0.101139456 | 0.021702696 | 0.035904112 |
| MATK | -0.227169541 | 1.88E-07 | 7.40E-07 |
| MATN1 | -0.106787607 | 0.01533185 | 0.026172433 |
| MATN2 | -0.144533634 | 0.001004344 | 0.002135846 |
| MATN4 | -0.198789412 | 5.47E-06 | 1.71E-05 |
| MAVS | -0.134481836 | 0.002225471 | 0.004451834 |
| MAZ | 0.197079009 | 6.61E-06 | 2.03E-05 |
| MBD5 | -0.177829226 | 4.95E-05 | 0.000132198 |
| MBD6 | -0.209809503 | 1.56E-06 | 5.30E-06 |
| MBIP | -0.273091461 | 2.93E-10 | 1.76E-09 |
| MBL1P | -0.443898397 | 2.81E-26 | 1.91E-24 |
| MBLAC2 | -0.127505072 | 0.003751223 | 0.007195034 |
| MBNL1 | -0.222014838 | 3.59E-07 | 1.35E-06 |
| MBNL2 | -0.142067012 | 0.001226734 | 0.002568611 |
| MBNL3 | -0.141067806 | 0.00132909 | 0.002763534 |
| MBOAT1 | -0.189501069 | 1.50E-05 | 4.36E-05 |
| MBOAT4 | -0.127881346 | 0.003649344 | 0.007011061 |
| MBP | -0.117505425 | 0.007598777 | 0.013800141 |
| MBTD1 | -0.134437349 | 0.002233066 | 0.004464793 |
| MBTPS2 | 0.202686224 | 3.54E-06 | 1.14E-05 |
| MB | 0.134832974 | 0.002166346 | 0.004343557 |
| MC1R | 0.162162896 | 0.000219361 | 0.00052712 |
| MC2R | -0.15599144 | 0.000380602 | 0.000870619 |
| MC4R | -0.111145221 | 0.011603388 | 0.020310756 |
| MC5R | -0.260547346 | 1.94E-09 | 1.03E-08 |
| MCAM | 0.120515095 | 0.006176451 | 0.011400398 |
| MCART1 | 0.115302478 | 0.008818494 | 0.015802767 |
| MCART6 | -0.183903681 | 2.68E-05 | 7.49E-05 |
| MCCC1 | -0.258374427 | 2.67E-09 | 1.38E-08 |
| MCCC2 | 0.105440481 | 0.016679984 | 0.028239837 |
| MCC | -0.273682197 | 2.67E-10 | 1.61E-09 |
| MCF2L2 | 0.131327709 | 0.002826522 | 0.005545398 |
| MCF2L | -0.163617465 | 0.000192082 | 0.000466128 |
| MCFD2 | 0.231583108 | 1.07E-07 | 4.37E-07 |
| MCHR1 | 0.111360729 | 0.011441849 | 0.020066656 |
| MCHR2 | 0.147195422 | 0.000806523 | 0.001744295 |
| MCM10 | 0.515123994 | 3.03E-36 | 1.10E-33 |
| MCM2 | 0.322272523 | 6.55E-14 | 6.82E-13 |
| MCM3AP | -0.24689347 | 1.37E-08 | 6.40E-08 |
| MCM3 | 0.206183742 | 2.38E-06 | 7.84E-06 |
| MCM4 | 0.439796894 | 9.01E-26 | 5.70E-24 |
| MCM5 | 0.149404493 | 0.000670421 | 0.001472083 |
| MCM6 | 0.39020421 | 3.55E-20 | 9.55E-19 |
| MCM7 | 0.358924747 | 4.20E-17 | 7.12E-16 |
| MCM8 | 0.257141587 | 3.20E-09 | 1.63E-08 |
| MCM9 | -0.213366839 | 1.03E-06 | 3.60E-06 |
| MCOLN1 | -0.276606669 | 1.69E-10 | 1.05E-09 |
| MCOLN2 | -0.187516975 | 1.84E-05 | 5.30E-05 |
| MCOLN3 | -0.219372569 | 4.97E-07 | 1.84E-06 |
| MCRS1 | 0.249837693 | 9.07E-09 | 4.37E-08 |
| MCTP2 | -0.184363735 | 2.56E-05 | 7.17E-05 |
| MCTS1 | 0.176275576 | 5.77E-05 | 0.000152012 |
| MDFI | 0.207266157 | 2.10E-06 | 6.98E-06 |
| MDGA1 | -0.234584985 | 7.21E-08 | 3.03E-07 |
| MDGA2 | 0.178768184 | 4.50E-05 | 0.000121164 |
| MDH1B | -0.199682406 | 4.96E-06 | 1.55E-05 |
| MDH1 | 0.254083843 | 4.97E-09 | 2.48E-08 |
| MDH2 | 0.36256394 | 1.92E-17 | 3.41E-16 |
| MDM4 | -0.272336405 | 3.29E-10 | 1.96E-09 |
| MDN1 | -0.182604623 | 3.06E-05 | 8.46E-05 |
| MDS2 | -0.166149301 | 0.000152033 | 0.000375389 |
| ME1 | 0.238452886 | 4.32E-08 | 1.88E-07 |
| MEA1 | 0.153717837 | 0.000463926 | 0.001046375 |
| MEAF6 | -0.17563608 | 6.14E-05 | 0.000161255 |
| MECOM | -0.238222862 | 4.45E-08 | 1.93E-07 |
| MECP2 | -0.322097254 | 6.76E-14 | 7.03E-13 |
| MED10 | 0.120193812 | 0.006315965 | 0.011638565 |
| MED12 | -0.114186117 | 0.009500832 | 0.016938956 |
| MED15 | -0.185293049 | 2.32E-05 | 6.55E-05 |
| MED16 | -0.217969219 | 5.89E-07 | 2.15E-06 |
| MED17 | 0.174099426 | 7.14E-05 | 0.000185665 |
| MED19 | 0.203577402 | 3.20E-06 | 1.03E-05 |
| MED20 | 0.230330747 | 1.25E-07 | 5.09E-07 |
| MED21 | 0.161513375 | 0.000232678 | 0.00055598 |
| MED22 | -0.109201312 | 0.013154103 | 0.022777805 |
| MED23 | -0.151189568 | 0.000576345 | 0.001278578 |
| MED25 | -0.219641946 | 4.80E-07 | 1.78E-06 |
| MED26 | -0.337469631 | 3.49E-15 | 4.46E-14 |
| MED27 | 0.264332211 | 1.11E-09 | 6.13E-09 |
| MED28 | 0.213901684 | 9.62E-07 | 3.39E-06 |
| MED29 | -0.183126007 | 2.90E-05 | 8.06E-05 |
| MED30 | 0.181245694 | 3.51E-05 | 9.63E-05 |
| MED31 | 0.111127209 | 0.011616981 | 0.020330988 |
| MED6 | 0.373223542 | 1.82E-18 | 3.76E-17 |
| MED8 | 0.145531879 | 0.000925424 | 0.001978964 |
| MED9 | -0.134417928 | 0.00223639 | 0.004470543 |
| MEF2A | -0.34104932 | 1.71E-15 | 2.29E-14 |
| MEF2B | -0.117296083 | 0.007707826 | 0.013977865 |
| MEF2C | -0.365137738 | 1.09E-17 | 2.03E-16 |
| MEF2D | -0.110928663 | 0.011767744 | 0.020562283 |
| MEG3 | -0.174489199 | 6.87E-05 | 0.000179287 |
| MEGF11 | -0.379827605 | 4.04E-19 | 9.34E-18 |
| MEGF6 | -0.480015015 | 4.87E-31 | 6.38E-29 |
| MEGF8 | -0.110598695 | 0.01202213 | 0.020961125 |
| MEGF9 | -0.152315746 | 0.000523463 | 0.001169571 |
| MEI1 | -0.100600299 | 0.022416732 | 0.036969862 |
| MEIS1 | -0.311389575 | 4.83E-13 | 4.42E-12 |
| MEIS3P1 | -0.185983453 | 2.16E-05 | 6.14E-05 |
| MELK | 0.497891426 | 1.30E-33 | 2.78E-31 |
| MEMO1 | 0.382705677 | 2.08E-19 | 5.01E-18 |
| MEOX1 | -0.357819257 | 5.31E-17 | 8.88E-16 |
| MEOX2 | -0.242369953 | 2.55E-08 | 1.15E-07 |
| MEPE | 0.13279911 | 0.002529805 | 0.0050075 |
| MERTK | -0.287043935 | 3.17E-11 | 2.20E-10 |
| MESDC2 | 0.242202322 | 2.60E-08 | 1.17E-07 |
| MESP1 | -0.1452994 | 0.000943274 | 0.002014118 |
| MESP2 | -0.137057247 | 0.001823983 | 0.003701671 |
| MESTIT1 | -0.140341875 | 0.001408318 | 0.002917042 |
| MEST | 0.371165219 | 2.88E-18 | 5.82E-17 |
| METAP1 | 0.341930982 | 1.43E-15 | 1.93E-14 |
| METAP2 | 0.270604489 | 4.29E-10 | 2.52E-09 |
| METRNL | -0.108388733 | 0.013854687 | 0.023877176 |
| METRN | -0.106895316 | 0.01522831 | 0.026017405 |
| METT10D | -0.114877164 | 0.009073129 | 0.016222712 |
| METT5D1 | 0.098322108 | 0.025663207 | 0.041798906 |
| METTL10 | 0.266387816 | 8.15E-10 | 4.59E-09 |
| METTL11A | 0.305303727 | 1.43E-12 | 1.22E-11 |
| METTL12 | 0.138615187 | 0.001614521 | 0.003305443 |
| METTL13 | 0.110220276 | 0.012319843 | 0.021442791 |
| METTL1 | 0.2594669 | 2.28E-09 | 1.19E-08 |
| METTL2A | 0.411978621 | 1.61E-22 | 6.08E-21 |
| METTL2B | 0.341996726 | 1.41E-15 | 1.91E-14 |
| METTL4 | 0.175975589 | 5.94E-05 | 0.000156399 |
| METTL5 | 0.354470617 | 1.08E-16 | 1.71E-15 |
| METTL6 | 0.221094429 | 4.02E-07 | 1.50E-06 |
| METTL7A | -0.414962436 | 7.47E-23 | 2.98E-21 |
| METTL8 | 0.301931696 | 2.57E-12 | 2.10E-11 |
| METTL9 | 0.287188262 | 3.10E-11 | 2.15E-10 |
| MEX3A | 0.179391812 | 4.23E-05 | 0.000114318 |
| MEX3D | 0.124103148 | 0.004796145 | 0.009043047 |
| MFAP1 | 0.137216772 | 0.001801444 | 0.003659646 |
| MFAP4 | -0.422699628 | 9.77E-24 | 4.46E-22 |
| MFAP5 | 0.19521053 | 8.11E-06 | 2.46E-05 |
| MFF | 0.264619112 | 1.06E-09 | 5.89E-09 |
| MFGE8 | -0.128966924 | 0.003369317 | 0.006518819 |
| MFI2 | 0.127347565 | 0.00379463 | 0.007272707 |
| MFN1 | 0.209844272 | 1.55E-06 | 5.28E-06 |
| MFN2 | -0.182689419 | 3.03E-05 | 8.40E-05 |
| MFNG | -0.263949261 | 1.17E-09 | 6.45E-09 |
| MFRP | -0.142118426 | 0.001221669 | 0.002558542 |
| MFSD10 | -0.143254545 | 0.001114546 | 0.00235157 |
| MFSD1 | -0.131826935 | 0.002722473 | 0.005358621 |
| MFSD2A | -0.323552095 | 5.15E-14 | 5.44E-13 |
| MFSD2B | 0.477960708 | 9.42E-31 | 1.17E-28 |
| MFSD3 | 0.117792853 | 0.007451298 | 0.013548307 |
| MFSD4 | -0.37606661 | 9.56E-19 | 2.08E-17 |
| MFSD5 | 0.189783068 | 1.45E-05 | 4.25E-05 |
| MFSD6L | -0.128259925 | 0.003549378 | 0.006836093 |
| MFSD6 | -0.150166459 | 0.000628646 | 0.001385683 |
| MFSD7 | -0.262829816 | 1.39E-09 | 7.52E-09 |
| MFSD9 | 0.2061836 | 2.38E-06 | 7.84E-06 |
| MGAM | -0.196034495 | 7.41E-06 | 2.26E-05 |
| MGAT1 | -0.180276307 | 3.87E-05 | 0.00010529 |
| MGAT2 | 0.458510222 | 3.90E-28 | 3.22E-26 |
| MGAT3 | -0.32037621 | 9.33E-14 | 9.48E-13 |
| MGC14436 | 0.20745161 | 2.05E-06 | 6.84E-06 |
| MGC16275 | -0.147398769 | 0.000793001 | 0.00171728 |
| MGC16384 | -0.199809525 | 4.89E-06 | 1.53E-05 |
| MGC16703 | -0.26366491 | 1.23E-09 | 6.71E-09 |
| MGC21881 | -0.182922467 | 2.96E-05 | 8.22E-05 |
| MGC23284 | -0.229349495 | 1.42E-07 | 5.71E-07 |
| MGC27382 | -0.251678538 | 6.99E-09 | 3.42E-08 |
| MGC2752 | -0.12741018 | 0.00377732 | 0.00724092 |
| MGC34034 | 0.171746773 | 8.96E-05 | 0.000229507 |
| MGC3771 | -0.272111463 | 3.41E-10 | 2.03E-09 |
| MGC42105 | -0.159097564 | 0.000289142 | 0.000677773 |
| MGC4473 | 0.108331265 | 0.013905456 | 0.023954344 |
| MGC45800 | 0.100034559 | 0.023187779 | 0.03813059 |
| MGC72080 | 0.269885962 | 4.79E-10 | 2.79E-09 |
| MGC87042 | 0.342960488 | 1.16E-15 | 1.59E-14 |
| MGEA5 | -0.217012839 | 6.62E-07 | 2.39E-06 |
| MGLL | -0.346604504 | 5.54E-16 | 7.97E-15 |
| MGP | -0.374294338 | 1.43E-18 | 3.03E-17 |
| MGRN1 | -0.413815959 | 1.01E-22 | 3.93E-21 |
| MGST1 | 0.142640532 | 0.001171315 | 0.002461596 |
| MGST2 | -0.09852953 | 0.02535172 | 0.041342105 |
| MGST3 | 0.213441807 | 1.02E-06 | 3.57E-06 |
| MIA3 | -0.113186656 | 0.010151135 | 0.018000434 |
| MIA | -0.149151083 | 0.000684875 | 0.001502335 |
| MIB1 | 0.195703639 | 7.69E-06 | 2.34E-05 |
| MIB2 | -0.294712116 | 8.86E-12 | 6.68E-11 |
| MICAL1 | -0.370838233 | 3.10E-18 | 6.25E-17 |
| MICAL2 | -0.157267212 | 0.000340183 | 0.000785715 |
| MICALCL | -0.285623047 | 4.00E-11 | 2.73E-10 |
| MICALL2 | -0.260318067 | 2.01E-09 | 1.06E-08 |
| MICA | -0.116357726 | 0.008214004 | 0.014820502 |
| MID1IP1 | -0.219750001 | 4.74E-07 | 1.76E-06 |
| MID2 | -0.263041016 | 1.35E-09 | 7.30E-09 |
| MIDN | -0.10111161 | 0.021739085 | 0.035955385 |
| MIER2 | -0.150344999 | 0.000619213 | 0.001366397 |
| MIER3 | 0.106777939 | 0.015341174 | 0.026186113 |
| MIF4GD | -0.101644874 | 0.02105135 | 0.034936432 |
| MIF | 0.26370846 | 1.22E-09 | 6.67E-09 |
| MIIP | -0.099295921 | 0.024228952 | 0.039656756 |
| MINA | 0.290137956 | 1.90E-11 | 1.37E-10 |
| MINK1 | -0.192720767 | 1.06E-05 | 3.17E-05 |
| MINPP1 | 0.290986467 | 1.65E-11 | 1.20E-10 |
| MIPOL1 | 0.223813443 | 2.87E-07 | 1.10E-06 |
| MIP | 0.104398423 | 0.017792922 | 0.029961661 |
| MIR155HG | -0.131307583 | 0.002830791 | 0.00555214 |
| MIR17HG | -0.139195852 | 0.001542272 | 0.003170191 |
| MIS12 | 0.158238414 | 0.000312137 | 0.000726395 |
| MITD1 | 0.154374221 | 0.000438276 | 0.000993677 |
| MITF | -0.182358943 | 3.14E-05 | 8.66E-05 |
| MKI67IP | 0.458864005 | 3.51E-28 | 2.93E-26 |
| MKI67 | 0.416673791 | 4.79E-23 | 1.96E-21 |
| MKKS | 0.215388498 | 8.05E-07 | 2.87E-06 |
| MKL2 | -0.180880877 | 3.64E-05 | 9.96E-05 |
| MKNK1 | -0.285119098 | 4.34E-11 | 2.94E-10 |
| MKNK2 | -0.187183123 | 1.91E-05 | 5.47E-05 |
| MKRN1 | -0.13403511 | 0.002302822 | 0.004592777 |
| MKRN3 | 0.129674457 | 0.003197462 | 0.006212177 |
| MKS1 | -0.130929031 | 0.0029122 | 0.005700064 |
| MLANA | -0.107917149 | 0.014276164 | 0.024546406 |
| MLC1 | -0.159328865 | 0.000283227 | 0.000665391 |
| MLF1IP | 0.425042911 | 5.22E-24 | 2.50E-22 |
| MLF2 | 0.27763948 | 1.44E-10 | 9.04E-10 |
| MLKL | 0.109105848 | 0.013234757 | 0.022893667 |
| MLL2 | -0.151156853 | 0.000577953 | 0.001281684 |
| MLL3 | -0.217588397 | 6.17E-07 | 2.24E-06 |
| MLL4 | -0.139683474 | 0.001483907 | 0.003060921 |
| MLL5 | -0.230499031 | 1.23E-07 | 4.98E-07 |
| MLLT11 | 0.298739093 | 4.46E-12 | 3.50E-11 |
| MLLT1 | -0.308151703 | 8.63E-13 | 7.62E-12 |
| MLLT3 | -0.131125635 | 0.002869658 | 0.005619547 |
| MLLT4 | -0.329314225 | 1.72E-14 | 1.96E-13 |
| MLLT6 | -0.340342755 | 1.97E-15 | 2.61E-14 |
| MLL | -0.207456309 | 2.05E-06 | 6.84E-06 |
| MLPH | -0.252071198 | 6.61E-09 | 3.24E-08 |
| MLXIPL | -0.236927827 | 5.29E-08 | 2.27E-07 |
| MLX | 0.094991487 | 0.031135432 | 0.049782819 |
| MLYCD | -0.204656457 | 2.83E-06 | 9.22E-06 |
| MMACHC | 0.16597645 | 0.000154497 | 0.000380962 |
| MMADHC | 0.354923344 | 9.82E-17 | 1.56E-15 |
| MMD | 0.24918451 | 9.94E-09 | 4.77E-08 |
| MMEL1 | -0.169159509 | 0.000114625 | 0.000288774 |
| MMP10 | 0.111835303 | 0.01109312 | 0.019503584 |
| MMP12 | 0.261816695 | 1.61E-09 | 8.65E-09 |
| MMP14 | 0.167281978 | 0.000136783 | 0.000340263 |
| MMP15 | -0.174038207 | 7.18E-05 | 0.000186645 |
| MMP19 | -0.275308952 | 2.07E-10 | 1.27E-09 |
| MMP1 | 0.235222177 | 6.63E-08 | 2.80E-07 |
| MMP21 | -0.244356249 | 1.94E-08 | 8.90E-08 |
| MMP23B | -0.261481466 | 1.69E-09 | 9.07E-09 |
| MMP24 | -0.299213717 | 4.11E-12 | 3.25E-11 |
| MMP25 | -0.103992484 | 0.018243723 | 0.03065878 |
| MMP28 | -0.295444749 | 7.82E-12 | 5.96E-11 |
| MMP2 | -0.110031389 | 0.012470867 | 0.021673567 |
| MMP7 | -0.118514178 | 0.007092398 | 0.012947562 |
| MMRN1 | -0.248345519 | 1.12E-08 | 5.30E-08 |
| MMRN2 | -0.21625737 | 7.25E-07 | 2.60E-06 |
| MNAT1 | 0.321487038 | 7.58E-14 | 7.83E-13 |
| MND1 | 0.43908695 | 1.10E-25 | 6.88E-24 |
| MNDA | -0.217909752 | 5.94E-07 | 2.17E-06 |
| MOAP1 | -0.268931623 | 5.55E-10 | 3.20E-09 |
| MOB2 | -0.235129959 | 6.71E-08 | 2.83E-07 |
| MOBKL1B | 0.282657462 | 6.46E-11 | 4.27E-10 |
| MOBKL2A | -0.113290278 | 0.010081924 | 0.017887226 |
| MOBKL2B | -0.159838744 | 0.000270586 | 0.000637866 |
| MOBKL2C | -0.375661862 | 1.05E-18 | 2.27E-17 |
| MOBKL3 | 0.31268665 | 3.82E-13 | 3.56E-12 |
| MOBP | -0.098563665 | 0.025300773 | 0.041265756 |
| MOCOS | 0.264542232 | 1.08E-09 | 5.95E-09 |
| MOCS1 | -0.428255044 | 2.19E-24 | 1.11E-22 |
| MOCS3 | 0.205012137 | 2.72E-06 | 8.87E-06 |
| MOGAT2 | -0.163197006 | 0.000199621 | 0.000483055 |
| MOGAT3 | 0.104921404 | 0.01722651 | 0.029071553 |
| MON2 | -0.096933925 | 0.027834033 | 0.045000943 |
| MORC3 | -0.23346162 | 8.35E-08 | 3.48E-07 |
| MORC4 | 0.109158871 | 0.013189906 | 0.02282596 |
| MORF4L1 | 0.134301887 | 0.00225634 | 0.004505916 |
| MORF4L2 | 0.340890976 | 1.77E-15 | 2.36E-14 |
| MORF4 | 0.097744284 | 0.026548392 | 0.043117508 |
| MORN1 | -0.264091185 | 1.15E-09 | 6.33E-09 |
| MORN3 | -0.229801212 | 1.34E-07 | 5.41E-07 |
| MORN4 | -0.190748898 | 1.31E-05 | 3.86E-05 |
| MORN5 | -0.194827486 | 8.45E-06 | 2.56E-05 |
| MOSC2 | -0.291097903 | 1.62E-11 | 1.18E-10 |
| MOSPD1 | 0.151426588 | 0.00056482 | 0.001254402 |
| MOXD1 | -0.211262339 | 1.32E-06 | 4.53E-06 |
| MPDU1 | 0.161639184 | 0.000230041 | 0.000550337 |
| MPDZ | -0.185134064 | 2.36E-05 | 6.65E-05 |
| MPEG1 | -0.231993323 | 1.01E-07 | 4.16E-07 |
| MPHOSPH10 | 0.201806772 | 3.91E-06 | 1.25E-05 |
| MPHOSPH6 | 0.277262075 | 1.53E-10 | 9.56E-10 |
| MPHOSPH8 | -0.232441347 | 9.54E-08 | 3.94E-07 |
| MPHOSPH9 | 0.276239626 | 1.79E-10 | 1.11E-09 |
| MPL | -0.294484202 | 9.20E-12 | 6.93E-11 |
| MPND | -0.280253415 | 9.50E-11 | 6.14E-10 |
| MPP3 | -0.121010041 | 0.005966936 | 0.011048367 |
| MPP5 | 0.144629453 | 0.000996507 | 0.002119858 |
| MPP6 | 0.268658547 | 5.78E-10 | 3.33E-09 |
| MPPE1 | -0.130564183 | 0.002992672 | 0.005844494 |
| MPPED1 | -0.142695813 | 0.001166096 | 0.002451145 |
| MPPED2 | -0.161299356 | 0.00023723 | 0.000565503 |
| MPRIP | -0.295170568 | 8.20E-12 | 6.22E-11 |
| MPV17L | -0.104940933 | 0.017205669 | 0.029043738 |
| MPZL1 | 0.097430914 | 0.027039361 | 0.043840261 |
| MPZL2 | -0.226418751 | 2.07E-07 | 8.10E-07 |
| MR1 | -0.212824223 | 1.09E-06 | 3.81E-06 |
| MRAS | -0.19107528 | 1.27E-05 | 3.73E-05 |
| MRC1 | -0.209803995 | 1.56E-06 | 5.30E-06 |
| MRC2 | -0.241551314 | 2.84E-08 | 1.27E-07 |
| MRE11A | 0.158739939 | 0.000298515 | 0.00069729 |
| MRGPRE | -0.146605394 | 0.000846975 | 0.001824282 |
| MRGPRX2 | -0.166238187 | 0.000150781 | 0.000372674 |
| MRGPRX3 | 0.111037229 | 0.011685093 | 0.020432302 |
| MRGPRX4 | 0.14382627 | 0.001063978 | 0.002251407 |
| MRI1 | -0.187262129 | 1.89E-05 | 5.43E-05 |
| MRP63 | 0.130294425 | 0.003053466 | 0.005954407 |
| MRPL10 | 0.221958253 | 3.61E-07 | 1.36E-06 |
| MRPL11 | 0.348046515 | 4.12E-16 | 6.03E-15 |
| MRPL12 | 0.366811903 | 7.58E-18 | 1.44E-16 |
| MRPL13 | 0.446202684 | 1.45E-26 | 1.01E-24 |
| MRPL14 | 0.103246055 | 0.019098712 | 0.031958565 |
| MRPL15 | 0.520535234 | 4.21E-37 | 1.79E-34 |
| MRPL16 | 0.235912166 | 6.05E-08 | 2.57E-07 |
| MRPL17 | 0.327423867 | 2.47E-14 | 2.74E-13 |
| MRPL18 | 0.175770027 | 6.06E-05 | 0.000159359 |
| MRPL19 | 0.432940814 | 6.09E-25 | 3.38E-23 |
| MRPL1 | 0.326736647 | 2.81E-14 | 3.11E-13 |
| MRPL21 | 0.373774414 | 1.61E-18 | 3.37E-17 |
| MRPL22 | 0.238303279 | 4.40E-08 | 1.91E-07 |
| MRPL24 | 0.174934928 | 6.58E-05 | 0.000172126 |
| MRPL27 | 0.216727304 | 6.85E-07 | 2.47E-06 |
| MRPL28 | 0.142420843 | 0.001192265 | 0.002501941 |
| MRPL2 | 0.200907407 | 4.32E-06 | 1.37E-05 |
| MRPL30 | 0.392856298 | 1.88E-20 | 5.22E-19 |
| MRPL32 | 0.238731717 | 4.16E-08 | 1.81E-07 |
| MRPL33 | 0.180331442 | 3.85E-05 | 0.000104793 |
| MRPL34 | 0.099708205 | 0.023642928 | 0.038783327 |
| MRPL35 | 0.37185623 | 2.47E-18 | 5.04E-17 |
| MRPL36 | 0.229760446 | 1.35E-07 | 5.44E-07 |
| MRPL37 | 0.348318397 | 3.90E-16 | 5.73E-15 |
| MRPL39 | 0.215746806 | 7.71E-07 | 2.75E-06 |
| MRPL3 | 0.471795963 | 6.63E-30 | 7.13E-28 |
| MRPL42P5 | -0.179185237 | 4.32E-05 | 0.000116517 |
| MRPL42 | 0.534579216 | 2.11E-39 | 1.57E-36 |
| MRPL43 | 0.145916837 | 0.000896551 | 0.001921333 |
| MRPL44 | 0.361884802 | 2.22E-17 | 3.91E-16 |
| MRPL45 | 0.340441571 | 1.93E-15 | 2.57E-14 |
| MRPL46 | 0.188072555 | 1.74E-05 | 5.02E-05 |
| MRPL47 | 0.350953516 | 2.26E-16 | 3.40E-15 |
| MRPL48 | 0.323684282 | 5.02E-14 | 5.32E-13 |
| MRPL49 | 0.147325186 | 0.000797869 | 0.001726701 |
| MRPL50 | 0.288470741 | 2.51E-11 | 1.76E-10 |
| MRPL51 | 0.417605014 | 3.75E-23 | 1.58E-21 |
| MRPL52 | 0.363534035 | 1.55E-17 | 2.80E-16 |
| MRPL53 | 0.131615485 | 0.00276611 | 0.005434344 |
| MRPL9 | 0.323461282 | 5.23E-14 | 5.52E-13 |
| MRPS10 | 0.410027294 | 2.66E-22 | 9.71E-21 |
| MRPS11 | 0.282543568 | 6.58E-11 | 4.35E-10 |
| MRPS12 | 0.332812655 | 8.73E-15 | 1.04E-13 |
| MRPS14 | 0.231069412 | 1.14E-07 | 4.65E-07 |
| MRPS15 | 0.282959495 | 6.16E-11 | 4.08E-10 |
| MRPS16 | 0.412941918 | 1.26E-22 | 4.84E-21 |
| MRPS17 | 0.396183807 | 8.40E-21 | 2.47E-19 |
| MRPS18A | 0.182372311 | 3.13E-05 | 8.65E-05 |
| MRPS18B | 0.135049507 | 0.002130605 | 0.004279194 |
| MRPS18C | 0.217544233 | 6.21E-07 | 2.26E-06 |
| MRPS22 | 0.27482701 | 2.24E-10 | 1.37E-09 |
| MRPS23 | 0.288381169 | 2.55E-11 | 1.78E-10 |
| MRPS24 | 0.304505998 | 1.64E-12 | 1.39E-11 |
| MRPS25 | -0.22251601 | 3.37E-07 | 1.28E-06 |
| MRPS27 | 0.136407623 | 0.001918459 | 0.003877917 |
| MRPS28 | 0.364533203 | 1.25E-17 | 2.29E-16 |
| MRPS2 | 0.273553532 | 2.73E-10 | 1.64E-09 |
| MRPS30 | 0.352878327 | 1.51E-16 | 2.34E-15 |
| MRPS33 | 0.261964667 | 1.58E-09 | 8.49E-09 |
| MRPS34 | 0.110613527 | 0.012010592 | 0.020942835 |
| MRPS35 | 0.493256399 | 6.23E-33 | 1.15E-30 |
| MRPS5 | 0.264938311 | 1.01E-09 | 5.63E-09 |
| MRPS7 | 0.238673651 | 4.19E-08 | 1.82E-07 |
| MRPS9 | 0.193228006 | 1.01E-05 | 3.01E-05 |
| MRRF | 0.107710315 | 0.014464553 | 0.024831871 |
| MRS2 | 0.164503943 | 0.000177051 | 0.000432709 |
| MRTO4 | 0.380103359 | 3.79E-19 | 8.82E-18 |
| MRVI1 | -0.210592281 | 1.42E-06 | 4.87E-06 |
| MS4A14 | -0.28432278 | 4.94E-11 | 3.32E-10 |
| MS4A15 | -0.363116348 | 1.70E-17 | 3.06E-16 |
| MS4A1 | -0.212963811 | 1.08E-06 | 3.75E-06 |
| MS4A2 | -0.394686902 | 1.21E-20 | 3.45E-19 |
| MS4A5 | 0.11130207 | 0.011485621 | 0.020134589 |
| MS4A6A | -0.141023844 | 0.001333769 | 0.002771533 |
| MS4A7 | -0.242819546 | 2.39E-08 | 1.08E-07 |
| MS4A8B | -0.234245578 | 7.53E-08 | 3.16E-07 |
| MSH2 | 0.30428759 | 1.71E-12 | 1.44E-11 |
| MSH6 | 0.27762804 | 1.44E-10 | 9.05E-10 |
| MSI1 | 0.106329786 | 0.015778878 | 0.026857551 |
| MSI2 | 0.100518677 | 0.022526584 | 0.037131895 |
| MSL2 | -0.188864455 | 1.60E-05 | 4.65E-05 |
| MSL3L2 | 0.11352265 | 0.00992824 | 0.017647458 |
| MSMB | 0.112689913 | 0.01048881 | 0.018535083 |
| MSN | -0.177310226 | 5.21E-05 | 0.00013853 |
| MSRA | -0.218452029 | 5.56E-07 | 2.04E-06 |
| MSRB3 | -0.159826787 | 0.000270876 | 0.000638475 |
| MST1P2 | -0.33335943 | 7.84E-15 | 9.41E-14 |
| MST1P9 | -0.312623606 | 3.87E-13 | 3.60E-12 |
| MST1R | -0.141740713 | 0.001259332 | 0.002633282 |
| MST1 | -0.197530768 | 6.29E-06 | 1.94E-05 |
| MST4 | 0.120557697 | 0.006158161 | 0.011368738 |
| MSTN | -0.259079715 | 2.41E-09 | 1.26E-08 |
| MSTO2P | -0.217841558 | 5.99E-07 | 2.18E-06 |
| MSX1 | 0.117375092 | 0.007666506 | 0.013911767 |
| MSX2P1 | -0.166272493 | 0.000150301 | 0.000371624 |
| MT1B | 0.134012499 | 0.002306802 | 0.004598879 |
| MT1G | 0.169219656 | 0.000113975 | 0.000287243 |
| MT1H | 0.262459379 | 1.47E-09 | 7.92E-09 |
| MT1IP | -0.113254088 | 0.010106048 | 0.017926844 |
| MT1X | 0.178622561 | 4.57E-05 | 0.000122805 |
| MT2A | 0.149368648 | 0.000672448 | 0.001475885 |
| MTA2 | 0.244107871 | 2.01E-08 | 9.19E-08 |
| MTA3 | 0.185703946 | 2.23E-05 | 6.30E-05 |
| MTAP | 0.10135284 | 0.021425599 | 0.035495639 |
| MTBP | 0.364993117 | 1.13E-17 | 2.09E-16 |
| MTCH2 | 0.415461747 | 6.56E-23 | 2.65E-21 |
| MTCP1 | -0.139190659 | 0.001542905 | 0.003171165 |
| MTDH | 0.378975826 | 4.92E-19 | 1.12E-17 |
| MTERFD1 | 0.369735014 | 3.97E-18 | 7.88E-17 |
| MTERFD3 | -0.104986994 | 0.017156598 | 0.02897314 |
| MTERF | 0.283537891 | 5.61E-11 | 3.74E-10 |
| MTF1 | -0.152930238 | 0.000496546 | 0.001113163 |
| MTFR1 | 0.481350661 | 3.16E-31 | 4.40E-29 |
| MTHFD1L | 0.281601986 | 7.66E-11 | 5.01E-10 |
| MTHFD1 | 0.395023348 | 1.11E-20 | 3.20E-19 |
| MTHFD2 | 0.549524444 | 5.72E-42 | 7.14E-39 |
| MTHFR | -0.2349561 | 6.86E-08 | 2.90E-07 |
| MTHFSD | -0.158854958 | 0.00029547 | 0.000690904 |
| MTHFS | 0.161436252 | 0.000234309 | 0.000559475 |
| MTIF2 | 0.334480759 | 6.30E-15 | 7.72E-14 |
| MTL5 | 0.387909042 | 6.13E-20 | 1.58E-18 |
| MTM1 | -0.22177314 | 3.69E-07 | 1.39E-06 |
| MTMR10 | -0.321281985 | 7.88E-14 | 8.10E-13 |
| MTMR11 | 0.131850149 | 0.002717721 | 0.005350848 |
| MTMR12 | -0.156539009 | 0.000362734 | 0.000833372 |
| MTMR14 | -0.185809845 | 2.20E-05 | 6.24E-05 |
| MTMR15 | -0.117665473 | 0.007516337 | 0.013657868 |
| MTMR1 | -0.146147505 | 0.000879652 | 0.001888559 |
| MTMR2 | 0.351005221 | 2.23E-16 | 3.37E-15 |
| MTMR3 | -0.344066838 | 9.30E-16 | 1.29E-14 |
| MTMR7 | 0.096325216 | 0.028834627 | 0.04645708 |
| MTMR8 | -0.229023602 | 1.48E-07 | 5.93E-07 |
| MTMR9L | -0.263211133 | 1.31E-09 | 7.13E-09 |
| MTP18 | 0.300289294 | 3.42E-12 | 2.73E-11 |
| MTPAP | 0.349151193 | 3.28E-16 | 4.88E-15 |
| MTRF1L | 0.259963648 | 2.12E-09 | 1.11E-08 |
| MTRR | -0.145668915 | 0.000915049 | 0.001958034 |
| MTR | -0.309909305 | 6.30E-13 | 5.70E-12 |
| MTSS1L | -0.279466203 | 1.08E-10 | 6.89E-10 |
| MTSS1 | -0.206608288 | 2.26E-06 | 7.49E-06 |
| MTUS1 | -0.210193571 | 1.49E-06 | 5.08E-06 |
| MTUS2 | -0.099055356 | 0.024576662 | 0.040183094 |
| MTVR2 | -0.228232247 | 1.64E-07 | 6.52E-07 |
| MTX1 | 0.248947249 | 1.03E-08 | 4.91E-08 |
| MTX2 | 0.146917884 | 0.000825323 | 0.001782062 |
| MTX3 | -0.235509645 | 6.38E-08 | 2.70E-07 |
| MUC13 | 0.21297646 | 1.07E-06 | 3.75E-06 |
| MUC15 | -0.280391626 | 9.29E-11 | 6.01E-10 |
| MUC1 | -0.281050904 | 8.36E-11 | 5.45E-10 |
| MUC20 | -0.180817081 | 3.67E-05 | 0.000100129 |
| MUC21 | -0.241933825 | 2.70E-08 | 1.21E-07 |
| MUC2 | 0.139226888 | 0.001538495 | 0.003163747 |
| MUCL1 | 0.160269147 | 0.000260327 | 0.000615497 |
| MUDENG | 0.293466239 | 1.09E-11 | 8.12E-11 |
| MUM1 | -0.302925412 | 2.16E-12 | 1.79E-11 |
| MURC | 0.200517878 | 4.52E-06 | 1.43E-05 |
| MUSK | -0.363964672 | 1.41E-17 | 2.57E-16 |
| MUSTN1 | -0.434328075 | 4.15E-25 | 2.37E-23 |
| MUTED | 0.187891334 | 1.77E-05 | 5.11E-05 |
| MUTYH | -0.109785773 | 0.012669693 | 0.021990433 |
| MVP | -0.310380649 | 5.79E-13 | 5.27E-12 |
| MX1 | -0.190102748 | 1.40E-05 | 4.12E-05 |
| MX2 | -0.127776134 | 0.003677575 | 0.007061905 |
| MXD1 | 0.107565606 | 0.014597652 | 0.025051762 |
| MXD4 | -0.270830275 | 4.15E-10 | 2.44E-09 |
| MXRA5 | 0.103924671 | 0.018319994 | 0.030774019 |
| MYADM | -0.098230517 | 0.025801797 | 0.042010942 |
| MYBL1 | 0.212620519 | 1.12E-06 | 3.89E-06 |
| MYBL2 | 0.448704841 | 7.04E-27 | 5.06E-25 |
| MYBPC2 | -0.263982721 | 1.17E-09 | 6.43E-09 |
| MYBPC3 | -0.226345227 | 2.08E-07 | 8.16E-07 |
| MYBPHL | -0.361384405 | 2.47E-17 | 4.34E-16 |
| MYBPH | -0.161979654 | 0.000223044 | 0.000534813 |
| MYCBP2 | -0.232317688 | 9.69E-08 | 4.00E-07 |
| MYCBPAP | -0.277335134 | 1.51E-10 | 9.46E-10 |
| MYCBP | 0.166572528 | 0.000146157 | 0.000362221 |
| MYCNOS | 0.152169468 | 0.000530068 | 0.001183401 |
| MYCT1 | -0.154654254 | 0.00042774 | 0.000971663 |
| MYC | 0.217652996 | 6.12E-07 | 2.23E-06 |
| MYEF2 | -0.177778575 | 4.97E-05 | 0.000132829 |
| MYEOV2 | 0.165790146 | 0.000157193 | 0.000387277 |
| MYEOV | 0.378277912 | 5.77E-19 | 1.30E-17 |
| MYH10 | -0.248550732 | 1.09E-08 | 5.17E-08 |
| MYH11 | -0.348152655 | 4.03E-16 | 5.92E-15 |
| MYH13 | 0.108702087 | 0.013580722 | 0.02344343 |
| MYH14 | -0.266315528 | 8.24E-10 | 4.64E-09 |
| MYH16 | 0.314068395 | 2.98E-13 | 2.82E-12 |
| MYH1 | -0.302961314 | 2.15E-12 | 1.78E-11 |
| MYH2 | -0.270194627 | 4.57E-10 | 2.67E-09 |
| MYH3 | -0.291653915 | 1.48E-11 | 1.08E-10 |
| MYH4 | -0.099848778 | 0.02344594 | 0.038498106 |
| MYH7B | -0.309944458 | 6.26E-13 | 5.67E-12 |
| MYH9 | -0.121484742 | 0.005772013 | 0.010712256 |
| MYL3 | -0.245163504 | 1.74E-08 | 8.01E-08 |
| MYL5 | -0.302666128 | 2.26E-12 | 1.87E-11 |
| MYL6B | 0.33854884 | 2.82E-15 | 3.66E-14 |
| MYL6 | 0.169691133 | 0.000108993 | 0.000275877 |
| MYL9 | -0.176987977 | 5.38E-05 | 0.000142524 |
| MYLIP | -0.291465214 | 1.53E-11 | 1.11E-10 |
| MYLK2 | 0.164043072 | 0.000184722 | 0.000449778 |
| MYLK3 | -0.105882385 | 0.016226735 | 0.027542457 |
| MYLK4 | -0.274884601 | 2.22E-10 | 1.35E-09 |
| MYLK | -0.16132631 | 0.000236652 | 0.000564261 |
| MYLPF | -0.137099666 | 0.001817964 | 0.003690582 |
| MYNN | 0.208737105 | 1.77E-06 | 5.97E-06 |
| MYO15A | -0.346680055 | 5.46E-16 | 7.87E-15 |
| MYO15B | -0.325407231 | 3.62E-14 | 3.94E-13 |
| MYO18A | -0.224277715 | 2.70E-07 | 1.04E-06 |
| MYO19 | 0.218537647 | 5.50E-07 | 2.02E-06 |
| MYO1A | -0.109843765 | 0.012622498 | 0.02191613 |
| MYO1B | -0.113025972 | 0.010259293 | 0.018174487 |
| MYO1C | -0.138435763 | 0.001637465 | 0.003350016 |
| MYO1D | -0.217381994 | 6.33E-07 | 2.30E-06 |
| MYO1E | 0.175271985 | 6.36E-05 | 0.00016685 |
| MYO1F | -0.302163711 | 2.47E-12 | 2.02E-11 |
| MYO1G | -0.234913868 | 6.90E-08 | 2.91E-07 |
| MYO1H | -0.115392764 | 0.008765266 | 0.015714427 |
| MYO3A | -0.168064443 | 0.0001271 | 0.000318075 |
| MYO5A | -0.097136219 | 0.02750818 | 0.044524537 |
| MYO5C | -0.199226748 | 5.22E-06 | 1.63E-05 |
| MYO6 | -0.234044022 | 7.74E-08 | 3.23E-07 |
| MYO7B | -0.12772712 | 0.003690794 | 0.007085928 |
| MYO9A | -0.242308486 | 2.57E-08 | 1.15E-07 |
| MYO9B | -0.279506315 | 1.07E-10 | 6.85E-10 |
| MYOCD | -0.333593394 | 7.49E-15 | 9.02E-14 |
| MYOC | -0.431475878 | 9.12E-25 | 4.90E-23 |
| MYOF | -0.124523038 | 0.004654316 | 0.008794713 |
| MYOM1 | -0.136144133 | 0.00195804 | 0.003954861 |
| MYOM2 | -0.299718357 | 3.77E-12 | 3.00E-11 |
| MYOT | -0.09502656 | 0.031072973 | 0.049694878 |
| MYOZ1 | -0.340494755 | 1.91E-15 | 2.54E-14 |
| MYOZ2 | -0.098351263 | 0.025619227 | 0.041744283 |
| MYOZ3 | -0.23053223 | 1.22E-07 | 4.97E-07 |
| MYPN | 0.098590464 | 0.025260838 | 0.041203985 |
| MYRIP | -0.23893311 | 4.05E-08 | 1.77E-07 |
| MYSM1 | -0.146552356 | 0.000850702 | 0.001831125 |
| MYST1 | -0.214098519 | 9.40E-07 | 3.32E-06 |
| MYST2 | -0.183413197 | 2.82E-05 | 7.83E-05 |
| MYST4 | -0.188105418 | 1.73E-05 | 5.01E-05 |
| MZF1 | -0.336601305 | 4.15E-15 | 5.22E-14 |
| N4BP1 | -0.317391441 | 1.62E-13 | 1.59E-12 |
| N4BP2L1 | -0.464965907 | 5.51E-29 | 5.19E-27 |
| N4BP2L2 | -0.283636855 | 5.52E-11 | 3.68E-10 |
| N4BP2 | 0.157231785 | 0.000341249 | 0.000787995 |
| N4BP3 | -0.22042622 | 4.36E-07 | 1.63E-06 |
| N6AMT1 | -0.161438439 | 0.000234263 | 0.000559431 |
| N6AMT2 | 0.104878781 | 0.017272075 | 0.029141068 |
| NAA10 | 0.272521833 | 3.20E-10 | 1.91E-09 |
| NAA15 | 0.442971892 | 3.66E-26 | 2.43E-24 |
| NAA16 | -0.216432185 | 7.10E-07 | 2.55E-06 |
| NAA20 | 0.300357381 | 3.38E-12 | 2.70E-11 |
| NAA25 | 0.346568352 | 5.58E-16 | 8.03E-15 |
| NAA30 | 0.331657128 | 1.09E-14 | 1.28E-13 |
| NAA35 | 0.314689644 | 2.66E-13 | 2.54E-12 |
| NAA38 | 0.240348559 | 3.35E-08 | 1.48E-07 |
| NAA50 | 0.393070551 | 1.79E-20 | 4.97E-19 |
| NAAA | -0.13895622 | 0.00157172 | 0.003224092 |
| NAALAD2 | -0.255653845 | 3.96E-09 | 2.00E-08 |
| NAALADL1 | -0.233716522 | 8.07E-08 | 3.37E-07 |
| NAALADL2 | -0.125907309 | 0.004213158 | 0.008010575 |
| NAB2 | -0.165984129 | 0.000154386 | 0.000380737 |
| NACA2 | 0.166794788 | 0.000143157 | 0.000355148 |
| NACAD | -0.169665475 | 0.000109259 | 0.000276457 |
| NACAP1 | 0.145882237 | 0.000899112 | 0.001925994 |
| NACA | 0.235180909 | 6.66E-08 | 2.81E-07 |
| NADSYN1 | -0.187011477 | 1.94E-05 | 5.56E-05 |
| NAE1 | 0.273774505 | 2.63E-10 | 1.59E-09 |
| NAF1 | 0.183282943 | 2.85E-05 | 7.93E-05 |
| NAGA | -0.177246525 | 5.24E-05 | 0.000139314 |
| NAGK | -0.154241979 | 0.000443335 | 0.001004464 |
| NAGLU | -0.165254648 | 0.000165193 | 0.000405486 |
| NAGPA | -0.143998516 | 0.001049163 | 0.002222647 |
| NAGS | -0.100074913 | 0.02313203 | 0.038048306 |
| NAIF1 | 0.108451014 | 0.01379985 | 0.02379498 |
| NAIP | -0.275568379 | 1.99E-10 | 1.22E-09 |
| NALCN | -0.213544923 | 1.00E-06 | 3.53E-06 |
| NAMPT | 0.396408403 | 7.95E-21 | 2.35E-19 |
| NANOG | -0.156722754 | 0.000356916 | 0.000821516 |
| NANP | 0.208121312 | 1.90E-06 | 6.38E-06 |
| NANS | 0.163122804 | 0.00020098 | 0.000486049 |
| NAP1L1 | 0.109390612 | 0.01299545 | 0.022518686 |
| NAP1L2 | -0.261374768 | 1.72E-09 | 9.20E-09 |
| NAP1L3 | -0.202034801 | 3.81E-06 | 1.22E-05 |
| NAP1L5 | -0.249516942 | 9.48E-09 | 4.56E-08 |
| NAPA | -0.359823349 | 3.46E-17 | 5.97E-16 |
| NAPB | -0.208981754 | 1.72E-06 | 5.81E-06 |
| NAPRT1 | -0.128482787 | 0.003491699 | 0.006734736 |
| NAPSA | -0.340255773 | 2.01E-15 | 2.66E-14 |
| NAPSB | -0.399003276 | 4.21E-21 | 1.30E-19 |
| NARFL | -0.148171852 | 0.000743485 | 0.001619913 |
| NARS2 | 0.247578265 | 1.24E-08 | 5.86E-08 |
| NARS | 0.284080255 | 5.14E-11 | 3.44E-10 |
| NAT10 | 0.140608713 | 0.001378704 | 0.002859852 |
| NAT14 | -0.152660926 | 0.00050818 | 0.001137841 |
| NAT15 | -0.266552036 | 7.95E-10 | 4.49E-09 |
| NAT6 | -0.25886847 | 2.49E-09 | 1.29E-08 |
| NAT8L | 0.163816502 | 0.000188607 | 0.000458231 |
| NAV1 | 0.130975066 | 0.002902187 | 0.005681579 |
| NBEAL1 | -0.234367928 | 7.41E-08 | 3.11E-07 |
| NBEAL2 | -0.250199566 | 8.62E-09 | 4.17E-08 |
| NBEA | -0.204174817 | 2.99E-06 | 9.70E-06 |
| NBLA00301 | -0.150261875 | 0.000623588 | 0.001375597 |
| NBN | 0.362699464 | 1.86E-17 | 3.32E-16 |
| NBPF10 | -0.215687483 | 7.77E-07 | 2.77E-06 |
| NBPF14 | -0.236692388 | 5.46E-08 | 2.33E-07 |
| NBPF15 | -0.112570822 | 0.010571231 | 0.018672478 |
| NBPF16 | -0.105072089 | 0.017066266 | 0.028842524 |
| NBPF1 | -0.238638756 | 4.21E-08 | 1.83E-07 |
| NBPF22P | 0.105332903 | 0.016791986 | 0.028417427 |
| NBPF3 | -0.25399149 | 5.03E-09 | 2.51E-08 |
| NBPF4 | 0.168650718 | 0.000120271 | 0.000302082 |
| NBPF6 | 0.131646327 | 0.002759706 | 0.005423894 |
| NBPF7 | -0.160285167 | 0.000259952 | 0.000614829 |
| NBPF9 | -0.195341876 | 7.99E-06 | 2.43E-05 |
| NBR1 | -0.161083227 | 0.00024191 | 0.000575562 |
| NBR2 | -0.184179881 | 2.60E-05 | 7.29E-05 |
| NCALD | -0.305204883 | 1.45E-12 | 1.24E-11 |
| NCAM1 | -0.169692314 | 0.000108981 | 0.000275877 |
| NCAM2 | -0.231863878 | 1.03E-07 | 4.22E-07 |
| NCAPD2 | 0.393505186 | 1.61E-20 | 4.49E-19 |
| NCAPD3 | 0.253033934 | 5.77E-09 | 2.85E-08 |
| NCAPG2 | 0.411458166 | 1.85E-22 | 6.88E-21 |
| NCAPG | 0.530877898 | 8.74E-39 | 5.46E-36 |
| NCAPH2 | -0.108932944 | 0.013381948 | 0.023120268 |
| NCAPH | 0.48378139 | 1.44E-31 | 2.11E-29 |
| NCBP1 | 0.319405715 | 1.12E-13 | 1.13E-12 |
| NCBP2 | 0.189633403 | 1.48E-05 | 4.31E-05 |
| NCDN | -0.292863761 | 1.21E-11 | 8.93E-11 |
| NCF1B | -0.167276754 | 0.00013685 | 0.000340387 |
| NCF1C | -0.170860864 | 9.75E-05 | 0.000248651 |
| NCF1 | -0.168714961 | 0.000119544 | 0.00030037 |
| NCF2 | -0.162684416 | 0.000209188 | 0.00050466 |
| NCF4 | -0.293990981 | 1.00E-11 | 7.48E-11 |
| NCK1 | 0.204079153 | 3.02E-06 | 9.80E-06 |
| NCKAP1L | -0.228532149 | 1.58E-07 | 6.29E-07 |
| NCKAP1 | 0.291405648 | 1.54E-11 | 1.12E-10 |
| NCKAP5L | -0.301757437 | 2.65E-12 | 2.16E-11 |
| NCKAP5 | -0.150160595 | 0.000628958 | 0.001386218 |
| NCL | 0.247753267 | 1.21E-08 | 5.73E-08 |
| NCOA1 | -0.142814398 | 0.001154975 | 0.002429556 |
| NCOA7 | -0.159798525 | 0.000271563 | 0.000639793 |
| NCOR2 | -0.161581962 | 0.000231237 | 0.000552735 |
| NCR3 | -0.188536907 | 1.66E-05 | 4.80E-05 |
| NCRNA00032 | -0.162090504 | 0.000220809 | 0.000530154 |
| NCRNA00052 | 0.132872126 | 0.002515848 | 0.004981848 |
| NCRNA00085 | -0.311283341 | 4.93E-13 | 4.51E-12 |
| NCRNA00086 | -0.175191462 | 6.41E-05 | 0.000168038 |
| NCRNA00087 | -0.325695607 | 3.43E-14 | 3.75E-13 |
| NCRNA00092 | -0.34528531 | 7.26E-16 | 1.02E-14 |
| NCRNA00093 | -0.109457256 | 0.012939999 | 0.022430378 |
| NCRNA00094 | -0.159842574 | 0.000270493 | 0.000637722 |
| NCRNA00105 | -0.257981751 | 2.83E-09 | 1.46E-08 |
| NCRNA00107 | -0.243254659 | 2.26E-08 | 1.02E-07 |
| NCRNA00110 | -0.160511083 | 0.000254721 | 0.000603242 |
| NCRNA00113 | -0.13441186 | 0.002237429 | 0.004471726 |
| NCRNA00115 | -0.212275441 | 1.17E-06 | 4.05E-06 |
| NCRNA00119 | 0.100325668 | 0.022788205 | 0.037526005 |
| NCRNA00152 | 0.265960413 | 8.69E-10 | 4.88E-09 |
| NCRNA00161 | -0.120985924 | 0.005976995 | 0.011064942 |
| NCRNA00169 | -0.147877269 | 0.000762005 | 0.001655538 |
| NCRNA00171 | -0.200698987 | 4.43E-06 | 1.40E-05 |
| NCRNA00173 | -0.275665399 | 1.96E-10 | 1.21E-09 |
| NCRNA00174 | -0.354254886 | 1.13E-16 | 1.78E-15 |
| NCRNA00175 | -0.118416528 | 0.007140061 | 0.013029813 |
| NCRNA00181 | -0.166196785 | 0.000151363 | 0.000373974 |
| NCRNA00182 | -0.311545482 | 4.70E-13 | 4.31E-12 |
| NCRNA00188 | 0.187881197 | 1.77E-05 | 5.12E-05 |
| NCRNA00201 | -0.336833373 | 3.96E-15 | 5.01E-14 |
| NCRNA00202 | -0.289373875 | 2.16E-11 | 1.54E-10 |
| NCRNA00203 | -0.166929393 | 0.000141369 | 0.000350972 |
| NCRNA00204B | -0.241501971 | 2.86E-08 | 1.28E-07 |
| NCRNA00230B | 0.125395969 | 0.004371512 | 0.008294834 |
| NCSTN | 0.096835581 | 0.02799364 | 0.04524435 |
| NDC80 | 0.471264813 | 7.83E-30 | 8.28E-28 |
| NDFIP2 | 0.219151445 | 5.10E-07 | 1.88E-06 |
| NDN | -0.283900349 | 5.29E-11 | 3.54E-10 |
| NDOR1 | -0.098709598 | 0.025083961 | 0.040938859 |
| NDRG1 | 0.119179825 | 0.006775014 | 0.012419203 |
| NDRG2 | -0.438948448 | 1.14E-25 | 7.10E-24 |
| NDRG3 | 0.11089911 | 0.011790332 | 0.020587503 |
| NDST1 | -0.19445855 | 8.80E-06 | 2.66E-05 |
| NDST2 | -0.361378027 | 2.48E-17 | 4.34E-16 |
| NDUFA12 | 0.355557524 | 8.59E-17 | 1.38E-15 |
| NDUFA1 | 0.14031318 | 0.001411537 | 0.002922499 |
| NDUFA4 | 0.169233282 | 0.000113828 | 0.000286945 |
| NDUFA5 | 0.116187234 | 0.008309094 | 0.014974499 |
| NDUFA8 | 0.208113839 | 1.90E-06 | 6.38E-06 |
| NDUFA9 | 0.419558381 | 2.25E-23 | 9.76E-22 |
| NDUFAB1 | 0.278918275 | 1.17E-10 | 7.48E-10 |
| NDUFAF2 | 0.20554088 | 2.56E-06 | 8.40E-06 |
| NDUFAF4 | 0.294647671 | 8.95E-12 | 6.75E-11 |
| NDUFB11 | 0.152612337 | 0.000510306 | 0.00114209 |
| NDUFB1 | 0.149382104 | 0.000671686 | 0.001474376 |
| NDUFB2 | 0.097389836 | 0.027104294 | 0.043931286 |
| NDUFB3 | 0.257308393 | 3.12E-09 | 1.60E-08 |
| NDUFB4 | 0.2081366 | 1.90E-06 | 6.37E-06 |
| NDUFB5 | 0.238954662 | 4.04E-08 | 1.76E-07 |
| NDUFB6 | 0.227439913 | 1.81E-07 | 7.17E-07 |
| NDUFB8 | 0.18351713 | 2.79E-05 | 7.76E-05 |
| NDUFB9 | 0.285813592 | 3.88E-11 | 2.65E-10 |
| NDUFC1 | 0.122358128 | 0.005428278 | 0.010131705 |
| NDUFC2 | 0.188937659 | 1.59E-05 | 4.61E-05 |
| NDUFS1 | 0.334211512 | 6.64E-15 | 8.10E-14 |
| NDUFS2 | 0.167786186 | 0.000130467 | 0.000325931 |
| NDUFS3 | 0.124798386 | 0.004563377 | 0.008630219 |
| NDUFS4 | 0.154926805 | 0.000417712 | 0.000949639 |
| NDUFS5 | 0.121747703 | 0.005666517 | 0.010537992 |
| NDUFS6 | 0.161137891 | 0.000240718 | 0.000573068 |
| NDUFS7 | -0.11359616 | 0.009880057 | 0.017568062 |
| NDUFS8 | 0.116280218 | 0.008257112 | 0.01489156 |
| NDUFV2 | 0.239397988 | 3.80E-08 | 1.66E-07 |
| NEAT1 | -0.362217833 | 2.07E-17 | 3.66E-16 |
| NECAB1 | -0.222860255 | 3.23E-07 | 1.23E-06 |
| NECAB2 | 0.195582983 | 7.79E-06 | 2.37E-05 |
| NECAB3 | -0.17905528 | 4.38E-05 | 0.000117882 |
| NECAP1 | 0.226233008 | 2.11E-07 | 8.27E-07 |
| NECAP2 | -0.12007641 | 0.006367647 | 0.011724072 |
| NEDD1 | 0.34549264 | 6.96E-16 | 9.83E-15 |
| NEDD4L | -0.150096492 | 0.000632379 | 0.001393296 |
| NEDD8 | 0.330988271 | 1.24E-14 | 1.44E-13 |
| NEDD9 | -0.301100736 | 2.97E-12 | 2.40E-11 |
| NEGR1 | -0.241373497 | 2.91E-08 | 1.30E-07 |
| NEIL1 | -0.420596454 | 1.71E-23 | 7.52E-22 |
| NEIL3 | 0.528373991 | 2.26E-38 | 1.26E-35 |
| NEK10 | -0.146889252 | 0.000827285 | 0.001785142 |
| NEK11 | -0.234286134 | 7.49E-08 | 3.14E-07 |
| NEK1 | -0.182278383 | 3.16E-05 | 8.72E-05 |
| NEK2 | 0.58281384 | 3.53E-48 | 1.18E-44 |
| NEK4 | 0.142018922 | 0.001231489 | 0.002577488 |
| NEK5 | -0.173718643 | 7.40E-05 | 0.000192163 |
| NEK8 | -0.390169635 | 3.58E-20 | 9.60E-19 |
| NEK9 | -0.164425258 | 0.00017834 | 0.000435457 |
| NELL1 | -0.263248074 | 1.30E-09 | 7.10E-09 |
| NELL2 | -0.227567195 | 1.79E-07 | 7.06E-07 |
| NEO1 | -0.117686999 | 0.007505311 | 0.013641553 |
| NETO1 | 0.100008186 | 0.023224276 | 0.038184325 |
| NETO2 | 0.250784148 | 7.94E-09 | 3.85E-08 |
| NEU2 | 0.148233663 | 0.000739652 | 0.001612056 |
| NEURL3 | -0.156887478 | 0.000351774 | 0.00081024 |
| NEURL4 | -0.194028538 | 9.22E-06 | 2.78E-05 |
| NEUROD2 | -0.117080568 | 0.00782155 | 0.014169957 |
| NEUROG2 | 0.139318204 | 0.001527432 | 0.003143243 |
| NFAM1 | -0.288499473 | 2.50E-11 | 1.75E-10 |
| NFASC | -0.358882645 | 4.23E-17 | 7.17E-16 |
| NFAT5 | -0.293389588 | 1.11E-11 | 8.21E-11 |
| NFATC1 | -0.389483804 | 4.22E-20 | 1.12E-18 |
| NFATC2IP | -0.229959928 | 1.31E-07 | 5.32E-07 |
| NFATC2 | -0.238754121 | 4.15E-08 | 1.81E-07 |
| NFATC3 | -0.320968252 | 8.35E-14 | 8.54E-13 |
| NFATC4 | -0.122452753 | 0.005392166 | 0.010073709 |
| NFE2L1 | -0.125737919 | 0.004265032 | 0.00810356 |
| NFE2L2 | -0.196317791 | 7.19E-06 | 2.20E-05 |
| NFIA | -0.263710988 | 1.22E-09 | 6.67E-09 |
| NFIB | -0.171639611 | 9.05E-05 | 0.000231672 |
| NFIC | -0.187673887 | 1.81E-05 | 5.22E-05 |
| NFIL3 | 0.162038356 | 0.000221858 | 0.00053248 |
| NFIX | -0.509688458 | 2.13E-35 | 6.70E-33 |
| NFKB1 | -0.1285072 | 0.003485432 | 0.006725442 |
| NFKBID | -0.367823838 | 6.06E-18 | 1.17E-16 |
| NFKBIL2 | 0.218029658 | 5.85E-07 | 2.14E-06 |
| NFKBIZ | -0.221528952 | 3.81E-07 | 1.43E-06 |
| NFRKB | -0.105466598 | 0.016652891 | 0.028198745 |
| NFS1 | 0.100480874 | 0.022577619 | 0.037200679 |
| NFU1 | 0.229656224 | 1.37E-07 | 5.51E-07 |
| NFXL1 | 0.362839975 | 1.80E-17 | 3.23E-16 |
| NFYA | 0.121191851 | 0.005891593 | 0.010916951 |
| NFYB | 0.206408575 | 2.32E-06 | 7.66E-06 |
| NFYC | -0.221743602 | 3.71E-07 | 1.40E-06 |
| NGB | -0.106360921 | 0.015748119 | 0.026818897 |
| NGDN | 0.235778641 | 6.16E-08 | 2.61E-07 |
| NGEF | 0.133517844 | 0.00239545 | 0.004763257 |
| NGFR | -0.284680173 | 4.66E-11 | 3.15E-10 |
| NGF | -0.095089298 | 0.030961518 | 0.049536447 |
| NHEDC2 | 0.147772416 | 0.000768699 | 0.00166888 |
| NHEG1 | 0.113482961 | 0.009954341 | 0.01768756 |
| NHEJ1 | 0.099764265 | 0.023564199 | 0.038673225 |
| NHLH2 | -0.124947154 | 0.004514915 | 0.008545844 |
| NHLRC2 | 0.208011922 | 1.92E-06 | 6.45E-06 |
| NHLRC3 | -0.205614207 | 2.54E-06 | 8.33E-06 |
| NHLRC4 | -0.24413307 | 2.00E-08 | 9.16E-08 |
| NHP2L1 | 0.10964678 | 0.012783443 | 0.022170539 |
| NHP2 | 0.131503166 | 0.002789548 | 0.005478235 |
| NHSL2 | -0.336281062 | 4.42E-15 | 5.54E-14 |
| NHS | -0.21003316 | 1.52E-06 | 5.17E-06 |
| NICN1 | -0.413070219 | 1.22E-22 | 4.71E-21 |
| NID2 | 0.167930328 | 0.000128712 | 0.000321869 |
| NIF3L1 | 0.337557022 | 3.43E-15 | 4.39E-14 |
| NINJ1 | -0.200678641 | 4.44E-06 | 1.40E-05 |
| NINJ2 | -0.19765424 | 6.21E-06 | 1.92E-05 |
| NINL | -0.171400587 | 9.26E-05 | 0.000236801 |
| NIP7 | 0.43484852 | 3.60E-25 | 2.06E-23 |
| NIPA2 | 0.246383839 | 1.47E-08 | 6.85E-08 |
| NIPAL1 | 0.133382128 | 0.00242031 | 0.004808862 |
| NIPAL3 | -0.383021847 | 1.93E-19 | 4.67E-18 |
| NIPBL | -0.132527864 | 0.00258227 | 0.00510276 |
| NIPSNAP1 | 0.259354136 | 2.32E-09 | 1.21E-08 |
| NIPSNAP3B | -0.336846017 | 3.95E-15 | 5.00E-14 |
| NISCH | -0.498891214 | 9.20E-34 | 2.00E-31 |
| NIT2 | 0.196764251 | 6.84E-06 | 2.10E-05 |
| NKAIN1 | 0.216064334 | 7.42E-07 | 2.66E-06 |
| NKAIN3 | 0.177537047 | 5.09E-05 | 0.000135759 |
| NKAIN4 | 0.106342679 | 0.015766135 | 0.02684346 |
| NKAPL | -0.16066245 | 0.000251271 | 0.000596132 |
| NKD1 | -0.143224862 | 0.001117231 | 0.002356607 |
| NKD2 | -0.27223914 | 3.34E-10 | 1.99E-09 |
| NKIRAS1 | -0.109423272 | 0.012968248 | 0.022475449 |
| NKIRAS2 | 0.165554828 | 0.000160663 | 0.000395143 |
| NKRF | 0.24685778 | 1.37E-08 | 6.43E-08 |
| NKTR | -0.336608654 | 4.14E-15 | 5.22E-14 |
| NKX1-2 | 0.207777227 | 1.98E-06 | 6.61E-06 |
| NKX2-1 | -0.279803058 | 1.02E-10 | 6.57E-10 |
| NKX2-3 | 0.149951334 | 0.000640189 | 0.001409106 |
| NKX2-5 | 0.138811814 | 0.001589715 | 0.003258328 |
| NLE1 | 0.29478273 | 8.75E-12 | 6.61E-11 |
| NLGN2 | -0.096282904 | 0.028905312 | 0.046563457 |
| NLGN3 | -0.268597632 | 5.83E-10 | 3.36E-09 |
| NLGN4Y | 0.153965661 | 0.000454082 | 0.001026022 |
| NLN | 0.346792413 | 5.33E-16 | 7.71E-15 |
| NLRC3 | -0.28950537 | 2.11E-11 | 1.51E-10 |
| NLRC4 | -0.248465726 | 1.10E-08 | 5.22E-08 |
| NLRC5 | -0.135312721 | 0.002087885 | 0.004199888 |
| NLRP11 | 0.110147513 | 0.012377828 | 0.021527636 |
| NLRP14 | -0.144060204 | 0.001043904 | 0.002212678 |
| NLRP1 | -0.437431286 | 1.75E-25 | 1.06E-23 |
| NLRP3 | -0.295705965 | 7.49E-12 | 5.72E-11 |
| NLRP5 | 0.138255422 | 0.001660829 | 0.003394687 |
| NLRP6 | -0.151425131 | 0.00056489 | 0.001254418 |
| NLRP8 | 0.152301903 | 0.000524085 | 0.001170829 |
| NLRP9 | -0.323880345 | 4.84E-14 | 5.14E-13 |
| NLRX1 | -0.174136829 | 7.11E-05 | 0.000185063 |
| NMBR | -0.242114045 | 2.64E-08 | 1.18E-07 |
| NMB | 0.24026514 | 3.38E-08 | 1.50E-07 |
| NMD3 | 0.388883927 | 4.86E-20 | 1.27E-18 |
| NME1-NME2 | 0.242322724 | 2.56E-08 | 1.15E-07 |
| NME1 | 0.501738123 | 3.45E-34 | 8.38E-32 |
| NME2P1 | 0.327679617 | 2.35E-14 | 2.62E-13 |
| NME2 | 0.383376067 | 1.78E-19 | 4.33E-18 |
| NME3 | -0.202880191 | 3.46E-06 | 1.11E-05 |
| NME4 | 0.182524025 | 3.08E-05 | 8.53E-05 |
| NME5 | -0.236009302 | 5.97E-08 | 2.54E-07 |
| NME6 | 0.244782116 | 1.83E-08 | 8.42E-08 |
| NME7 | 0.140250678 | 0.001418572 | 0.002936152 |
| NMI | 0.136464714 | 0.00190998 | 0.003862472 |
| NMNAT1 | -0.108489034 | 0.013766469 | 0.023747664 |
| NMNAT2 | -0.138668006 | 0.001607823 | 0.003292741 |
| NMNAT3 | -0.275946119 | 1.88E-10 | 1.16E-09 |
| NMUR1 | -0.257326002 | 3.11E-09 | 1.59E-08 |
| NMUR2 | -0.184152301 | 2.61E-05 | 7.31E-05 |
| NMU | 0.1976507 | 6.21E-06 | 1.92E-05 |
| NNAT | -0.241480488 | 2.87E-08 | 1.28E-07 |
| NNMT | 0.170291205 | 0.000102949 | 0.000261559 |
| NOB1 | 0.260808211 | 1.87E-09 | 9.93E-09 |
| NOC2L | 0.116077358 | 0.008370894 | 0.015073642 |
| NOC3L | 0.279434352 | 1.08E-10 | 6.92E-10 |
| NOC4L | 0.15293702 | 0.000496256 | 0.001112752 |
| NOD1 | -0.443326238 | 3.31E-26 | 2.21E-24 |
| NOD2 | -0.205398318 | 2.60E-06 | 8.52E-06 |
| NODAL | -0.09991958 | 0.023347261 | 0.038364441 |
| NOG | -0.104391566 | 0.017800455 | 0.029971822 |
| NOL10 | 0.351368863 | 2.07E-16 | 3.14E-15 |
| NOL11 | 0.368219536 | 5.55E-18 | 1.08E-16 |
| NOL6 | 0.151913295 | 0.000541821 | 0.001206865 |
| NOL7 | 0.324607826 | 4.22E-14 | 4.54E-13 |
| NOLC1 | 0.390085139 | 3.65E-20 | 9.77E-19 |
| NONO | 0.204680202 | 2.82E-06 | 9.20E-06 |
| NOP10 | 0.32203594 | 6.84E-14 | 7.10E-13 |
| NOP14 | 0.271065025 | 4.00E-10 | 2.36E-09 |
| NOP16 | 0.334104602 | 6.78E-15 | 8.25E-14 |
| NOP2 | 0.368904528 | 4.77E-18 | 9.37E-17 |
| NOP56 | 0.342964107 | 1.16E-15 | 1.59E-14 |
| NOP58 | 0.314868823 | 2.57E-13 | 2.46E-12 |
| NOS1AP | -0.177853044 | 4.93E-05 | 0.000131904 |
| NOSTRIN | -0.220834472 | 4.15E-07 | 1.55E-06 |
| NOTCH1 | -0.253710715 | 5.24E-09 | 2.61E-08 |
| NOTCH2 | -0.173942101 | 7.25E-05 | 0.000188208 |
| NOTCH4 | -0.263052013 | 1.34E-09 | 7.29E-09 |
| NOTO | -0.156600369 | 0.000360782 | 0.000829226 |
| NOTUM | -0.096642548 | 0.02830921 | 0.045710033 |
| NOVA2 | -0.173101835 | 7.86E-05 | 0.000203076 |
| NOX1 | -0.121202996 | 0.005887002 | 0.010909457 |
| NOX4 | 0.152633503 | 0.000509379 | 0.001140143 |
| NOX5 | 0.107447251 | 0.014707311 | 0.025224793 |
| NOXA1 | -0.185636648 | 2.24E-05 | 6.34E-05 |
| NPC1 | -0.115049951 | 0.008968902 | 0.016052044 |
| NPC2 | -0.313445646 | 3.33E-13 | 3.14E-12 |
| NPDC1 | -0.196130812 | 7.34E-06 | 2.24E-05 |
| NPEPL1 | -0.259049256 | 2.42E-09 | 1.26E-08 |
| NPEPPS | 0.137973437 | 0.001697974 | 0.003467419 |
| NPFFR1 | -0.234014769 | 7.76E-08 | 3.25E-07 |
| NPFF | -0.265452234 | 9.38E-10 | 5.24E-09 |
| NPHP1 | -0.22340437 | 3.02E-07 | 1.15E-06 |
| NPHP3 | -0.3082677 | 8.45E-13 | 7.48E-12 |
| NPHP4 | -0.285557891 | 4.04E-11 | 2.75E-10 |
| NPHS1 | -0.106235166 | 0.01587268 | 0.02699882 |
| NPIPL3 | -0.292268847 | 1.34E-11 | 9.81E-11 |
| NPIP | -0.359562999 | 3.66E-17 | 6.27E-16 |
| NPL | -0.121095331 | 0.005931484 | 0.010983741 |
| NPM1 | 0.259782015 | 2.18E-09 | 1.14E-08 |
| NPM2 | -0.163272566 | 0.000198246 | 0.000480076 |
| NPM3 | 0.37705044 | 7.64E-19 | 1.69E-17 |
| NPNT | -0.254752295 | 4.51E-09 | 2.26E-08 |
| NPPA | -0.185889866 | 2.18E-05 | 6.19E-05 |
| NPR1 | -0.30766948 | 9.40E-13 | 8.27E-12 |
| NPR2 | -0.264322861 | 1.11E-09 | 6.14E-09 |
| NPTN | 0.166708579 | 0.000144314 | 0.00035784 |
| NPTX1 | -0.106690451 | 0.015425775 | 0.02631479 |
| NPTX2 | 0.100870734 | 0.022056073 | 0.036437456 |
| NPTXR | -0.231260548 | 1.11E-07 | 4.55E-07 |
| NPW | 0.211413002 | 1.29E-06 | 4.45E-06 |
| NPY1R | -0.160974874 | 0.000244289 | 0.000580808 |
| NPY6R | -0.283091221 | 6.03E-11 | 4.00E-10 |
| NQO1 | 0.207490798 | 2.04E-06 | 6.81E-06 |
| NQO2 | 0.114315011 | 0.009419723 | 0.016803339 |
| NR0B1 | 0.216652022 | 6.91E-07 | 2.49E-06 |
| NR0B2 | -0.252930446 | 5.85E-09 | 2.89E-08 |
| NR1D1 | -0.101328228 | 0.021457402 | 0.035542434 |
| NR1D2 | -0.148334957 | 0.00073341 | 0.001599324 |
| NR1H2 | -0.194840937 | 8.44E-06 | 2.56E-05 |
| NR1H3 | -0.149387063 | 0.000671406 | 0.001474084 |
| NR2C2AP | 0.12745049 | 0.003766214 | 0.007221015 |
| NR2C2 | -0.255800646 | 3.88E-09 | 1.96E-08 |
| NR2E3 | -0.229810646 | 1.34E-07 | 5.41E-07 |
| NR2F1 | -0.271462082 | 3.76E-10 | 2.23E-09 |
| NR2F2 | -0.097940163 | 0.026245417 | 0.042674425 |
| NR2F6 | 0.128652834 | 0.003448262 | 0.006658023 |
| NR3C2 | -0.475922272 | 1.80E-30 | 2.17E-28 |
| NR4A1 | -0.139887341 | 0.001460112 | 0.003014639 |
| NR4A3 | -0.1561688 | 0.000374727 | 0.000858064 |
| NR5A1 | 0.113494297 | 0.009946879 | 0.017675873 |
| NR5A2 | -0.10408467 | 0.018140483 | 0.030500671 |
| NRADDP | -0.237699342 | 4.77E-08 | 2.06E-07 |
| NRAP | -0.199221559 | 5.22E-06 | 1.63E-05 |
| NRARP | 0.249551546 | 9.44E-09 | 4.54E-08 |
| NRAS | 0.400245151 | 3.10E-21 | 9.78E-20 |
| NRBF2 | 0.323957773 | 4.77E-14 | 5.07E-13 |
| NRBP1 | 0.212277271 | 1.17E-06 | 4.05E-06 |
| NRBP2 | -0.195579119 | 7.79E-06 | 2.37E-05 |
| NRCAM | -0.095625807 | 0.030022288 | 0.048215128 |
| NRD1 | 0.155370285 | 0.000401862 | 0.000915584 |
| NRG2 | -0.257894702 | 2.87E-09 | 1.48E-08 |
| NRG3 | -0.157629207 | 0.000329465 | 0.000762812 |
| NRGN | -0.281376125 | 7.94E-11 | 5.19E-10 |
| NRIP1 | 0.245422181 | 1.68E-08 | 7.75E-08 |
| NRIP2 | -0.386764795 | 8.03E-20 | 2.03E-18 |
| NRM | 0.11229444 | 0.010764728 | 0.018984064 |
| NRN1L | -0.21949355 | 4.89E-07 | 1.81E-06 |
| NRN1 | -0.133894647 | 0.002327646 | 0.004637659 |
| NRXN1 | -0.143105246 | 0.001128111 | 0.002376798 |
| NRXN2 | -0.331534037 | 1.12E-14 | 1.31E-13 |
| NRXN3 | -0.214747597 | 8.70E-07 | 3.08E-06 |
| NSD1 | -0.120725768 | 0.006086477 | 0.011244709 |
| NSDHL | 0.24046896 | 3.29E-08 | 1.46E-07 |
| NSF | 0.13773615 | 0.00172982 | 0.003525608 |
| NSL1 | 0.208675515 | 1.78E-06 | 6.01E-06 |
| NSMAF | 0.13264048 | 0.002560368 | 0.005062483 |
| NSMCE2 | 0.28081919 | 8.68E-11 | 5.65E-10 |
| NSUN2 | 0.224241845 | 2.72E-07 | 1.04E-06 |
| NSUN5P1 | -0.210853206 | 1.38E-06 | 4.74E-06 |
| NSUN5P2 | -0.20056989 | 4.49E-06 | 1.42E-05 |
| NSUN5 | 0.107018518 | 0.015110629 | 0.025845562 |
| NSUN6 | -0.09585362 | 0.029630918 | 0.047647851 |
| NSUN7 | -0.199020454 | 5.34E-06 | 1.67E-05 |
| NT5C1A | -0.28294377 | 6.17E-11 | 4.09E-10 |
| NT5C1B | -0.188140563 | 1.73E-05 | 4.99E-05 |
| NT5C3L | 0.195583076 | 7.79E-06 | 2.37E-05 |
| NT5C3 | 0.335561768 | 5.09E-15 | 6.33E-14 |
| NT5DC1 | -0.148527556 | 0.000721677 | 0.001575802 |
| NT5DC3 | 0.155729148 | 0.000389448 | 0.000889531 |
| NTF3 | -0.157413737 | 0.000335806 | 0.000776276 |
| NTF4 | -0.355804425 | 8.15E-17 | 1.32E-15 |
| NTN1 | -0.172808536 | 8.09E-05 | 0.000208431 |
| NTN3 | -0.110386161 | 0.012188544 | 0.021232754 |
| NTN4 | -0.286877648 | 3.26E-11 | 2.25E-10 |
| NTN5 | -0.315046406 | 2.49E-13 | 2.39E-12 |
| NTNG1 | -0.167564614 | 0.000133208 | 0.000331907 |
| NTRK1 | -0.273617087 | 2.70E-10 | 1.63E-09 |
| NTRK2 | -0.306119372 | 1.24E-12 | 1.07E-11 |
| NTRK3 | -0.368170992 | 5.61E-18 | 1.09E-16 |
| NTSR1 | 0.186570644 | 2.03E-05 | 5.80E-05 |
| NTS | 0.184882836 | 2.42E-05 | 6.81E-05 |
| NUAK2 | -0.165704909 | 0.000158442 | 0.000390017 |
| NUBP1 | -0.190870398 | 1.29E-05 | 3.81E-05 |
| NUBPL | 0.205215635 | 2.66E-06 | 8.69E-06 |
| NUCB1 | -0.272719267 | 3.10E-10 | 1.85E-09 |
| NUDCD1 | 0.459969335 | 2.51E-28 | 2.13E-26 |
| NUDCD2 | 0.182961498 | 2.95E-05 | 8.19E-05 |
| NUDT12 | -0.167589737 | 0.000132894 | 0.000331167 |
| NUDT13 | -0.155455062 | 0.000398896 | 0.000909449 |
| NUDT14 | -0.126122608 | 0.00414805 | 0.007898564 |
| NUDT15 | 0.383361331 | 1.78E-19 | 4.34E-18 |
| NUDT16L1 | -0.186750284 | 2.00E-05 | 5.71E-05 |
| NUDT16 | -0.320291618 | 9.48E-14 | 9.62E-13 |
| NUDT17 | -0.111759971 | 0.01114784 | 0.019590559 |
| NUDT18 | -0.183870302 | 2.69E-05 | 7.51E-05 |
| NUDT19 | 0.181099997 | 3.56E-05 | 9.76E-05 |
| NUDT1 | 0.278765238 | 1.20E-10 | 7.65E-10 |
| NUDT21 | 0.186606308 | 2.03E-05 | 5.78E-05 |
| NUDT2 | 0.212969779 | 1.08E-06 | 3.75E-06 |
| NUDT3 | 0.303166887 | 2.08E-12 | 1.72E-11 |
| NUDT5 | 0.321329622 | 7.81E-14 | 8.04E-13 |
| NUDT7 | -0.187970115 | 1.76E-05 | 5.07E-05 |
| NUDT9 | 0.136788716 | 0.001862506 | 0.00377334 |
| NUF2 | 0.532895157 | 4.04E-39 | 2.72E-36 |
| NUFIP1 | 0.309609404 | 6.65E-13 | 5.99E-12 |
| NUMA1 | -0.293003587 | 1.18E-11 | 8.73E-11 |
| NUP107 | 0.319618483 | 1.07E-13 | 1.09E-12 |
| NUP133 | 0.113972519 | 0.009636612 | 0.017170316 |
| NUP153 | 0.211459275 | 1.29E-06 | 4.43E-06 |
| NUP155 | 0.293077005 | 1.17E-11 | 8.63E-11 |
| NUP160 | 0.11598973 | 0.008420474 | 0.015152002 |
| NUP205 | 0.321725497 | 7.25E-14 | 7.51E-13 |
| NUP210L | -0.221478924 | 3.83E-07 | 1.44E-06 |
| NUP214 | -0.106376868 | 0.015732385 | 0.026796669 |
| NUP35 | 0.346493508 | 5.67E-16 | 8.13E-15 |
| NUP37 | 0.531287221 | 7.48E-39 | 4.82E-36 |
| NUP50 | 0.128571579 | 0.003468956 | 0.006697333 |
| NUP54 | 0.32155548 | 7.49E-14 | 7.74E-13 |
| NUP62CL | 0.155038579 | 0.000413663 | 0.000941291 |
| NUP62 | 0.103118633 | 0.019248104 | 0.032186991 |
| NUP85 | 0.187144643 | 1.92E-05 | 5.49E-05 |
| NUP88 | 0.204755551 | 2.80E-06 | 9.13E-06 |
| NUP93 | 0.219216579 | 5.06E-07 | 1.87E-06 |
| NUP98 | 0.152148212 | 0.000531034 | 0.001185293 |
| NUPL2 | 0.141168526 | 0.001318428 | 0.002743362 |
| NUPR1 | -0.220140852 | 4.52E-07 | 1.68E-06 |
| NUS1 | 0.314452552 | 2.78E-13 | 2.64E-12 |
| NUSAP1 | 0.485390995 | 8.49E-32 | 1.31E-29 |
| NUTF2 | 0.30388153 | 1.83E-12 | 1.54E-11 |
| NWD1 | -0.351764137 | 1.91E-16 | 2.91E-15 |
| NXF1 | -0.353608089 | 1.30E-16 | 2.03E-15 |
| NXF2B | -0.160383812 | 0.000257656 | 0.000609903 |
| NXF2 | -0.111464263 | 0.011364953 | 0.0199493 |
| NXF3 | -0.232629719 | 9.30E-08 | 3.85E-07 |
| NXF5 | -0.124675217 | 0.004603856 | 0.008702655 |
| NXNL1 | -0.18063069 | 3.74E-05 | 0.000101862 |
| NXN | -0.191587647 | 1.20E-05 | 3.55E-05 |
| NXPH1 | 0.095410819 | 0.03039568 | 0.048731521 |
| NXPH3 | -0.342527068 | 1.27E-15 | 1.73E-14 |
| NXPH4 | 0.123794775 | 0.004902777 | 0.009225824 |
| NXT1 | 0.234331806 | 7.45E-08 | 3.12E-07 |
| NXT2 | 0.139051009 | 0.001560011 | 0.003203688 |
| NYNRIN | -0.216598015 | 6.96E-07 | 2.50E-06 |
| NYX | 0.106516512 | 0.015595193 | 0.026572049 |
| OAS1 | 0.161321821 | 0.000236748 | 0.000564422 |
| OAS3 | 0.104747765 | 0.017412795 | 0.029356187 |
| OAT | 0.166262841 | 0.000150436 | 0.000371912 |
| OAZ2 | -0.121665464 | 0.005699322 | 0.010592101 |
| OBFC1 | -0.135372365 | 0.002078315 | 0.004181735 |
| OBFC2B | 0.426442996 | 3.58E-24 | 1.79E-22 |
| OBP2A | 0.19049032 | 1.35E-05 | 3.96E-05 |
| OBP2B | 0.166372016 | 0.000148914 | 0.000368424 |
| OBSCN | -0.24319785 | 2.27E-08 | 1.03E-07 |
| OBSL1 | -0.151160285 | 0.000577784 | 0.001281486 |
| OCEL1 | -0.105369024 | 0.016754306 | 0.028358462 |
| OCIAD1 | 0.20699142 | 2.17E-06 | 7.19E-06 |
| OCIAD2 | 0.143105501 | 0.001128088 | 0.002376798 |
| OCLN | -0.286459421 | 3.49E-11 | 2.40E-10 |
| OCM | -0.223416204 | 3.01E-07 | 1.15E-06 |
| OCRL | 0.124067402 | 0.004808397 | 0.009064439 |
| ODAM | -0.241628935 | 2.81E-08 | 1.26E-07 |
| ODC1 | 0.225037726 | 2.46E-07 | 9.51E-07 |
| ODF1 | -0.116668721 | 0.008043051 | 0.014546191 |
| ODF3B | -0.254136691 | 4.93E-09 | 2.46E-08 |
| ODF3L1 | -0.158611516 | 0.000301949 | 0.000705065 |
| ODZ2 | -0.188740094 | 1.62E-05 | 4.70E-05 |
| OFD1 | -0.33331233 | 7.91E-15 | 9.48E-14 |
| OGDH | -0.11533528 | 0.008799123 | 0.015770881 |
| OGFOD1 | 0.226451153 | 2.06E-07 | 8.07E-07 |
| OGFOD2 | -0.133563565 | 0.002387128 | 0.004747653 |
| OGFRL1 | -0.097979351 | 0.026185162 | 0.042590042 |
| OGFR | -0.217013273 | 6.62E-07 | 2.39E-06 |
| OGN | -0.314487618 | 2.76E-13 | 2.62E-12 |
| OGT | -0.284471297 | 4.82E-11 | 3.25E-10 |
| OIP5 | 0.524942749 | 8.21E-38 | 4.10E-35 |
| OIT3 | -0.140513561 | 0.001389198 | 0.002879528 |
| OLA1 | 0.46689879 | 3.04E-29 | 2.96E-27 |
| OLAH | 0.159811364 | 0.000271251 | 0.000639208 |
| OLFM1 | -0.403234615 | 1.48E-21 | 4.93E-20 |
| OLFML1 | -0.225448947 | 2.33E-07 | 9.06E-07 |
| OLFML2B | 0.102370959 | 0.020145376 | 0.033552685 |
| OLFML3 | -0.167464284 | 0.000134467 | 0.000334751 |
| OLIG1 | -0.267307953 | 7.09E-10 | 4.03E-09 |
| OLR1 | -0.263540997 | 1.25E-09 | 6.81E-09 |
| OMA1 | -0.174390254 | 6.94E-05 | 0.000180906 |
| OMD | -0.095052925 | 0.031026093 | 0.049629466 |
| OMG | -0.140445113 | 0.001396792 | 0.002894068 |
| OOEP | 0.116519448 | 0.008124706 | 0.014675378 |
| OPA1 | 0.255180643 | 4.24E-09 | 2.14E-08 |
| OPHN1 | -0.249236929 | 9.86E-09 | 4.73E-08 |
| OPN3 | 0.358768215 | 4.34E-17 | 7.33E-16 |
| OPRK1 | -0.172374195 | 8.43E-05 | 0.000216734 |
| OPRL1 | -0.149007353 | 0.0006932 | 0.001518598 |
| OR10A3 | -0.115114773 | 0.008930078 | 0.015991255 |
| OR10AD1 | -0.227930733 | 1.70E-07 | 6.76E-07 |
| OR10V1 | -0.150086661 | 0.000632905 | 0.001394302 |
| OR13A1 | -0.153867091 | 0.000457974 | 0.001033647 |
| OR13C2 | -0.110159554 | 0.012368216 | 0.021513872 |
| OR13C5 | -0.103515909 | 0.018785663 | 0.03147953 |
| OR13J1 | -0.199243879 | 5.21E-06 | 1.63E-05 |
| OR1B1 | -0.108782283 | 0.013511378 | 0.023327755 |
| OR1F1 | 0.268891148 | 5.58E-10 | 3.22E-09 |
| OR1F2P | 0.157679345 | 0.000328006 | 0.000759961 |
| OR1K1 | -0.106552818 | 0.015559696 | 0.02651835 |
| OR1L8 | -0.172216542 | 8.56E-05 | 0.000219796 |
| OR2A1 | -0.267869007 | 6.52E-10 | 3.73E-09 |
| OR2A4 | -0.129009725 | 0.003358688 | 0.00650077 |
| OR2A7 | -0.110349085 | 0.012217782 | 0.021281833 |
| OR2A9P | -0.134942642 | 0.002148176 | 0.004310153 |
| OR2B11 | -0.145551844 | 0.000923906 | 0.00197614 |
| OR2B6 | 0.154863638 | 0.000420016 | 0.000954661 |
| OR2C1 | -0.148013453 | 0.000753391 | 0.001639496 |
| OR2K2 | -0.107581927 | 0.014582587 | 0.025028057 |
| OR2M3 | 0.097569573 | 0.026821162 | 0.043535926 |
| OR2T10 | -0.143140331 | 0.00112491 | 0.002370803 |
| OR3A2 | -0.096966655 | 0.027781088 | 0.044926245 |
| OR4C6 | 0.129022515 | 0.003355517 | 0.00649715 |
| OR4D10 | -0.11151858 | 0.011324794 | 0.0198823 |
| OR4E2 | -0.096392262 | 0.028722924 | 0.046303348 |
| OR51B2 | 0.09969053 | 0.023667798 | 0.038817753 |
| OR51E1 | 0.342119182 | 1.38E-15 | 1.87E-14 |
| OR51E2 | 0.141452737 | 0.001288763 | 0.002688352 |
| OR51I1 | -0.150542505 | 0.000608931 | 0.001345194 |
| OR52A4 | 0.12729748 | 0.003808528 | 0.007293748 |
| OR52B6 | -0.147081613 | 0.000814183 | 0.001759721 |
| OR52D1 | -0.107145991 | 0.01498971 | 0.025673892 |
| OR52E2 | 0.17720647 | 5.26E-05 | 0.00013983 |
| OR52H1 | -0.142042797 | 0.001229126 | 0.002572812 |
| OR52K2 | -0.156343995 | 0.000369006 | 0.000846419 |
| OR52N4 | -0.324183173 | 4.57E-14 | 4.87E-13 |
| OR56A3 | 0.103220284 | 0.019128845 | 0.032000951 |
| OR56B1 | -0.304060477 | 1.78E-12 | 1.49E-11 |
| OR5AK2 | -0.143805582 | 0.00106577 | 0.002254722 |
| OR5AU1 | -0.114749292 | 0.009150954 | 0.016356011 |
| OR5C1 | -0.110631194 | 0.011996862 | 0.020920718 |
| OR5K1 | -0.14804559 | 0.000751371 | 0.001635813 |
| OR5K2 | -0.250031386 | 8.82E-09 | 4.26E-08 |
| OR5P2 | -0.100815549 | 0.022129258 | 0.036552318 |
| OR5P3 | -0.10402641 | 0.018205669 | 0.030599976 |
| OR6F1 | 0.119911749 | 0.006440772 | 0.011848887 |
| OR6K3 | -0.187683925 | 1.81E-05 | 5.22E-05 |
| OR6N1 | -0.113676351 | 0.009827733 | 0.017481243 |
| OR6S1 | -0.104911879 | 0.017236684 | 0.029086267 |
| OR7E37P | -0.146450328 | 0.000857913 | 0.001845654 |
| OR8B2 | 0.104741804 | 0.017419221 | 0.029364544 |
| OR8S1 | 0.135227095 | 0.002101696 | 0.004224102 |
| OR9G4 | -0.14754983 | 0.000783091 | 0.001697661 |
| ORAI3 | -0.161169638 | 0.000240029 | 0.000571494 |
| ORC1L | 0.473587688 | 3.78E-30 | 4.34E-28 |
| ORC2L | 0.259327867 | 2.33E-09 | 1.22E-08 |
| ORC4L | 0.19016432 | 1.39E-05 | 4.09E-05 |
| ORC5L | 0.381003695 | 3.08E-19 | 7.25E-18 |
| ORC6L | 0.442025707 | 4.80E-26 | 3.12E-24 |
| ORMDL1 | -0.155498311 | 0.000397392 | 0.000906328 |
| ORMDL2 | 0.313032352 | 3.59E-13 | 3.37E-12 |
| ORMDL3 | -0.429481065 | 1.57E-24 | 8.08E-23 |
| OS9 | -0.131583015 | 0.002772867 | 0.005446547 |
| OSBP2 | -0.163457569 | 0.000194917 | 0.000472472 |
| OSBPL10 | 0.145080925 | 0.000960338 | 0.002048366 |
| OSBPL1A | -0.120184804 | 0.006319917 | 0.0116437 |
| OSBPL2 | -0.113053336 | 0.010240802 | 0.018144947 |
| OSBPL6 | -0.228559201 | 1.57E-07 | 6.27E-07 |
| OSBPL7 | -0.356741218 | 6.69E-17 | 1.10E-15 |
| OSBPL8 | 0.196739811 | 6.86E-06 | 2.10E-05 |
| OSCAR | -0.250765071 | 7.96E-09 | 3.86E-08 |
| OSCP1 | -0.227244151 | 1.86E-07 | 7.34E-07 |
| OSGIN1 | 0.129980763 | 0.003125564 | 0.006084318 |
| OSGIN2 | 0.255958948 | 3.79E-09 | 1.92E-08 |
| OSR1 | -0.298991046 | 4.27E-12 | 3.36E-11 |
| OST4 | 0.178158799 | 4.79E-05 | 0.000128213 |
| OSTCL | 0.209564502 | 1.61E-06 | 5.45E-06 |
| OSTC | 0.416595355 | 4.88E-23 | 2.00E-21 |
| OTC | -0.303984871 | 1.80E-12 | 1.51E-11 |
| OTOA | -0.239679359 | 3.66E-08 | 1.61E-07 |
| OTUB1 | 0.116312403 | 0.008239187 | 0.014860574 |
| OTUB2 | 0.287219051 | 3.08E-11 | 2.14E-10 |
| OTUD1 | -0.275403741 | 2.04E-10 | 1.25E-09 |
| OTUD3 | -0.345754803 | 6.59E-16 | 9.35E-15 |
| OTUD5 | -0.196111178 | 7.35E-06 | 2.24E-05 |
| OTUD6B | 0.364646263 | 1.22E-17 | 2.24E-16 |
| OTUD7A | -0.198255576 | 5.81E-06 | 1.80E-05 |
| OTUD7B | -0.122430841 | 0.005400509 | 0.01008741 |
| OTX1 | 0.162683474 | 0.000209206 | 0.00050466 |
| OVCA2 | 0.24174917 | 2.77E-08 | 1.24E-07 |
| OVCH1 | -0.280779283 | 8.73E-11 | 5.68E-10 |
| OVCH2 | -0.26973263 | 4.91E-10 | 2.85E-09 |
| OVGP1 | -0.176391288 | 5.70E-05 | 0.000150429 |
| OXA1L | 0.142353667 | 0.00119874 | 0.002513154 |
| OXCT1 | -0.114970153 | 0.009016904 | 0.016127954 |
| OXER1 | -0.398525951 | 4.74E-21 | 1.45E-19 |
| OXNAD1 | 0.152156323 | 0.000530665 | 0.001184602 |
| OXSM | 0.182267302 | 3.17E-05 | 8.73E-05 |
| OXSR1 | 0.116227104 | 0.008286769 | 0.014938306 |
| P2RX1 | -0.229822073 | 1.34E-07 | 5.40E-07 |
| P2RX2 | -0.329569065 | 1.64E-14 | 1.87E-13 |
| P2RX4 | -0.143540407 | 0.001088991 | 0.002301412 |
| P2RX6 | -0.257936412 | 2.85E-09 | 1.47E-08 |
| P2RX7 | -0.264826846 | 1.03E-09 | 5.72E-09 |
| P2RY11 | -0.193267692 | 1.00E-05 | 3.00E-05 |
| P2RY12 | -0.300749249 | 3.16E-12 | 2.54E-11 |
| P2RY13 | -0.267054657 | 7.37E-10 | 4.18E-09 |
| P2RY14 | -0.214602842 | 8.85E-07 | 3.13E-06 |
| P2RY2 | -0.150228278 | 0.000625365 | 0.001379059 |
| P2RY6 | 0.108396366 | 0.013847957 | 0.023867634 |
| P2RY8 | -0.20172503 | 3.94E-06 | 1.26E-05 |
| P4HA1 | 0.3214024 | 7.70E-14 | 7.94E-13 |
| P4HA3 | 0.189147518 | 1.55E-05 | 4.52E-05 |
| P4HB | 0.154368899 | 0.000438479 | 0.000993911 |
| P4HTM | -0.273099823 | 2.92E-10 | 1.76E-09 |
| P704P | 0.129033215 | 0.003352867 | 0.006493276 |
| PA2G4P4 | 0.351194261 | 2.15E-16 | 3.25E-15 |
| PA2G4 | 0.492957538 | 6.89E-33 | 1.24E-30 |
| PABPC1L2A | -0.095634983 | 0.030006439 | 0.048193547 |
| PABPC1L2B | -0.111833477 | 0.011094444 | 0.019503584 |
| PABPC1L | -0.259308764 | 2.33E-09 | 1.22E-08 |
| PABPC1P2 | 0.136658452 | 0.001881461 | 0.003807883 |
| PABPC1 | 0.285500775 | 4.08E-11 | 2.78E-10 |
| PABPC3 | 0.294108656 | 9.80E-12 | 7.35E-11 |
| PABPC5 | -0.100598583 | 0.022419036 | 0.036969862 |
| PABPN1L | -0.103919217 | 0.018326141 | 0.030781757 |
| PACRGL | 0.137791416 | 0.001722354 | 0.003512182 |
| PACRG | -0.226712092 | 1.99E-07 | 7.82E-07 |
| PACS1 | 0.1530015 | 0.000493509 | 0.001107348 |
| PACS2 | -0.108029493 | 0.014174747 | 0.024393012 |
| PACSIN2 | -0.140660192 | 0.001373057 | 0.002849026 |
| PADI2 | -0.272972981 | 2.98E-10 | 1.79E-09 |
| PAEP | 0.27073895 | 4.21E-10 | 2.47E-09 |
| PAFAH1B1 | -0.097374375 | 0.027128769 | 0.043960262 |
| PAFAH1B2 | 0.207628016 | 2.01E-06 | 6.71E-06 |
| PAFAH1B3 | 0.248188365 | 1.14E-08 | 5.41E-08 |
| PAG1 | -0.268270082 | 6.13E-10 | 3.52E-09 |
| PAH | 0.203302755 | 3.30E-06 | 1.06E-05 |
| PAICS | 0.525317605 | 7.13E-38 | 3.66E-35 |
| PAIP1 | 0.265718379 | 9.02E-10 | 5.05E-09 |
| PAIP2B | -0.109806717 | 0.012652631 | 0.021964633 |
| PAK1IP1 | 0.489947499 | 1.89E-32 | 3.22E-30 |
| PAK1 | 0.159945389 | 0.000268009 | 0.000632389 |
| PAK2 | 0.261343378 | 1.73E-09 | 9.23E-09 |
| PAK6 | -0.238015842 | 4.58E-08 | 1.98E-07 |
| PAK7 | -0.250667893 | 8.07E-09 | 3.91E-08 |
| PALB2 | 0.145426452 | 0.00093348 | 0.001994697 |
| PALM3 | -0.282236169 | 6.92E-11 | 4.56E-10 |
| PALMD | -0.266110682 | 8.50E-10 | 4.78E-09 |
| PALM | -0.294429518 | 9.29E-12 | 6.99E-11 |
| PAMR1 | -0.158127625 | 0.000315224 | 0.000732895 |
| PAM | -0.124932041 | 0.004519817 | 0.008552693 |
| PAN2 | -0.260879881 | 1.85E-09 | 9.84E-09 |
| PAN3 | -0.172636071 | 8.22E-05 | 0.000211657 |
| PANK1 | 0.142284492 | 0.001205441 | 0.002526938 |
| PANK3 | 0.218797058 | 5.33E-07 | 1.96E-06 |
| PANK4 | -0.124396674 | 0.004696595 | 0.008868734 |
| PANX1 | 0.250378128 | 8.40E-09 | 4.07E-08 |
| PAOX | -0.254639537 | 4.59E-09 | 2.29E-08 |
| PAPD4 | -0.109753695 | 0.012695866 | 0.022030121 |
| PAPD5 | 0.100050473 | 0.023165779 | 0.038100683 |
| PAPD7 | -0.117962014 | 0.007365704 | 0.013413419 |
| PAPLN | -0.355401455 | 8.88E-17 | 1.42E-15 |
| PAPOLA | 0.298200294 | 4.89E-12 | 3.82E-11 |
| PAPSS1 | -0.142471689 | 0.001187386 | 0.002492225 |
| PAPSS2 | -0.19628929 | 7.21E-06 | 2.20E-05 |
| PAQR3 | 0.220462259 | 4.34E-07 | 1.62E-06 |
| PAQR6 | -0.116656213 | 0.008049865 | 0.01455588 |
| PAQR8 | -0.224726055 | 2.56E-07 | 9.87E-07 |
| PAQR9 | 0.232939073 | 8.94E-08 | 3.71E-07 |
| PAR-SN | -0.168414796 | 0.000122976 | 0.00030849 |
| PAR1 | -0.152039491 | 0.000536001 | 0.001195046 |
| PAR5 | -0.18946278 | 1.50E-05 | 4.38E-05 |
| PARD3B | -0.310408232 | 5.77E-13 | 5.25E-12 |
| PARD3 | 0.245955629 | 1.56E-08 | 7.23E-08 |
| PARD6B | -0.18043463 | 3.81E-05 | 0.000103781 |
| PARG | 0.228947965 | 1.50E-07 | 5.99E-07 |
| PARK2 | -0.185627315 | 2.24E-05 | 6.35E-05 |
| PARK7 | 0.129910955 | 0.00314182 | 0.006112724 |
| PARL | 0.22370353 | 2.91E-07 | 1.11E-06 |
| PARM1 | -0.25573497 | 3.92E-09 | 1.98E-08 |
| PARN | -0.174946506 | 6.57E-05 | 0.000171977 |
| PARP10 | -0.217488317 | 6.25E-07 | 2.27E-06 |
| PARP11 | -0.134219298 | 0.002270637 | 0.00453311 |
| PARP14 | -0.224017589 | 2.79E-07 | 1.07E-06 |
| PARP15 | -0.309674901 | 6.57E-13 | 5.92E-12 |
| PARP16 | -0.209725125 | 1.58E-06 | 5.35E-06 |
| PARP1 | 0.172637798 | 8.22E-05 | 0.000211649 |
| PARP2 | 0.229879026 | 1.33E-07 | 5.37E-07 |
| PARP3 | -0.306435812 | 1.17E-12 | 1.01E-11 |
| PARP4 | -0.110678571 | 0.011960109 | 0.020865729 |
| PARP6 | -0.149456899 | 0.000667467 | 0.00146608 |
| PARP8 | -0.219164906 | 5.09E-07 | 1.88E-06 |
| PARS2 | 0.137718166 | 0.001732255 | 0.003529852 |
| PARVA | -0.098794061 | 0.02495921 | 0.040748566 |
| PARVB | 0.118044186 | 0.007324443 | 0.013347273 |
| PARVG | -0.334347693 | 6.46E-15 | 7.90E-14 |
| PASD1 | 0.098880155 | 0.0248326 | 0.04056174 |
| PASK | -0.136957775 | 0.001838167 | 0.003728187 |
| PATE2 | 0.213277302 | 1.04E-06 | 3.63E-06 |
| PATL1 | 0.237753194 | 4.74E-08 | 2.04E-07 |
| PATL2 | -0.329299969 | 1.72E-14 | 1.96E-13 |
| PATZ1 | -0.102650525 | 0.019805701 | 0.033039254 |
| PAWR | 0.438845287 | 1.18E-25 | 7.29E-24 |
| PAX5 | -0.148794677 | 0.000705692 | 0.001544102 |
| PAX6 | -0.216904549 | 6.71E-07 | 2.42E-06 |
| PAX7 | -0.108483089 | 0.013771684 | 0.02375461 |
| PAX9 | 0.198549441 | 5.62E-06 | 1.75E-05 |
| PAXIP1 | 0.152539247 | 0.00051352 | 0.001148896 |
| PBK | 0.546685267 | 1.80E-41 | 1.91E-38 |
| PBOV1 | -0.226107432 | 2.15E-07 | 8.39E-07 |
| PBX2 | -0.146524355 | 0.000852675 | 0.001834978 |
| PBX3 | -0.112700096 | 0.010481788 | 0.018525951 |
| PBX4 | -0.322737102 | 6.00E-14 | 6.27E-13 |
| PBXIP1 | -0.410163917 | 2.57E-22 | 9.41E-21 |
| PCBD2 | -0.176613183 | 5.58E-05 | 0.000147486 |
| PCBP1 | 0.248762148 | 1.05E-08 | 5.03E-08 |
| PCBP2 | 0.32426597 | 4.50E-14 | 4.80E-13 |
| PCBP3 | -0.19437693 | 8.88E-06 | 2.68E-05 |
| PCCA | -0.14844793 | 0.000726507 | 0.001585654 |
| PCCB | 0.210972135 | 1.36E-06 | 4.67E-06 |
| PCDH10 | -0.160865427 | 0.000246714 | 0.000586225 |
| PCDH11X | -0.303714514 | 1.89E-12 | 1.58E-11 |
| PCDH12 | -0.126234606 | 0.004114542 | 0.007840735 |
| PCDH15 | -0.306179645 | 1.22E-12 | 1.06E-11 |
| PCDH17 | -0.128496651 | 0.003488139 | 0.006729168 |
| PCDH19 | -0.175266032 | 6.37E-05 | 0.000166903 |
| PCDH1 | -0.107535191 | 0.014625764 | 0.025095696 |
| PCDH20 | -0.396368451 | 8.03E-21 | 2.37E-19 |
| PCDH9 | -0.178674831 | 4.55E-05 | 0.000122232 |
| PCDHA10 | -0.206344621 | 2.33E-06 | 7.71E-06 |
| PCDHA12 | -0.115995043 | 0.008417461 | 0.015147943 |
| PCDHA1 | 0.132584928 | 0.002571151 | 0.005081792 |
| PCDHA3 | -0.17131642 | 9.33E-05 | 0.000238627 |
| PCDHAC2 | -0.171171757 | 9.46E-05 | 0.000241738 |
| PCDHB15 | -0.117047197 | 0.007839292 | 0.014199526 |
| PCDHB1 | -0.216779849 | 6.81E-07 | 2.45E-06 |
| PCDHB2 | 0.133968908 | 0.002314492 | 0.004612829 |
| PCDHB4 | -0.192642001 | 1.07E-05 | 3.19E-05 |
| PCDHB7 | -0.110642797 | 0.011987851 | 0.020910478 |
| PCDHB8 | 0.172372458 | 8.43E-05 | 0.000216743 |
| PCDHGA10 | -0.123064419 | 0.005163904 | 0.009679838 |
| PCDHGA11 | -0.103491137 | 0.018814212 | 0.031516801 |
| PCDHGA12 | -0.151878739 | 0.000543424 | 0.001209843 |
| PCDHGA1 | 0.128566142 | 0.003470345 | 0.00669872 |
| PCDHGA5 | -0.168376824 | 0.000123417 | 0.000309479 |
| PCDHGA6 | -0.20205334 | 3.80E-06 | 1.21E-05 |
| PCDHGA9 | -0.16248894 | 0.000212948 | 0.000513068 |
| PCDHGB1 | 0.15315818 | 0.000486892 | 0.00109373 |
| PCDHGB6 | -0.173587794 | 7.50E-05 | 0.000194366 |
| PCDHGB7 | -0.219902112 | 4.65E-07 | 1.73E-06 |
| PCDHGB8P | -0.100036637 | 0.023184906 | 0.038129002 |
| PCDHGC3 | -0.192982178 | 1.03E-05 | 3.08E-05 |
| PCDHGC4 | -0.095461313 | 0.030307625 | 0.048606982 |
| PCDP1 | -0.450295066 | 4.43E-27 | 3.28E-25 |
| PCF11 | -0.314363156 | 2.82E-13 | 2.68E-12 |
| PCGF3 | -0.162937096 | 0.00020442 | 0.000493829 |
| PCGF5 | 0.13312813 | 0.00246747 | 0.004891865 |
| PCGF6 | 0.351487099 | 2.02E-16 | 3.07E-15 |
| PCK1 | 0.227021459 | 1.91E-07 | 7.53E-07 |
| PCLO | -0.13778255 | 0.00172355 | 0.003513903 |
| PCM1 | -0.099325759 | 0.024186124 | 0.039596383 |
| PCMT1 | 0.196184378 | 7.29E-06 | 2.23E-05 |
| PCMTD2 | -0.153800125 | 0.000460636 | 0.001039538 |
| PCNA | 0.381699631 | 2.62E-19 | 6.27E-18 |
| PCNP | 0.164133688 | 0.00018319 | 0.000446427 |
| PCNT | -0.171114482 | 9.52E-05 | 0.000242973 |
| PCNXL2 | -0.146728068 | 0.000838413 | 0.00180691 |
| PCNX | -0.101577835 | 0.021136757 | 0.035060706 |
| PCP2 | -0.307039482 | 1.05E-12 | 9.18E-12 |
| PCP4L1 | -0.228373167 | 1.61E-07 | 6.41E-07 |
| PCSK1N | -0.173388462 | 7.65E-05 | 0.000197919 |
| PCSK1 | 0.299538319 | 3.89E-12 | 3.08E-11 |
| PCSK2 | -0.204293897 | 2.95E-06 | 9.58E-06 |
| PCSK4 | -0.220875738 | 4.13E-07 | 1.54E-06 |
| PCSK5 | -0.219859587 | 4.68E-07 | 1.74E-06 |
| PCSK7 | -0.237070551 | 5.19E-08 | 2.22E-07 |
| PCYOX1L | -0.329068684 | 1.80E-14 | 2.05E-13 |
| PCYT1A | 0.122821132 | 0.00525363 | 0.009833277 |
| PCYT1B | -0.170515896 | 0.000100769 | 0.00025632 |
| PDAP1 | 0.232835193 | 9.06E-08 | 3.76E-07 |
| PDCD10 | 0.313380688 | 3.37E-13 | 3.17E-12 |
| PDCD11 | 0.224640129 | 2.58E-07 | 9.97E-07 |
| PDCD2L | 0.334205234 | 6.65E-15 | 8.10E-14 |
| PDCD2 | 0.286887087 | 3.25E-11 | 2.25E-10 |
| PDCD4 | -0.13340225 | 0.002416609 | 0.004802464 |
| PDCD5 | 0.315373939 | 2.35E-13 | 2.26E-12 |
| PDCD6 | 0.194396284 | 8.86E-06 | 2.67E-05 |
| PDCL3 | 0.332001428 | 1.02E-14 | 1.20E-13 |
| PDCL | 0.169129228 | 0.000114954 | 0.000289566 |
[truncated: 218,349 more chars]
