# Supplementary material for: Potential role of glucosamine-phosphate N-acetyltransferase 1 in the development of lung adenocarcinoma
Source: Aging (Albany NY). 2021 Mar 3;13(5):7430–53. doi: 10.18632/aging.202604 (PMC7993716; doi:10.18632/aging.202604)
Supplement: Supplementary Tables 2, 3, 4, 5 and 6 [file aging-13-202604-s003.pdf]

SUPPLEMENTARY TABLES

Supplementary Table 2. GO term annotation of GNPNAT1 co-expressed genes.

Gene Set	Description	Size	Leading Edge Number	ES	NES	P Value	FDR
GO:0050000	chromosome localization	67	25	0.80986	2.4816	0	0
GO:0034502	protein localization to chromosome	68	32	0.79979	2.4853	0	0
GO:0007059	chromosome segregation	262	85	0.75424	2.8388	0	0
GO:0006399	tRNA metabolic process	162	83	0.72772	2.5441	0	0
GO:0006414	translational elongation	123	74	0.72479	2.4747	0	0
GO:0034470	ncRNA processing	312	154	0.71468	2.7154	0	0
GO:0016072	rRNA metabolic process	210	132	0.70636	2.5728	0	0
GO:0031123	RNA 3'-end processing	111	52	0.68634	2.3087	0	0
GO:0032200	telomere organization	136	46	0.68176	2.3781	0	0
GO:0048285	organelle fission	404	128	0.68041	2.6252	0	0
GO:0044839	cell cycle G2/M phase transition	192	62	0.6801	2.4743	0	0
GO:0051321	meiotic cell cycle	217	64	0.67269	2.4339	0	0
GO:0006413	translational initiation	184	104	0.66986	2.3769	0	0
GO:0022613	ribonucleoprotein complex biogenesis	386	186	0.66862	2.6023	0	0
GO:0006260	DNA replication	233	94	0.66663	2.4424	0	0

Supplementary Table 3. KEGG annotation of GNPNAT1 co-expressed genes.

Gene Set	Description	Size	Leading Edge Number	ES	NES	P Value	FDR
hsa04110	Cell cycle	118	55	0.76905	2.6367	0	0
hsa03010	Ribosome	131	89	0.7476	2.5746	0	0
hsa03050	Proteasome	44	32	0.86201	2.4789	0	0
hsa03013	RNA transport	158	69	0.68131	2.3982	0	0
hsa03008	Ribosome biogenesis in eukaryotes	70	39	0.75238	2.3585	0	0
hsa03040	Spliceosome	115	58	0.6893	2.355	0	0
hsa03030	DNA replication	36	20	0.83389	2.3474	0	0
hsa00970	Aminoacyl-tRNA biosynthesis	43	18	0.7767	2.2158	0	0
hsa00240	Pyrimidine metabolism	96	46	0.66328	2.1687	0	0
hsa03430	Mismatch repair	23	11	0.8588	2.1161	0	0
hsa03440	Homologous recombination	34	14	0.75934	2.0666	0	0
hsa01230	Biosynthesis of amino acids	69	26	0.63926	2.021	0	0
hsa04973	Carbohydrate digestion and absorption	41	12	-0.69606	-1.9057	0	0
hsa04640	Hematopoietic cell lineage	93	41	-0.62775	-1.9117	0	0
hsa05150	Staphylococcus aureus infection	52	21	-0.70608	-1.9824	0	0

Supplementary Table 4. Kinases enrichment of GNPNAT1 co-expressed genes.

Gene Set	Description	Size	Leading Edge Number	ES	NES	P Value	FDR
Kinase_CDK1	cyclin dependent kinase 1	258	84	0.67502	2.5115	0	0
Kinase_PLK1	polo like kinase 1	90	30	0.74839	2.4721	0	0
Kinase_AURKB	aurora kinase B	87	34	0.68299	2.1948	0	0
Kinase_CDK2	cyclin dependent kinase 2	278	90	0.58084	2.1651	0	0
Kinase_AURKA	aurora kinase A	46	14	0.69064	2.0178	0	0.00018945
Kinase_ATR	ATR serine/threonine kinase	66	24	0.66378	2.0493	0	0.00022103
Kinase_ATM	ATM serine/threonine kinase	123	38	0.61277	2.0683	0	0.00026523
Kinase_CHEK1	checkpoint kinase 1	130	40	0.57089	1.9762	0	0.00033154
Kinase_PLK3	polo like kinase 3	20	11	0.77975	1.9258	0	0.00073676
Kinase_CSNK2A1	casein kinase 2 alpha 1	256	78	0.50468	1.8799	0	0.0015914
Kinase_CHEK2	checkpoint kinase 2	27	10	0.70791	1.8659	0	0.0016879
Kinase_TTK	TTK protein kinase	12	8	0.84249	1.8095	0	0.0061888
Kinase_BRSK1	BR serine/threonine kinase 1	6	2	0.92299	1.7393	0	0.019484
Kinase_WEE1	WEE1 G2 checkpoint kinase	5	5	0.9652	1.7001	0	0.034765
Kinase_SYK	spleen associated tyrosine kinase	35	19	-0.6636	-1.7288	0	0.051696

Supplementary Table 5. miRNA enrichment of GNPNAT1 co-expressed genes.

Gene Set	Size	Leading Edge Number	ES	NES	P Value	FDR
GGGGCCC, MIR-296	67	27	-0.54235	-1.5749	0.0031696	0.085139
CCTGTGA, MIR-513	118	47	-0.50051	-1.5758	0.0014771	0.10451
CCCAGAG, MIR-326	141	49	-0.48688	-1.5844	0	0.12147
GAGCCTG, MIR-484	98	40	-0.53109	-1.6521	0	0.14007
AGCGCTT, MIR-518F, MIR-518E, MIR-518A	16	7	-0.69131	-1.5058	0.032787	0.1568
CAGGGTC, MIR-504	79	29	-0.52777	-1.5939	0.0014925	0.16032
AGGGCAG, MIR-18A	130	43	-0.44353	-1.4185	0.0061538	0.16062
CTTTGCA, MIR-527	222	63	-0.41799	-1.4216	0.0013793	0.16492
GGCCAGT, MIR-193A, MIR-193B	83	18	-0.47446	-1.426	0.014331	0.1673
CAGCACT, MIR-512-3P	143	51	-0.45013	-1.4577	0.0028694	0.16883
ATGCTGG, MIR-338	104	29	-0.48116	-1.5086	0.0047022	0.17491
CCAGGGG, MIR-331	83	28	-0.47609	-1.4274	0.016692	0.17648
GCACCTT, MIR-18A,MIR-18B	112	39	-0.47258	-1.4821	0.0014948	0.17974
ACAGGGT,	119	33	-0.45265	-1.4311	0.0088496	0.18111

MIR-10A, MIR-10B						
GAGCTGG, MIR-337	147	57	-0.45475	-1.4701	0.0044577	0.18214

Supplementary Table 6. Transcription factor enrichment of GNPNAT1 co-expressed genes.

Gene Set	Size	Leading Edge Number	ES	NES	P Value	FDR
V$E2F_Q6	211	81	0.59091	2.1599	0	0
V$E2F1_Q6	213	85	0.5917	2.1597	0	0
V$E2F_Q4	212	81	0.58525	2.1476	0	0
V$E2F1DP1_01	218	82	0.58088	2.128	0	0
V$E2F1DP2_01	218	82	0.58088	2.128	0	0
V$E2F4DP2_01	218	82	0.58088	2.128	0	0
V$E2F_02	218	82	0.58057	2.1245	0	0
V$E2F4DP1_01	220	82	0.57988	2.1119	0	0
V$ELK1_02	226	71	0.57447	2.1078	0	0
SGCGSSAAA_V$E2F1DP2_01	155	62	0.58411	2.0639	0	0
V$E2F_Q3	204	63	0.56112	2.0575	0	0
V$E2F_Q4_01	215	77	0.56263	2.0537	0	0
V$E2F1_Q3	225	75	0.5513	2.0241	0	0
V$E2F1DP1RB_01	210	61	0.54952	2.0132	0	0
V$E2F_Q3_01	215	68	0.54189	1.9856	0	0
