# Supplementary material for: Potential role of glucosamine-phosphate N-acetyltransferase 1 in the development of lung adenocarcinoma
Source: Aging (Albany NY). 2021 Mar 3;13(5):7430–53. doi: 10.18632/aging.202604 (PMC7993716; doi:10.18632/aging.202604)
Supplement: Supplementary Table 7 [file aging-13-202604-s004.docx]

**Supplementary Table 7. GNPNAT1 significant co-occurence genes.**

| Gene | Cytoband | Alteration | Altered group | Unaltered group | Log Ratio | p-Value | q-Value |
| --- | --- | --- | --- | --- | --- | --- | --- |
| DDHD1 | 14q22.1 | Amp | 14 (35.90%) | 0 (0.00%) | >10 | 2.73E-18 | 1.62E-14 |
| ERO1A | 14q22.1 | Amp | 14 (35.90%) | 0 (0.00%) | >10 | 2.73E-18 | 1.62E-14 |
| FERMT2 | 14q22.1 | Amp | 14 (35.90%) | 0 (0.00%) | >10 | 2.73E-18 | 1.62E-14 |
| GPR137C | 14q22.1 | Amp | 14 (35.90%) | 0 (0.00%) | >10 | 2.73E-18 | 1.62E-14 |
| PSMC6 | 14q22.1 | Amp | 14 (35.90%) | 0 (0.00%) | >10 | 2.73E-18 | 1.62E-14 |
| STYX | 14q22.1 | Amp | 14 (35.90%) | 0 (0.00%) | >10 | 2.73E-18 | 1.62E-14 |
| PTGER2 | 14q22.1 | Amp | 13 (33.33%) | 0 (0.00%) | >10 | 6.02E-17 | 3.07E-13 |
| TXNDC16 | 14q22.1 | Amp | 14 (35.90%) | 1 (0.21%) | 7.42 | 2.37E-16 | 1.06E-12 |
| PTGDR | 14q22.1 | Amp | 12 (30.77%) | 0 (0.00%) | >10 | 1.28E-15 | 5.07E-12 |
| BMP4 | 14q22.2 | Amp | 10 (25.64%) | 0 (0.00%) | >10 | 5.22E-13 | 1.86E-09 |
| FRMD6 | 14q22.1 | Amp | 12 (30.77%) | 3 (0.63%) | 5.61 | 2.34E-12 | 5.95E-09 |
| NID2 | 14q22.1 | Amp | 12 (30.77%) | 3 (0.63%) | 5.61 | 2.34E-12 | 5.95E-09 |
| RNA5SP385 | 14q22.1 | Amp | 12 (30.77%) | 3 (0.63%) | 5.61 | 2.34E-12 | 5.95E-09 |
| RTRAF | 14q22.1 | Amp | 12 (30.77%) | 3 (0.63%) | 5.61 | 2.34E-12 | 5.95E-09 |
| ABHD12B | 14q22.1 | Amp | 11 (28.21%) | 3 (0.63%) | 5.49 | 3.36E-11 | 5.70E-08 |
| GNG2 | 14q22.1 | Amp | 11 (28.21%) | 3 (0.63%) | 5.49 | 3.36E-11 | 5.70E-08 |
| LINC00519 | 14q21.3 | Amp | 11 (28.21%) | 3 (0.63%) | 5.49 | 3.36E-11 | 5.70E-08 |
| LINC00640 | 14q22.1 | Amp | 11 (28.21%) | 3 (0.63%) | 5.49 | 3.36E-11 | 5.70E-08 |
| PYGL | 14q22.1 | Amp | 11 (28.21%) | 3 (0.63%) | 5.49 | 3.36E-11 | 5.70E-08 |
| TMX1 | 14q22.1 | Amp | 11 (28.21%) | 3 (0.63%) | 5.49 | 3.36E-11 | 5.70E-08 |
| TRIM9 | 14q22.1 | Amp | 11 (28.21%) | 3 (0.63%) | 5.49 | 3.36E-11 | 5.70E-08 |
| CDKN3 | 14q22.2 | Amp | 9 (23.08%) | 1 (0.21%) | 6.78 | 3.01E-10 | 3.16E-07 |
| CGRRF1 | 14q22.2 | Amp | 9 (23.08%) | 1 (0.21%) | 6.78 | 3.01E-10 | 3.16E-07 |
| CNIH1 | 14q22.2 | Amp | 9 (23.08%) | 1 (0.21%) | 6.78 | 3.01E-10 | 3.16E-07 |
| DLGAP5 | 14q22.3 | Amp | 9 (23.08%) | 1 (0.21%) | 6.78 | 3.01E-10 | 3.16E-07 |
| FBXO34 | 14q22.3 | Amp | 9 (23.08%) | 1 (0.21%) | 6.78 | 3.01E-10 | 3.16E-07 |
| GCH1 | 14q22.2 | Amp | 9 (23.08%) | 1 (0.21%) | 6.78 | 3.01E-10 | 3.16E-07 |
| GMFB | 14q22.2 | Amp | 9 (23.08%) | 1 (0.21%) | 6.78 | 3.01E-10 | 3.16E-07 |
| LGALS3 | 14q22.3 | Amp | 9 (23.08%) | 1 (0.21%) | 6.78 | 3.01E-10 | 3.16E-07 |
| MAPK1IP1L | 14q22.3 | Amp | 9 (23.08%) | 1 (0.21%) | 6.78 | 3.01E-10 | 3.16E-07 |
| RNU6ATAC9P | 14q22.2 | Amp | 9 (23.08%) | 1 (0.21%) | 6.78 | 3.01E-10 | 3.16E-07 |
| SAMD4A | 14q22.2 | Amp | 9 (23.08%) | 1 (0.21%) | 6.78 | 3.01E-10 | 3.16E-07 |
| SOCS4 | 14q22.3 | Amp | 9 (23.08%) | 1 (0.21%) | 6.78 | 3.01E-10 | 3.16E-07 |
| WDHD1 | 14q22.2-q22.3 | Amp | 9 (23.08%) | 1 (0.21%) | 6.78 | 3.01E-10 | 3.16E-07 |
| NIN | 14q22.1 | Amp | 10 (25.64%) | 3 (0.63%) | 5.35 | 4.58E-10 | 4.67E-07 |
| RN7SL452P | 14q22.1 | Amp | 10 (25.64%) | 4 (0.84%) | 4.93 | 1.52E-09 | 1.47E-06 |
| SAV1 | 14q22.1 | Amp | 10 (25.64%) | 4 (0.84%) | 4.93 | 1.52E-09 | 1.47E-06 |
| LINC00520 | 14q22.3 | Amp | 7 (17.95%) | 0 (0.00%) | >10 | 3.39E-09 | 3.02E-06 |
| PELI2 | 14q22.3 | Amp | 7 (17.95%) | 0 (0.00%) | >10 | 3.39E-09 | 3.02E-06 |
| RPL13AP3 | 14q22.3 | Amp | 7 (17.95%) | 0 (0.00%) | >10 | 3.39E-09 | 3.02E-06 |
| ATL1 | 14q22.1 | Amp | 10 (25.64%) | 5 (1.05%) | 4.61 | 4.32E-09 | 3.30E-06 |
| CDKL1 | 14q21.3 | Amp | 10 (25.64%) | 5 (1.05%) | 4.61 | 4.32E-09 | 3.30E-06 |
| DMAC2L | 14q21.3 | Amp | 10 (25.64%) | 5 (1.05%) | 4.61 | 4.32E-09 | 3.30E-06 |
| L2HGDH | 14q21.3 | Amp | 10 (25.64%) | 5 (1.05%) | 4.61 | 4.32E-09 | 3.30E-06 |
| MAP4K5 | 14q22.1 | Amp | 10 (25.64%) | 5 (1.05%) | 4.61 | 4.32E-09 | 3.30E-06 |
| SOS2 | 14q21.3 | Amp | 10 (25.64%) | 5 (1.05%) | 4.61 | 4.32E-09 | 3.30E-06 |
| ATG14 | 14q22.3 | Amp | 8 (20.51%) | 1 (0.21%) | 6.61 | 4.44E-09 | 3.30E-06 |
| TBPL2 | 14q22.3 | Amp | 8 (20.51%) | 1 (0.21%) | 6.61 | 4.44E-09 | 3.30E-06 |
| RN7SL1 | 14q21.3 | Amp | 9 (23.08%) | 4 (0.84%) | 4.78 | 1.83E-08 | 1.28E-05 |
| RNA5SP384 | 14q21.3 | Amp | 9 (23.08%) | 4 (0.84%) | 4.78 | 1.83E-08 | 1.28E-05 |
| RPS29 | 14q21.3 | Amp | 9 (23.08%) | 4 (0.84%) | 4.78 | 1.83E-08 | 1.28E-05 |
| ARF6 | 14q21.3 | Amp | 9 (23.08%) | 5 (1.05%) | 4.46 | 4.84E-08 | 2.58E-05 |
| DNAAF2 | 14q21.3 | Amp | 9 (23.08%) | 5 (1.05%) | 4.46 | 4.84E-08 | 2.58E-05 |
| KLHDC1 | 14q21.3 | Amp | 9 (23.08%) | 5 (1.05%) | 4.46 | 4.84E-08 | 2.58E-05 |
| KLHDC2 | 14q21.3 | Amp | 9 (23.08%) | 5 (1.05%) | 4.46 | 4.84E-08 | 2.58E-05 |
| LINC01588 | 14q21.3 | Amp | 9 (23.08%) | 5 (1.05%) | 4.46 | 4.84E-08 | 2.58E-05 |
| LINC01599 | 14q21.3 | Amp | 9 (23.08%) | 5 (1.05%) | 4.46 | 4.84E-08 | 2.58E-05 |
| LRR1 | 14q21.3 | Amp | 9 (23.08%) | 5 (1.05%) | 4.46 | 4.84E-08 | 2.58E-05 |
| MGAT2 | 14q21.3 | Amp | 9 (23.08%) | 5 (1.05%) | 4.46 | 4.84E-08 | 2.58E-05 |
| NEMF | 14q21.3 | Amp | 9 (23.08%) | 5 (1.05%) | 4.46 | 4.84E-08 | 2.58E-05 |
| POLE2 | 14q21.3 | Amp | 9 (23.08%) | 5 (1.05%) | 4.46 | 4.84E-08 | 2.58E-05 |
| RN7SKP193 | 14q21.3 | Amp | 9 (23.08%) | 5 (1.05%) | 4.46 | 4.84E-08 | 2.58E-05 |
| RN7SL2 | 14q21.3 | Amp | 9 (23.08%) | 5 (1.05%) | 4.46 | 4.84E-08 | 2.58E-05 |
| RN7SL3 | 14q21.3 | Amp | 9 (23.08%) | 5 (1.05%) | 4.46 | 4.84E-08 | 2.58E-05 |
| RNU6ATAC30P | 14q21.3 | Amp | 9 (23.08%) | 5 (1.05%) | 4.46 | 4.84E-08 | 2.58E-05 |
| RPL36AL | 14q21.3 | Amp | 9 (23.08%) | 5 (1.05%) | 4.46 | 4.84E-08 | 2.58E-05 |
| VCPKMT | 14q21.3 | Amp | 9 (23.08%) | 5 (1.05%) | 4.46 | 4.84E-08 | 2.58E-05 |
| KTN1 | 14q22.3 | Amp | 7 (17.95%) | 1 (0.21%) | 6.42 | 6.27E-08 | 3.24E-05 |
| SLC35F4 | 14q22.3-q23.1 | Amp | 7 (17.95%) | 1 (0.21%) | 6.42 | 6.27E-08 | 3.24E-05 |
| MDGA2 | 14q21.3 | Amp | 9 (23.08%) | 6 (1.26%) | 4.2 | 1.15E-07 | 5.83E-05 |
| TMEM260 | 14q22.3 | Amp | 7 (17.95%) | 2 (0.42%) | 5.42 | 2.67E-07 | 1.34E-04 |
| LINC00648 | 14q21.3 | Amp | 8 (20.51%) | 5 (1.05%) | 4.29 | 5.07E-07 | 2.51E-04 |
| ARMH4 | 14q23.1 | Amp | 7 (17.95%) | 3 (0.63%) | 4.83 | 8.39E-07 | 3.82E-04 |
| AP5M1 | 14q22.3 | Amp | 6 (15.38%) | 1 (0.21%) | 6.2 | 8.47E-07 | 3.82E-04 |
| EXOC5 | 14q22.3 | Amp | 6 (15.38%) | 1 (0.21%) | 6.2 | 8.47E-07 | 3.82E-04 |
| OTX2 | 14q22.3 | Amp | 6 (15.38%) | 1 (0.21%) | 6.2 | 8.47E-07 | 3.82E-04 |
| RN7SKP99 | 14q23.1 | Amp | 6 (15.38%) | 1 (0.21%) | 6.2 | 8.47E-07 | 3.82E-04 |
| RN7SL461P | 14q22.3 | Amp | 6 (15.38%) | 1 (0.21%) | 6.2 | 8.47E-07 | 3.82E-04 |
| RTN1 | 14q23.1 | Amp | 6 (15.38%) | 1 (0.21%) | 6.2 | 8.47E-07 | 3.82E-04 |
| ACTR10 | 14q23.1 | Amp | 6 (15.38%) | 2 (0.42%) | 5.2 | 3.20E-06 | 1.36E-03 |
| CCDC198 | 14q22.3 | Amp | 6 (15.38%) | 2 (0.42%) | 5.2 | 3.20E-06 | 1.36E-03 |
| DAAM1 | 14q23.1 | Amp | 6 (15.38%) | 2 (0.42%) | 5.2 | 3.20E-06 | 1.36E-03 |
| NAA30 | 14q22.3 | Amp | 6 (15.38%) | 2 (0.42%) | 5.2 | 3.20E-06 | 1.36E-03 |
| RN7SL598P | 14q23.1 | Amp | 6 (15.38%) | 2 (0.42%) | 5.2 | 3.20E-06 | 1.36E-03 |
| ARID4A | 14q23.1 | Amp | 6 (15.38%) | 3 (0.63%) | 4.61 | 9.07E-06 | 3.40E-03 |
| C14ORF39 | 14q23.1 | Amp | 6 (15.38%) | 3 (0.63%) | 4.61 | 9.07E-06 | 3.40E-03 |
| CCDC175 | 14q23.1 | Amp | 6 (15.38%) | 3 (0.63%) | 4.61 | 9.07E-06 | 3.40E-03 |
| DACT1 | 14q23.1 | Amp | 6 (15.38%) | 3 (0.63%) | 4.61 | 9.07E-06 | 3.40E-03 |
| GPR135 | 14q23.1 | Amp | 6 (15.38%) | 3 (0.63%) | 4.61 | 9.07E-06 | 3.40E-03 |
| JKAMP | 14q23.1 | Amp | 6 (15.38%) | 3 (0.63%) | 4.61 | 9.07E-06 | 3.40E-03 |
| KIAA0586 | 14q23.1 | Amp | 6 (15.38%) | 3 (0.63%) | 4.61 | 9.07E-06 | 3.40E-03 |
| L3HYPDH | 14q23.1 | Amp | 6 (15.38%) | 3 (0.63%) | 4.61 | 9.07E-06 | 3.40E-03 |
| PSMA3 | 14q23.1 | Amp | 6 (15.38%) | 3 (0.63%) | 4.61 | 9.07E-06 | 3.40E-03 |
| TIMM9 | 14q23.1 | Amp | 6 (15.38%) | 3 (0.63%) | 4.61 | 9.07E-06 | 3.40E-03 |
| TOMM20L | 14q23.1 | Amp | 6 (15.38%) | 3 (0.63%) | 4.61 | 9.07E-06 | 3.40E-03 |
| RPL10L | 14q21.2 | Amp | 7 (17.95%) | 6 (1.26%) | 3.83 | 1.01E-05 | 3.76E-03 |
| PRKCH | 14q23.1 | Amp | 6 (15.38%) | 4 (0.84%) | 4.2 | 2.14E-05 | 7.87E-03 |
| LINC00517 | 14q21.1 | Amp | 14 (35.90%) | 45 (9.43%) | 1.93 | 2.38E-05 | 8.67E-03 |
| BAZ1A | 14q13.1-q13.2 | Amp | 14 (35.90%) | 46 (9.64%) | 1.9 | 2.95E-05 | 0.0106 |
| LINC00871 | 14q21.2 | Amp | 7 (17.95%) | 8 (1.68%) | 3.42 | 3.39E-05 | 0.0121 |
| LRRC9 | 14q23.1 | Amp | 5 (12.82%) | 2 (0.42%) | 4.93 | 3.61E-05 | 0.0124 |
| PPM1A | 14q23.1 | Amp | 5 (12.82%) | 2 (0.42%) | 4.93 | 3.61E-05 | 0.0124 |
| TRMT5 | 14q23.1 | Amp | 5 (12.82%) | 2 (0.42%) | 4.93 | 3.61E-05 | 0.0124 |
| FOXA1 | 14q21.1 | Amp | 14 (35.90%) | 47 (9.85%) | 1.87 | 3.64E-05 | 0.0124 |
| TTC6 | 14q21.1 | Amp | 14 (35.90%) | 47 (9.85%) | 1.87 | 3.64E-05 | 0.0124 |
| FANCM | 14q21.2 | Amp | 7 (17.95%) | 9 (1.89%) | 3.25 | 5.69E-05 | 0.0184 |
| FKBP3 | 14q21.2 | Amp | 7 (17.95%) | 9 (1.89%) | 3.25 | 5.69E-05 | 0.0184 |
| MIS18BP1 | 14q21.2 | Amp | 7 (17.95%) | 9 (1.89%) | 3.25 | 5.69E-05 | 0.0184 |
| PRPF39 | 14q21.2 | Amp | 7 (17.95%) | 9 (1.89%) | 3.25 | 5.69E-05 | 0.0184 |
| SNORD127 | 14q21.2 | Amp | 7 (17.95%) | 9 (1.89%) | 3.25 | 5.69E-05 | 0.0184 |
| MIPOL1 | 14q13.3-q21.1 | Amp | 14 (35.90%) | 51 (10.69%) | 1.75 | 8.02E-05 | 0.0258 |
| CFL2 | 14q13.1 | Amp | 13 (33.33%) | 44 (9.22%) | 1.85 | 8.34E-05 | 0.0265 |
| MNAT1 | 14q23.1 | Amp | 5 (12.82%) | 3 (0.63%) | 4.35 | 9.09E-05 | 0.0277 |
| SIX4 | 14q23.1 | Amp | 5 (12.82%) | 3 (0.63%) | 4.35 | 9.09E-05 | 0.0277 |
| SIX6 | 14q23.1 | Amp | 5 (12.82%) | 3 (0.63%) | 4.35 | 9.09E-05 | 0.0277 |
| SLC38A6 | 14q23.1 | Amp | 5 (12.82%) | 3 (0.63%) | 4.35 | 9.09E-05 | 0.0277 |
| TMEM30B | 14q23.1 | Amp | 5 (12.82%) | 3 (0.63%) | 4.35 | 9.09E-05 | 0.0277 |
| SLC25A21 | 14q13.3 | Amp | 15 (38.46%) | 60 (12.58%) | 1.61 | 1.03E-04 | 0.0311 |
| KLHL28 | 14q21.2 | Amp | 7 (17.95%) | 11 (2.31%) | 2.96 | 1.41E-04 | 0.042 |
| TOGARAM1 | 14q21.2 | Amp | 7 (17.95%) | 11 (2.31%) | 2.96 | 1.41E-04 | 0.042 |
