# Supplementary material for: Potential role of glucosamine-phosphate N-acetyltransferase 1 in the development of lung adenocarcinoma
Source: Aging (Albany NY). 2021 Mar 3;13(5):7430–53. doi: 10.18632/aging.202604 (PMC7993716; doi:10.18632/aging.202604)
Supplement: Supplementary Table 8 [file aging-13-202604-s005.pdf]

SUPPLEMENTARY TABLE

Supplementary Table 8. Functional annotation of GNPNAT1 AMP significant co-occurrence genes.

GO BP term	Total	Expected	Hits	P-Value	FDR
G1/S transition of mitotic cell cycle	209	1.14	6	0.000976	0.8
DNA_dependent DNA replication	121	0.661	4	0.00431	1
S phase of mitotic cell cycle	144	0.786	4	0.00793	1
S phase	153	0.835	4	0.00977	1
Regulation of mitotic cell cycle	351	1.92	6	0.0123	1
Regulation of cell cycle	886	4.84	10	0.0219	1
Chromatin remodeling	111	0.606	3	0.0228	1
Interphase of mitotic cell cycle	435	2.37	6	0.0314	1
Adenylate cyclase-activating G-protein coupled receptor signaling pathway	53	0.289	2	0.0339	1
Interphase	443	2.42	6	0.0339	1
Mitotic cell cycle	968	5.28	10	0.0372	1
Epithelial cell differentiation	339	1.85	5	0.0378	1
Negative regulation of cellular protein metabolic process	463	2.53	6	0.0407	1
DNA replication	346	1.89	5	0.0407	1
KEGG Pathway					
Hippo signaling pathway -multiple species	29	0.112	2	0.00553	0.963
Basal transcription factors	45	0.174	2	0.013	0.963
Proteasome	45	0.174	2	0.013	0.963
Nucleotide excision repair	47	0.182	2	0.0141	0.963
Insulin signaling pathway	137	0.531	3	0.0156	0.963
Ribosome	153	0.593	3	0.0208	0.963
Hippo signaling pathway	154	0.597	3	0.0212	0.963
Prolactin signaling pathway	70	0.271	2	0.0298	1
Fc gamma R-mediated phagocytosis	91	0.353	2	0.0481	1
Inflammatory mediator regulation of TRP channels	100	0.388	2	0.0569	1
